# Supplementary material for: TKI-mediated inhibition of NLRP1 inflammasome restores erythropoiesis in DBA syndrome
Source: EMBO Mol Med. 2026 Jan 9;18(2):702–24. doi: 10.1038/s44321-025-00368-3 (PMC12905221; doi:10.1038/s44321-025-00368-3)
Supplement: Supplementary file 1 — Table EV1 [file 44321_2025_368_MOESM1_ESM.docx]

**Table EV1.** Genetic tools used for CRISPR/Cas9 experiments with zebrafish embryo. The gene symbols followed the Zebrafish Nomenclature Guidelines (<http://zfin.org/zf_info/nomen.html>).

| Gene name  (ENSEMBL or ENA accession number) | crRNA Sequence (5’🡪3’) | Primer name | Primer sequence (5’🡪3’) | Primer use |
| --- | --- | --- | --- | --- |
| *nlrp1*  *(*ENSDARG00000088423) | tcacagaagactcaactagc | F  R | TGAGCCTGACTGAGCTCTTGA | PCR amplification of target sequence |
|  |  |  | AGCCAGTCCTGGTTACACTCT |  |
| *rps19*  *(ENSDARG00000030602)* | CGTCACCTGTACCTGCGTGG | F | GTGTCATCAGTTTCTGGTTTATGGT |  |
|  |  | R | GTCTACGCCCGCTGATGAAG |  |
| *zaka*  *(ENSDARG00000006978)* | AAGCCCCTCCAGACCTTTGA | F | TTGGCCATCATTTAATGGACCCGT |  |
|  |  | R | TTTTGGTTCAGTCGCCCAGCA |  |
