# Supplementary material for: TKI-mediated inhibition of NLRP1 inflammasome restores erythropoiesis in DBA syndrome
Source: EMBO Mol Med. 2026 Jan 9;18(2):702–24. doi: 10.1038/s44321-025-00368-3 (PMC12905221; doi:10.1038/s44321-025-00368-3)
Supplement: Supplementary file 2 — Table EV2 [file 44321_2025_368_MOESM2_ESM.docx]

**Table EV2.** Primers used for RT-qPCR.

| Gene name  (ENSEMBL accession number) | Specie | Primer name | Primer sequence (5’🡪3’) |
| --- | --- | --- | --- |
| *ACTB*  *(ENSG00000075624)* | human | F | GGCACCACACCTTCTACAATG |
|  |  | R | GTGGTGGTGAAGCTGTAGCC |
| *ALAS2*  *(ENSG00000158578)* | human | FH1_ALAS2 | Sigma-Aldrich  (Ref: KSPQ12012G) |
|  |  | BH1_ALAS2 |  |
| *ALAD*  *(ENSG00000148218)* | human | FH1_ALAD |  |
|  |  | BH1_ALAD |  |
| *FECH*  *(ENSG00000066926)* | human | FH1_FECH |  |
|  |  | BH1_FECH |  |
| *HMBS*  *(ENSG00000256269)* | human | FH1_HMBS |  |
|  |  | BH1_HMBS |  |
| *GYPC*  *(ENSG00000136732)* | human | FH1_GYPC |  |
|  |  | BH1_GYPC |  |
| *SLC4A1*  *(ENSG00000004939)* | human | FH1_SLC4A1 |  |
|  |  | BH1_SLC4A1 |  |
| *HBA1*  *(ENSG00000206172)* | human | FH1_HBA1 |  |
|  |  | BH1_HBA1 |  |
| *HBA2*  *(ENSG00000188536)* | human | FH1_HBA2 |  |
|  |  | BH1_HBA2 |  |
| *EPOR*  *(ENSG00000187266)* | human | FH1_EPOR |  |
|  |  | BH1_EPOR |  |
| *SOD3*  *(ENSG00000109610)* | human | FH1_SOD3 |  |
|  |  | BH1_SOD3 |  |
| *NFE2*  *(ENSG00000123405)* | human | FH1_NEF2 |  |
|  |  | BH1_NEF2 |  |
