# Supplementary material for: TKI-mediated inhibition of NLRP1 inflammasome restores erythropoiesis in DBA syndrome
Source: EMBO Mol Med. 2026 Jan 9;18(2):702–24. doi: 10.1038/s44321-025-00368-3 (PMC12905221; doi:10.1038/s44321-025-00368-3)
Supplement: Supplementary file 3 — Table EV3 [file 44321_2025_368_MOESM3_ESM.docx]

**Table EV3.** List of mutations of each DBAS patient who participated in this study

| Patient ID | Genetic variant | Gender |
| --- | --- | --- |
| DBA_01 | *RPS10: c.268_275del, p.Val90HisfsTer5* | *Female* |
| DBA_02 | *RPS26: c.328+1G>T* | *Male* |
| DBA_03 | *RPS19: c.356+1G>T* | *Female* |
| DBA_04 | *RPS10: c.337C>T, p.Arg113Ter* | *Female* |
| DBA_05 | *RPL11: c.6+2T>C* | *Male* |
| DBA_06 | *RPL11: c.372_385del, p.Ile125LeufsTer7* | *Female* |
| DBA_07 | *RPL35A: c.82_84insCTT* | *Male* |
| DBA_08 | *RPS19: c.388_389del, p.Asp130SerfsTer23* | *Male* |
| DBA_09 | *RPL5: c.175_176delGA, p.Asp59TyrfsTer53* | *Female* |
| DBA_95 | *RPS19: c.172+1G>A* | *No data* |
| DBA_102 | *RPS19: Mutation unavailable* | *No data* |
| DBA_103 | *RPS26: c.131_132del, p.Ile44SerfsTer11* | *No data* |
| DBA_104 | *RPL11: Mutation unavailable* | *No data* |
