# Supplementary figures and images for: TKI-mediated inhibition of NLRP1 inflammasome restores erythropoiesis in DBA syndrome (part 2 of 4)
Source: EMBO Mol Med. 2026 Jan 9;18(2):702–24. doi: 10.1038/s44321-025-00368-3 (PMC12905221; doi:10.1038/s44321-025-00368-3)

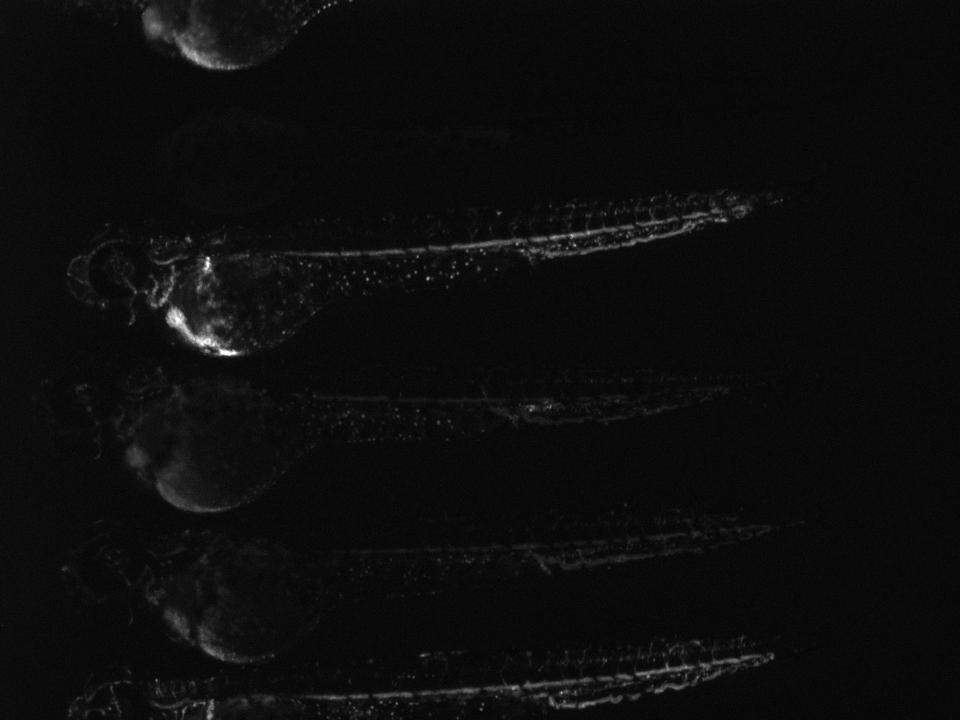

Supplement: Supplementary file 7 — Source data Fig. 3 [file 44321_2025_368_MOESM7_ESM.zip › FIGURE_3/3E/DMSO (9).tif]

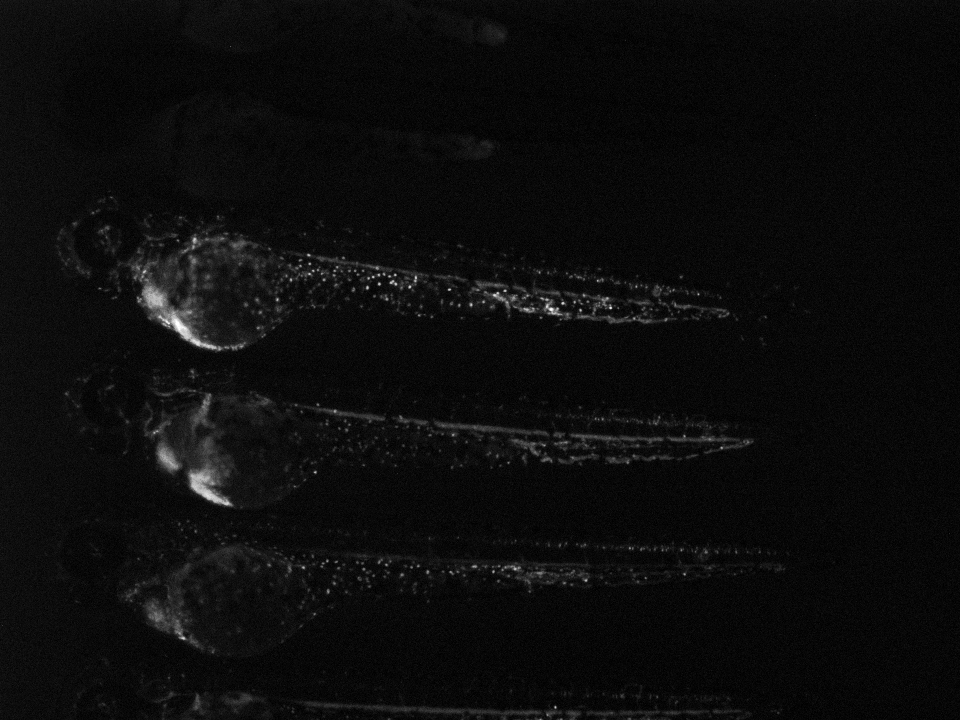

Supplement: Supplementary file 7 — Source data Fig. 3 [file 44321_2025_368_MOESM7_ESM.zip › FIGURE_3/3E/PONATINIB_01uM (1).tif]

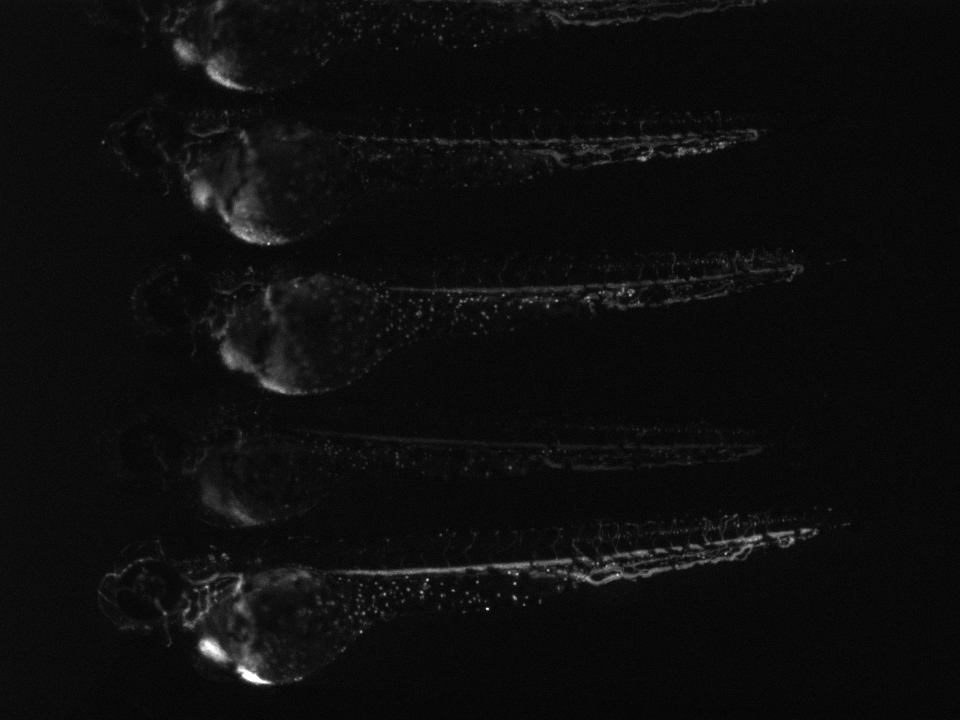

Supplement: Supplementary file 7 — Source data Fig. 3 [file 44321_2025_368_MOESM7_ESM.zip › FIGURE_3/3E/PONATINIB_01uM (10).tif]

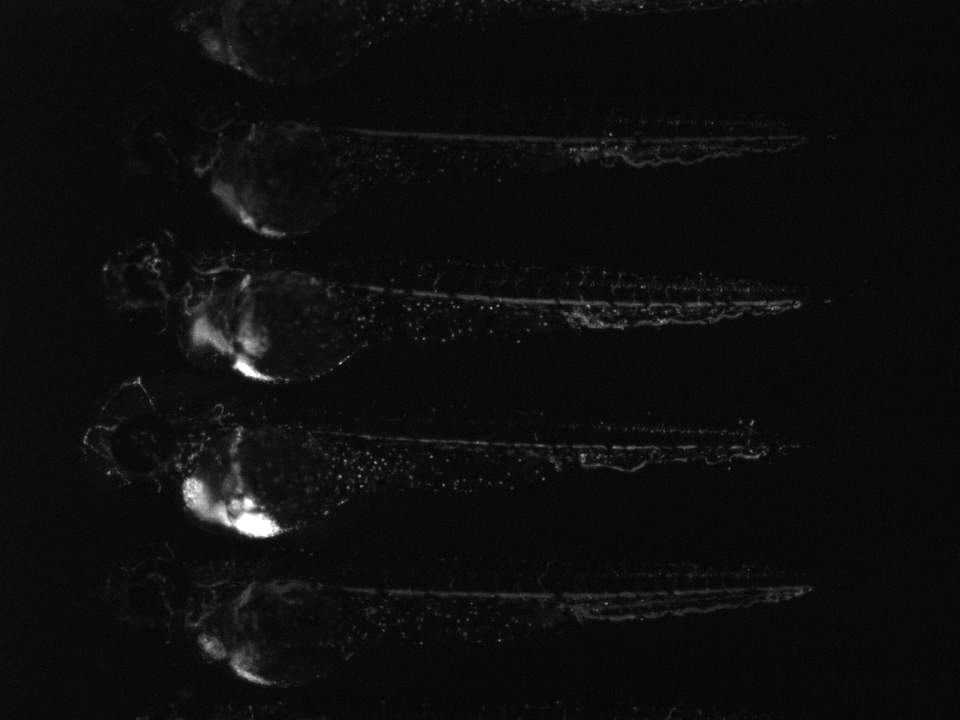

Supplement: Supplementary file 7 — Source data Fig. 3 [file 44321_2025_368_MOESM7_ESM.zip › FIGURE_3/3E/PONATINIB_01uM (11).tif]

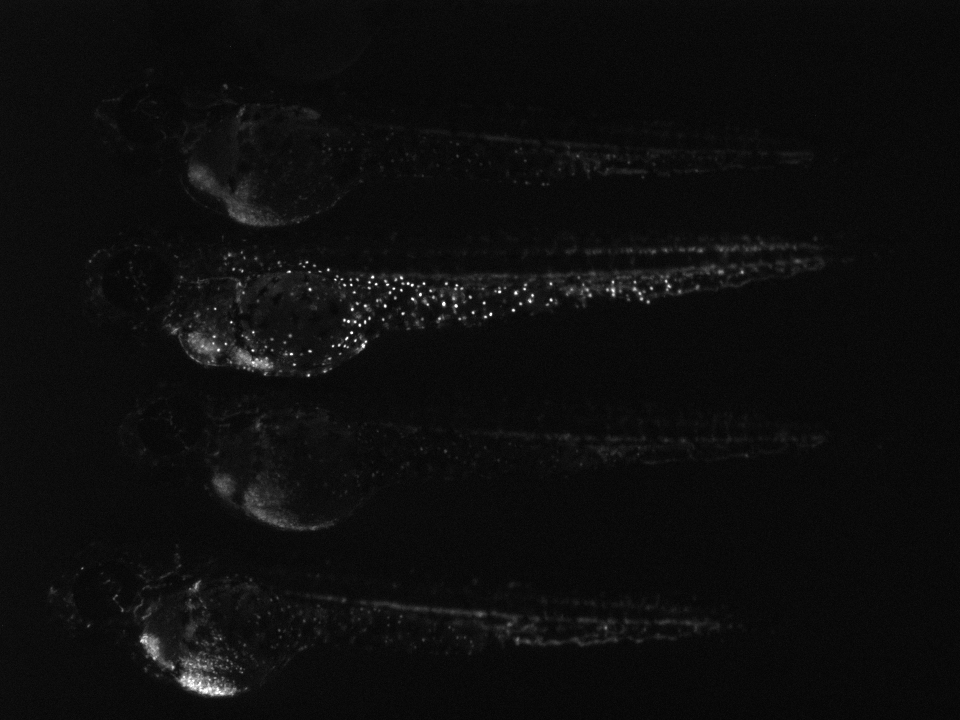

Supplement: Supplementary file 7 — Source data Fig. 3 [file 44321_2025_368_MOESM7_ESM.zip › FIGURE_3/3E/PONATINIB_01uM (2).tif]

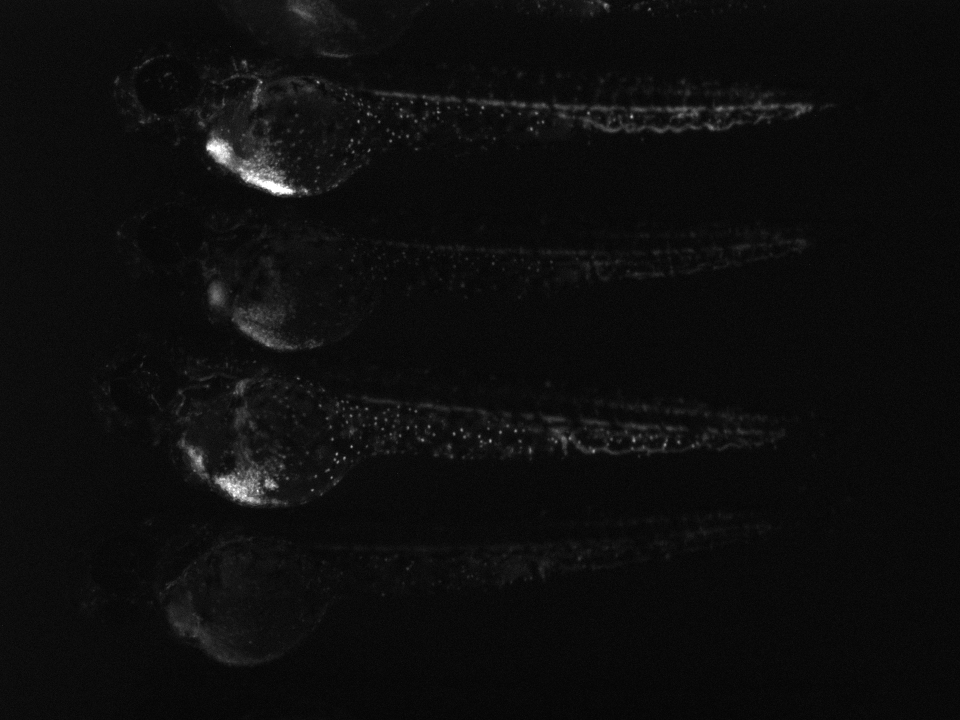

Supplement: Supplementary file 7 — Source data Fig. 3 [file 44321_2025_368_MOESM7_ESM.zip › FIGURE_3/3E/PONATINIB_01uM (3).tif]

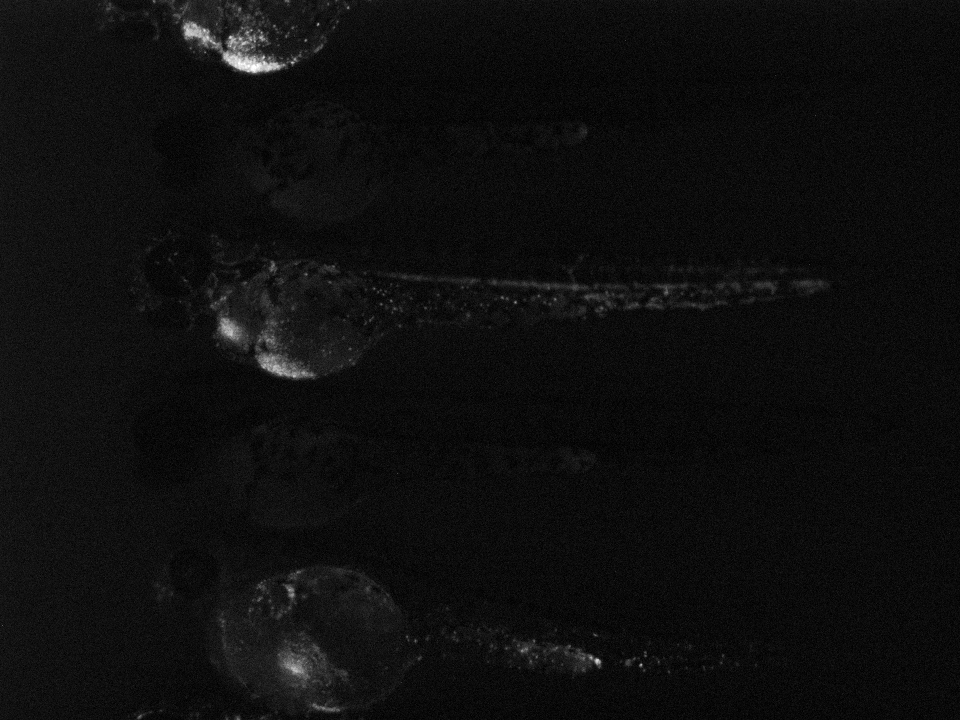

Supplement: Supplementary file 7 — Source data Fig. 3 [file 44321_2025_368_MOESM7_ESM.zip › FIGURE_3/3E/PONATINIB_01uM (4).tif]

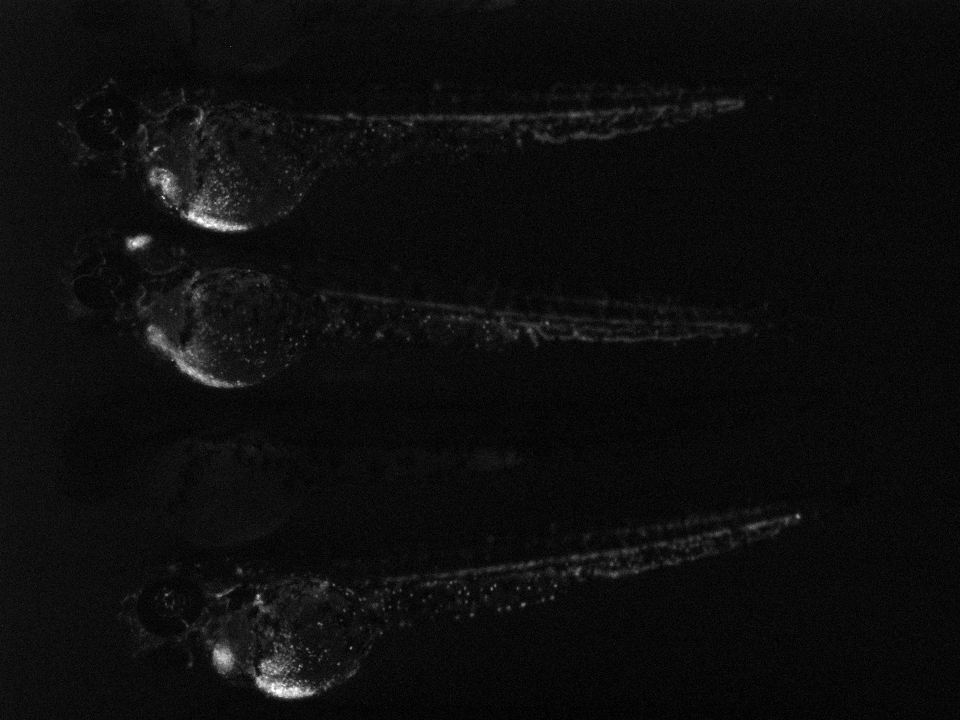

Supplement: Supplementary file 7 — Source data Fig. 3 [file 44321_2025_368_MOESM7_ESM.zip › FIGURE_3/3E/PONATINIB_01uM (5).tif]

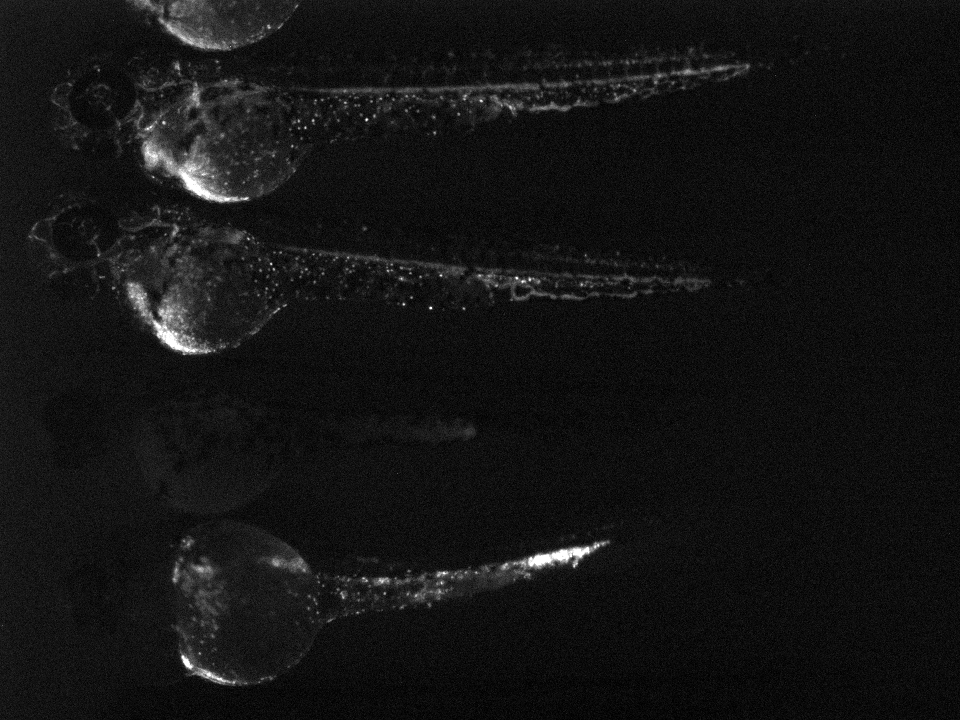

Supplement: Supplementary file 7 — Source data Fig. 3 [file 44321_2025_368_MOESM7_ESM.zip › FIGURE_3/3E/PONATINIB_01uM (6).tif]

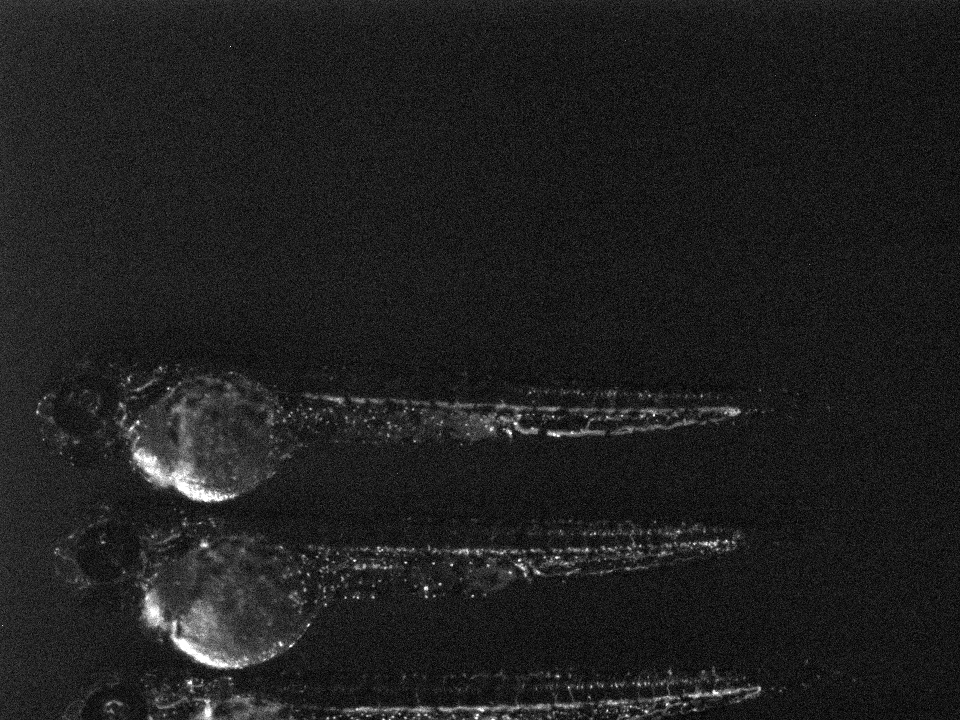

Supplement: Supplementary file 7 — Source data Fig. 3 [file 44321_2025_368_MOESM7_ESM.zip › FIGURE_3/3E/PONATINIB_01uM (7).tif]

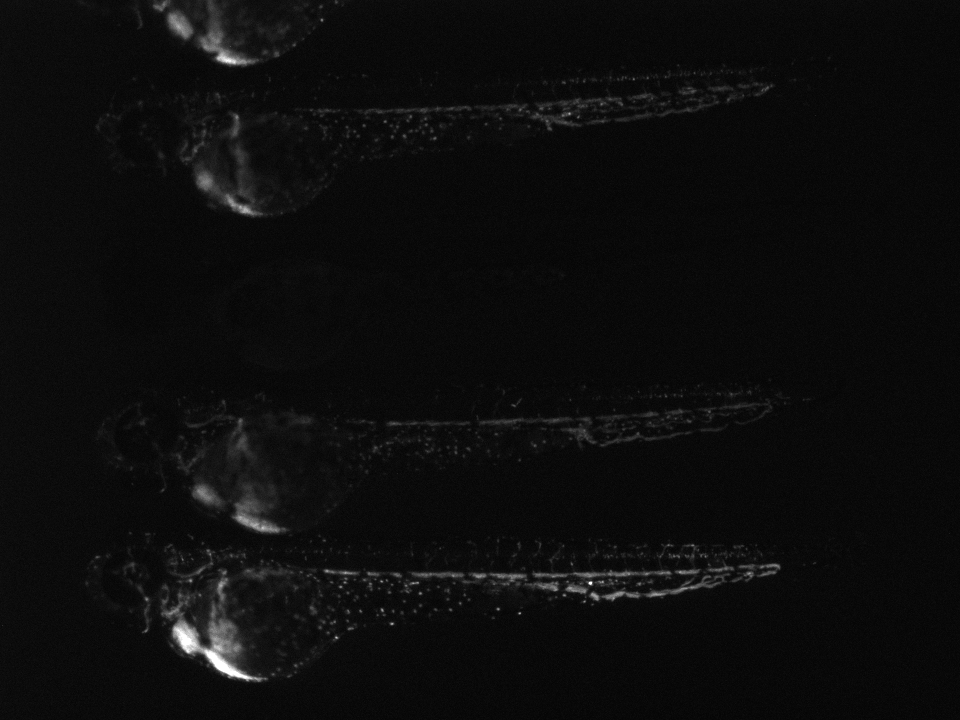

Supplement: Supplementary file 7 — Source data Fig. 3 [file 44321_2025_368_MOESM7_ESM.zip › FIGURE_3/3E/PONATINIB_01uM (8).tif]

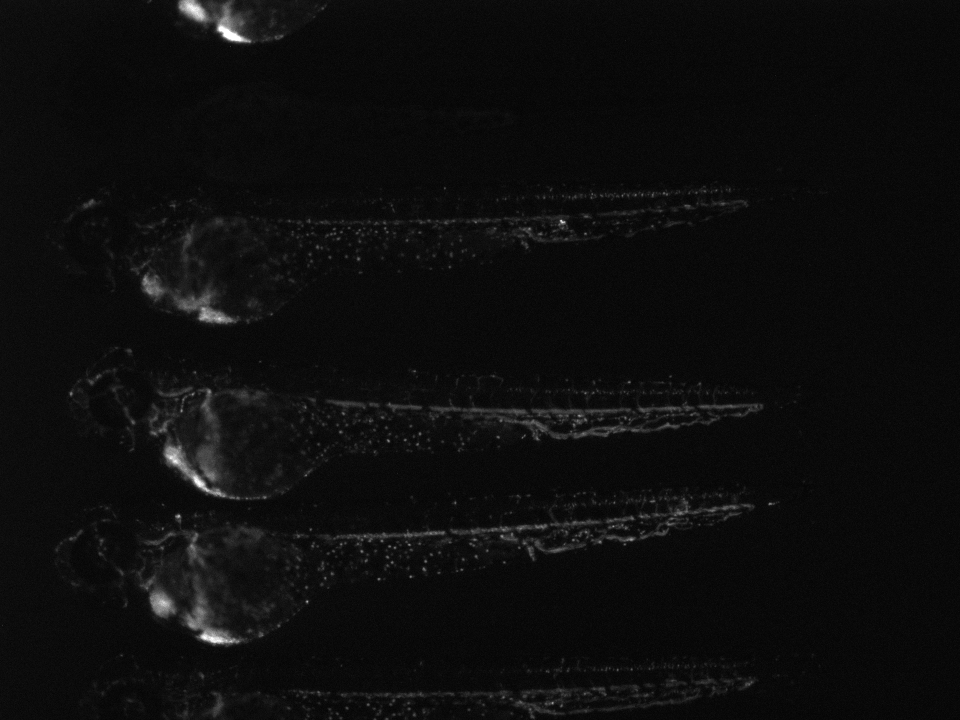

Supplement: Supplementary file 7 — Source data Fig. 3 [file 44321_2025_368_MOESM7_ESM.zip › FIGURE_3/3E/PONATINIB_01uM (9).tif]

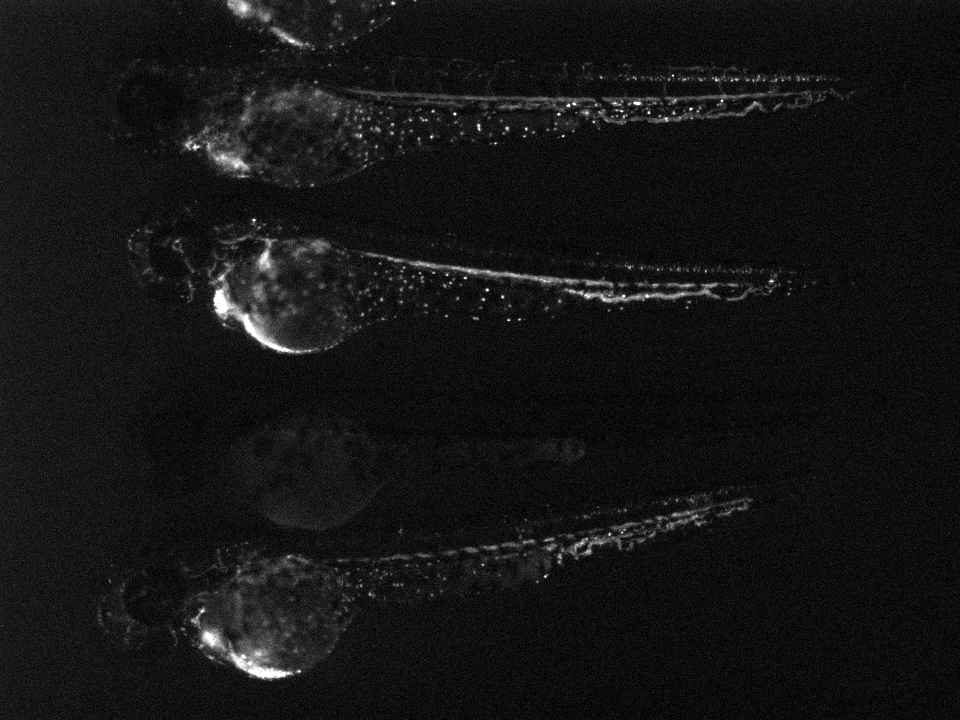

Supplement: Supplementary file 7 — Source data Fig. 3 [file 44321_2025_368_MOESM7_ESM.zip › FIGURE_3/3E/PONATINIB_1uM (1).tif]

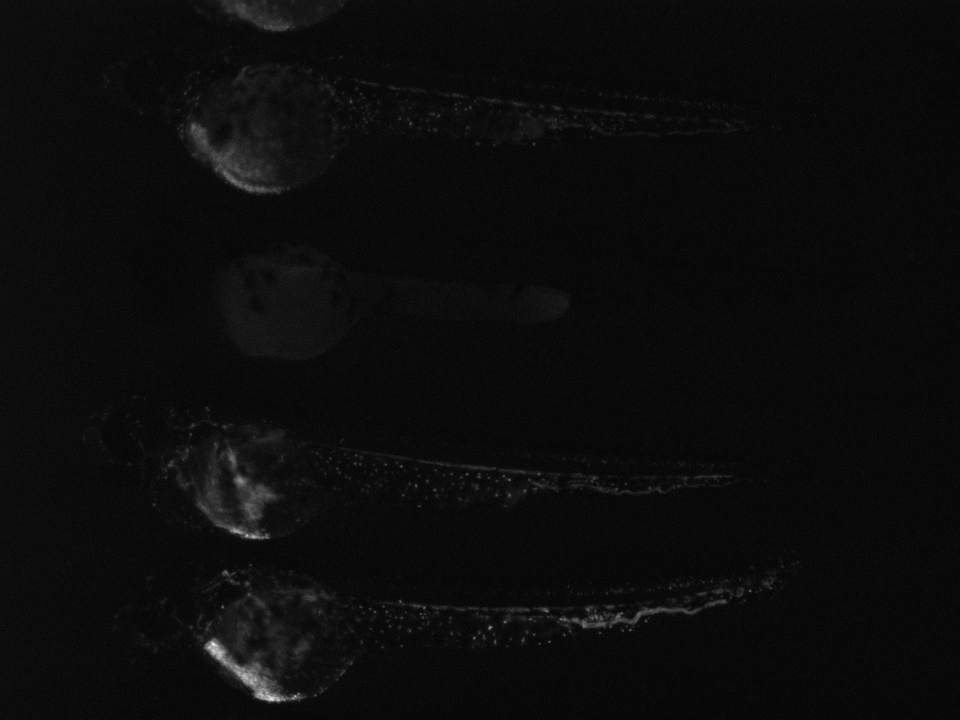

Supplement: Supplementary file 7 — Source data Fig. 3 [file 44321_2025_368_MOESM7_ESM.zip › FIGURE_3/3E/PONATINIB_1uM (10).tif]

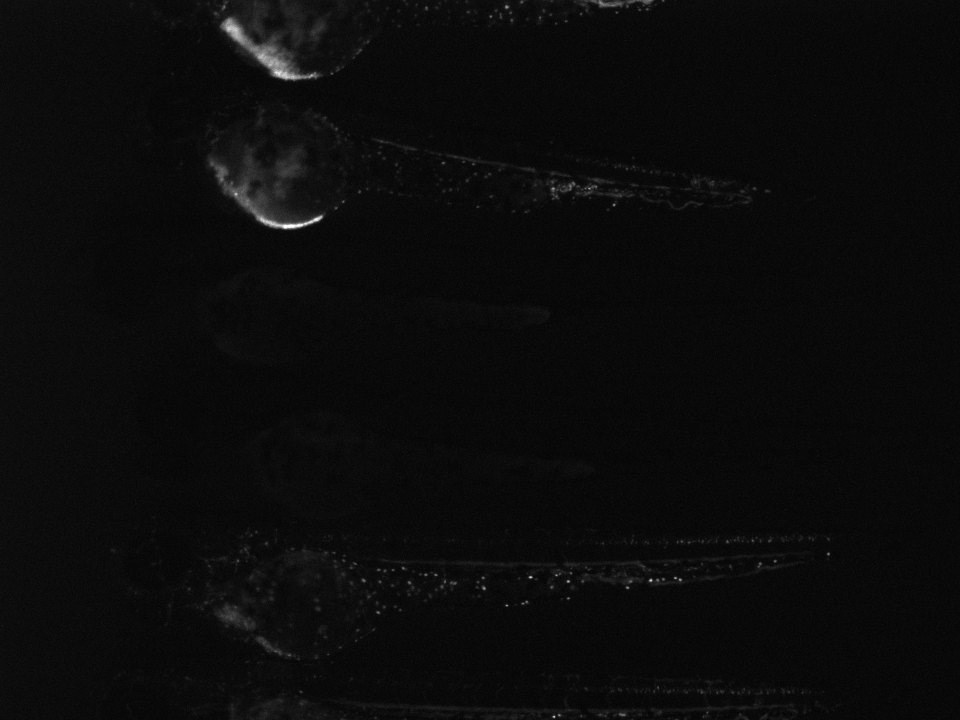

Supplement: Supplementary file 7 — Source data Fig. 3 [file 44321_2025_368_MOESM7_ESM.zip › FIGURE_3/3E/PONATINIB_1uM (11).tif]

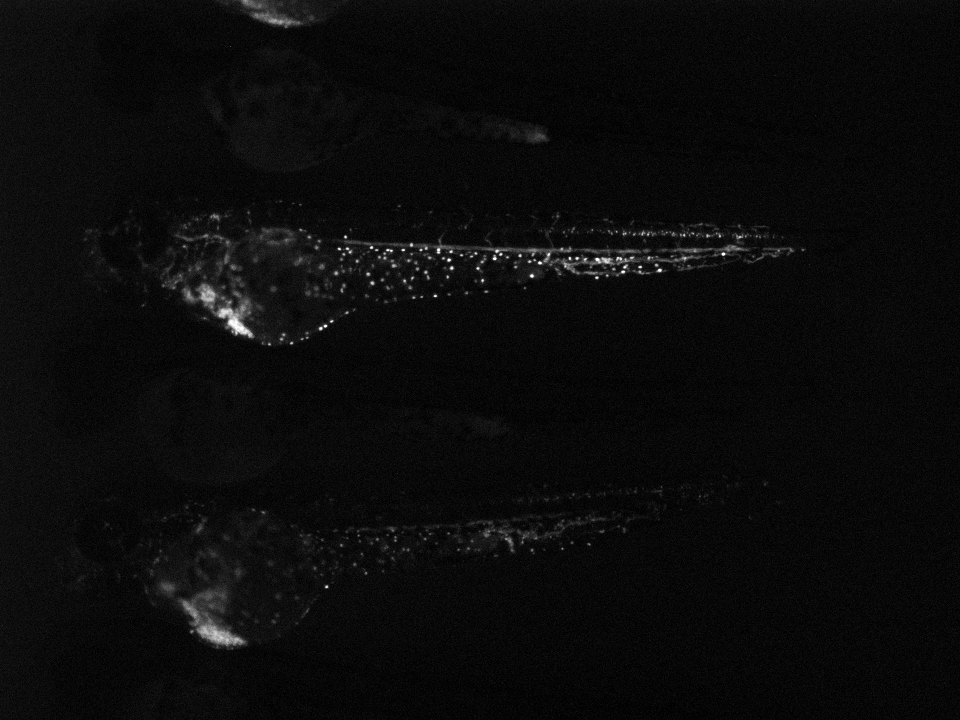

Supplement: Supplementary file 7 — Source data Fig. 3 [file 44321_2025_368_MOESM7_ESM.zip › FIGURE_3/3E/PONATINIB_1uM (2).tif]

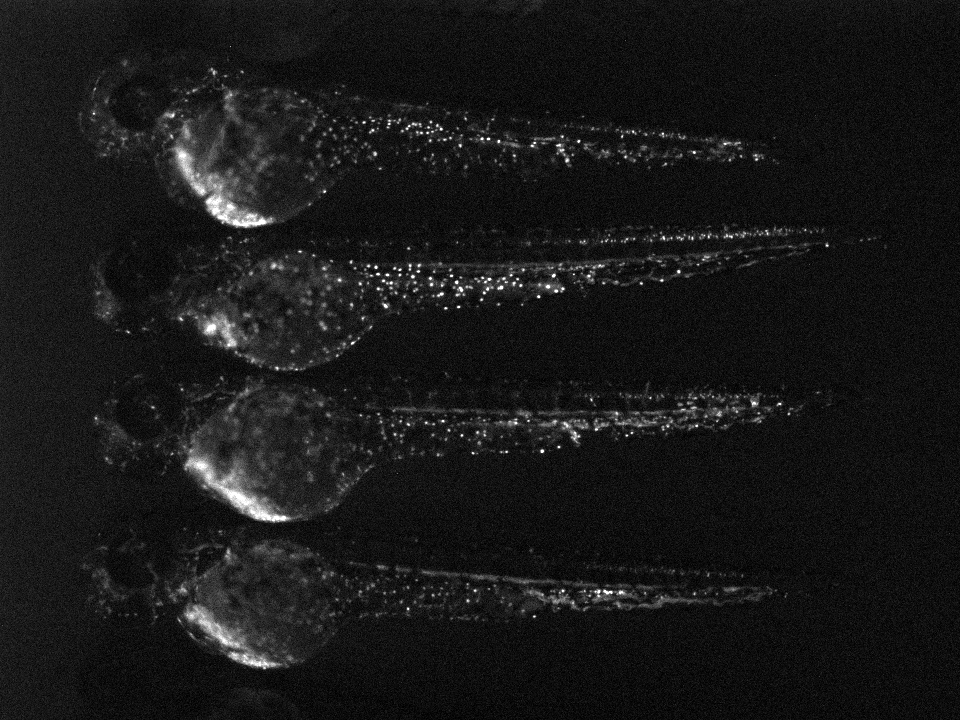

Supplement: Supplementary file 7 — Source data Fig. 3 [file 44321_2025_368_MOESM7_ESM.zip › FIGURE_3/3E/PONATINIB_1uM (3).tif]

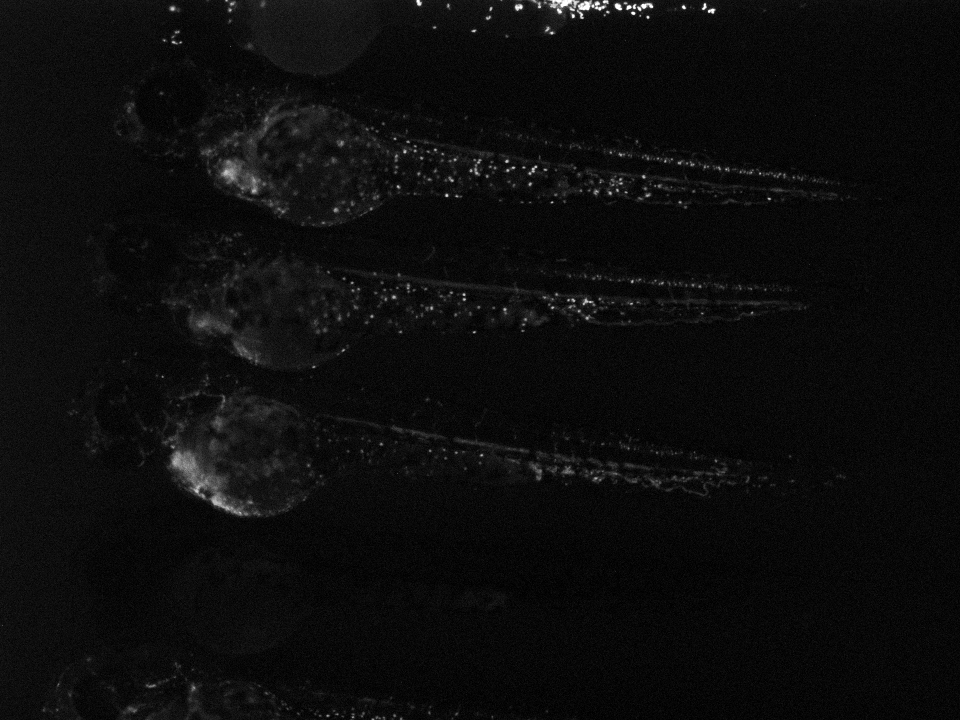

Supplement: Supplementary file 7 — Source data Fig. 3 [file 44321_2025_368_MOESM7_ESM.zip › FIGURE_3/3E/PONATINIB_1uM (4).tif]

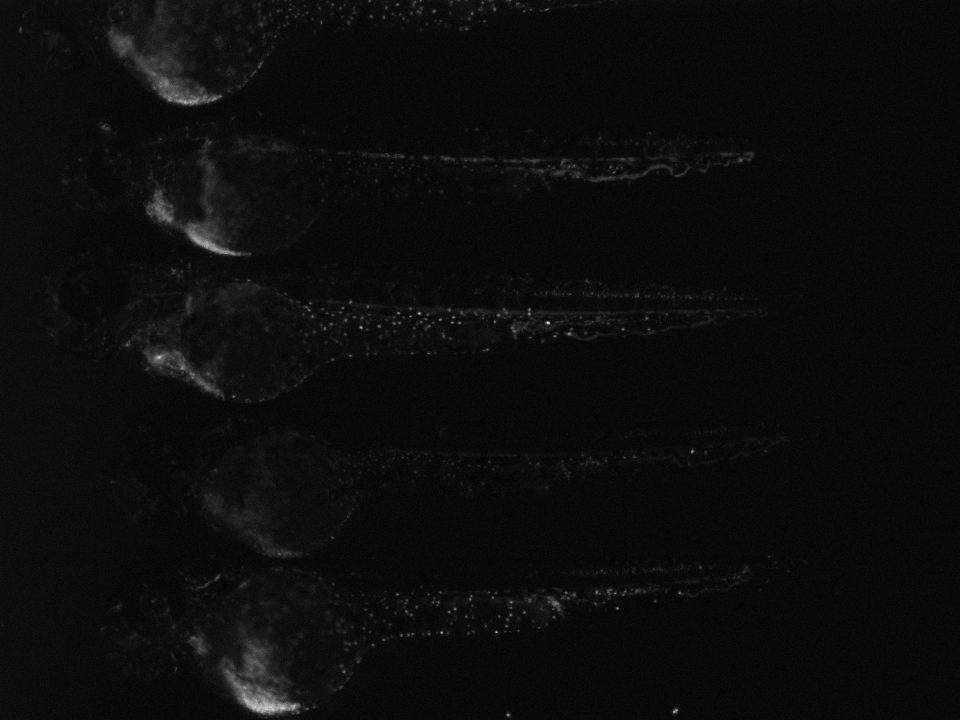

Supplement: Supplementary file 7 — Source data Fig. 3 [file 44321_2025_368_MOESM7_ESM.zip › FIGURE_3/3E/PONATINIB_1uM (5).tif]

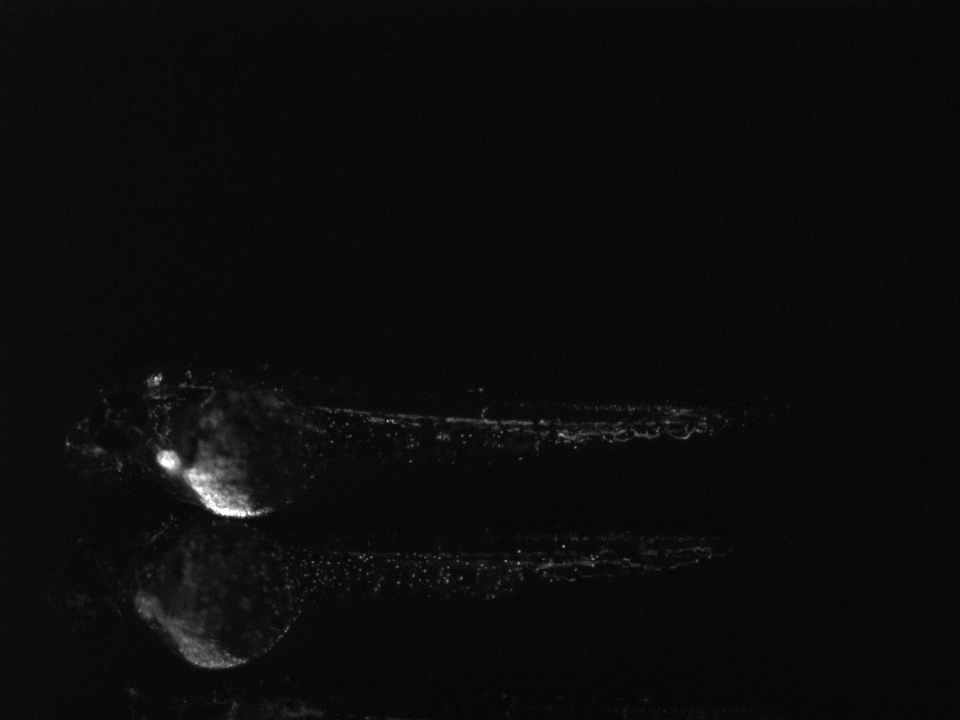

Supplement: Supplementary file 7 — Source data Fig. 3 [file 44321_2025_368_MOESM7_ESM.zip › FIGURE_3/3E/PONATINIB_1uM (6).tif]

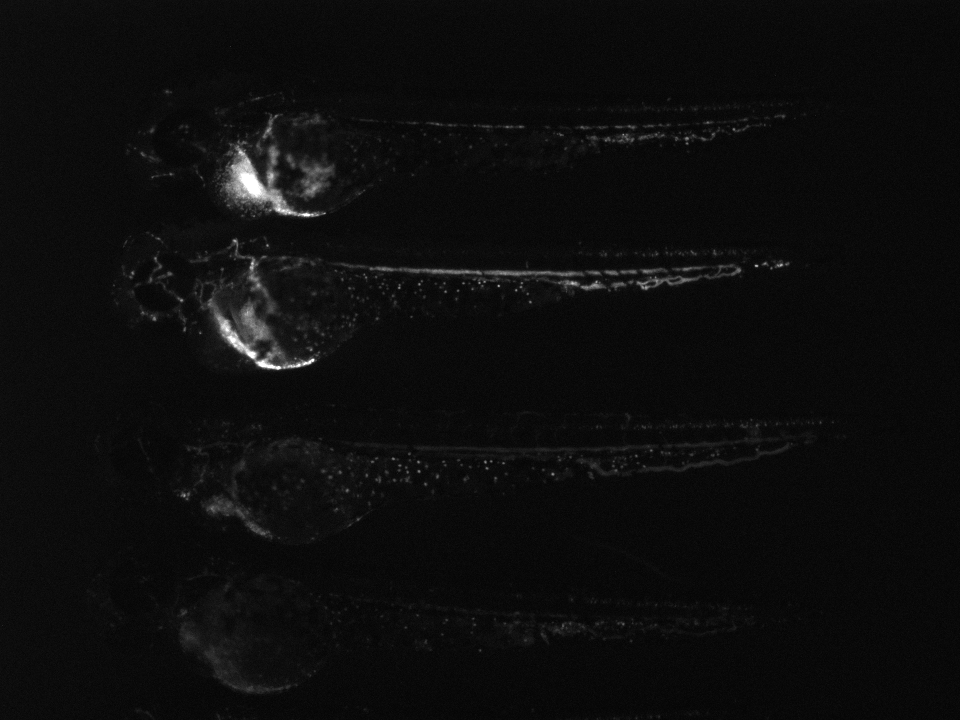

Supplement: Supplementary file 7 — Source data Fig. 3 [file 44321_2025_368_MOESM7_ESM.zip › FIGURE_3/3E/PONATINIB_1uM (7).tif]

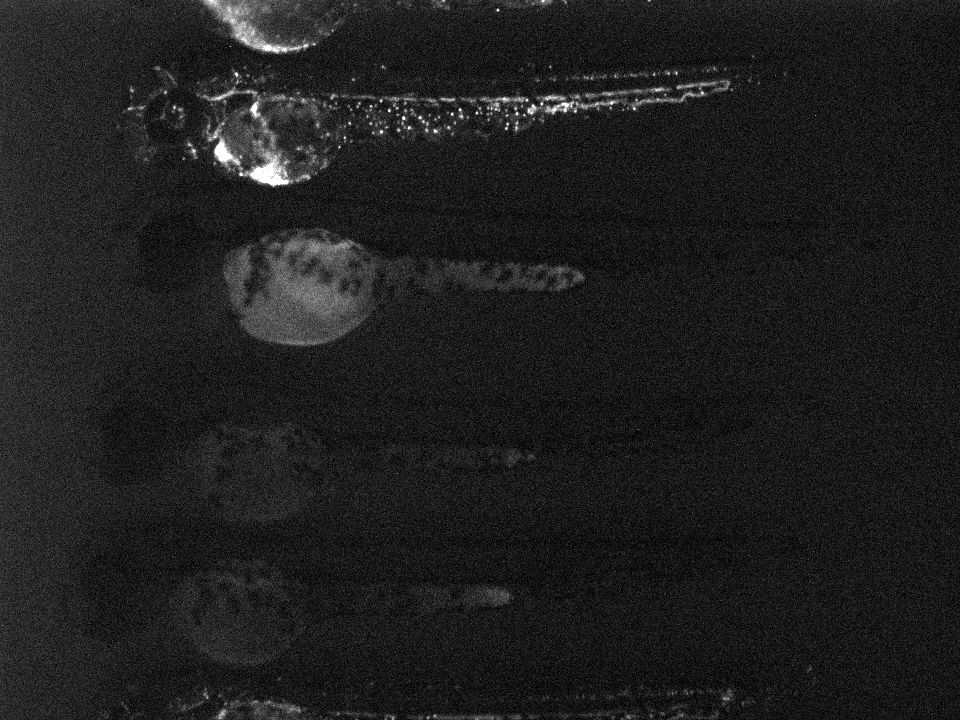

Supplement: Supplementary file 7 — Source data Fig. 3 [file 44321_2025_368_MOESM7_ESM.zip › FIGURE_3/3E/PONATINIB_1uM (8).tif]

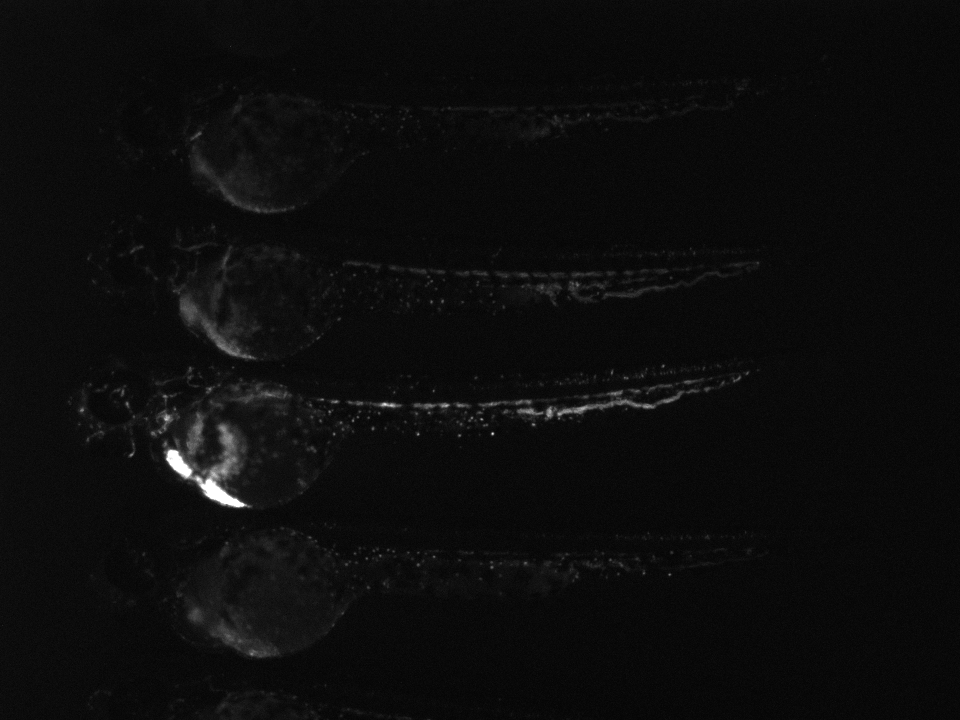

Supplement: Supplementary file 7 — Source data Fig. 3 [file 44321_2025_368_MOESM7_ESM.zip › FIGURE_3/3E/PONATINIB_1uM (9).tif]

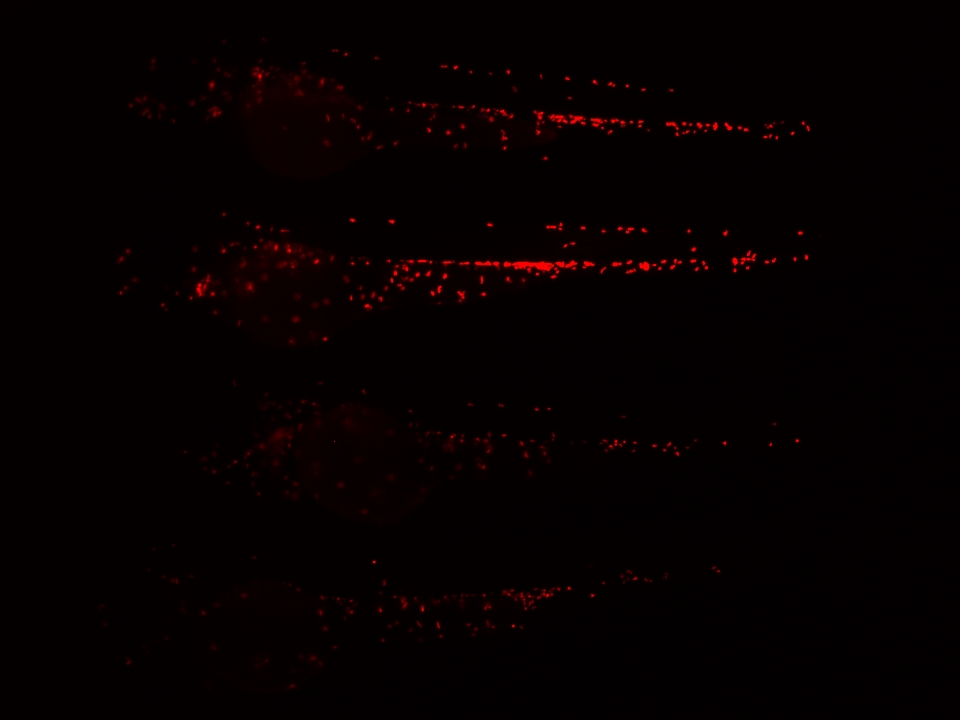

Supplement: Supplementary file 8 — Source data Fig. 4 [file 44321_2025_368_MOESM8_ESM.zip › FIGURE_4/4B/BOSUTINIB_01uM (1).jpg]

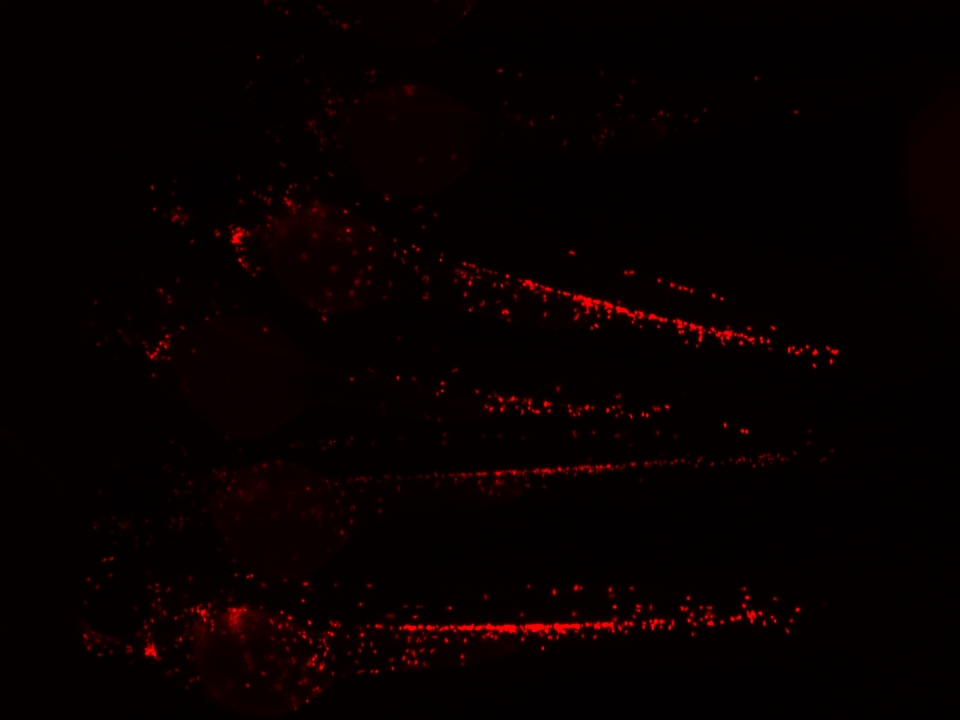

Supplement: Supplementary file 8 — Source data Fig. 4 [file 44321_2025_368_MOESM8_ESM.zip › FIGURE_4/4B/BOSUTINIB_01uM (10).jpg]

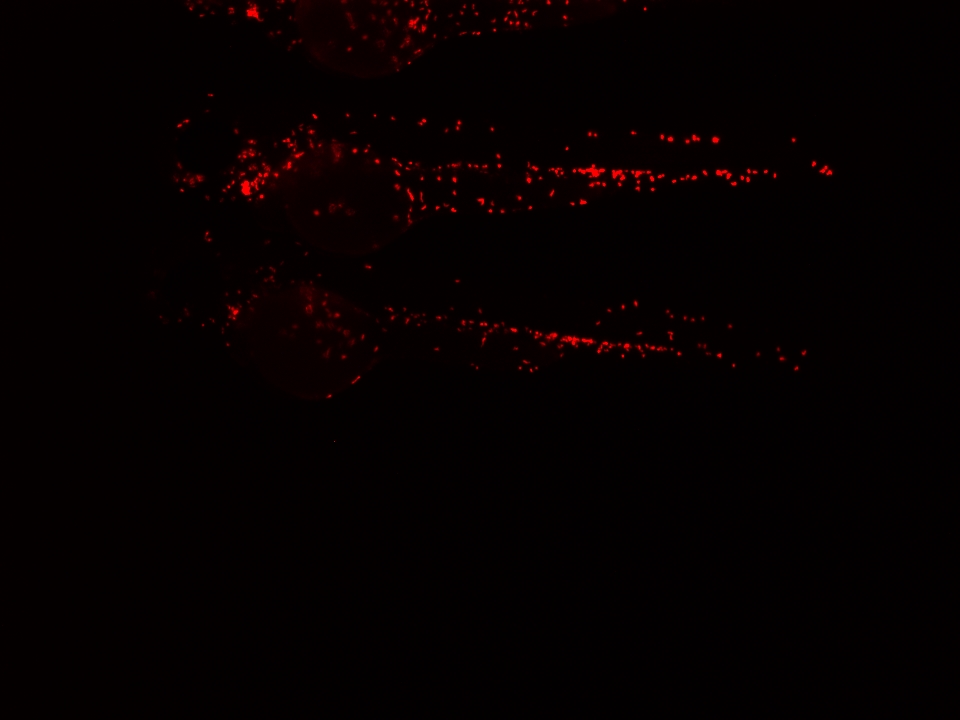

Supplement: Supplementary file 8 — Source data Fig. 4 [file 44321_2025_368_MOESM8_ESM.zip › FIGURE_4/4B/BOSUTINIB_01uM (11).jpg]

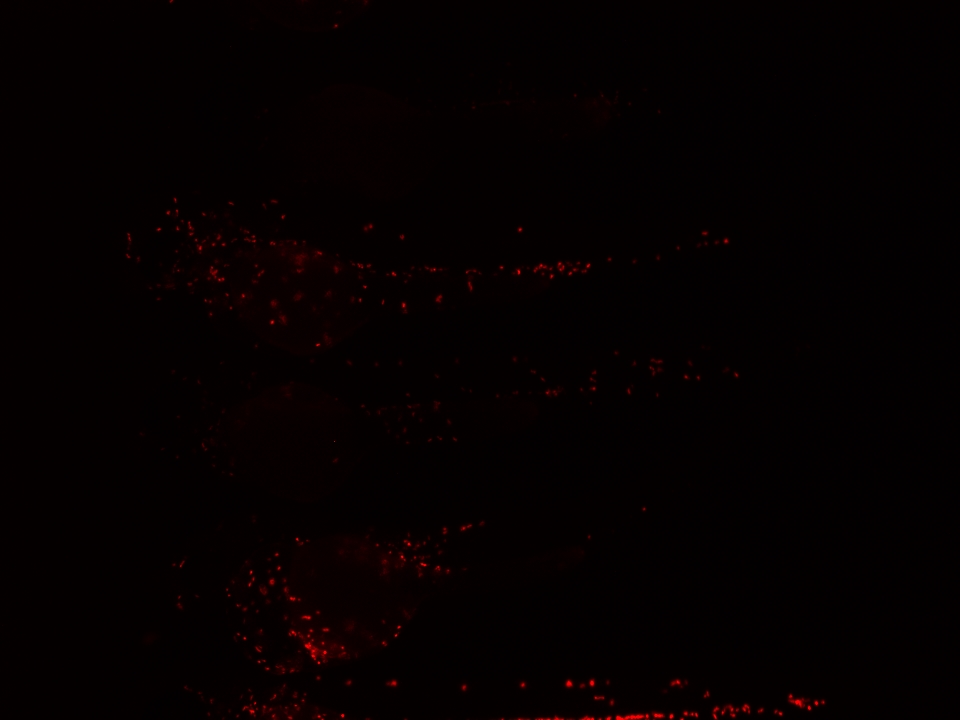

Supplement: Supplementary file 8 — Source data Fig. 4 [file 44321_2025_368_MOESM8_ESM.zip › FIGURE_4/4B/BOSUTINIB_01uM (2).jpg]

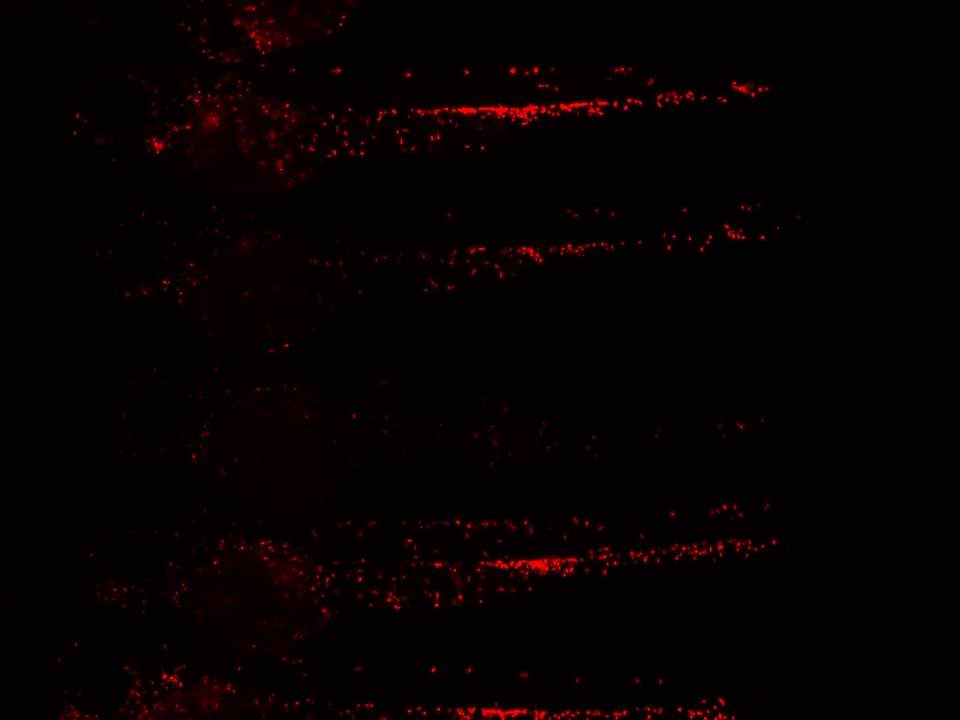

Supplement: Supplementary file 8 — Source data Fig. 4 [file 44321_2025_368_MOESM8_ESM.zip › FIGURE_4/4B/BOSUTINIB_01uM (3).jpg]

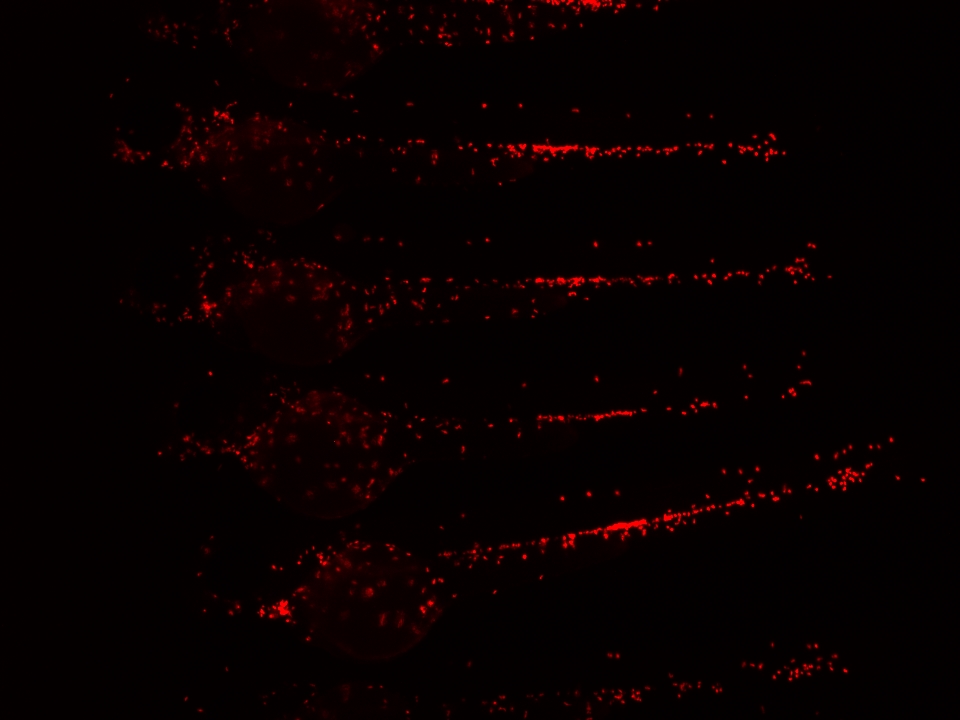

Supplement: Supplementary file 8 — Source data Fig. 4 [file 44321_2025_368_MOESM8_ESM.zip › FIGURE_4/4B/BOSUTINIB_01uM (4).jpg]

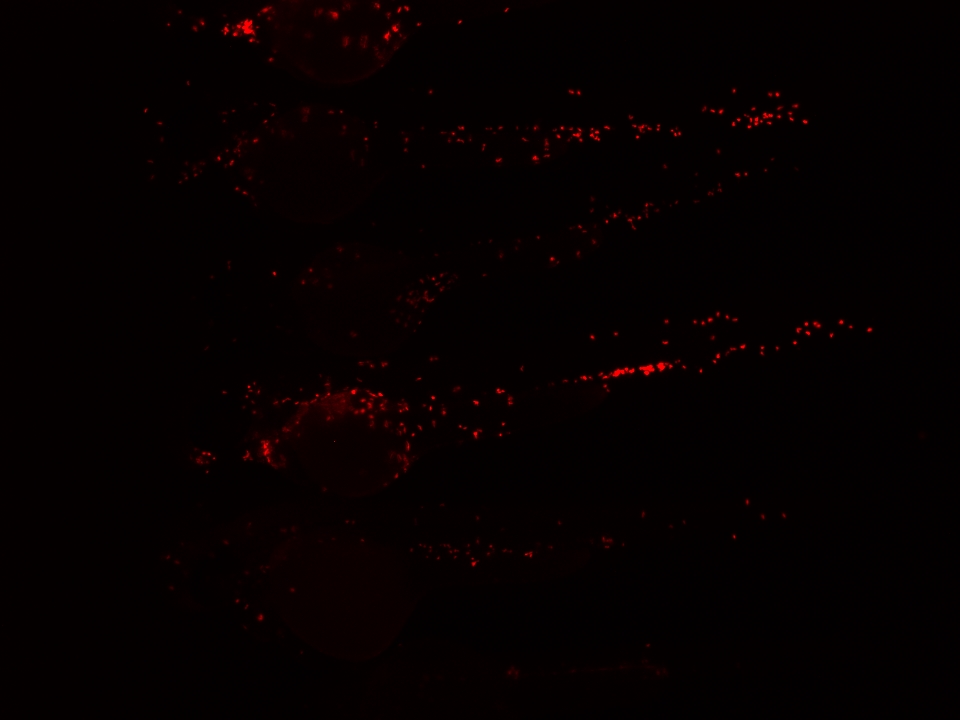

Supplement: Supplementary file 8 — Source data Fig. 4 [file 44321_2025_368_MOESM8_ESM.zip › FIGURE_4/4B/BOSUTINIB_01uM (5).jpg]

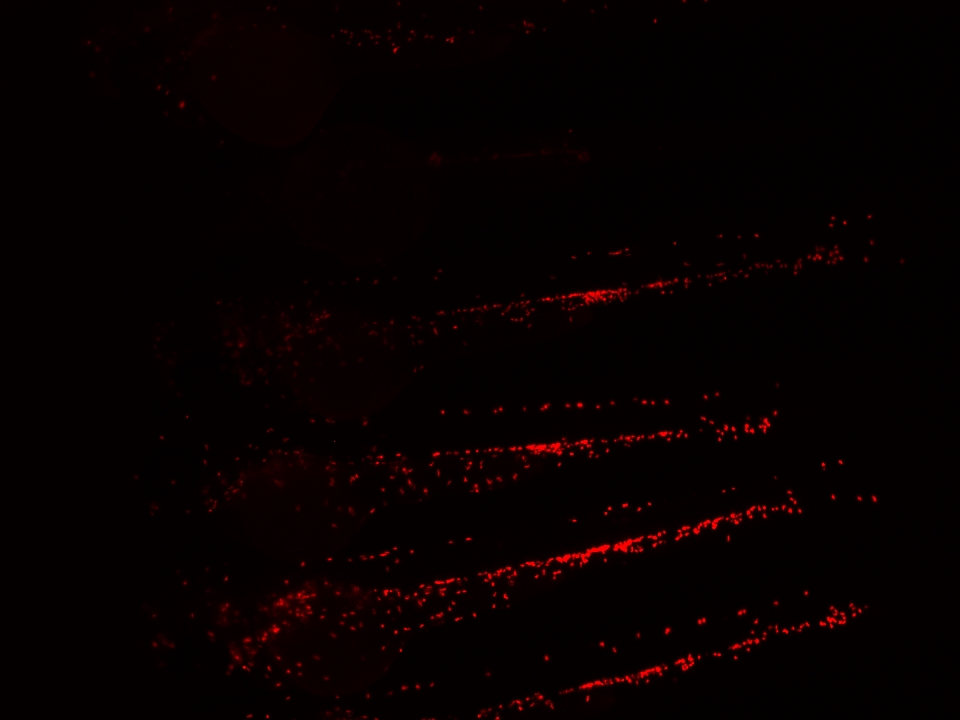

Supplement: Supplementary file 8 — Source data Fig. 4 [file 44321_2025_368_MOESM8_ESM.zip › FIGURE_4/4B/BOSUTINIB_01uM (6).jpg]

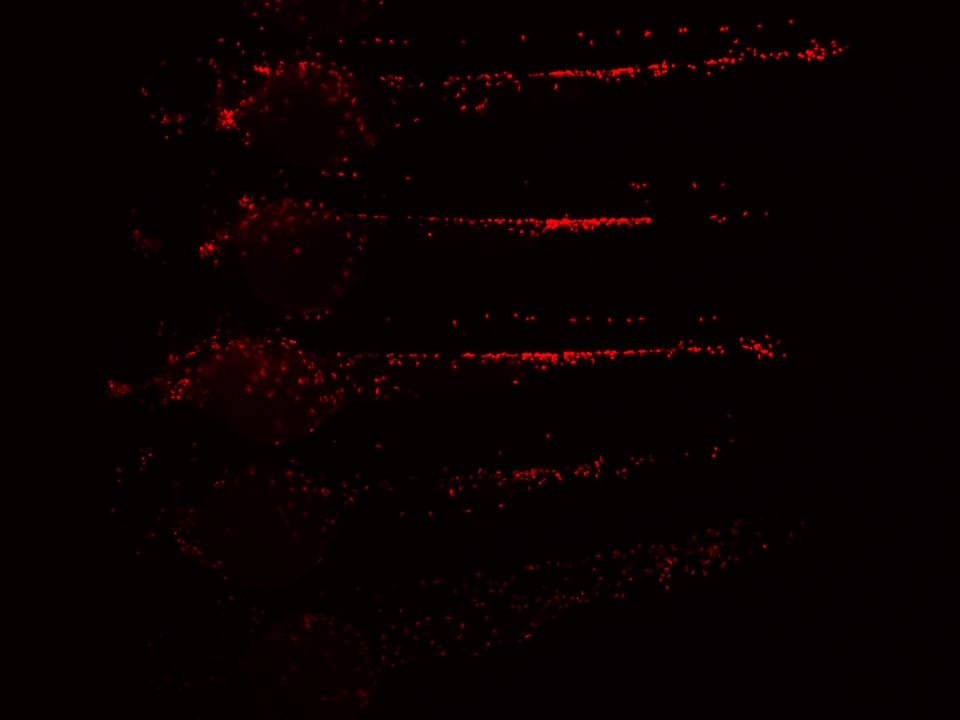

Supplement: Supplementary file 8 — Source data Fig. 4 [file 44321_2025_368_MOESM8_ESM.zip › FIGURE_4/4B/BOSUTINIB_01uM (7).jpg]

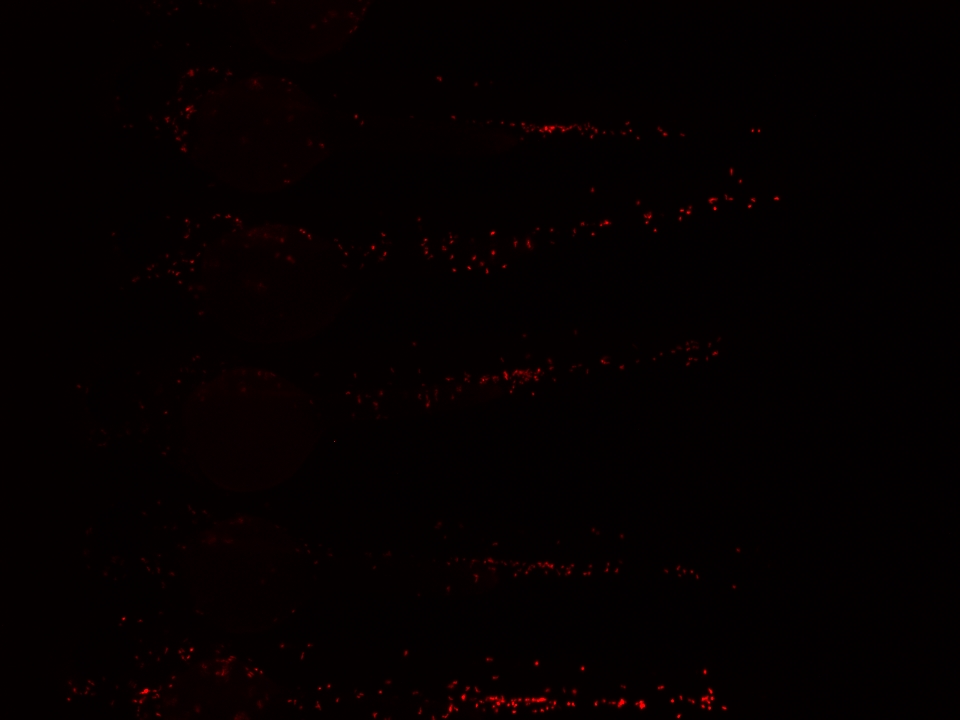

Supplement: Supplementary file 8 — Source data Fig. 4 [file 44321_2025_368_MOESM8_ESM.zip › FIGURE_4/4B/BOSUTINIB_01uM (8).jpg]

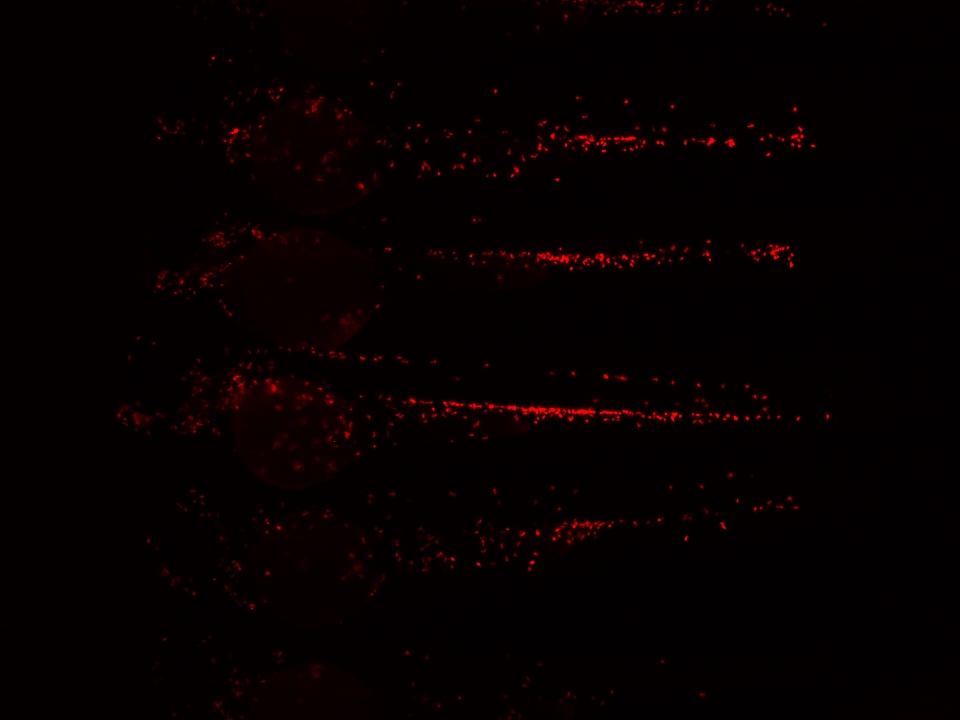

Supplement: Supplementary file 8 — Source data Fig. 4 [file 44321_2025_368_MOESM8_ESM.zip › FIGURE_4/4B/BOSUTINIB_01uM (9).jpg]

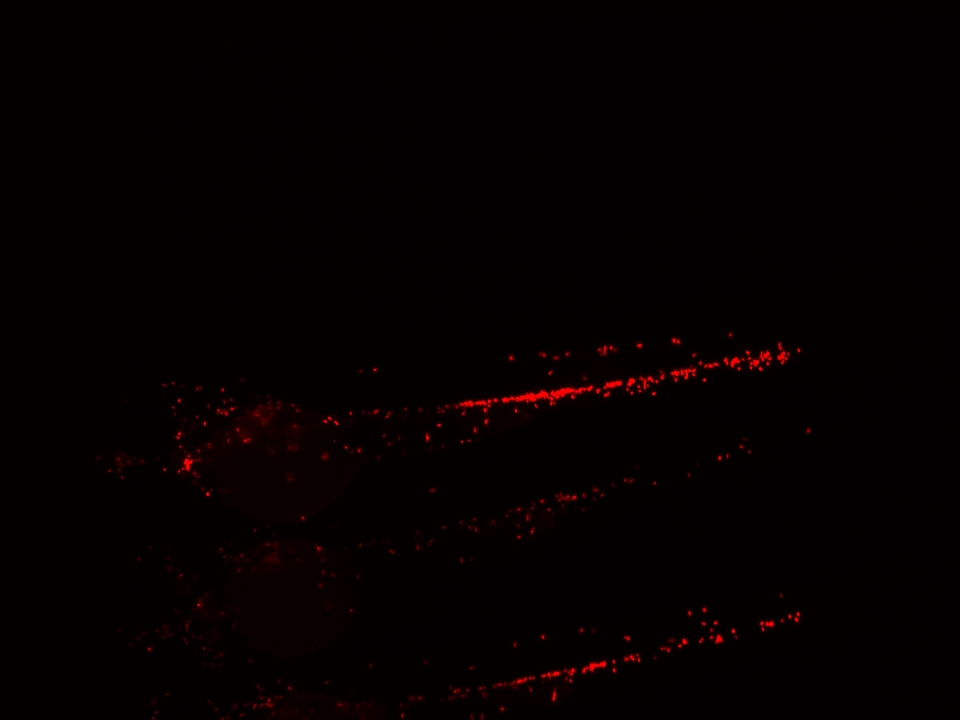

Supplement: Supplementary file 8 — Source data Fig. 4 [file 44321_2025_368_MOESM8_ESM.zip › FIGURE_4/4B/BOSUTINIB_1uM (1).jpg]

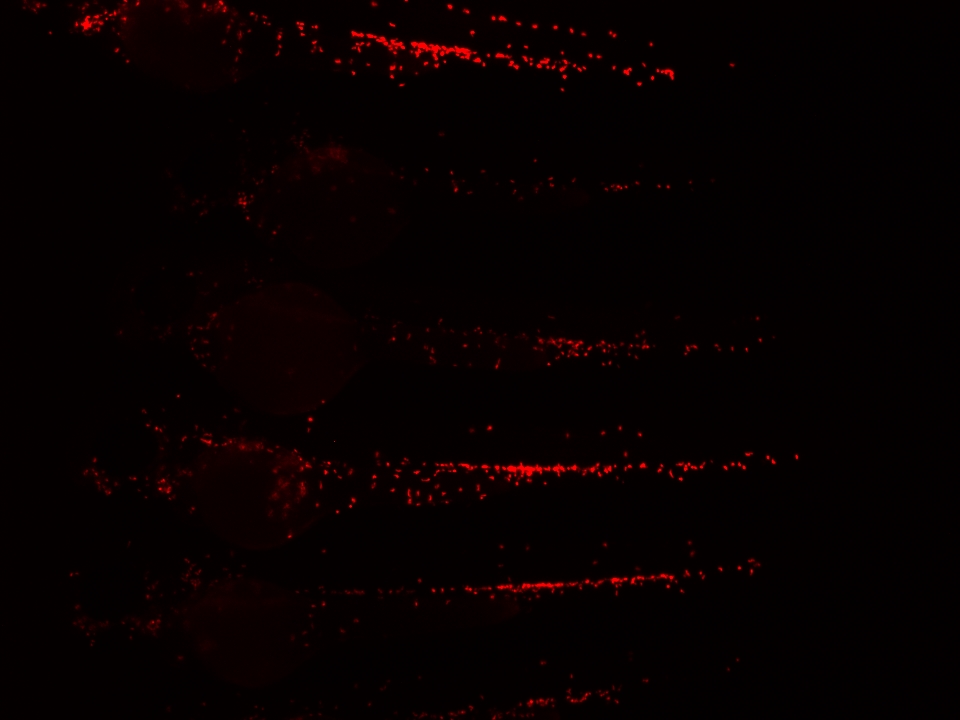

Supplement: Supplementary file 8 — Source data Fig. 4 [file 44321_2025_368_MOESM8_ESM.zip › FIGURE_4/4B/BOSUTINIB_1uM (10).jpg]

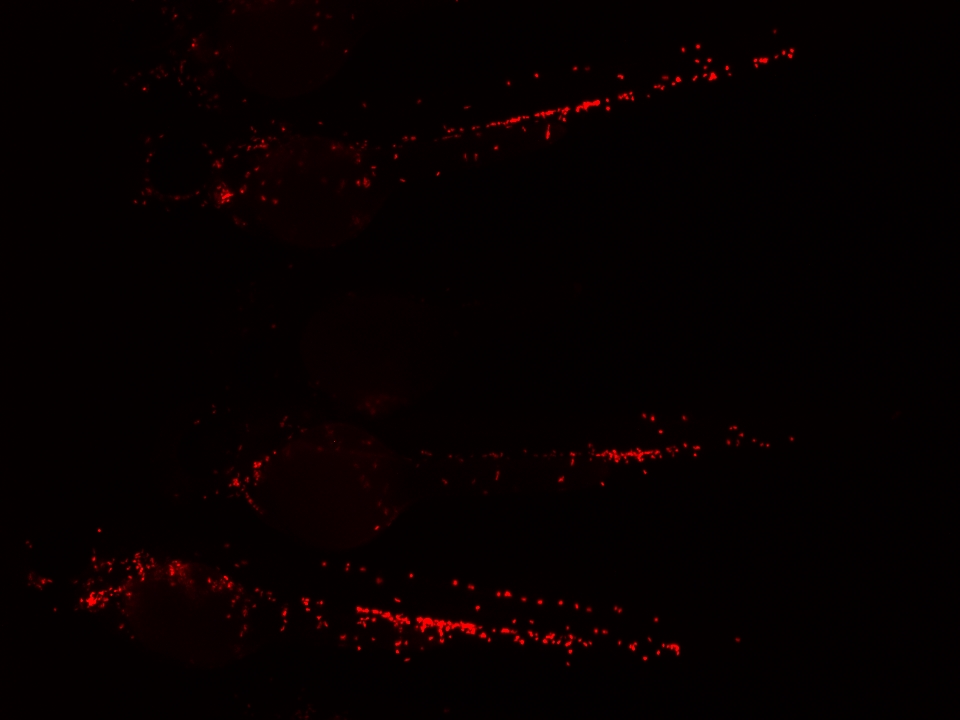

Supplement: Supplementary file 8 — Source data Fig. 4 [file 44321_2025_368_MOESM8_ESM.zip › FIGURE_4/4B/BOSUTINIB_1uM (11).jpg]

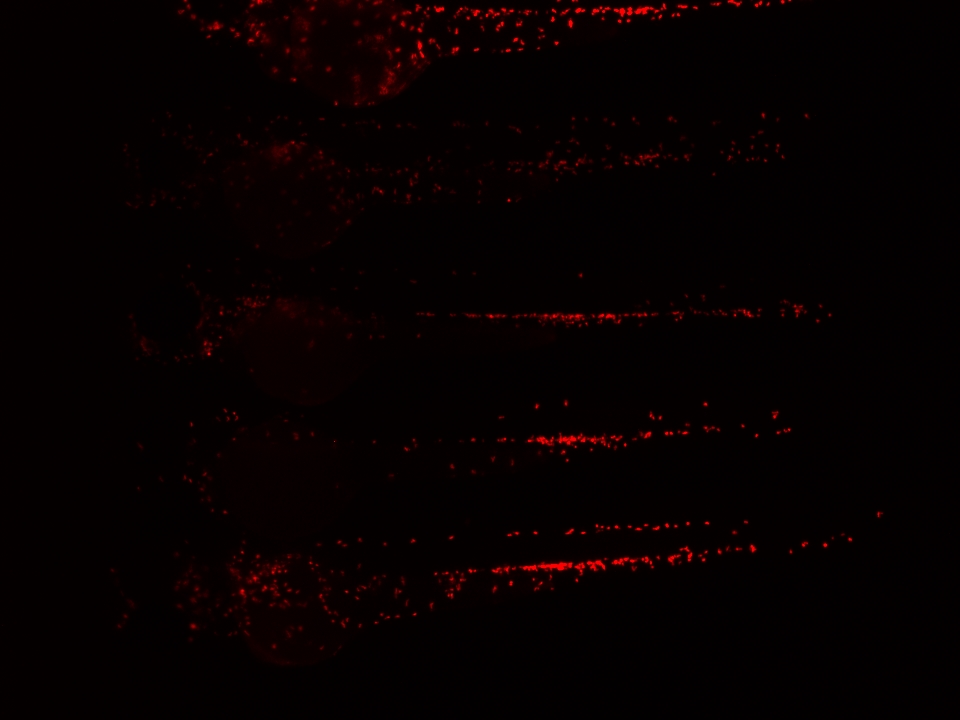

Supplement: Supplementary file 8 — Source data Fig. 4 [file 44321_2025_368_MOESM8_ESM.zip › FIGURE_4/4B/BOSUTINIB_1uM (2).jpg]

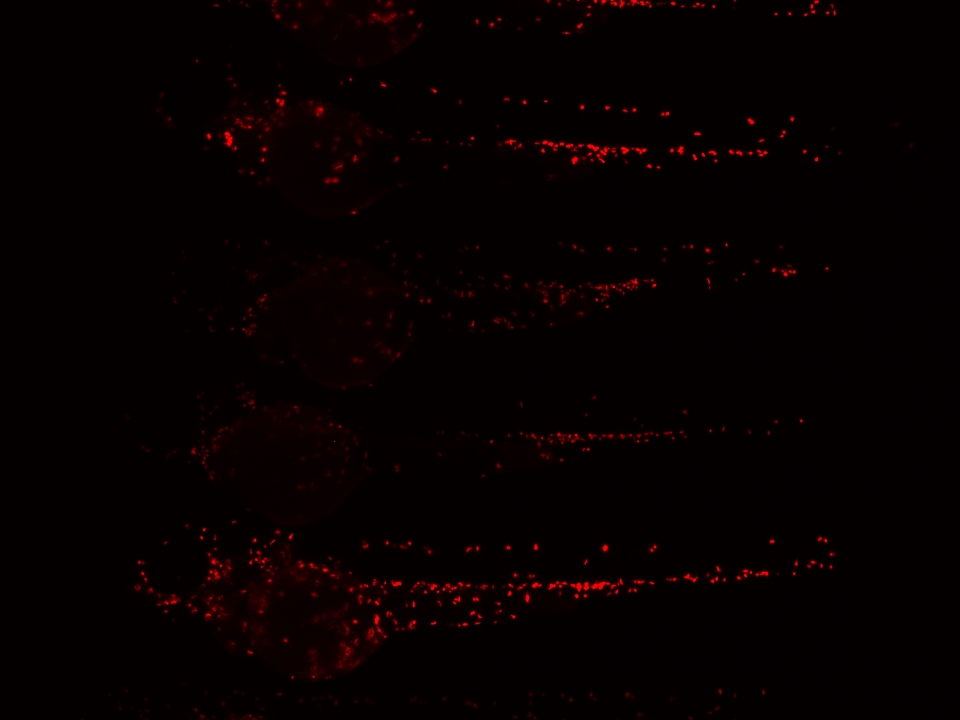

Supplement: Supplementary file 8 — Source data Fig. 4 [file 44321_2025_368_MOESM8_ESM.zip › FIGURE_4/4B/BOSUTINIB_1uM (3).jpg]

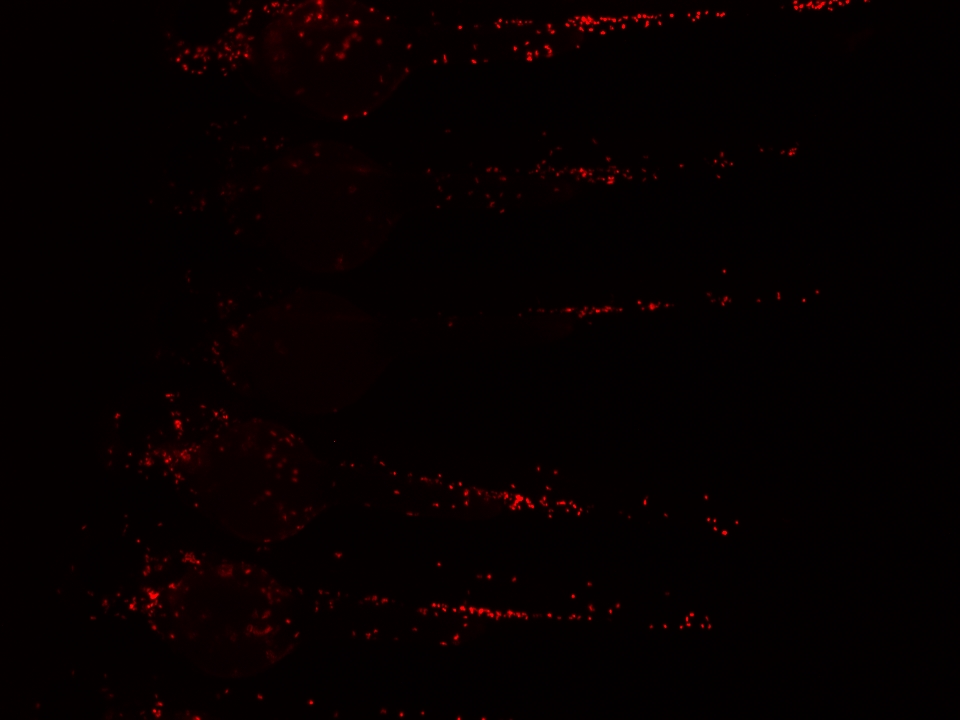

Supplement: Supplementary file 8 — Source data Fig. 4 [file 44321_2025_368_MOESM8_ESM.zip › FIGURE_4/4B/BOSUTINIB_1uM (4).jpg]

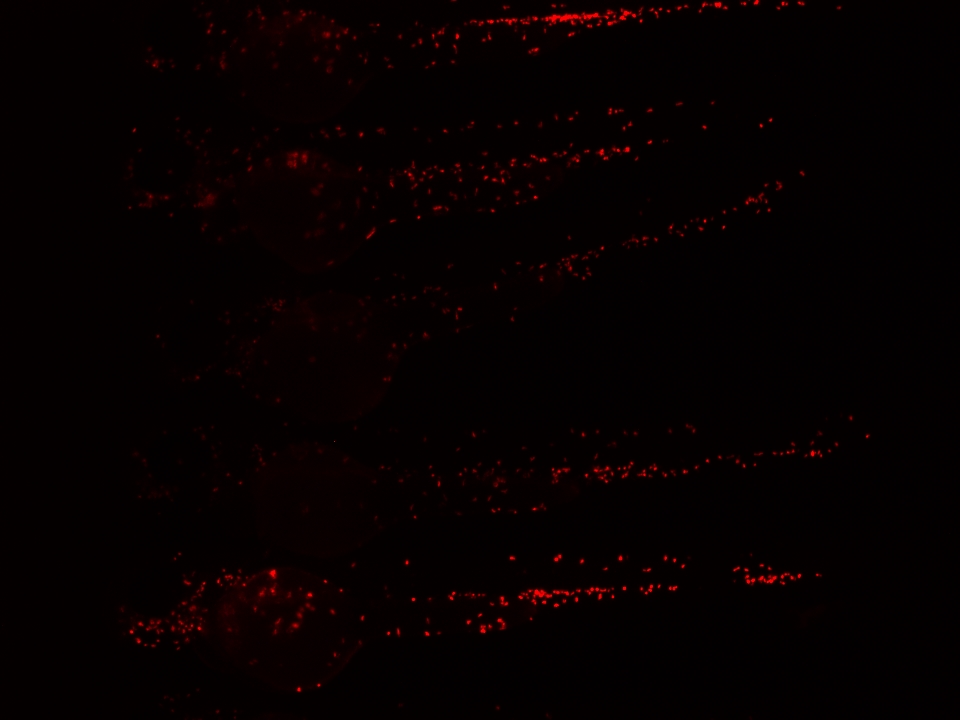

Supplement: Supplementary file 8 — Source data Fig. 4 [file 44321_2025_368_MOESM8_ESM.zip › FIGURE_4/4B/BOSUTINIB_1uM (5).jpg]

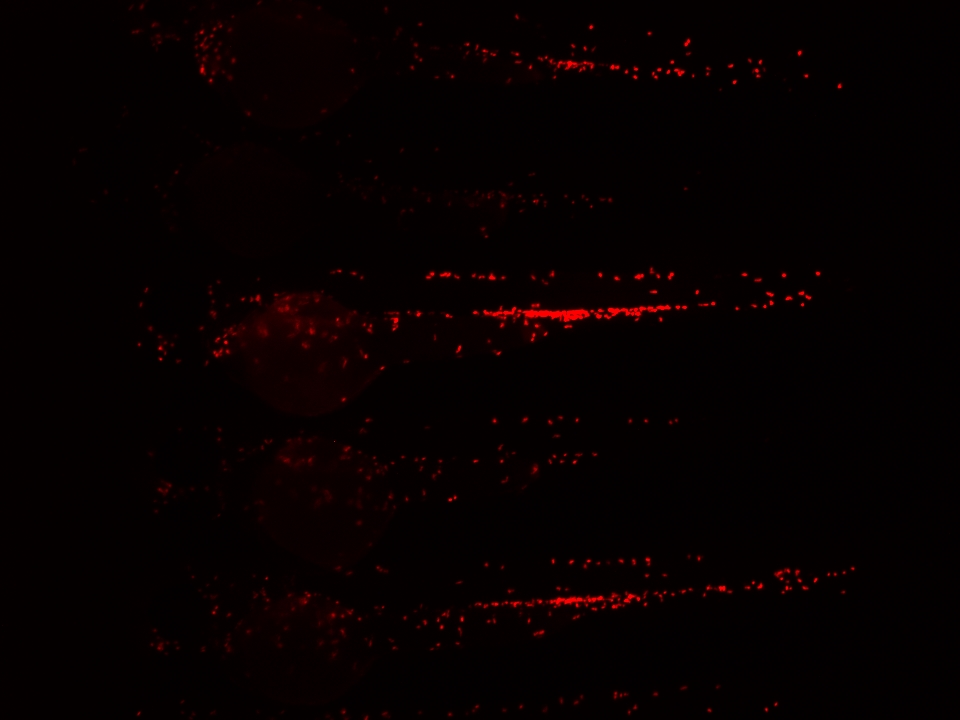

Supplement: Supplementary file 8 — Source data Fig. 4 [file 44321_2025_368_MOESM8_ESM.zip › FIGURE_4/4B/BOSUTINIB_1uM (6).jpg]

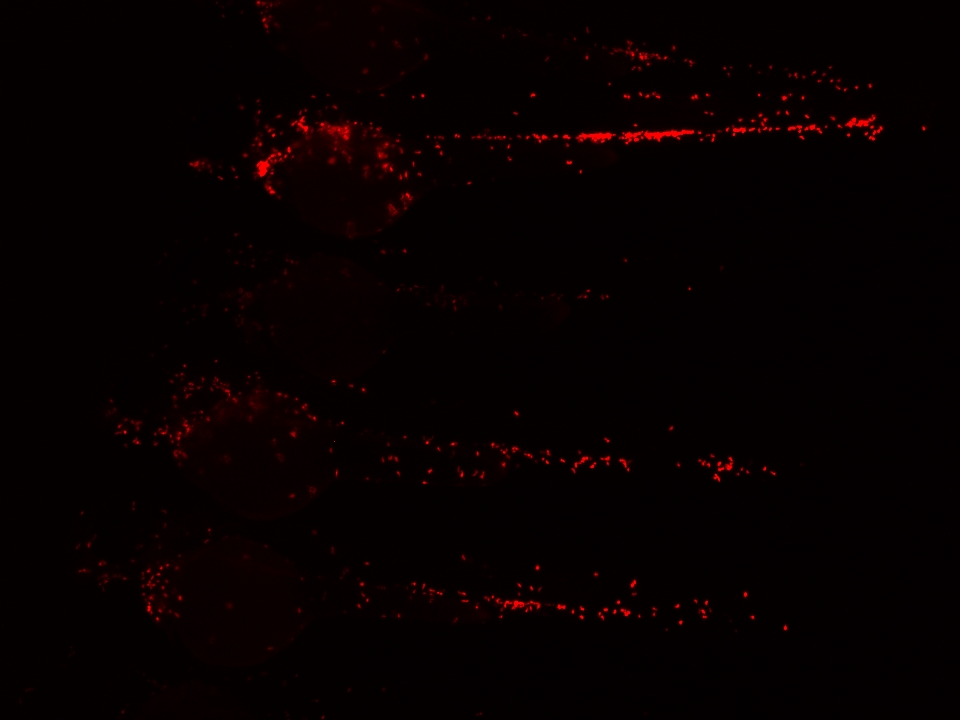

Supplement: Supplementary file 8 — Source data Fig. 4 [file 44321_2025_368_MOESM8_ESM.zip › FIGURE_4/4B/BOSUTINIB_1uM (7).jpg]

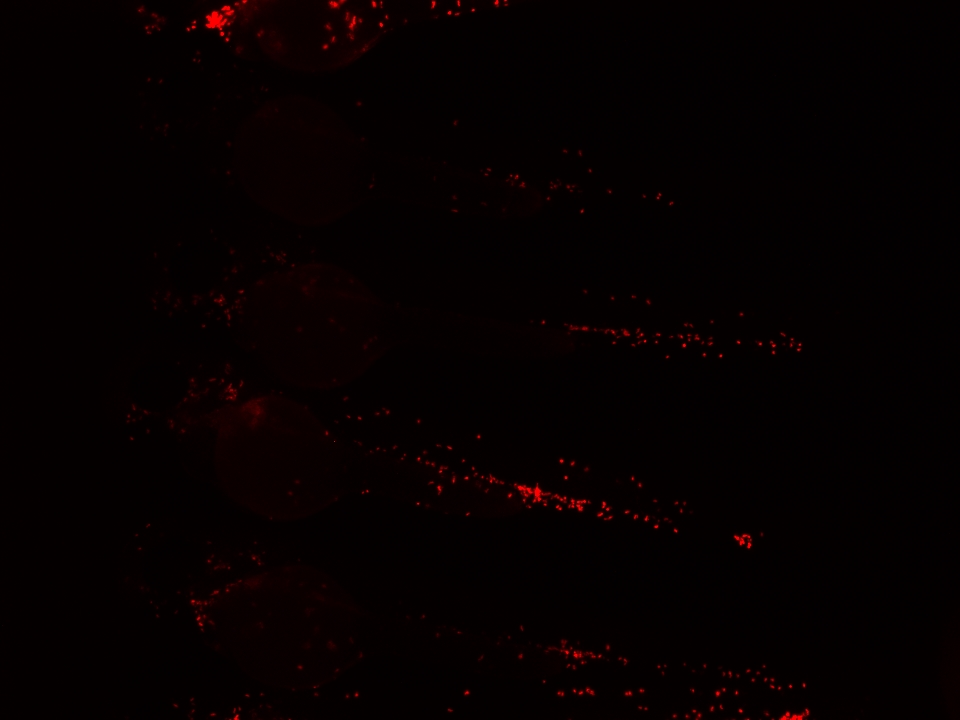

Supplement: Supplementary file 8 — Source data Fig. 4 [file 44321_2025_368_MOESM8_ESM.zip › FIGURE_4/4B/BOSUTINIB_1uM (8).jpg]

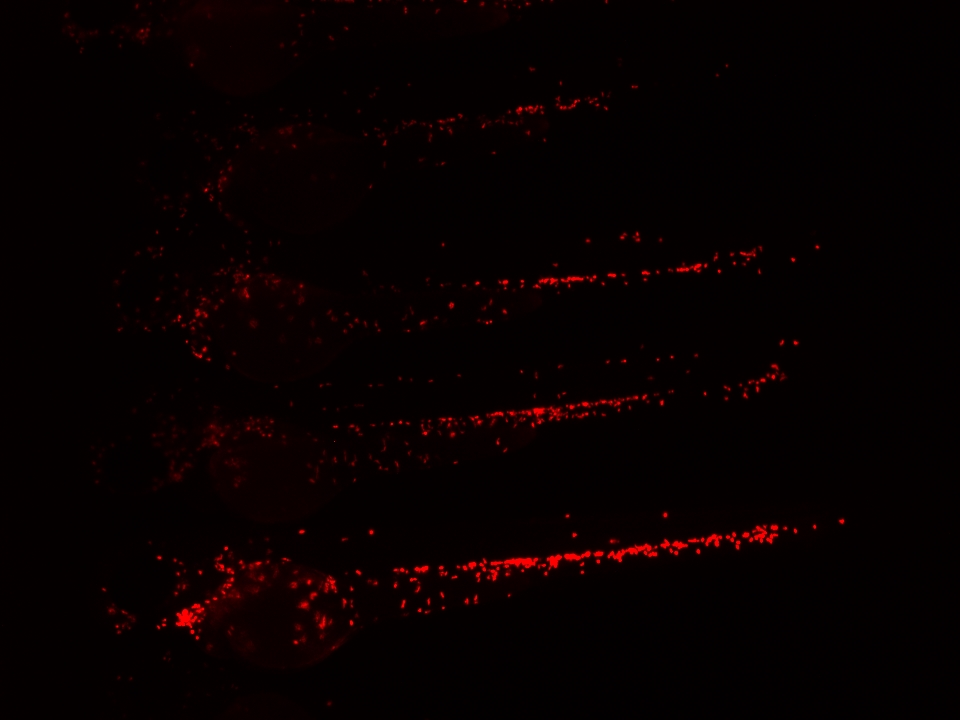

Supplement: Supplementary file 8 — Source data Fig. 4 [file 44321_2025_368_MOESM8_ESM.zip › FIGURE_4/4B/BOSUTINIB_1uM (9).jpg]

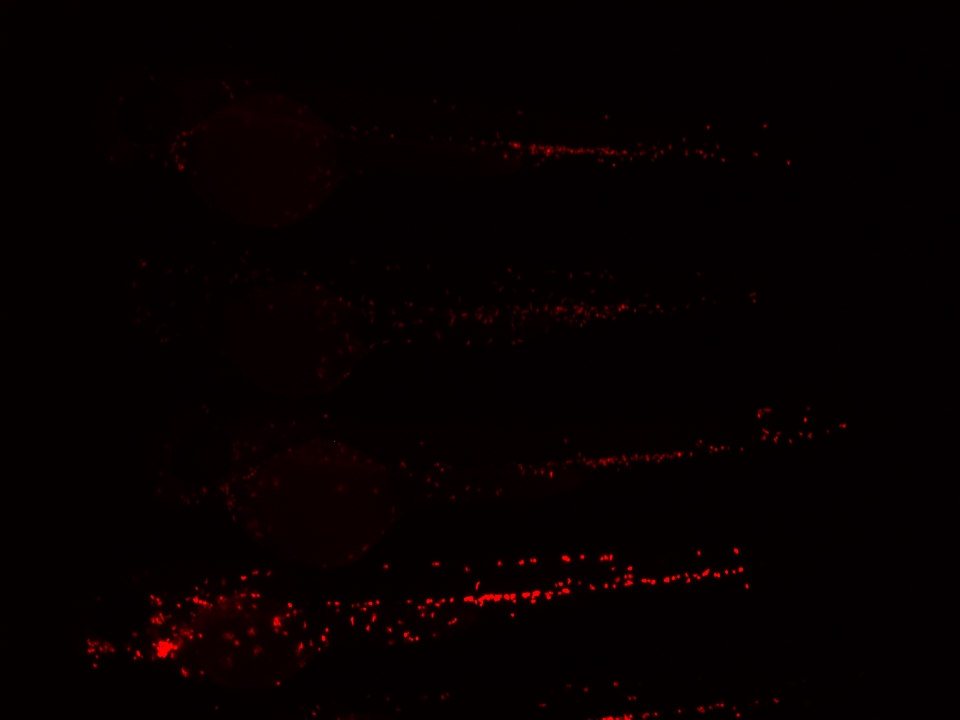

Supplement: Supplementary file 8 — Source data Fig. 4 [file 44321_2025_368_MOESM8_ESM.zip › FIGURE_4/4B/DASATINIB_01uM (1).jpg]

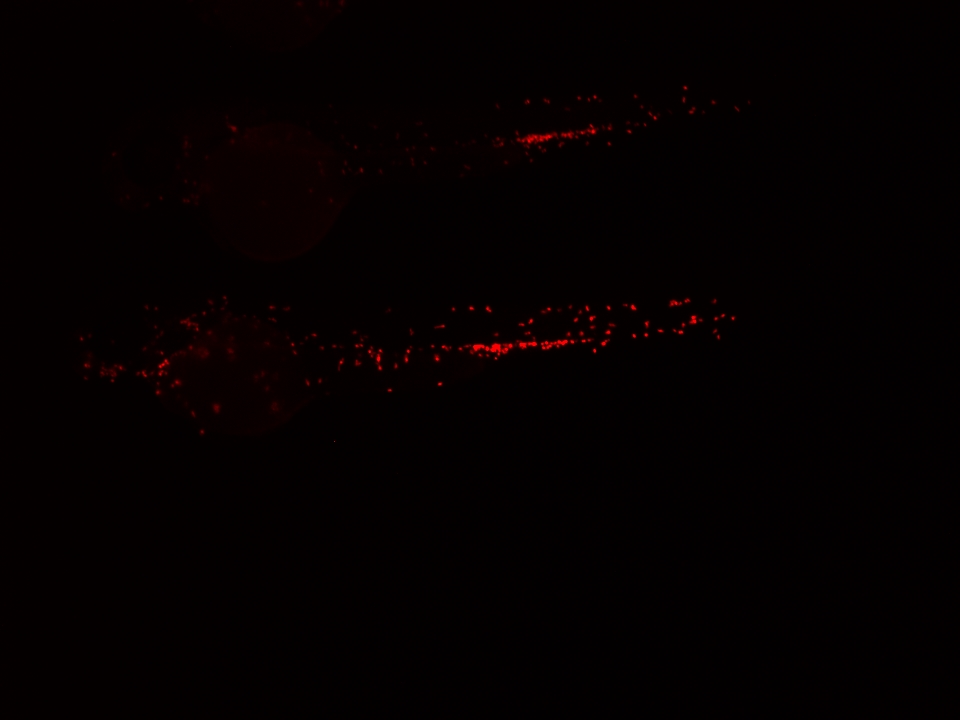

Supplement: Supplementary file 8 — Source data Fig. 4 [file 44321_2025_368_MOESM8_ESM.zip › FIGURE_4/4B/DASATINIB_01uM (10).jpg]

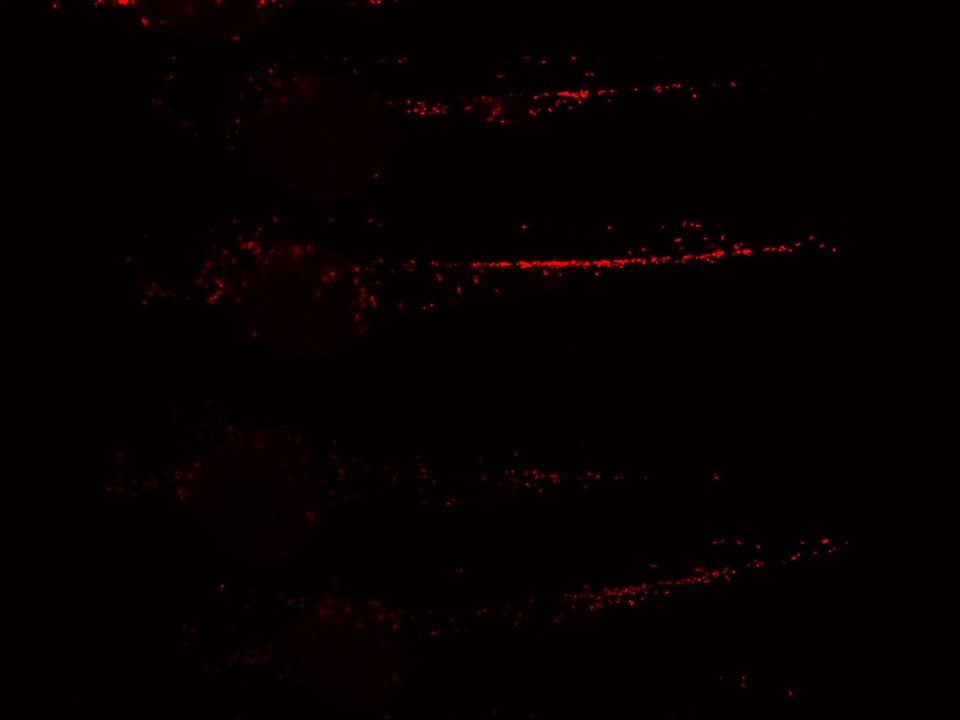

Supplement: Supplementary file 8 — Source data Fig. 4 [file 44321_2025_368_MOESM8_ESM.zip › FIGURE_4/4B/DASATINIB_01uM (2).jpg]

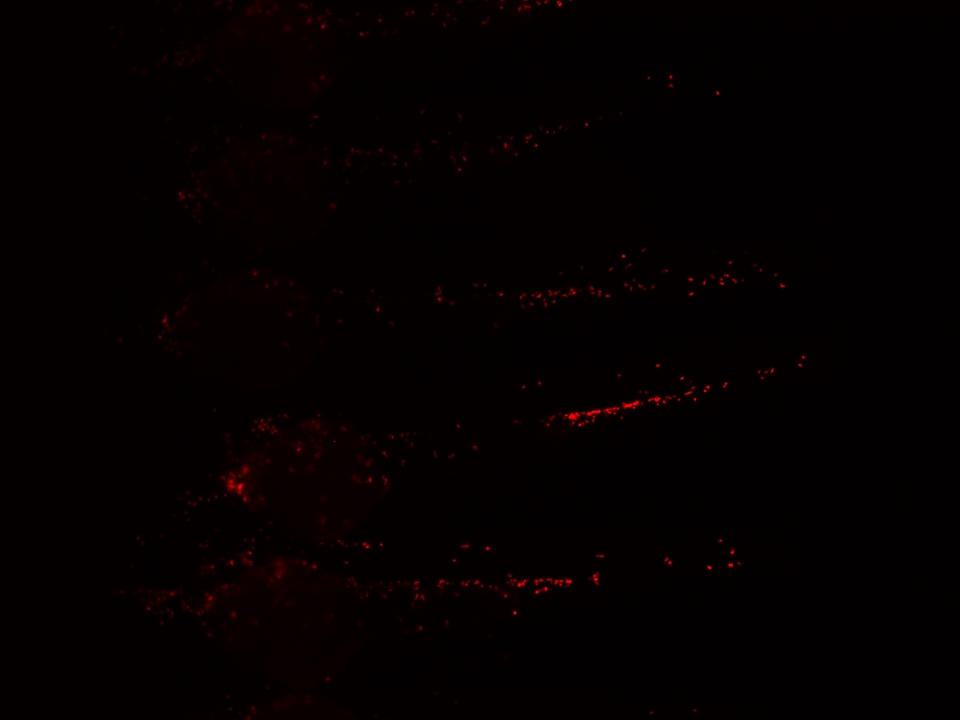

Supplement: Supplementary file 8 — Source data Fig. 4 [file 44321_2025_368_MOESM8_ESM.zip › FIGURE_4/4B/DASATINIB_01uM (3).jpg]

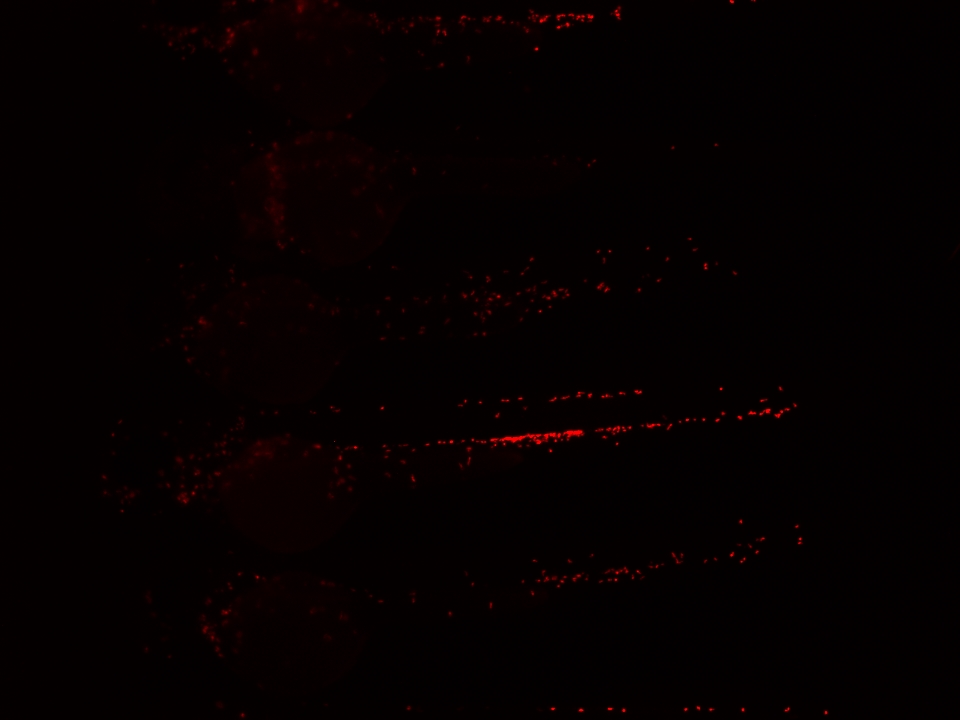

Supplement: Supplementary file 8 — Source data Fig. 4 [file 44321_2025_368_MOESM8_ESM.zip › FIGURE_4/4B/DASATINIB_01uM (4).jpg]

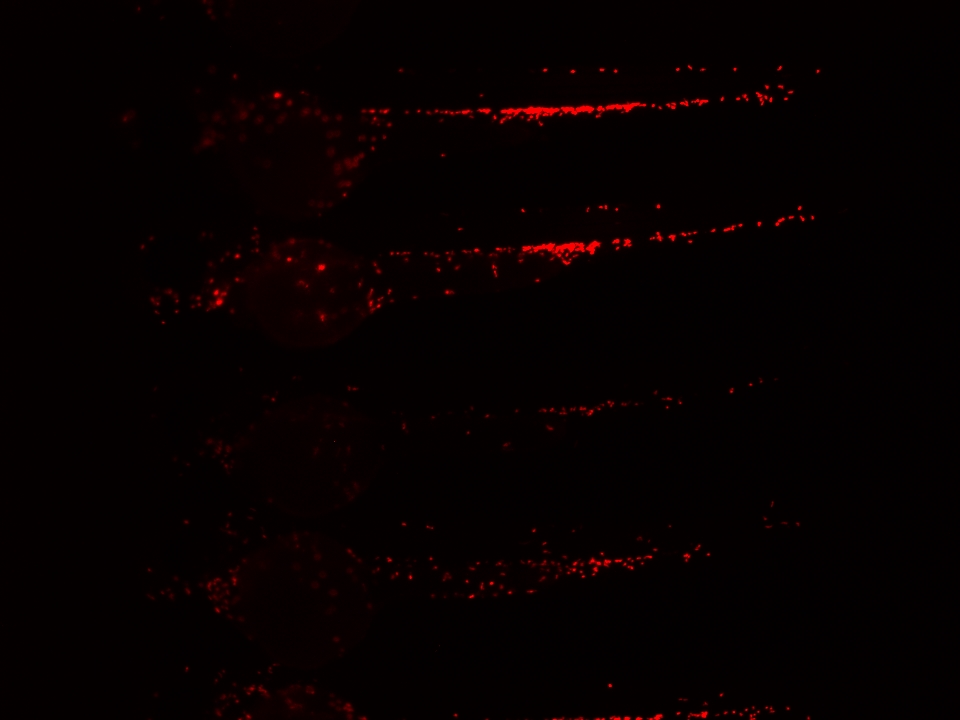

Supplement: Supplementary file 8 — Source data Fig. 4 [file 44321_2025_368_MOESM8_ESM.zip › FIGURE_4/4B/DASATINIB_01uM (5).jpg]

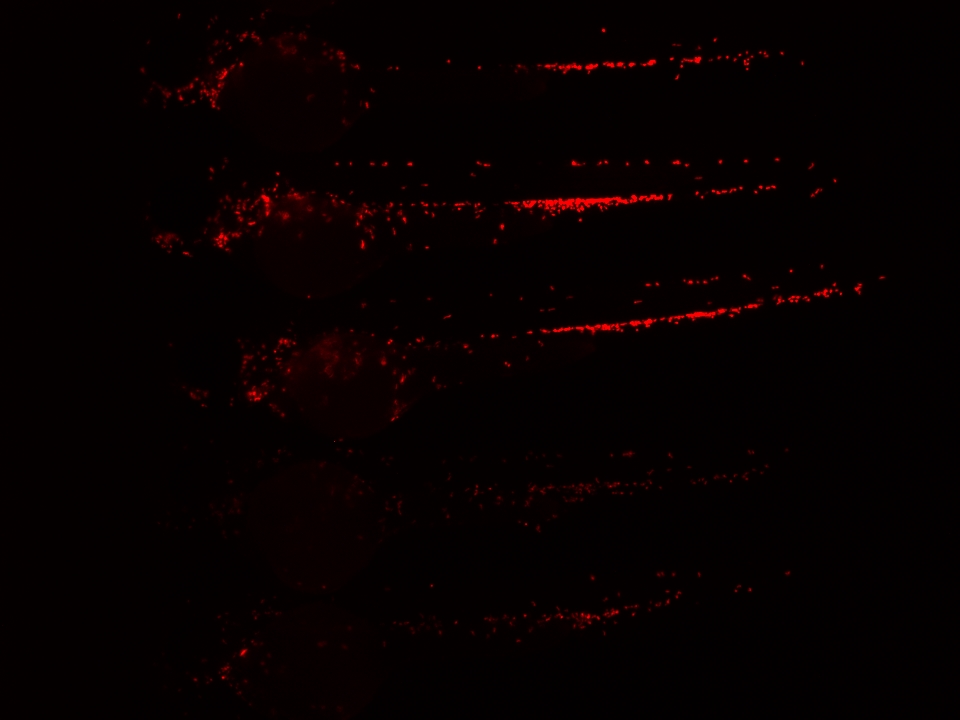

Supplement: Supplementary file 8 — Source data Fig. 4 [file 44321_2025_368_MOESM8_ESM.zip › FIGURE_4/4B/DASATINIB_01uM (6).jpg]

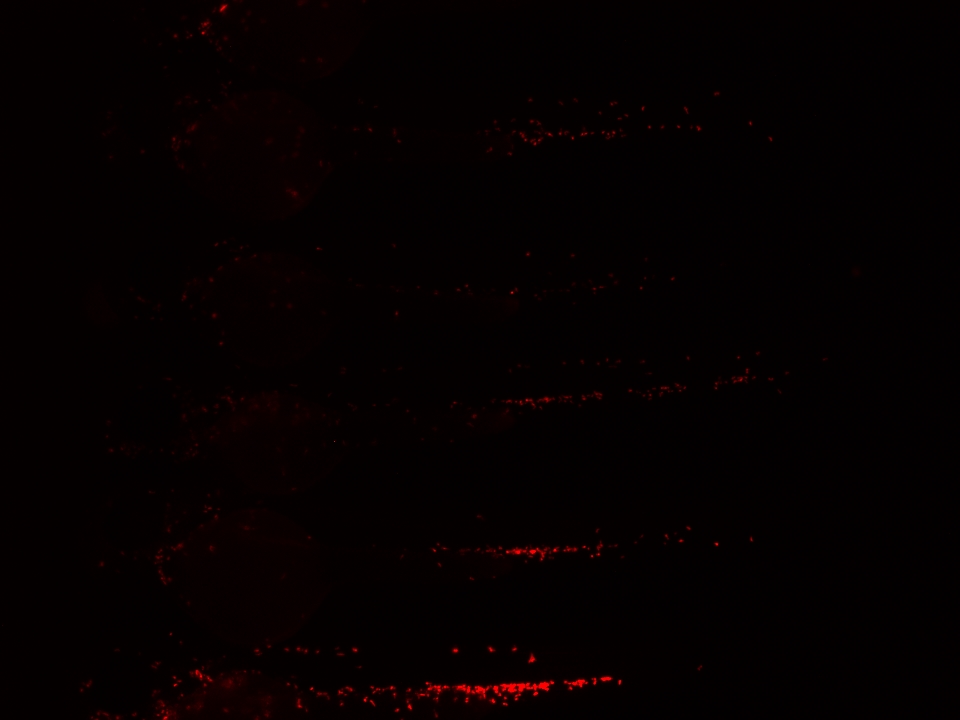

Supplement: Supplementary file 8 — Source data Fig. 4 [file 44321_2025_368_MOESM8_ESM.zip › FIGURE_4/4B/DASATINIB_01uM (7).jpg]

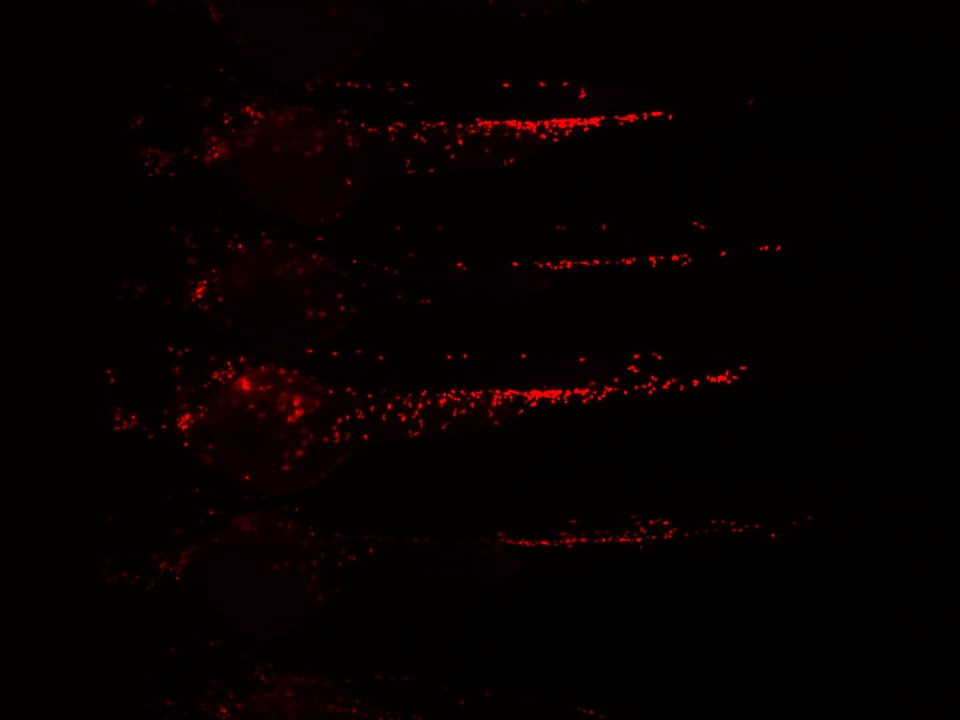

Supplement: Supplementary file 8 — Source data Fig. 4 [file 44321_2025_368_MOESM8_ESM.zip › FIGURE_4/4B/DASATINIB_01uM (8).jpg]

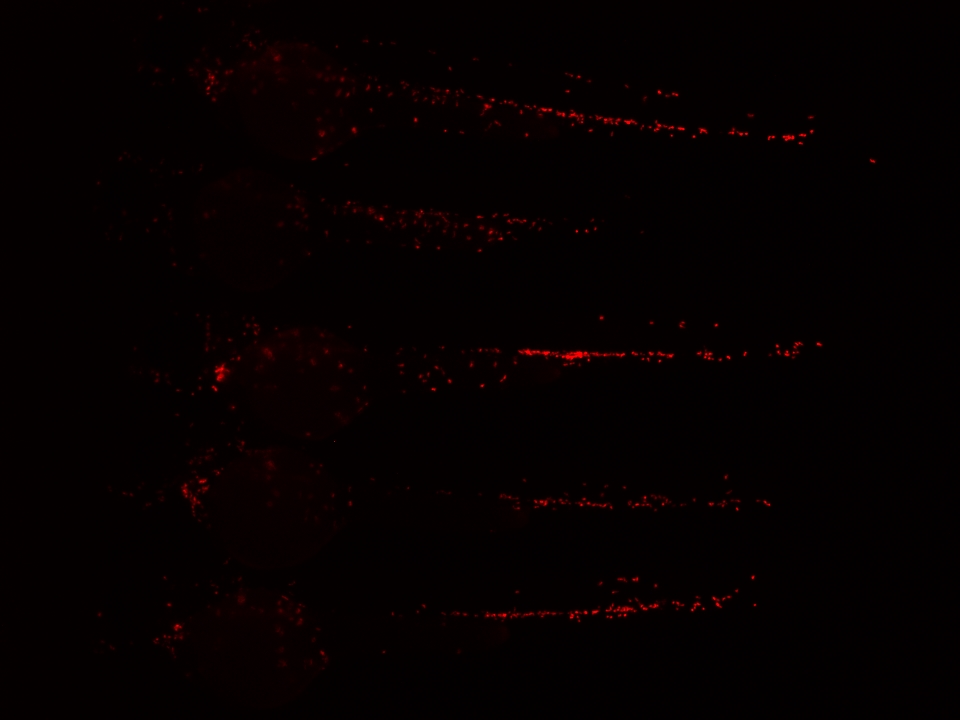

Supplement: Supplementary file 8 — Source data Fig. 4 [file 44321_2025_368_MOESM8_ESM.zip › FIGURE_4/4B/DASATINIB_01uM (9).jpg]

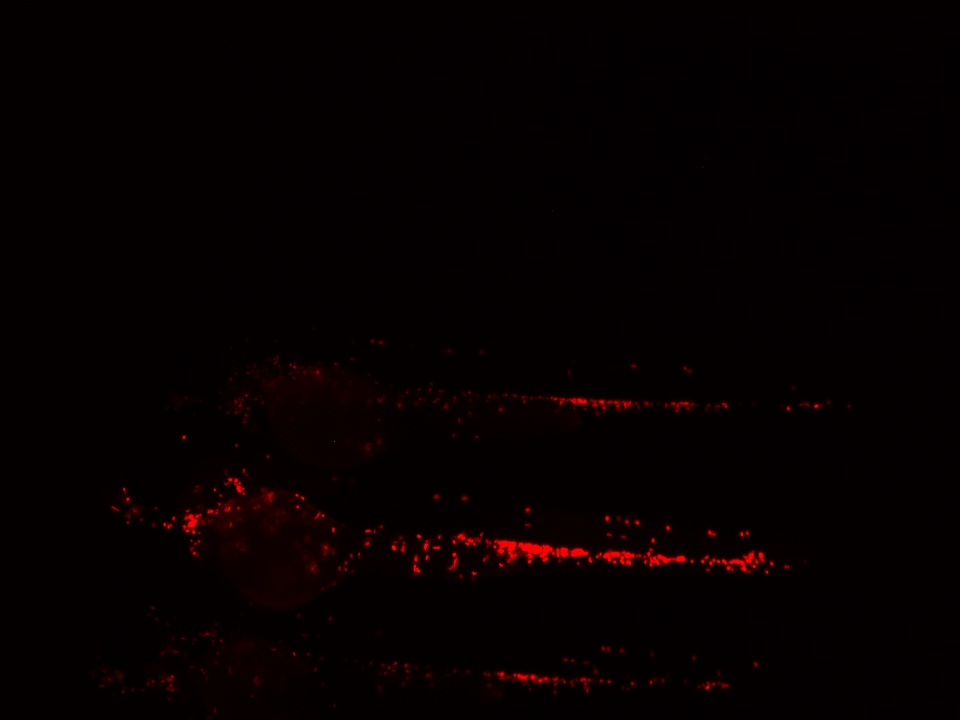

Supplement: Supplementary file 8 — Source data Fig. 4 [file 44321_2025_368_MOESM8_ESM.zip › FIGURE_4/4B/DASATINIB_1uM (1).jpg]

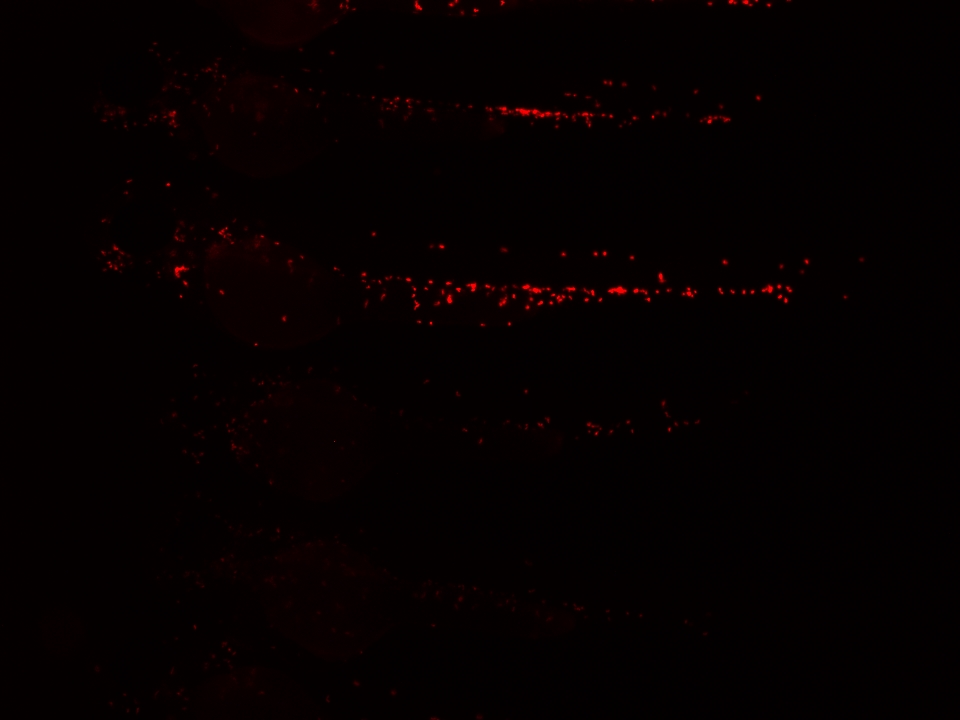

Supplement: Supplementary file 8 — Source data Fig. 4 [file 44321_2025_368_MOESM8_ESM.zip › FIGURE_4/4B/DASATINIB_1uM (10).jpg]

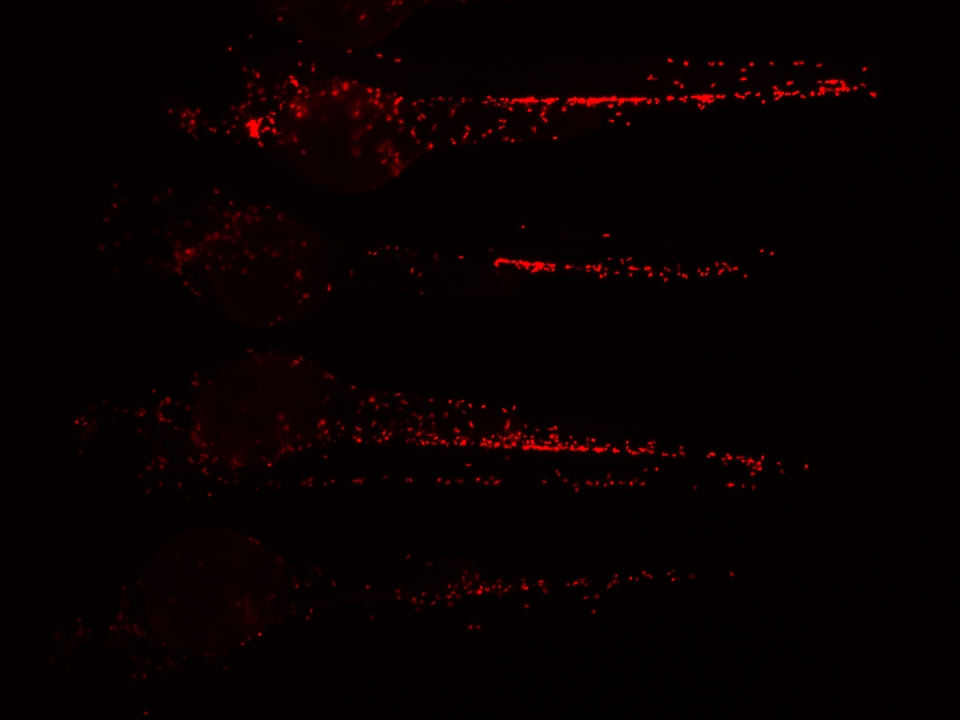

Supplement: Supplementary file 8 — Source data Fig. 4 [file 44321_2025_368_MOESM8_ESM.zip › FIGURE_4/4B/DASATINIB_1uM (2).jpg]

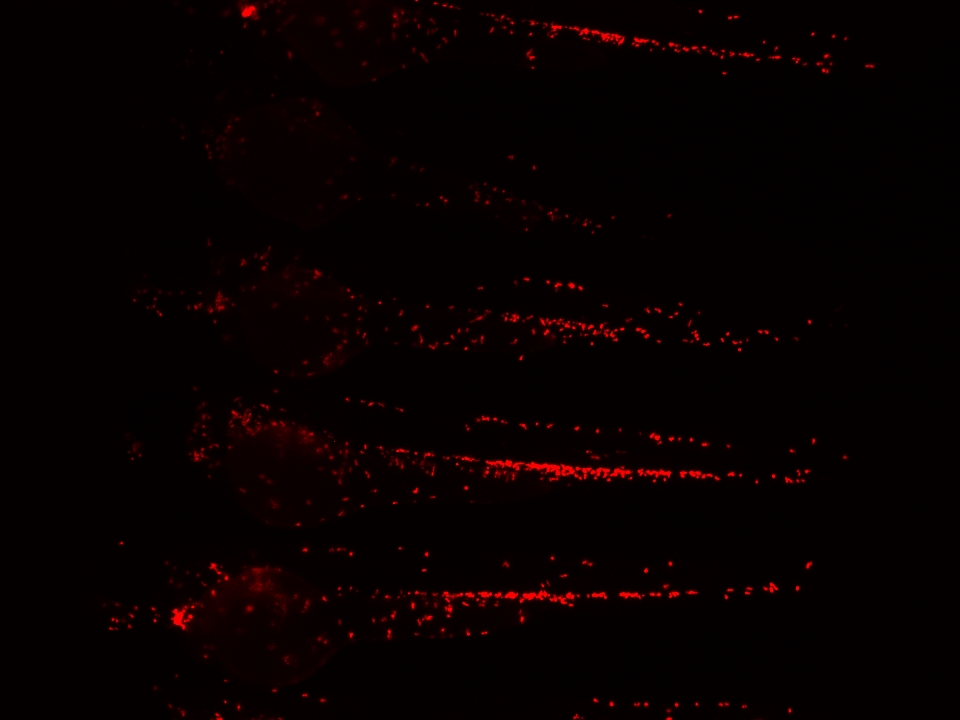

Supplement: Supplementary file 8 — Source data Fig. 4 [file 44321_2025_368_MOESM8_ESM.zip › FIGURE_4/4B/DASATINIB_1uM (3).jpg]

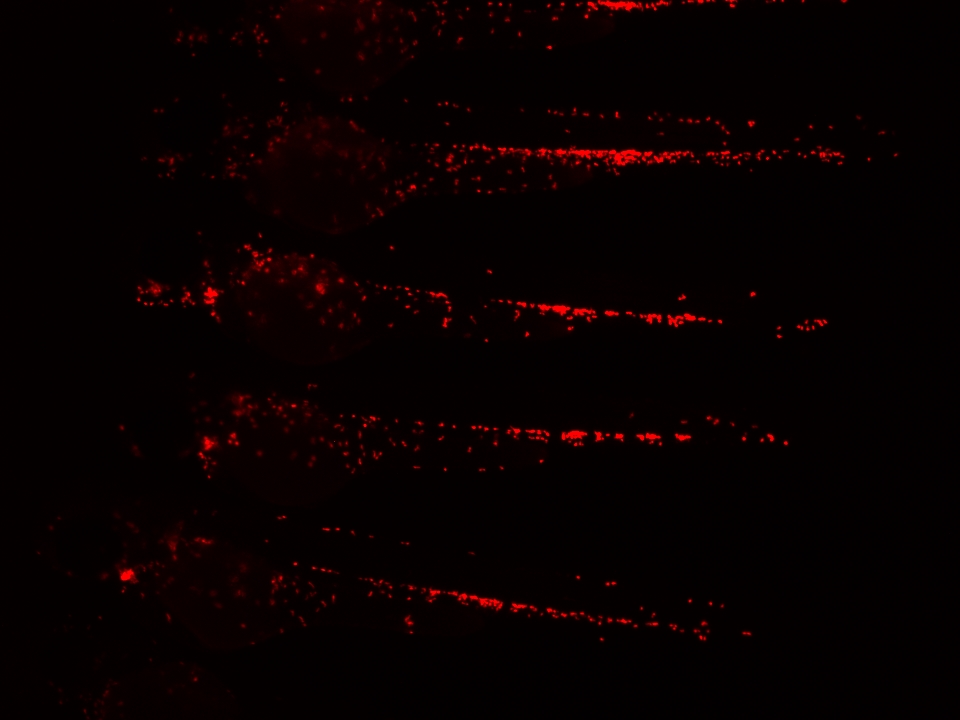

Supplement: Supplementary file 8 — Source data Fig. 4 [file 44321_2025_368_MOESM8_ESM.zip › FIGURE_4/4B/DASATINIB_1uM (4).jpg]

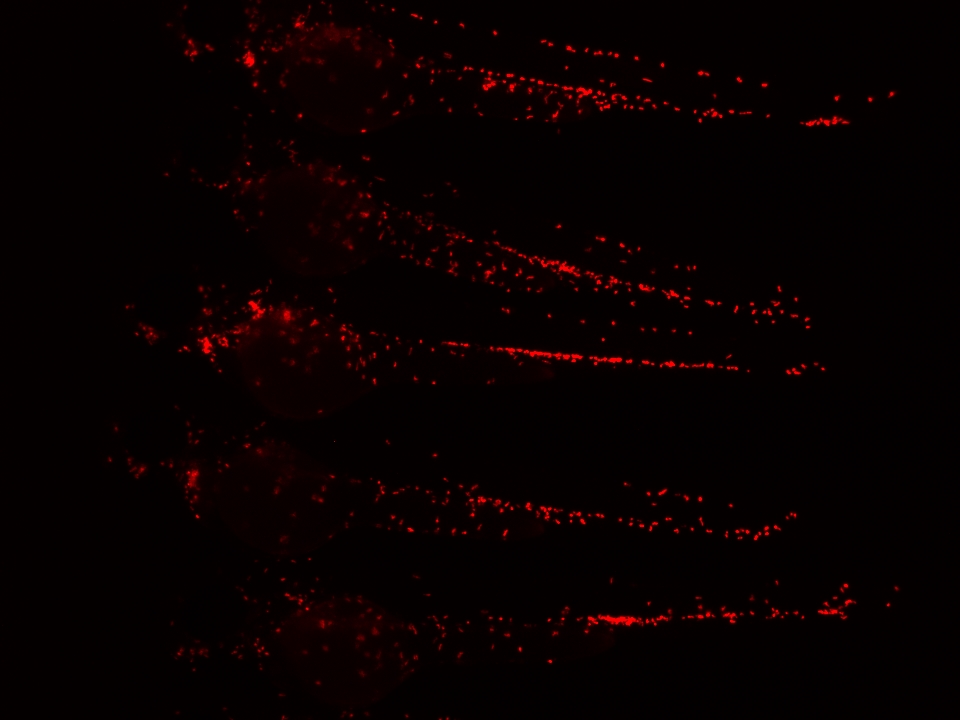

Supplement: Supplementary file 8 — Source data Fig. 4 [file 44321_2025_368_MOESM8_ESM.zip › FIGURE_4/4B/DASATINIB_1uM (5).jpg]

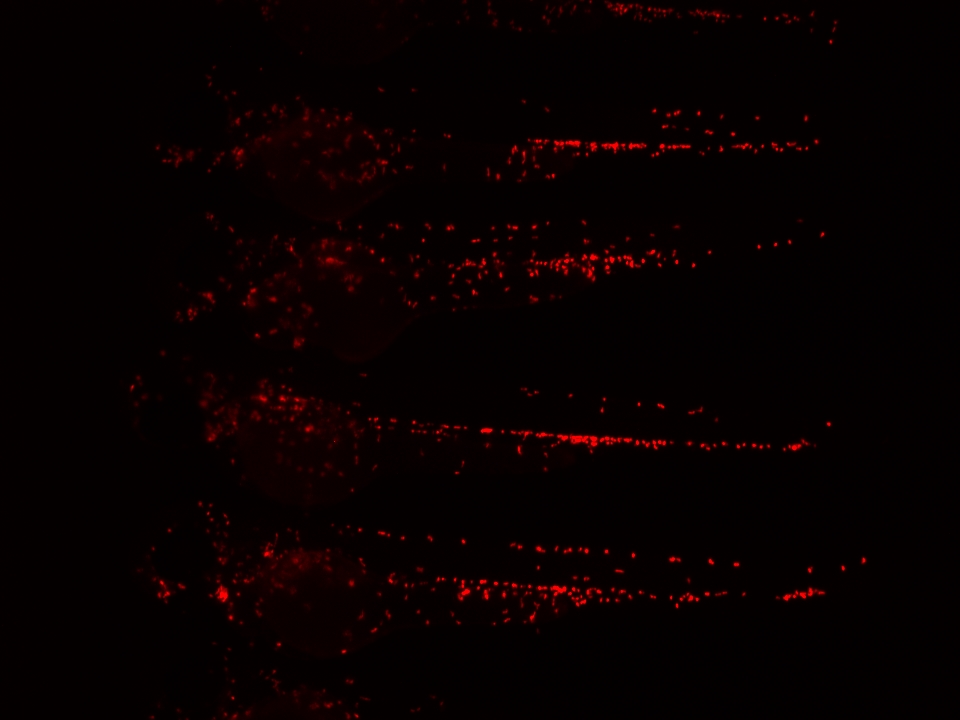

Supplement: Supplementary file 8 — Source data Fig. 4 [file 44321_2025_368_MOESM8_ESM.zip › FIGURE_4/4B/DASATINIB_1uM (6).jpg]

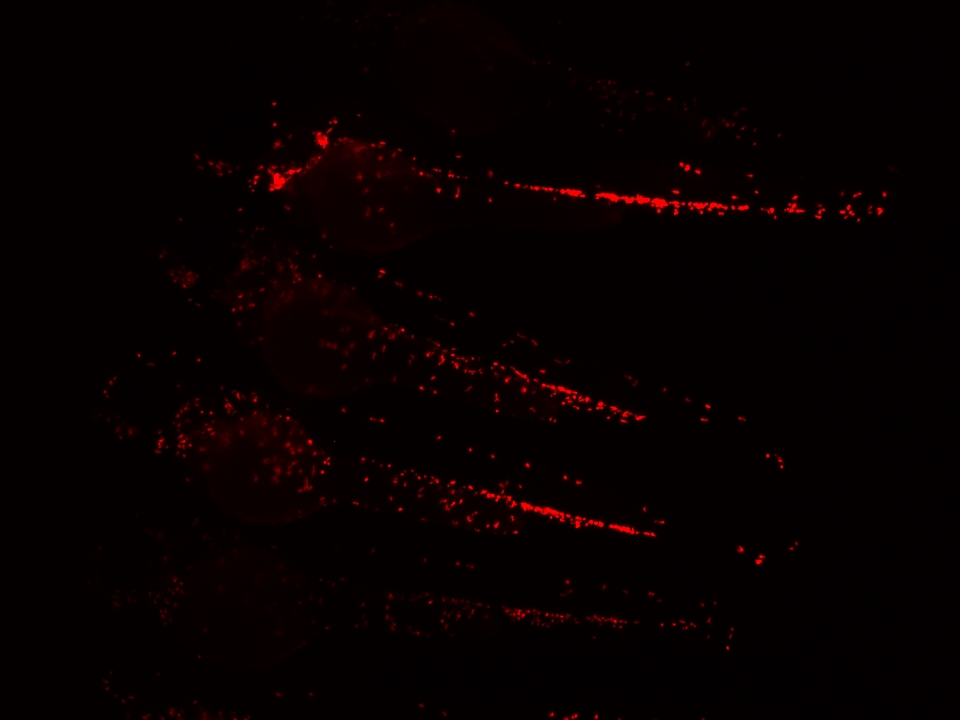

Supplement: Supplementary file 8 — Source data Fig. 4 [file 44321_2025_368_MOESM8_ESM.zip › FIGURE_4/4B/DASATINIB_1uM (7).jpg]

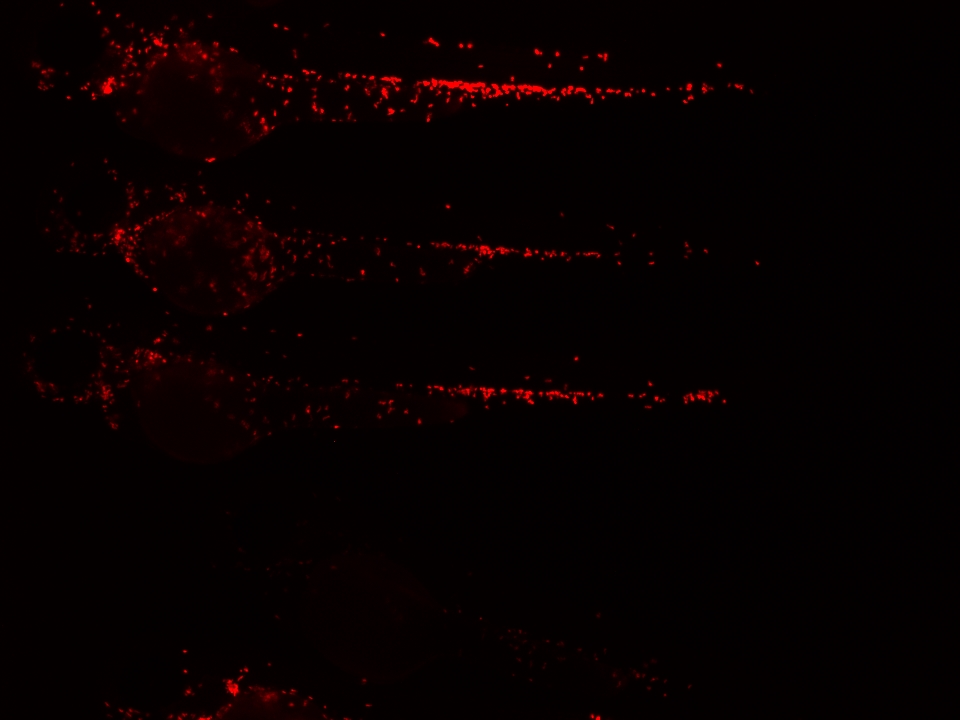

Supplement: Supplementary file 8 — Source data Fig. 4 [file 44321_2025_368_MOESM8_ESM.zip › FIGURE_4/4B/DASATINIB_1uM (8).jpg]

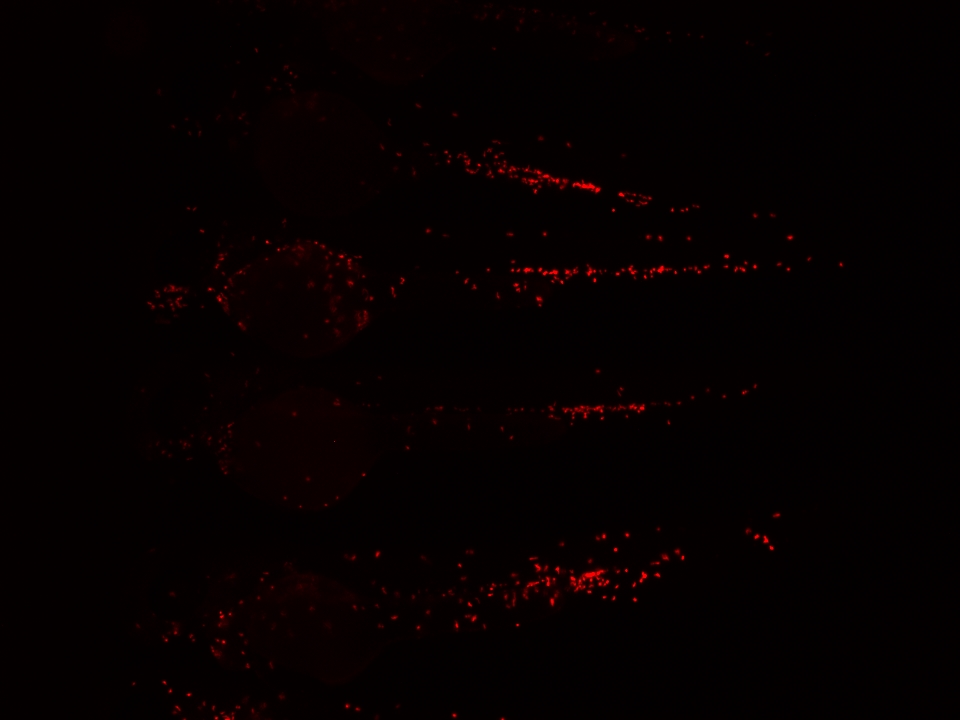

Supplement: Supplementary file 8 — Source data Fig. 4 [file 44321_2025_368_MOESM8_ESM.zip › FIGURE_4/4B/DASATINIB_1uM (9).jpg]

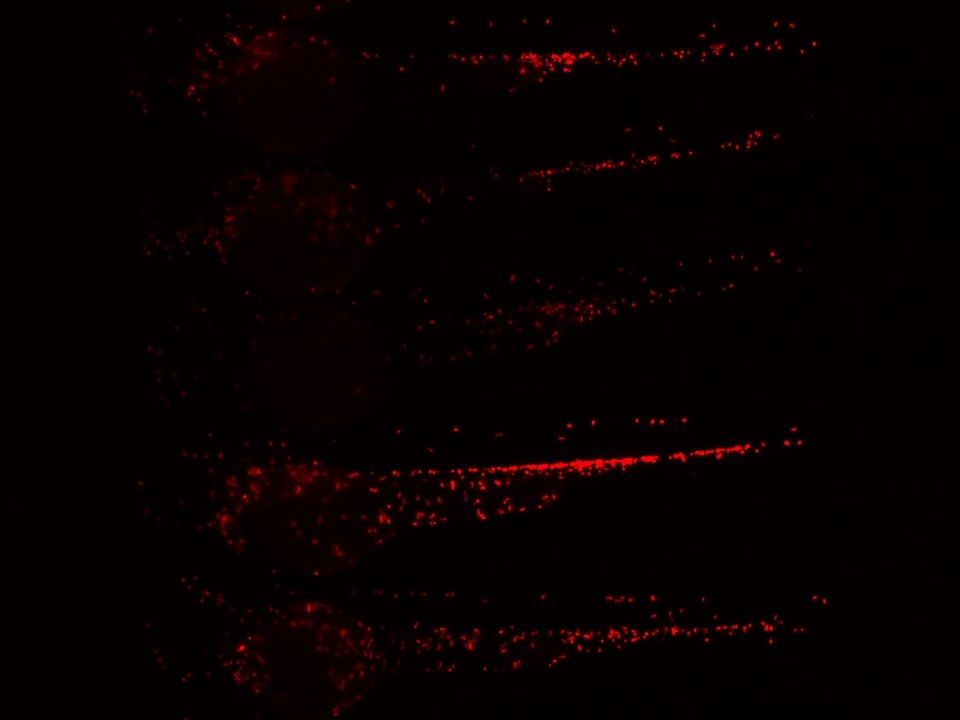

Supplement: Supplementary file 8 — Source data Fig. 4 [file 44321_2025_368_MOESM8_ESM.zip › FIGURE_4/4B/DMSO (1).jpg]

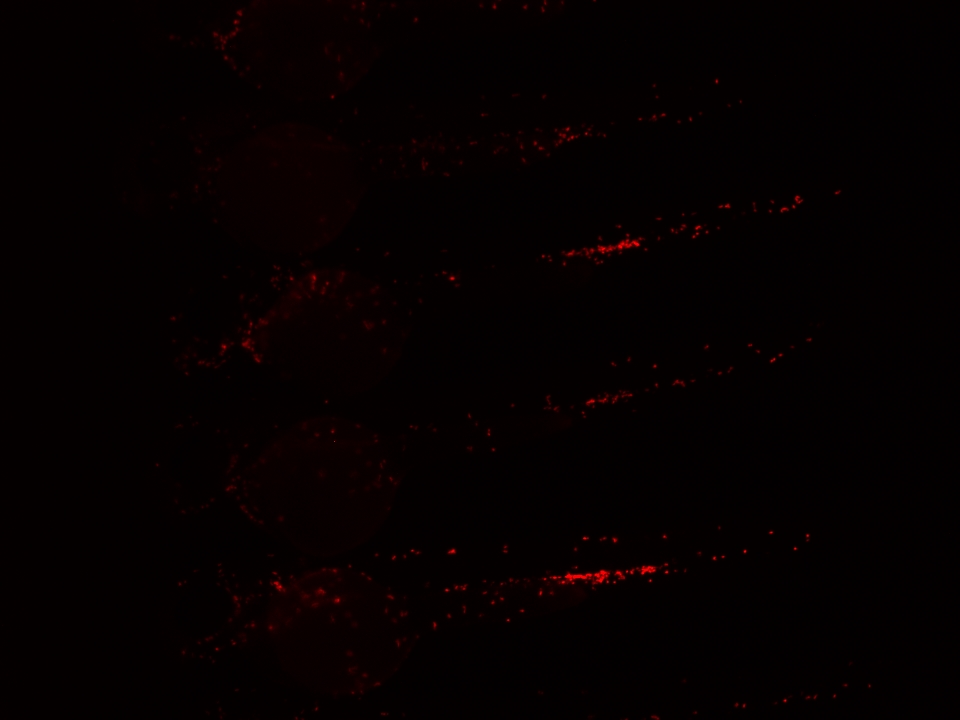

Supplement: Supplementary file 8 — Source data Fig. 4 [file 44321_2025_368_MOESM8_ESM.zip › FIGURE_4/4B/DMSO (10).jpg]

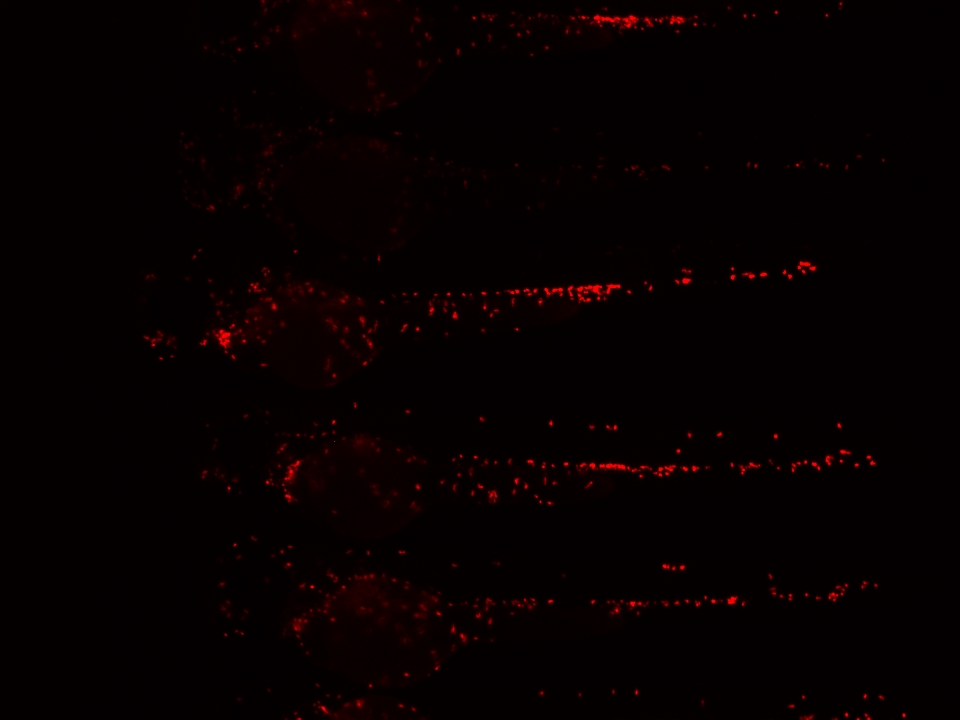

Supplement: Supplementary file 8 — Source data Fig. 4 [file 44321_2025_368_MOESM8_ESM.zip › FIGURE_4/4B/DMSO (11).jpg]

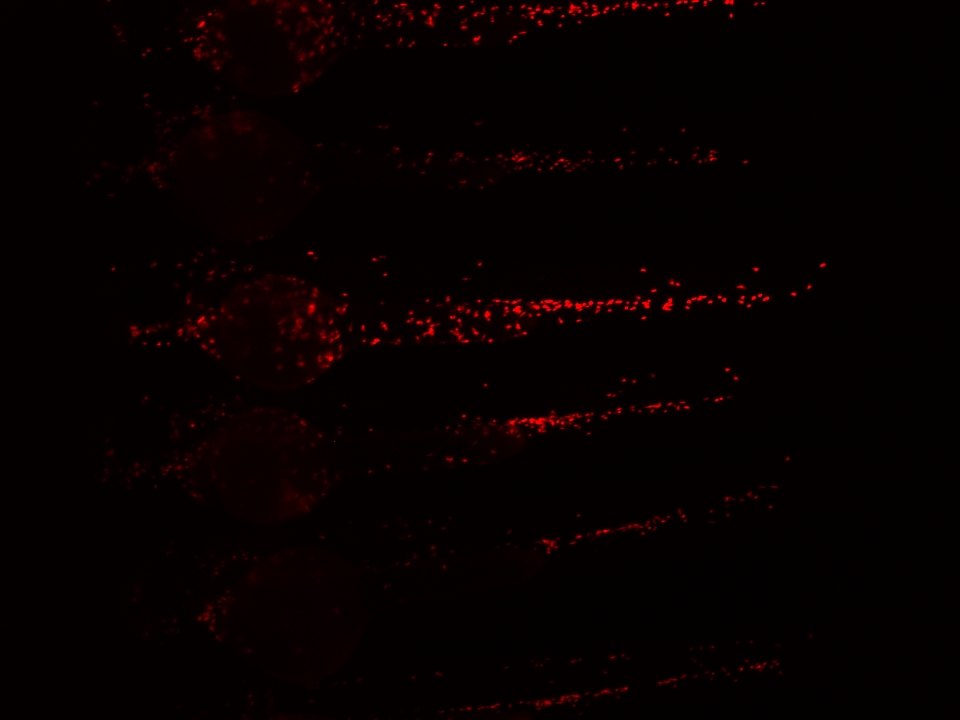

Supplement: Supplementary file 8 — Source data Fig. 4 [file 44321_2025_368_MOESM8_ESM.zip › FIGURE_4/4B/DMSO (2).jpg]

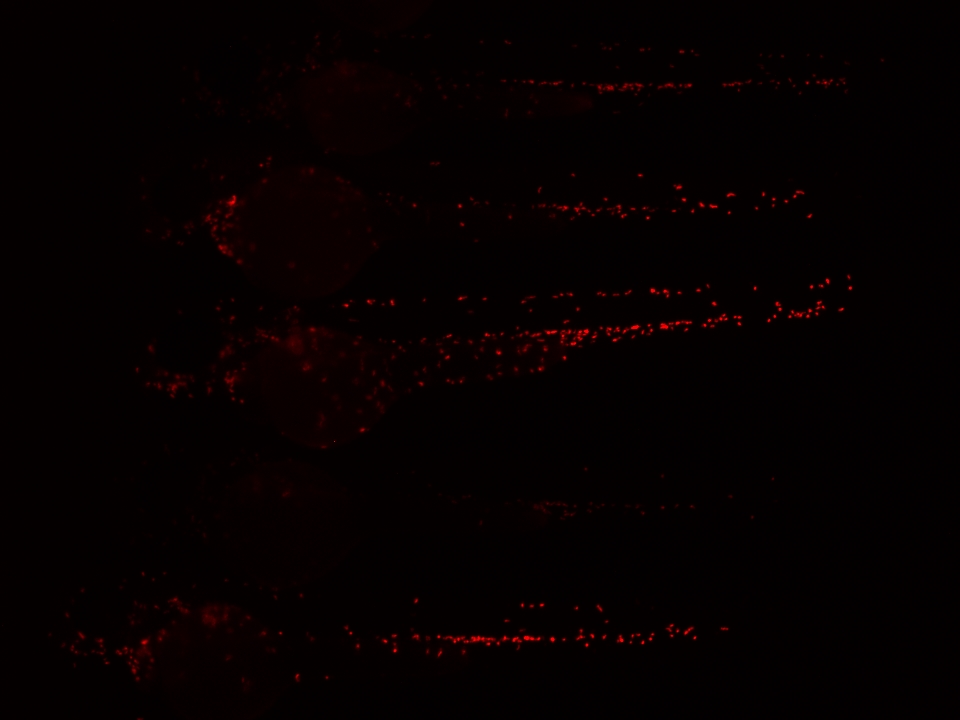

Supplement: Supplementary file 8 — Source data Fig. 4 [file 44321_2025_368_MOESM8_ESM.zip › FIGURE_4/4B/DMSO (3).jpg]

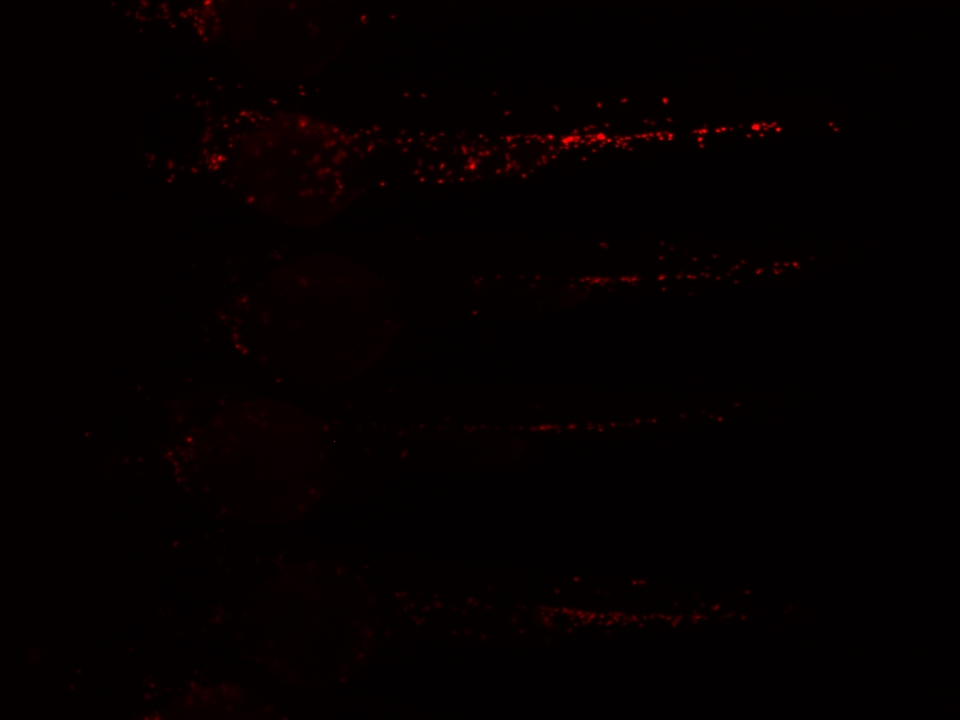

Supplement: Supplementary file 8 — Source data Fig. 4 [file 44321_2025_368_MOESM8_ESM.zip › FIGURE_4/4B/DMSO (4).jpg]

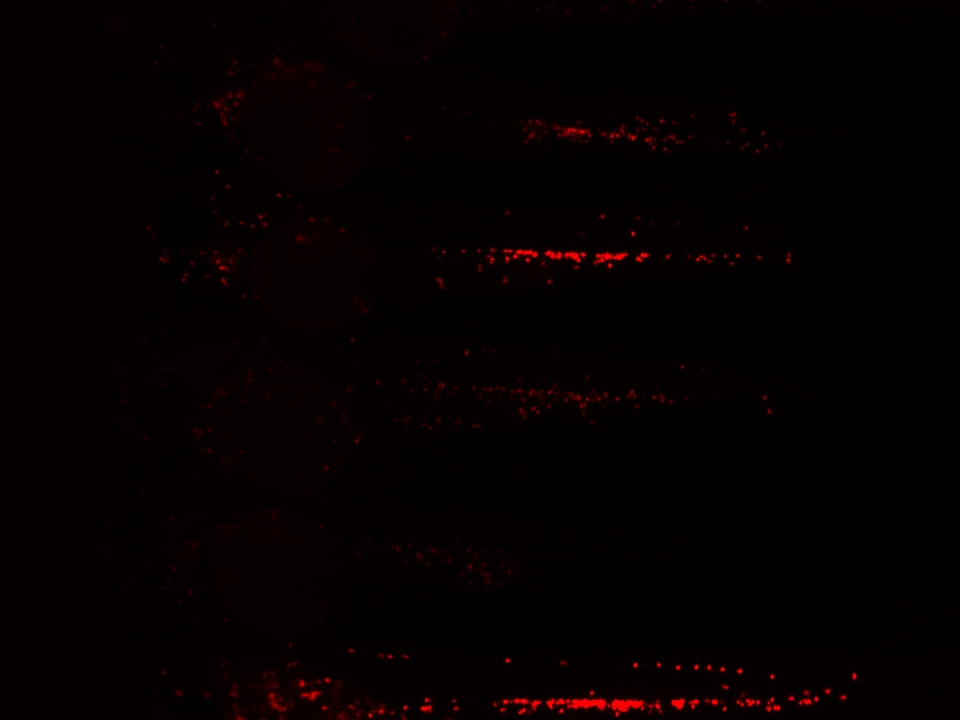

Supplement: Supplementary file 8 — Source data Fig. 4 [file 44321_2025_368_MOESM8_ESM.zip › FIGURE_4/4B/DMSO (5).jpg]

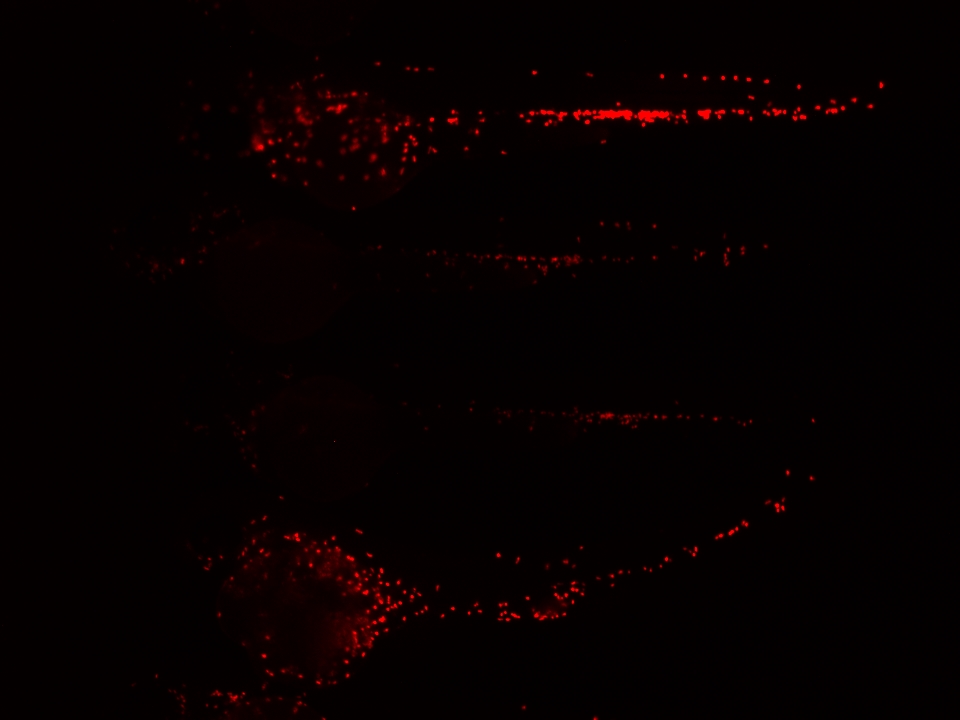

Supplement: Supplementary file 8 — Source data Fig. 4 [file 44321_2025_368_MOESM8_ESM.zip › FIGURE_4/4B/DMSO (6).jpg]

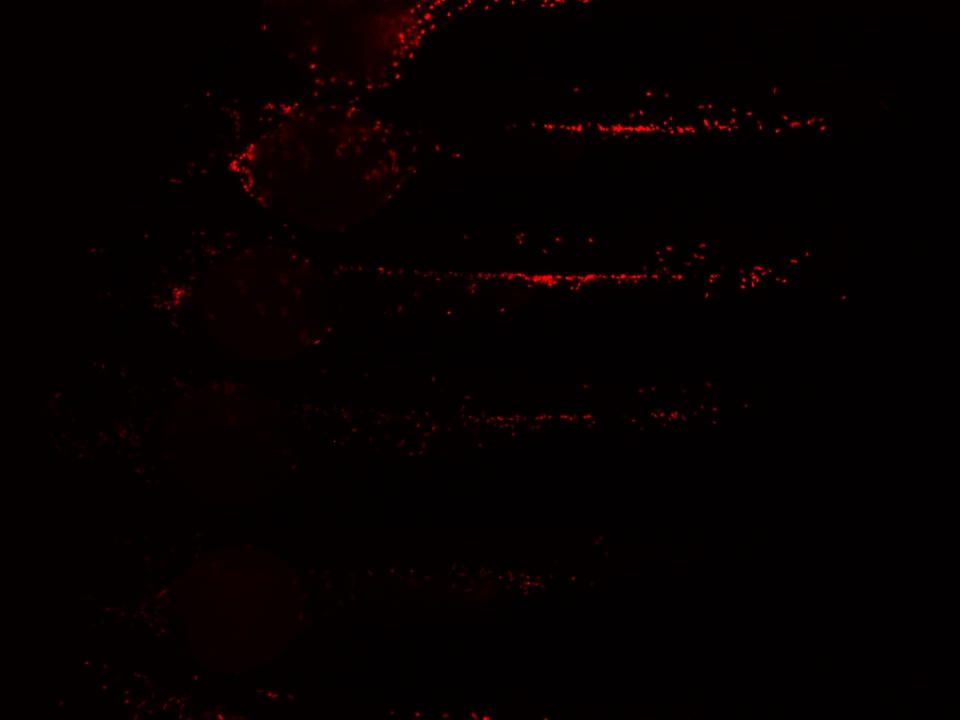

Supplement: Supplementary file 8 — Source data Fig. 4 [file 44321_2025_368_MOESM8_ESM.zip › FIGURE_4/4B/DMSO (7).jpg]

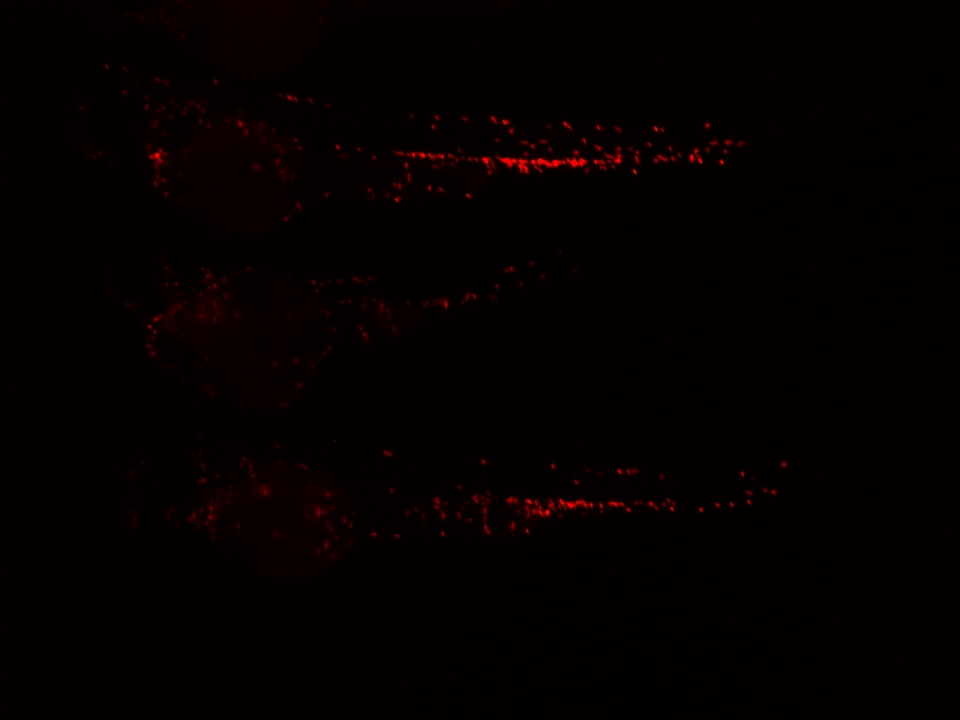

Supplement: Supplementary file 8 — Source data Fig. 4 [file 44321_2025_368_MOESM8_ESM.zip › FIGURE_4/4B/DMSO (8).jpg]

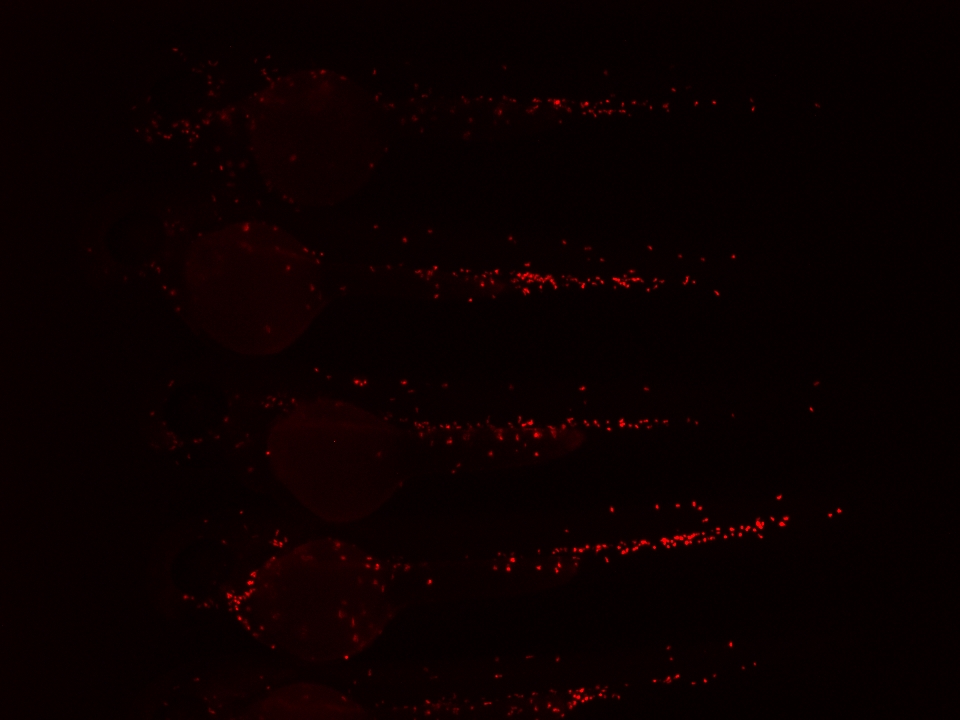

Supplement: Supplementary file 8 — Source data Fig. 4 [file 44321_2025_368_MOESM8_ESM.zip › FIGURE_4/4B/DMSO (9).jpg]

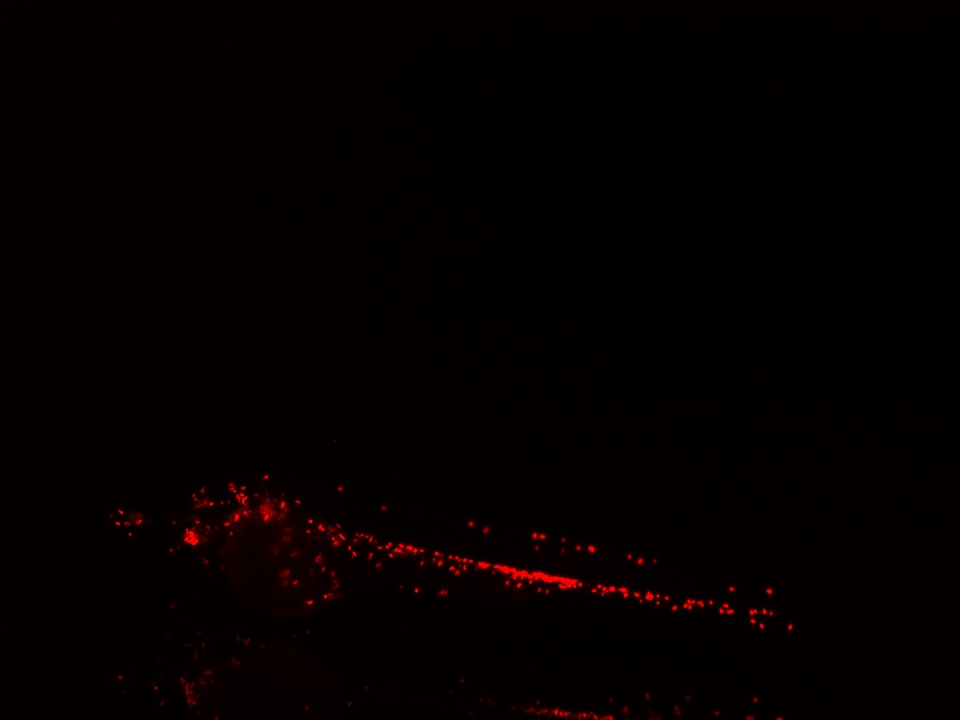

Supplement: Supplementary file 8 — Source data Fig. 4 [file 44321_2025_368_MOESM8_ESM.zip › FIGURE_4/4B/IMATINIB_1uM (1).jpg]

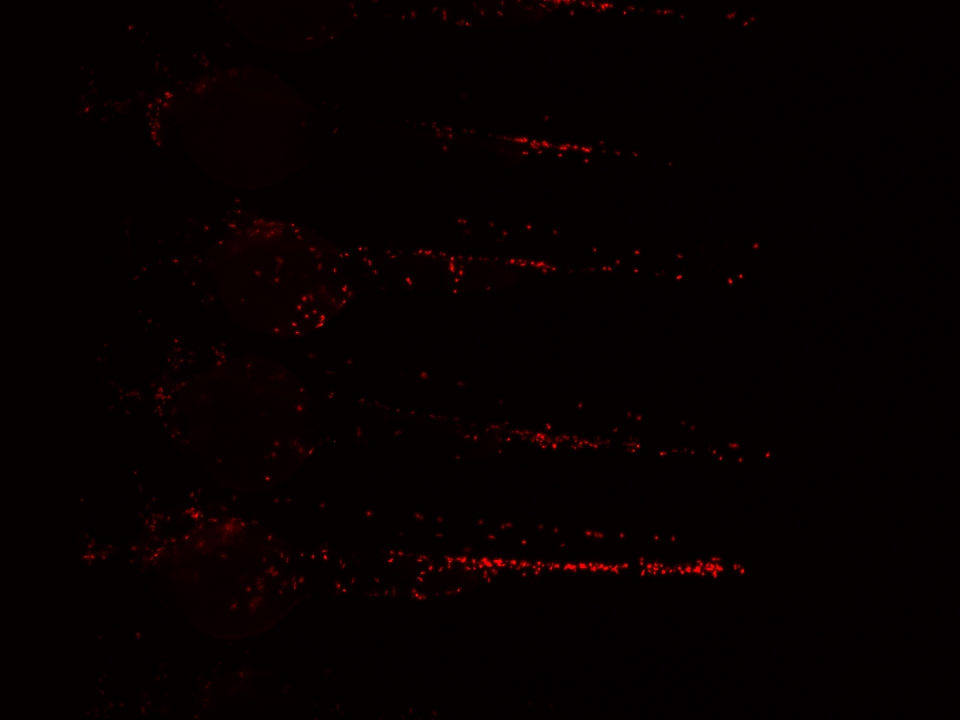

Supplement: Supplementary file 8 — Source data Fig. 4 [file 44321_2025_368_MOESM8_ESM.zip › FIGURE_4/4B/IMATINIB_1uM (10).jpg]

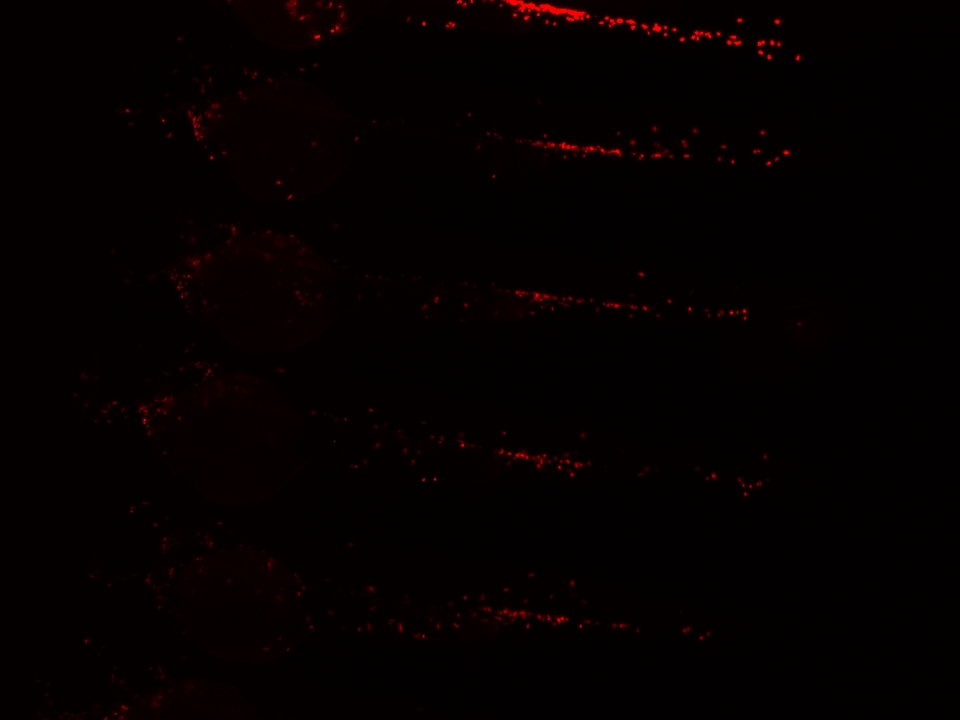

Supplement: Supplementary file 8 — Source data Fig. 4 [file 44321_2025_368_MOESM8_ESM.zip › FIGURE_4/4B/IMATINIB_1uM (11).jpg]

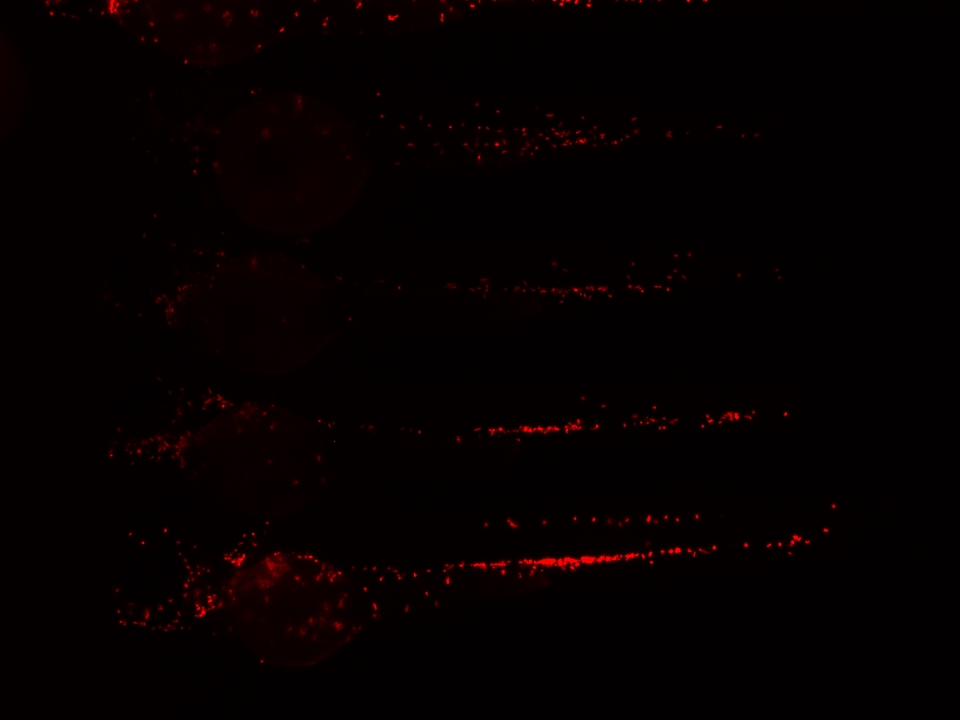

Supplement: Supplementary file 8 — Source data Fig. 4 [file 44321_2025_368_MOESM8_ESM.zip › FIGURE_4/4B/IMATINIB_1uM (2).jpg]

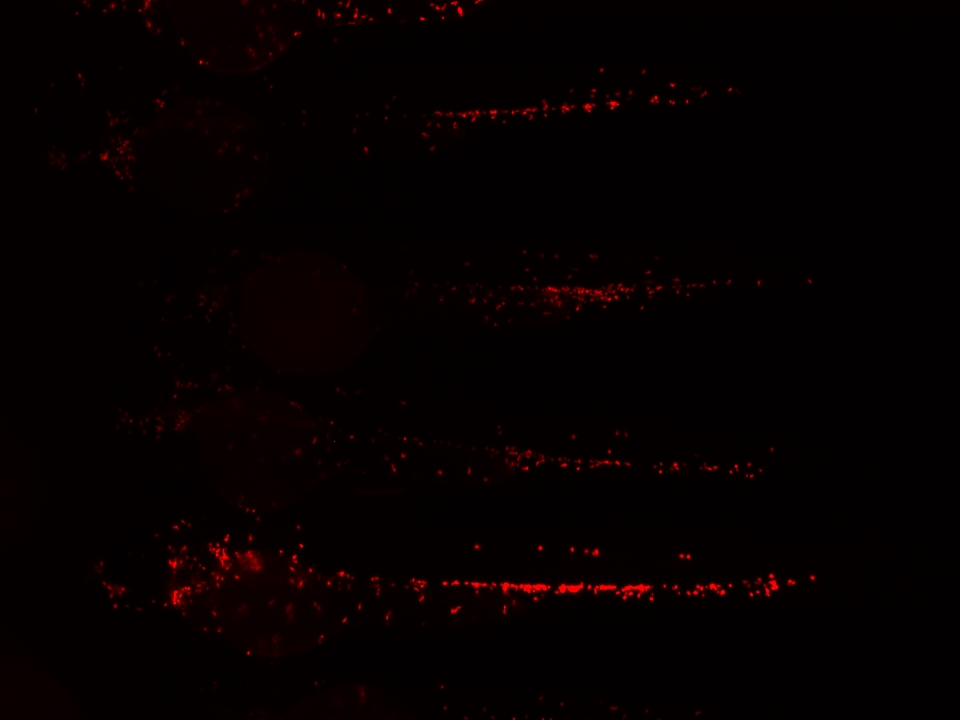

Supplement: Supplementary file 8 — Source data Fig. 4 [file 44321_2025_368_MOESM8_ESM.zip › FIGURE_4/4B/IMATINIB_1uM (3).jpg]

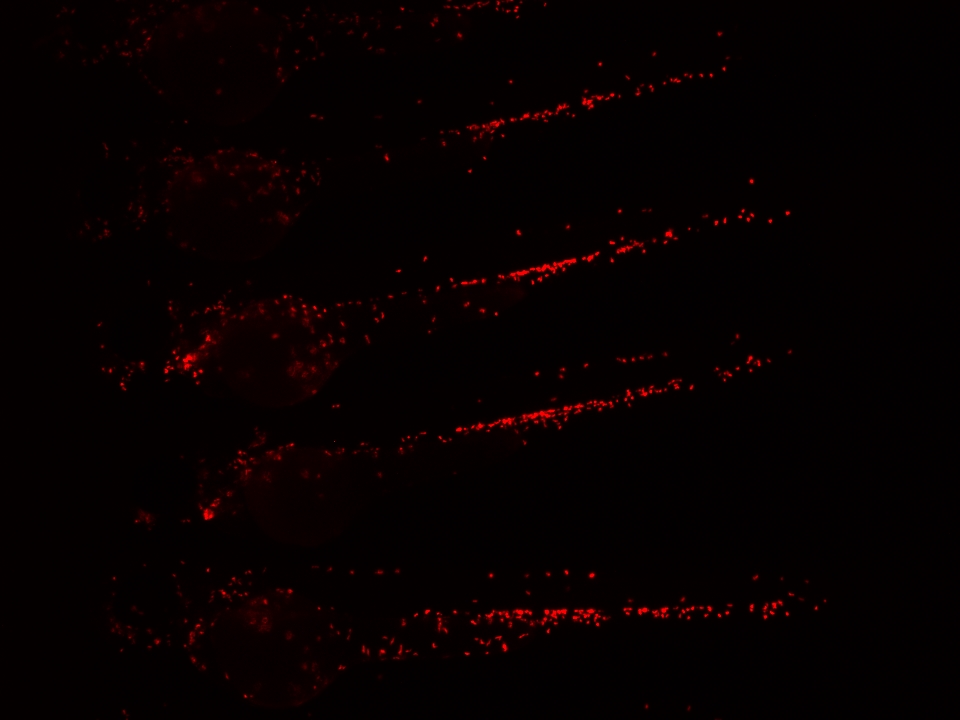

Supplement: Supplementary file 8 — Source data Fig. 4 [file 44321_2025_368_MOESM8_ESM.zip › FIGURE_4/4B/IMATINIB_1uM (4).jpg]

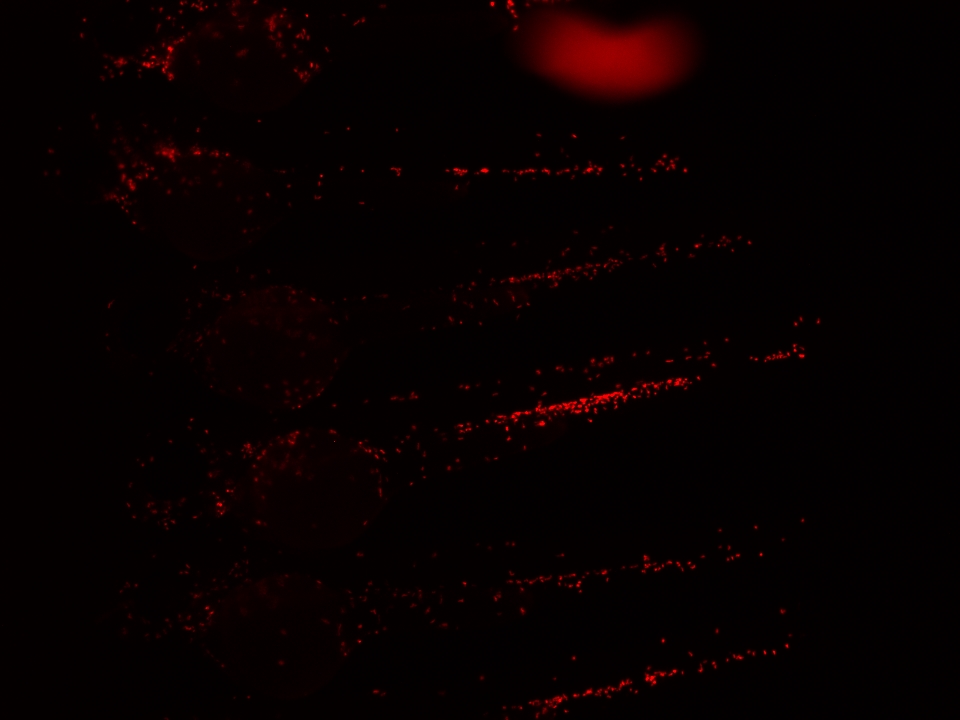

Supplement: Supplementary file 8 — Source data Fig. 4 [file 44321_2025_368_MOESM8_ESM.zip › FIGURE_4/4B/IMATINIB_1uM (5).jpg]

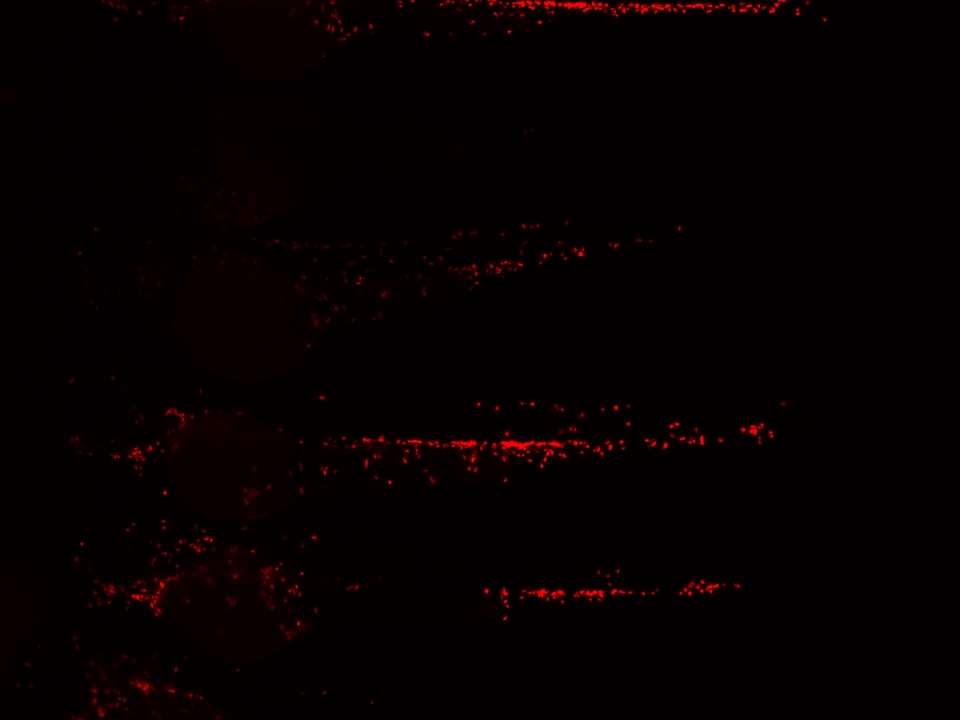

Supplement: Supplementary file 8 — Source data Fig. 4 [file 44321_2025_368_MOESM8_ESM.zip › FIGURE_4/4B/IMATINIB_1uM (6).jpg]

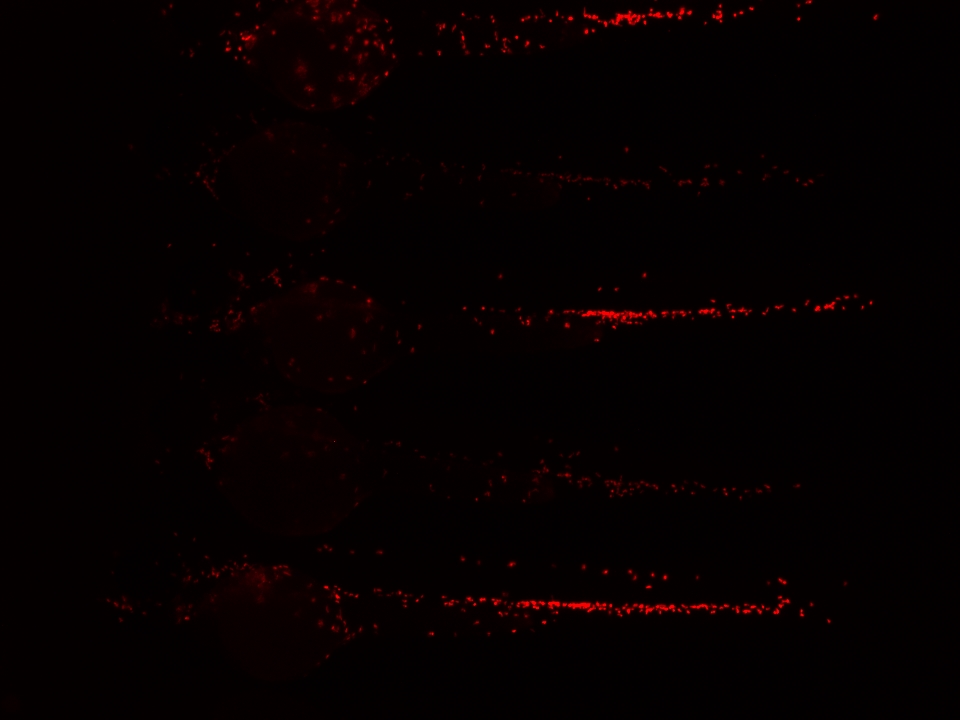

Supplement: Supplementary file 8 — Source data Fig. 4 [file 44321_2025_368_MOESM8_ESM.zip › FIGURE_4/4B/IMATINIB_1uM (7).jpg]

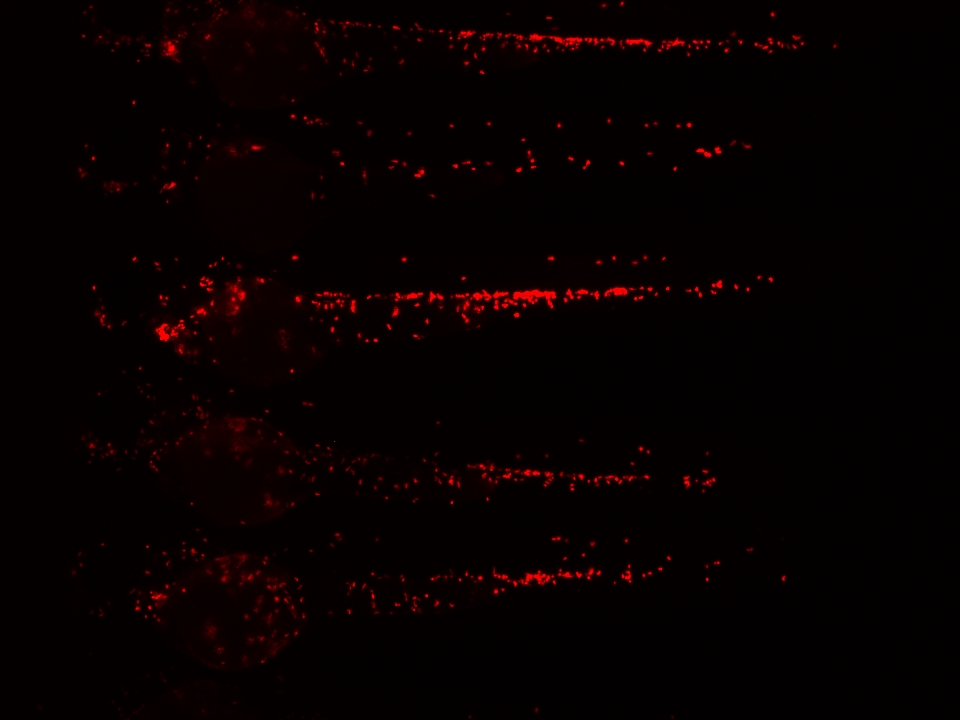

Supplement: Supplementary file 8 — Source data Fig. 4 [file 44321_2025_368_MOESM8_ESM.zip › FIGURE_4/4B/IMATINIB_1uM (8).jpg]

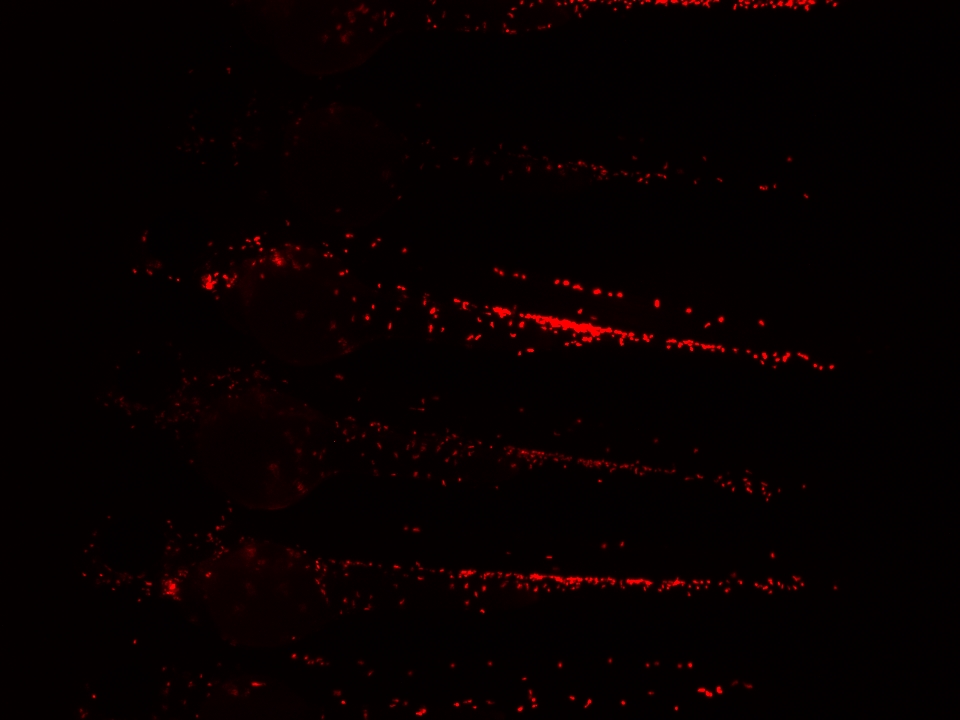

Supplement: Supplementary file 8 — Source data Fig. 4 [file 44321_2025_368_MOESM8_ESM.zip › FIGURE_4/4B/IMATINIB_1uM (9).jpg]

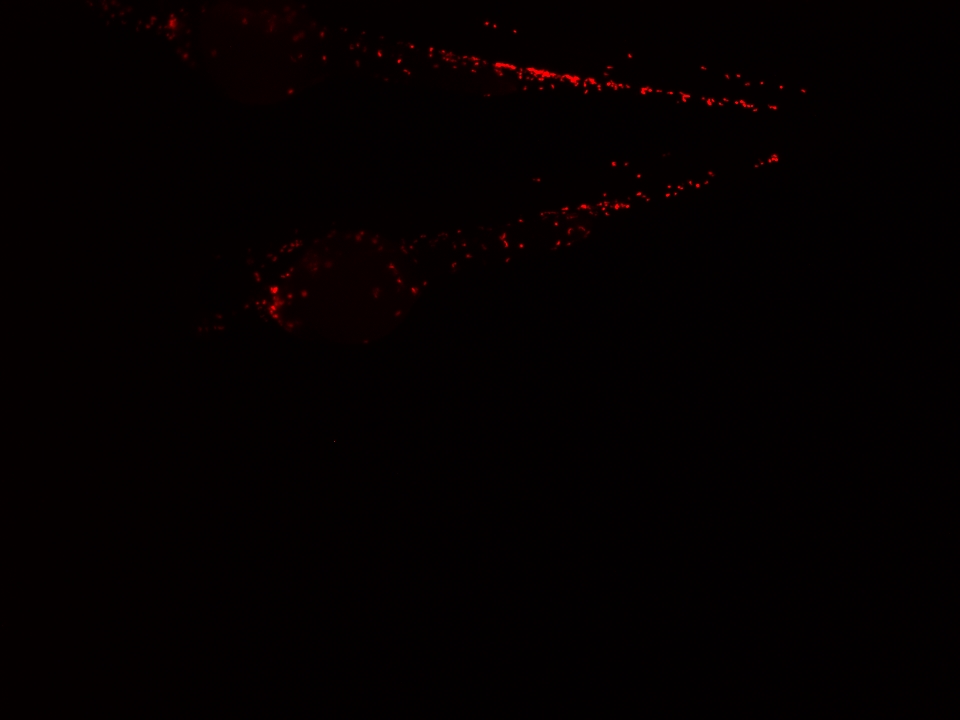

Supplement: Supplementary file 8 — Source data Fig. 4 [file 44321_2025_368_MOESM8_ESM.zip › FIGURE_4/4B/PONATINIB_01uM (1).jpg]

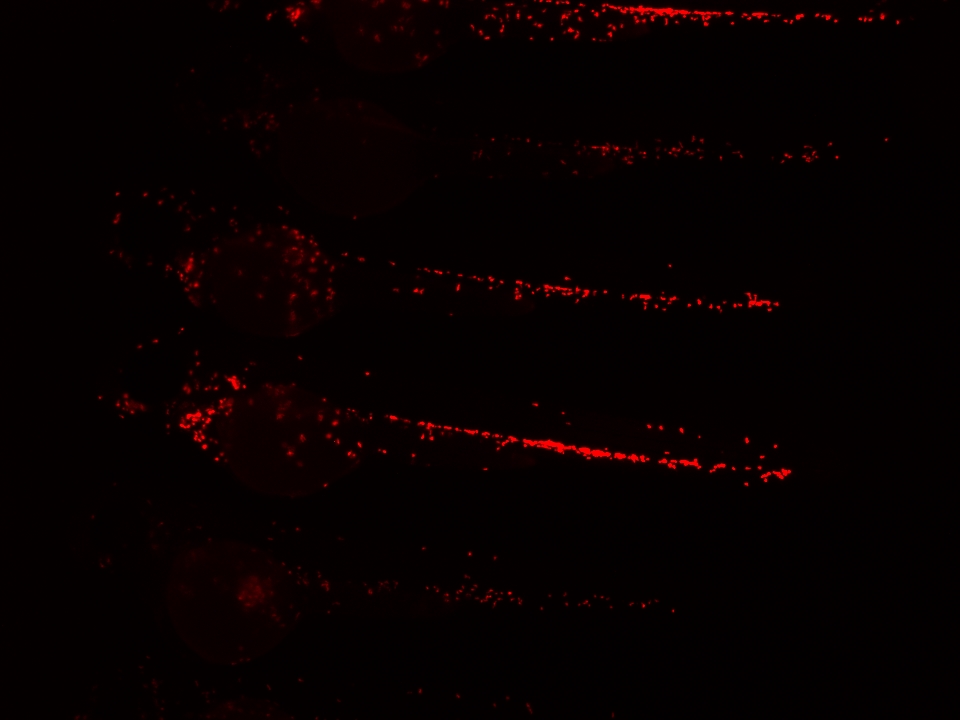

Supplement: Supplementary file 8 — Source data Fig. 4 [file 44321_2025_368_MOESM8_ESM.zip › FIGURE_4/4B/PONATINIB_01uM (10).jpg]

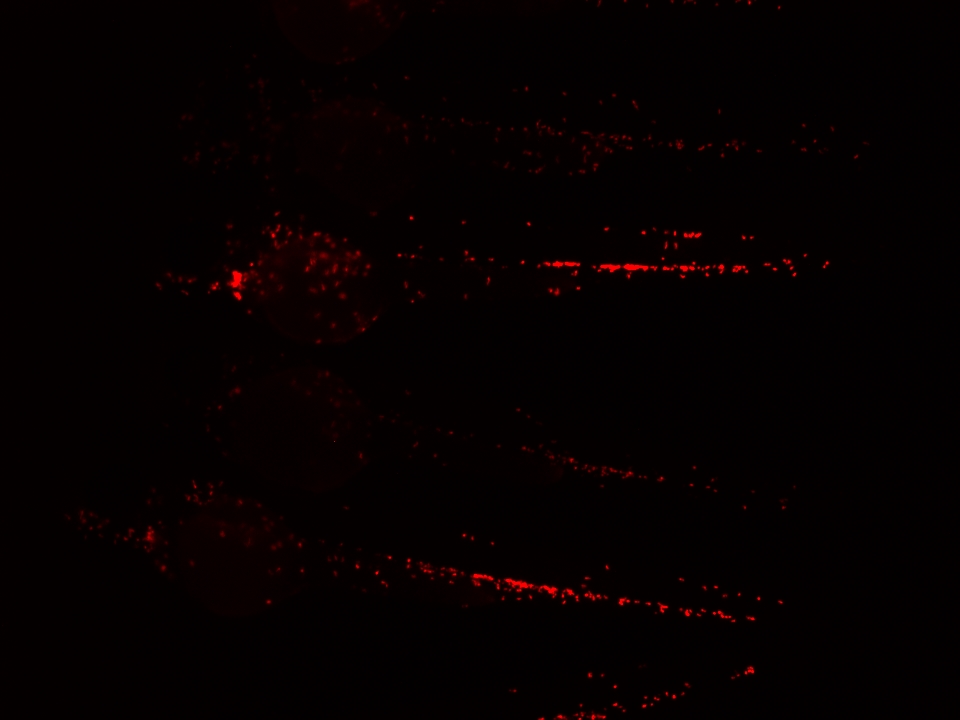

Supplement: Supplementary file 8 — Source data Fig. 4 [file 44321_2025_368_MOESM8_ESM.zip › FIGURE_4/4B/PONATINIB_01uM (11).jpg]

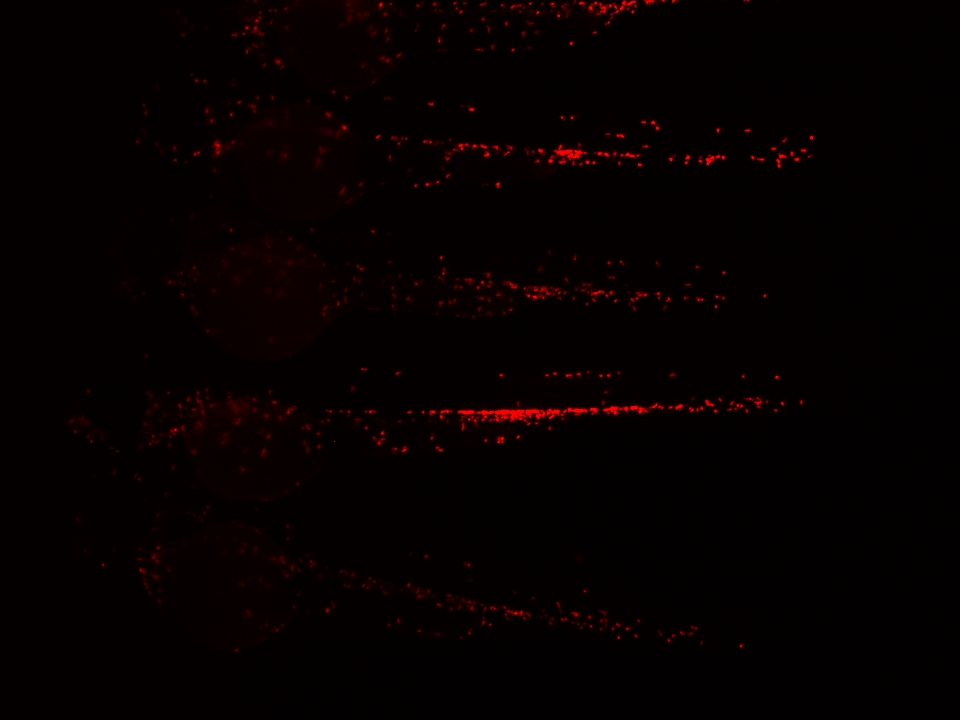

Supplement: Supplementary file 8 — Source data Fig. 4 [file 44321_2025_368_MOESM8_ESM.zip › FIGURE_4/4B/PONATINIB_01uM (2).jpg]

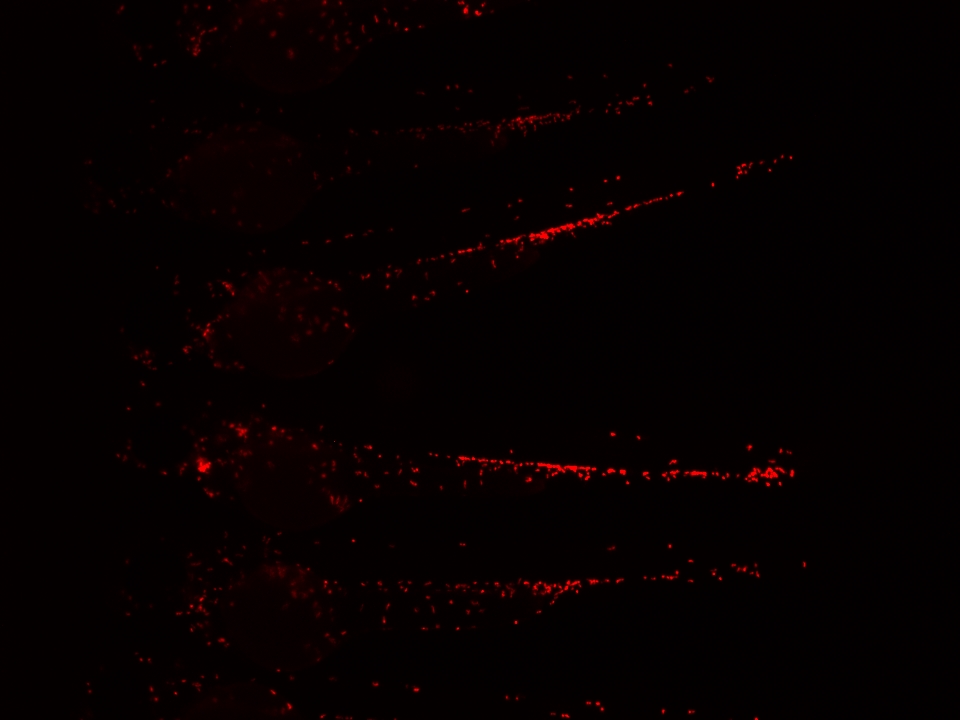

Supplement: Supplementary file 8 — Source data Fig. 4 [file 44321_2025_368_MOESM8_ESM.zip › FIGURE_4/4B/PONATINIB_01uM (3).jpg]

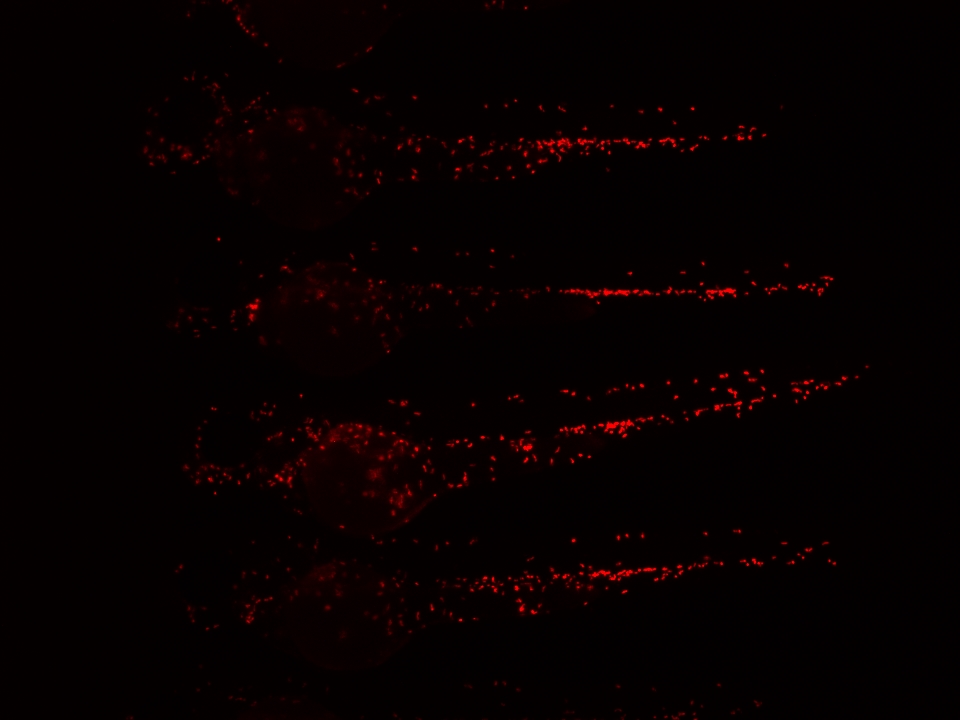

Supplement: Supplementary file 8 — Source data Fig. 4 [file 44321_2025_368_MOESM8_ESM.zip › FIGURE_4/4B/PONATINIB_01uM (4).jpg]

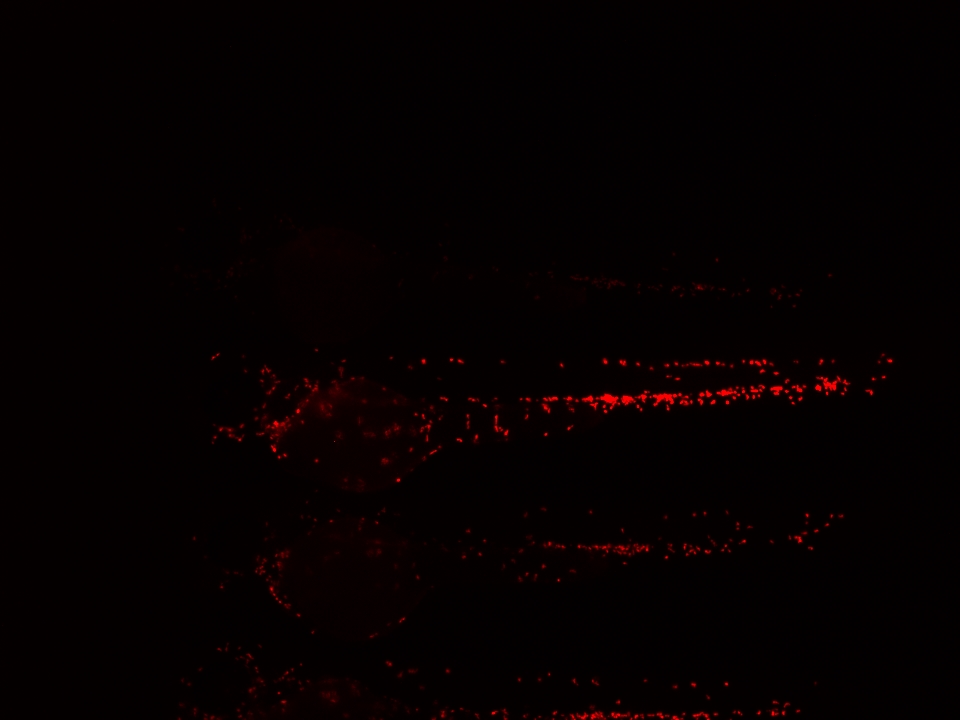

Supplement: Supplementary file 8 — Source data Fig. 4 [file 44321_2025_368_MOESM8_ESM.zip › FIGURE_4/4B/PONATINIB_01uM (5).jpg]

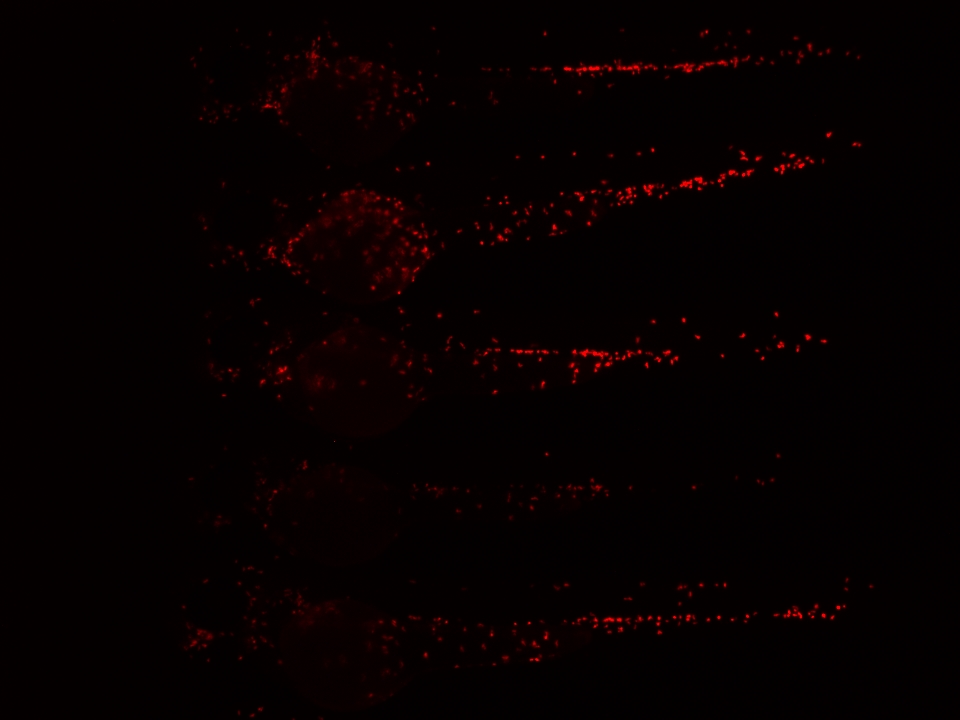

Supplement: Supplementary file 8 — Source data Fig. 4 [file 44321_2025_368_MOESM8_ESM.zip › FIGURE_4/4B/PONATINIB_01uM (6).jpg]

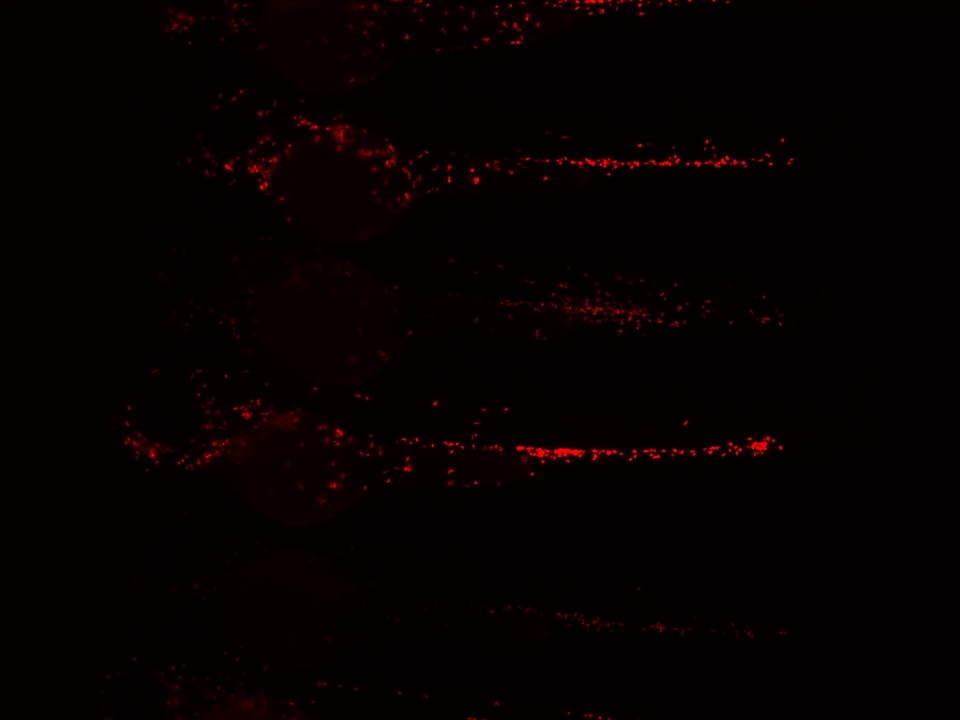

Supplement: Supplementary file 8 — Source data Fig. 4 [file 44321_2025_368_MOESM8_ESM.zip › FIGURE_4/4B/PONATINIB_01uM (7).jpg]

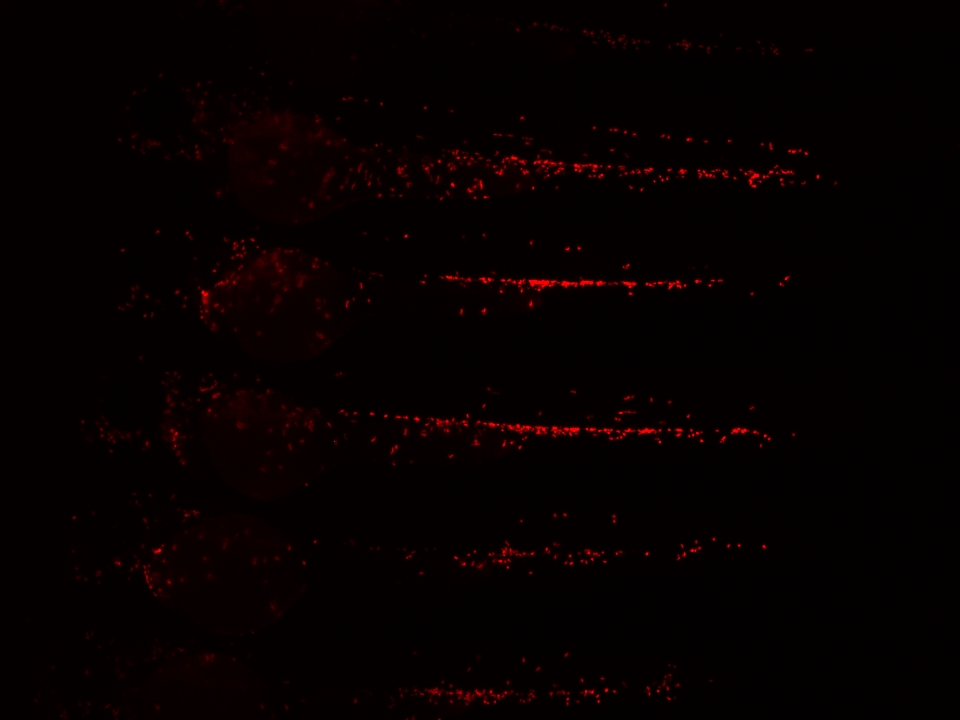

Supplement: Supplementary file 8 — Source data Fig. 4 [file 44321_2025_368_MOESM8_ESM.zip › FIGURE_4/4B/PONATINIB_01uM (8).jpg]

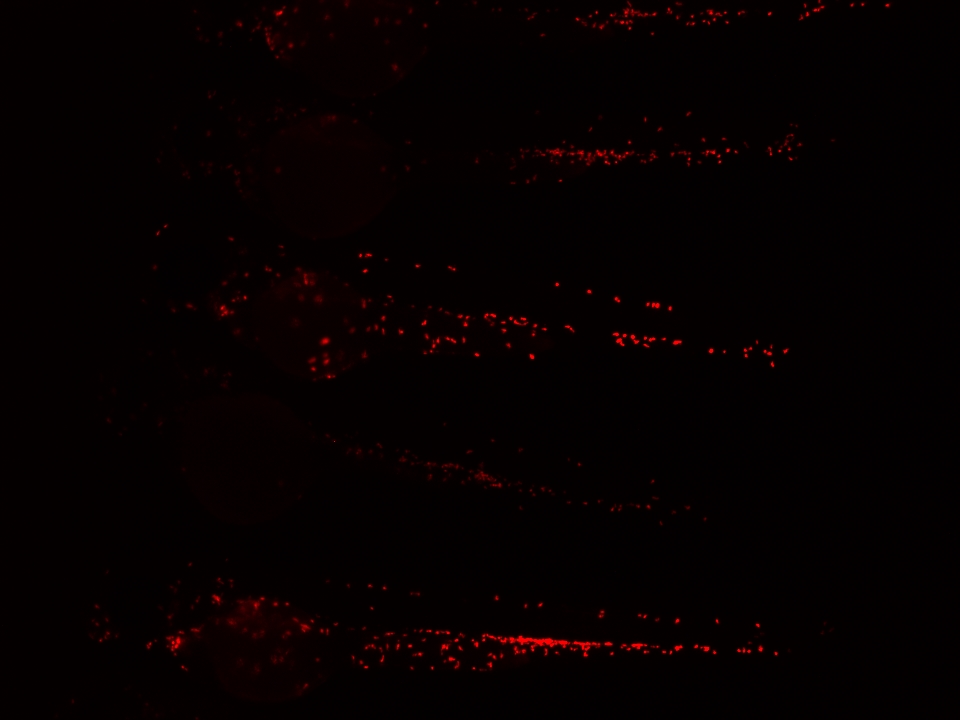

Supplement: Supplementary file 8 — Source data Fig. 4 [file 44321_2025_368_MOESM8_ESM.zip › FIGURE_4/4B/PONATINIB_01uM (9).jpg]

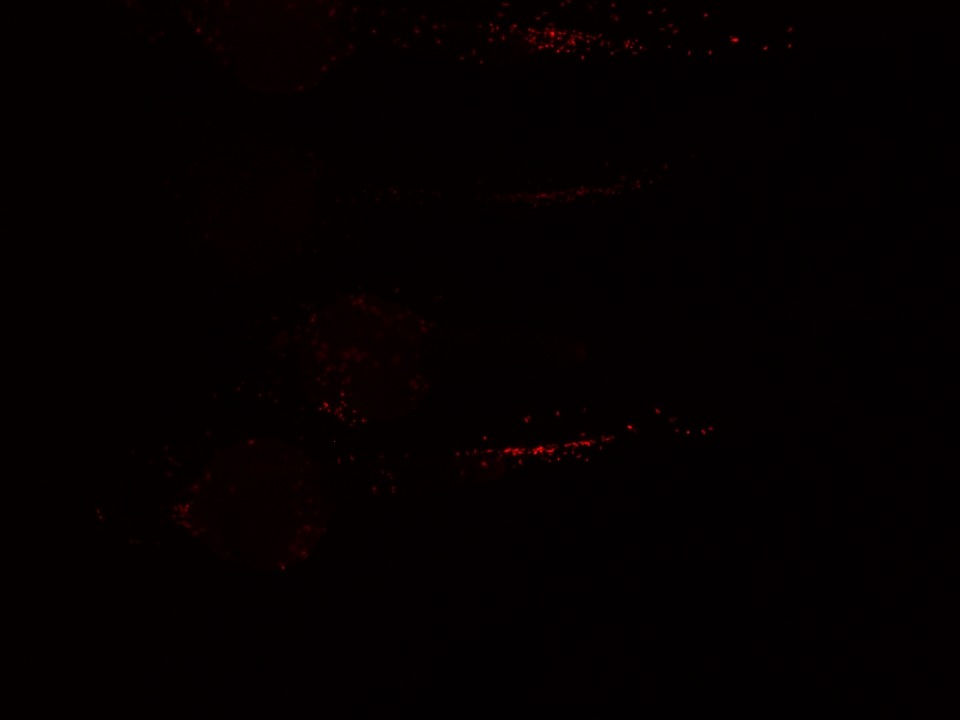

Supplement: Supplementary file 8 — Source data Fig. 4 [file 44321_2025_368_MOESM8_ESM.zip › FIGURE_4/4B/PONATINIB_1uM (1).jpg]

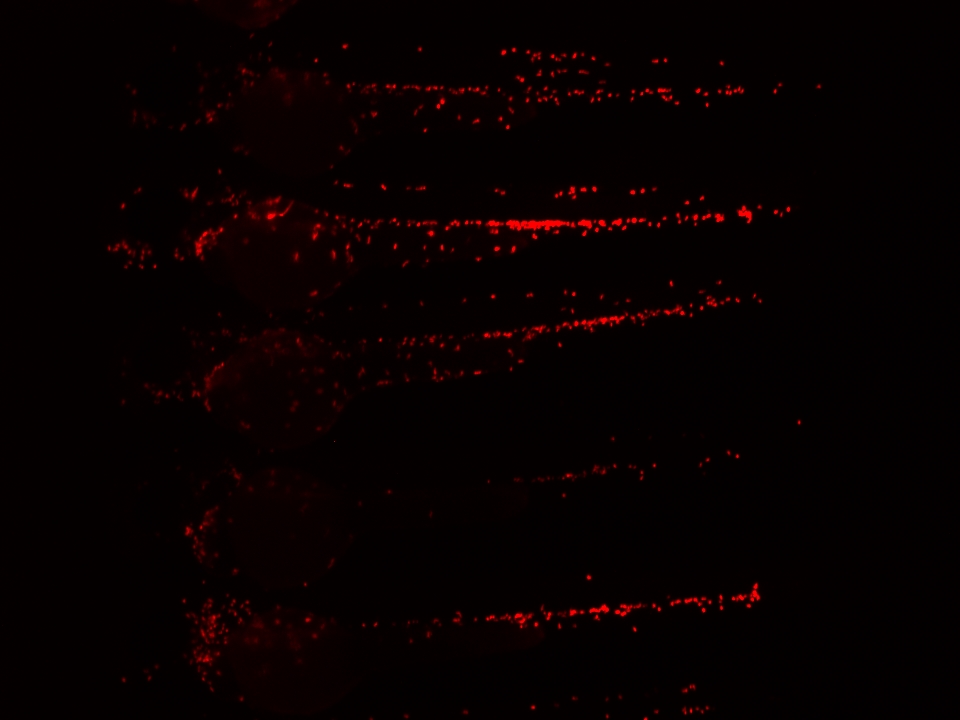

Supplement: Supplementary file 8 — Source data Fig. 4 [file 44321_2025_368_MOESM8_ESM.zip › FIGURE_4/4B/PONATINIB_1uM (10).jpg]
