# Supplementary figures and images for: TKI-mediated inhibition of NLRP1 inflammasome restores erythropoiesis in DBA syndrome (part 1 of 4)
Source: EMBO Mol Med. 2026 Jan 9;18(2):702–24. doi: 10.1038/s44321-025-00368-3 (PMC12905221; doi:10.1038/s44321-025-00368-3)

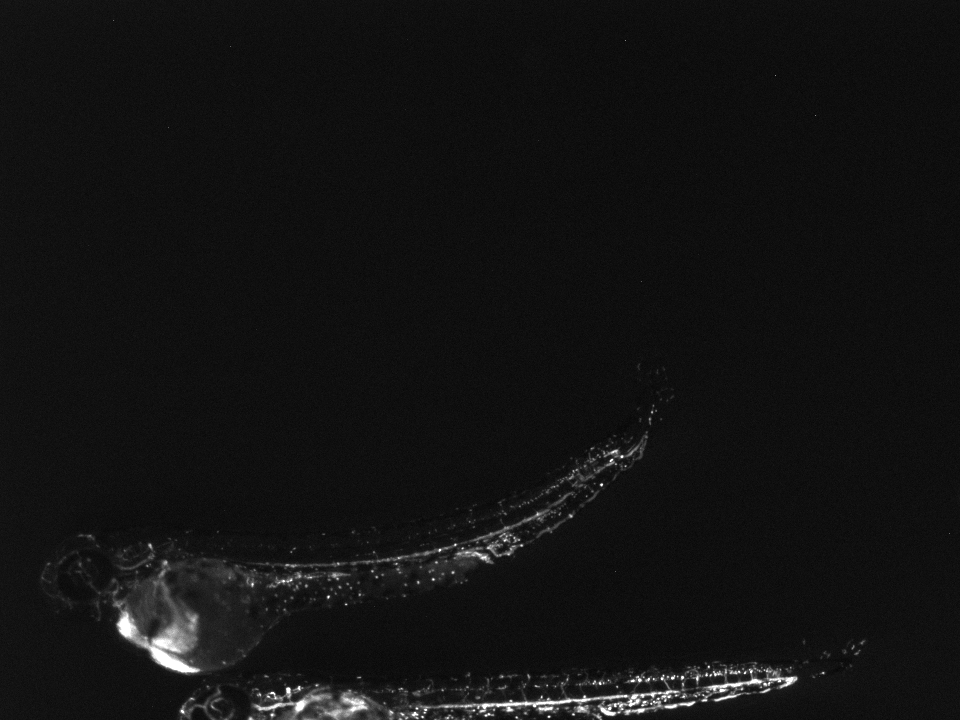

Supplement: Supplementary file 7 — Source data Fig. 3 [file 44321_2025_368_MOESM7_ESM.zip › FIGURE_3/3D/DASATINIB_01uM (1).tif]

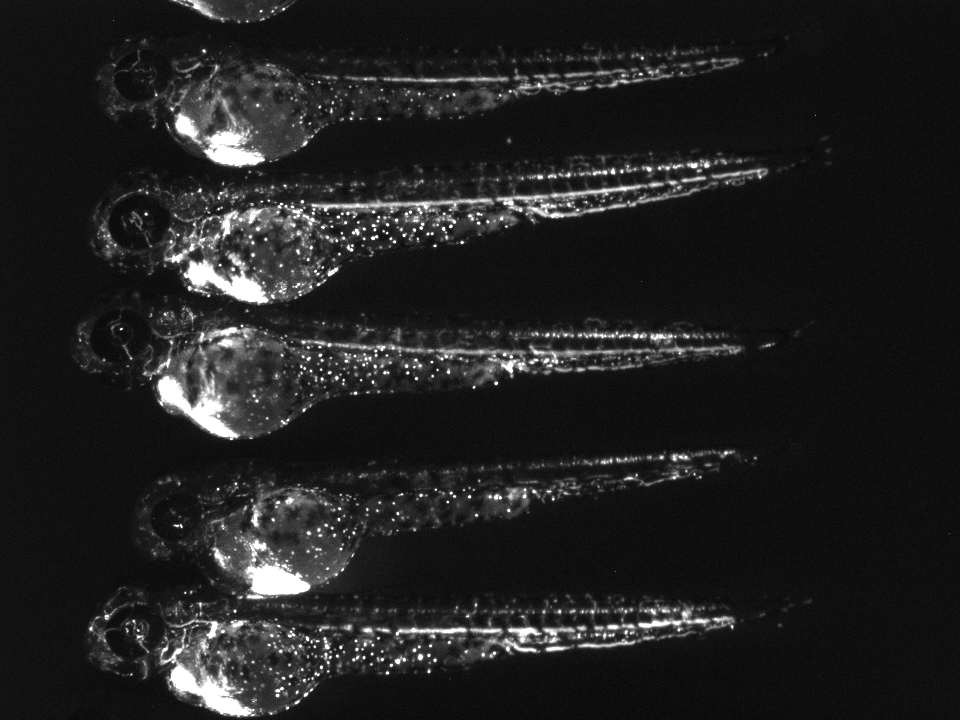

Supplement: Supplementary file 7 — Source data Fig. 3 [file 44321_2025_368_MOESM7_ESM.zip › FIGURE_3/3D/DASATINIB_01uM (10).tif]

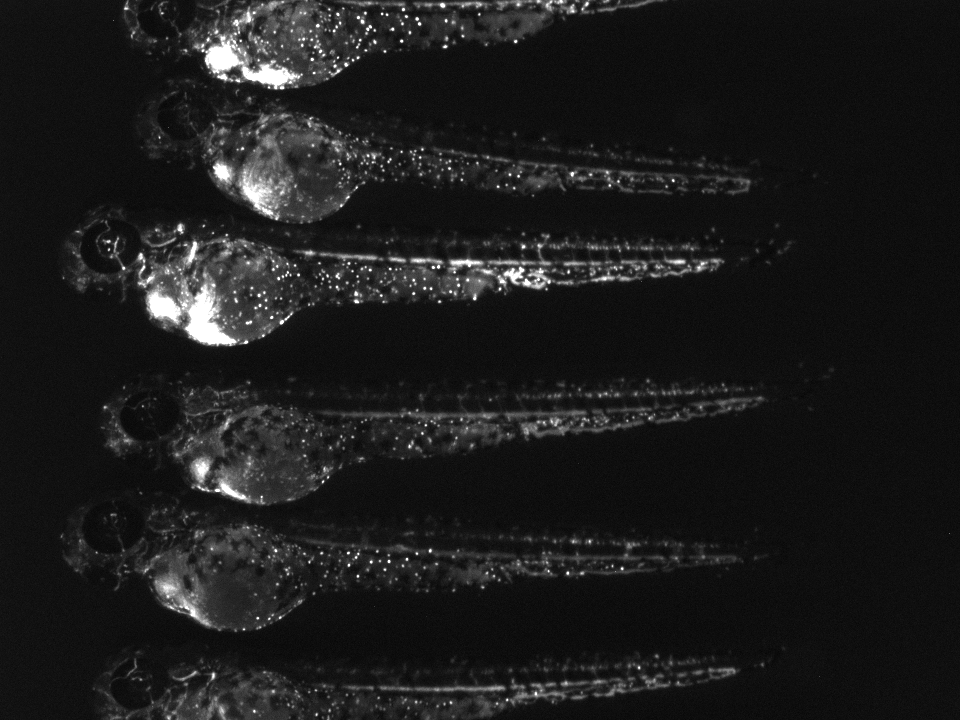

Supplement: Supplementary file 7 — Source data Fig. 3 [file 44321_2025_368_MOESM7_ESM.zip › FIGURE_3/3D/DASATINIB_01uM (12).tif]

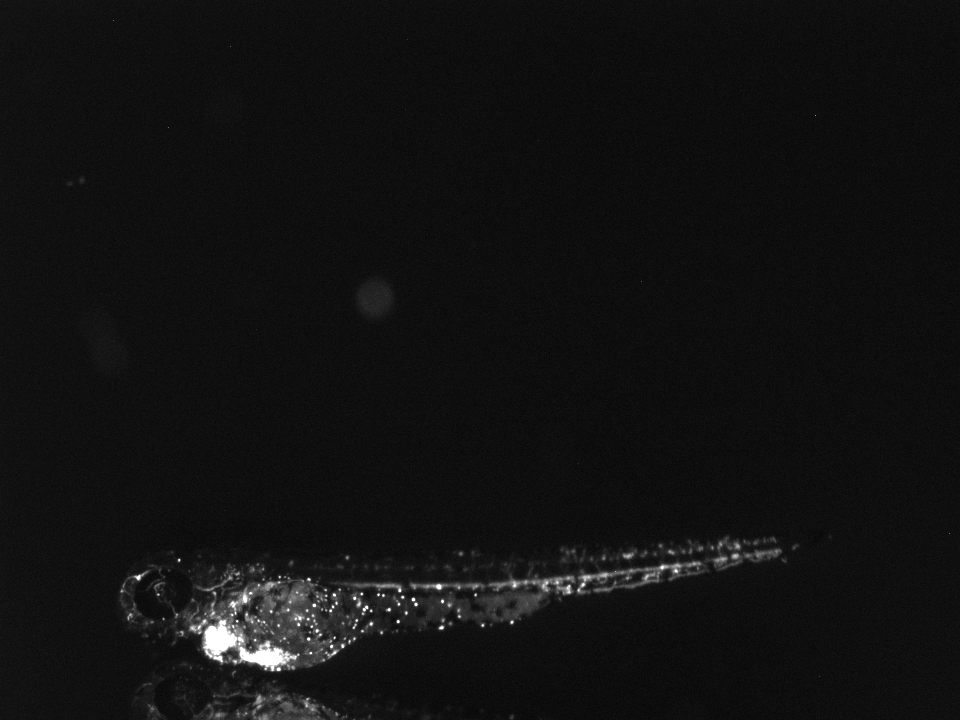

Supplement: Supplementary file 7 — Source data Fig. 3 [file 44321_2025_368_MOESM7_ESM.zip › FIGURE_3/3D/DASATINIB_01uM (13).tif]

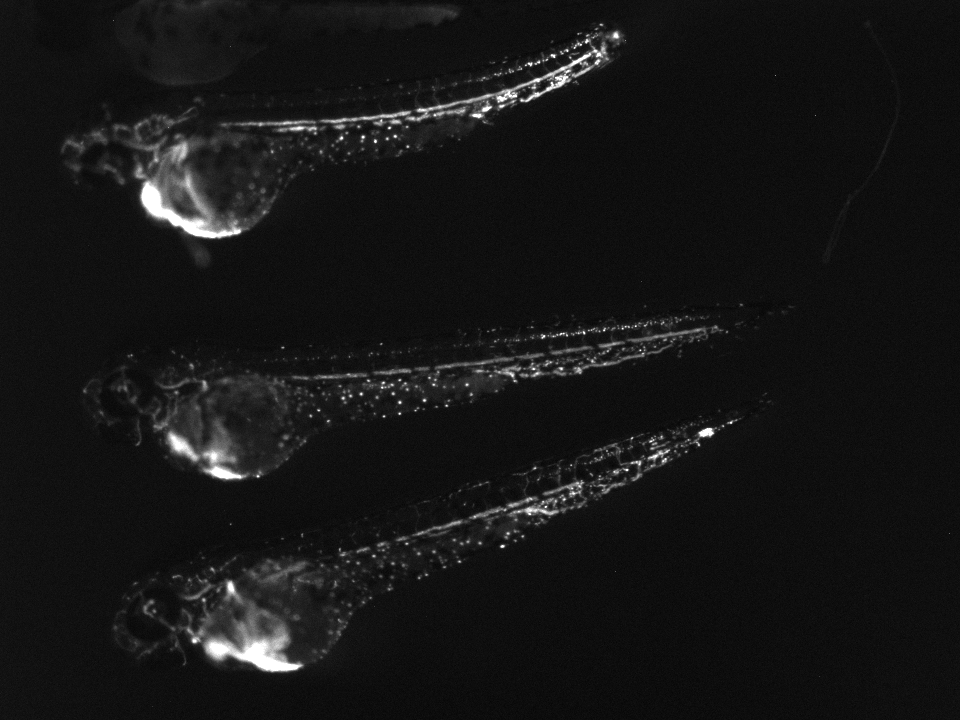

Supplement: Supplementary file 7 — Source data Fig. 3 [file 44321_2025_368_MOESM7_ESM.zip › FIGURE_3/3D/DASATINIB_01uM (14).tif]

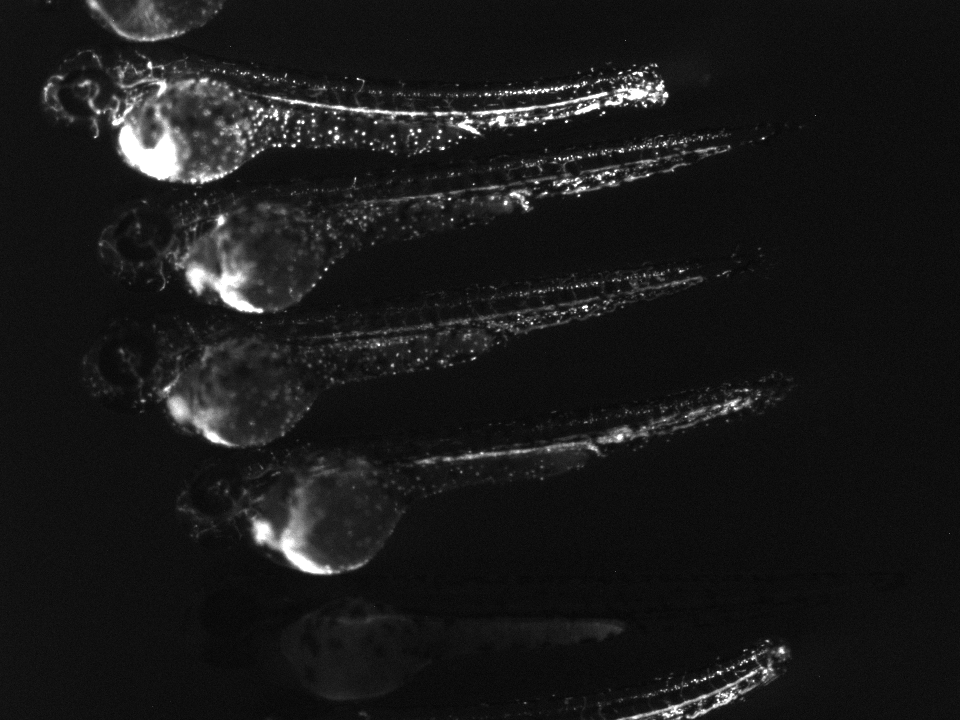

Supplement: Supplementary file 7 — Source data Fig. 3 [file 44321_2025_368_MOESM7_ESM.zip › FIGURE_3/3D/DASATINIB_01uM (15).tif]

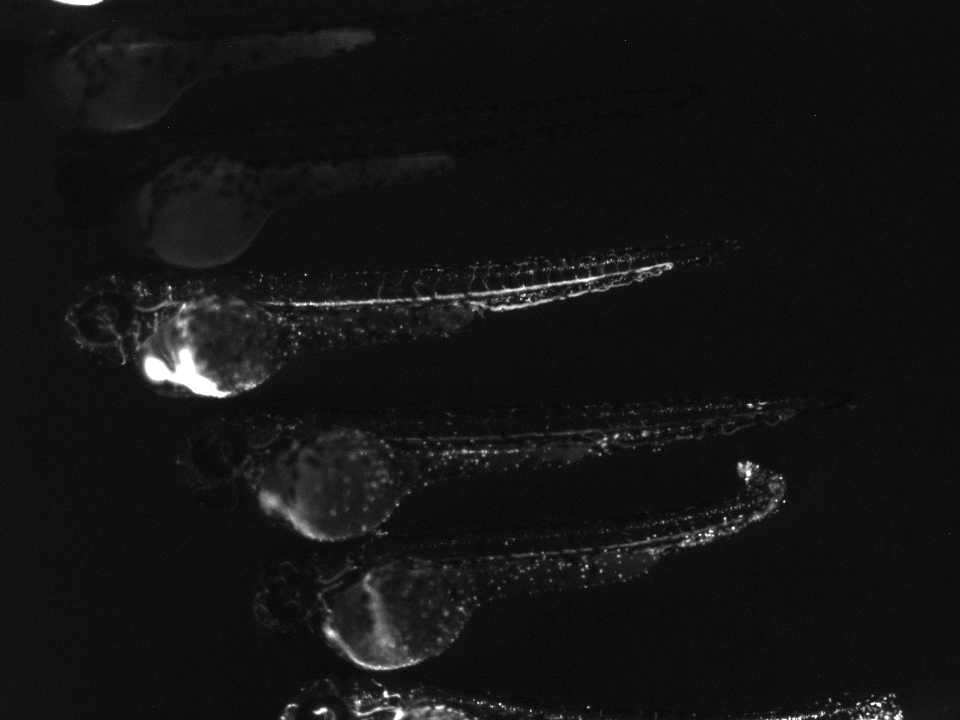

Supplement: Supplementary file 7 — Source data Fig. 3 [file 44321_2025_368_MOESM7_ESM.zip › FIGURE_3/3D/DASATINIB_01uM (16).tif]

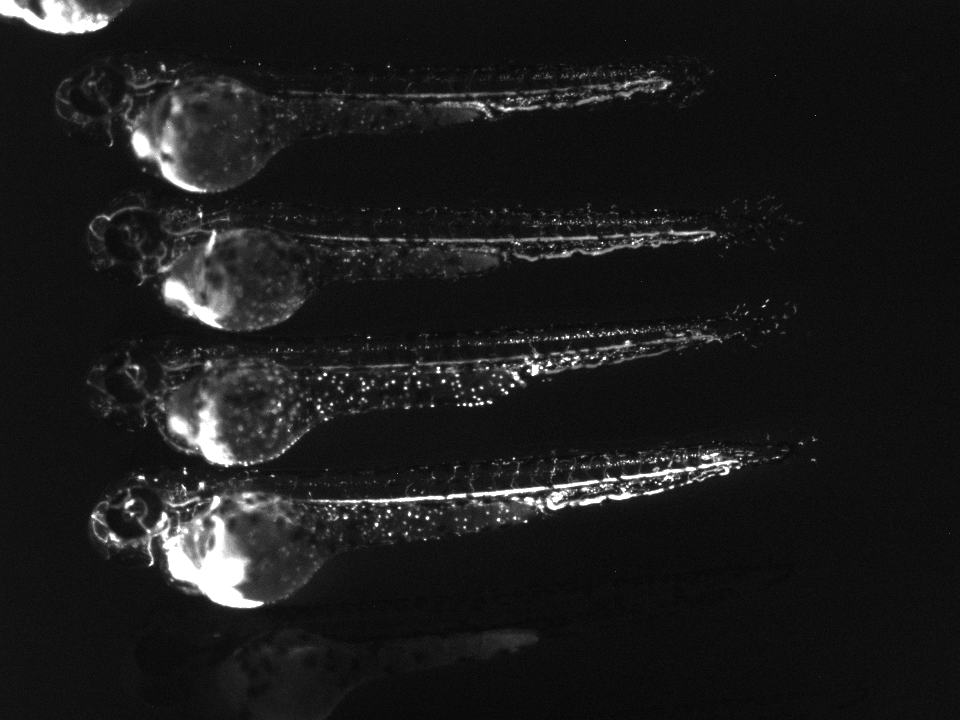

Supplement: Supplementary file 7 — Source data Fig. 3 [file 44321_2025_368_MOESM7_ESM.zip › FIGURE_3/3D/DASATINIB_01uM (17).tif]

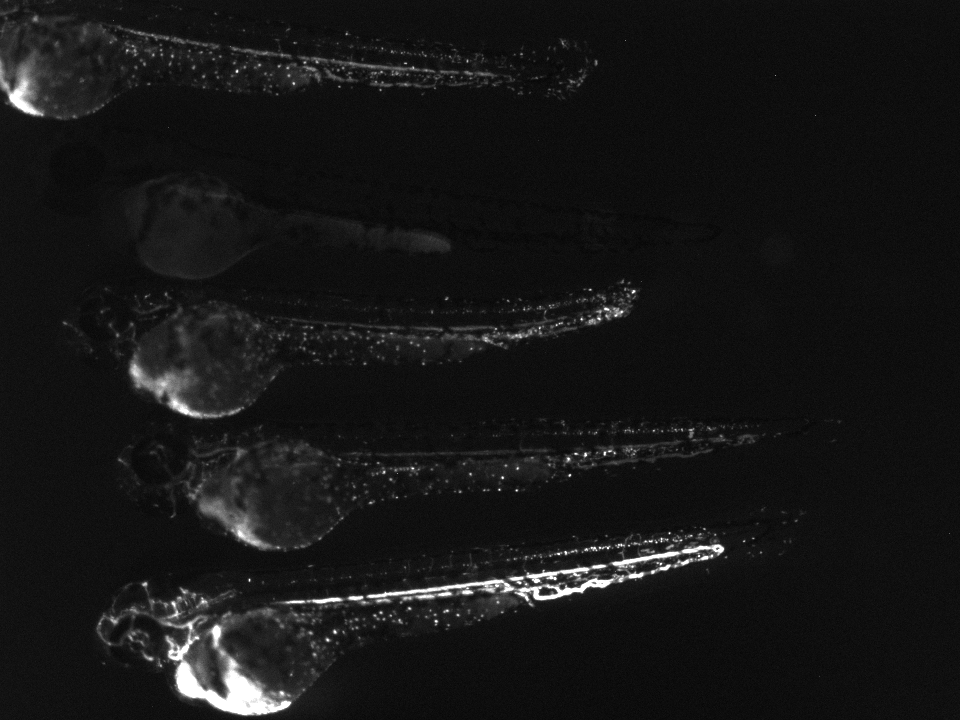

Supplement: Supplementary file 7 — Source data Fig. 3 [file 44321_2025_368_MOESM7_ESM.zip › FIGURE_3/3D/DASATINIB_01uM (18).tif]

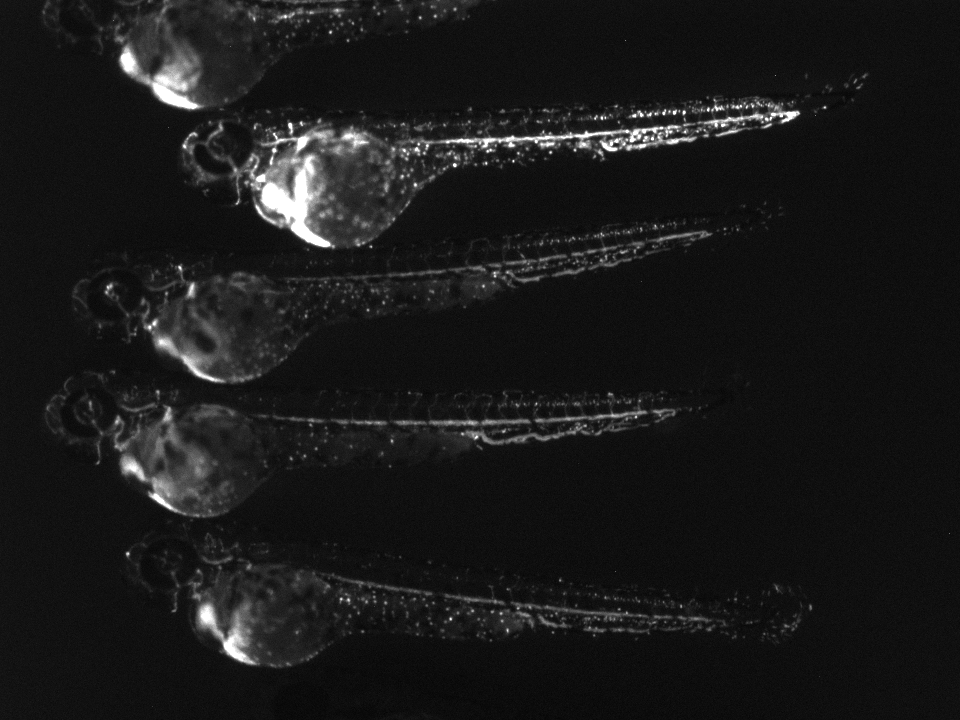

Supplement: Supplementary file 7 — Source data Fig. 3 [file 44321_2025_368_MOESM7_ESM.zip › FIGURE_3/3D/DASATINIB_01uM (2).tif]

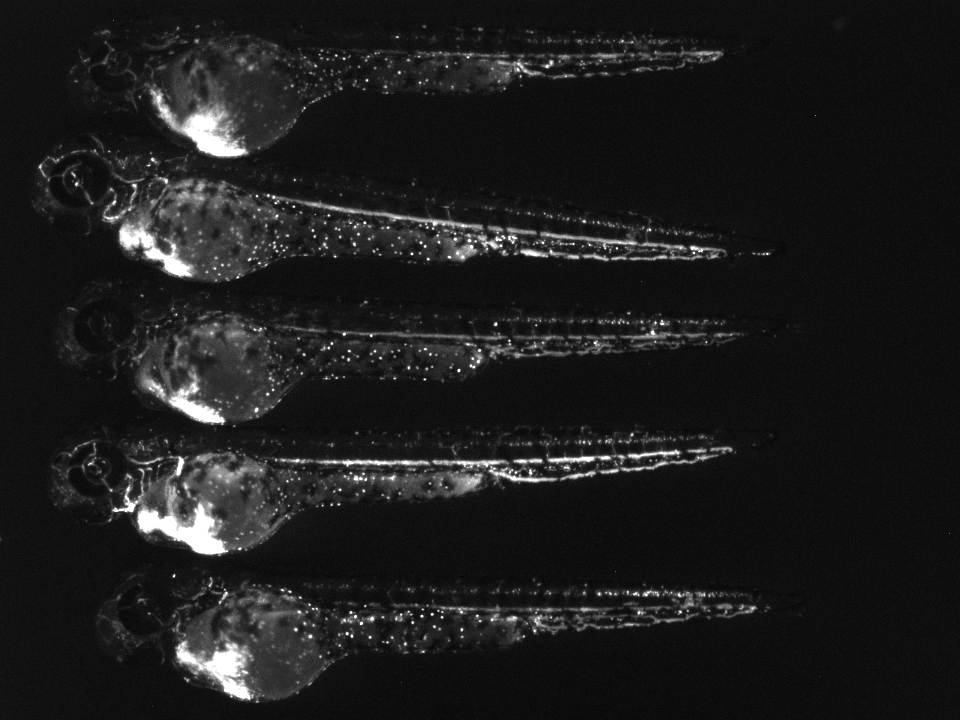

Supplement: Supplementary file 7 — Source data Fig. 3 [file 44321_2025_368_MOESM7_ESM.zip › FIGURE_3/3D/DASATINIB_01uM (4).tif]

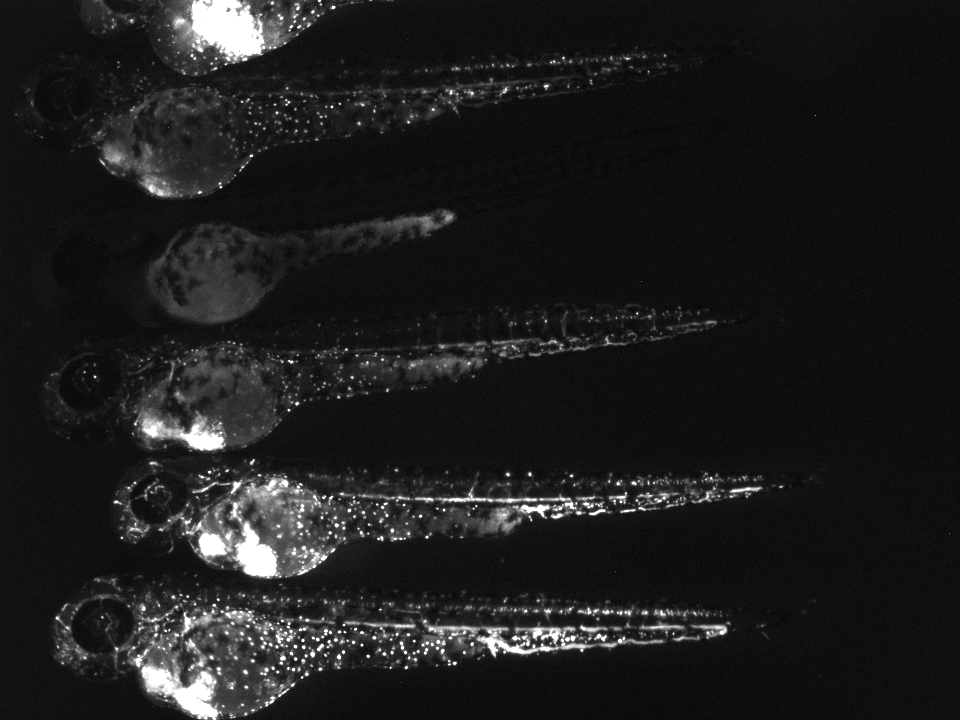

Supplement: Supplementary file 7 — Source data Fig. 3 [file 44321_2025_368_MOESM7_ESM.zip › FIGURE_3/3D/DASATINIB_01uM (6).tif]

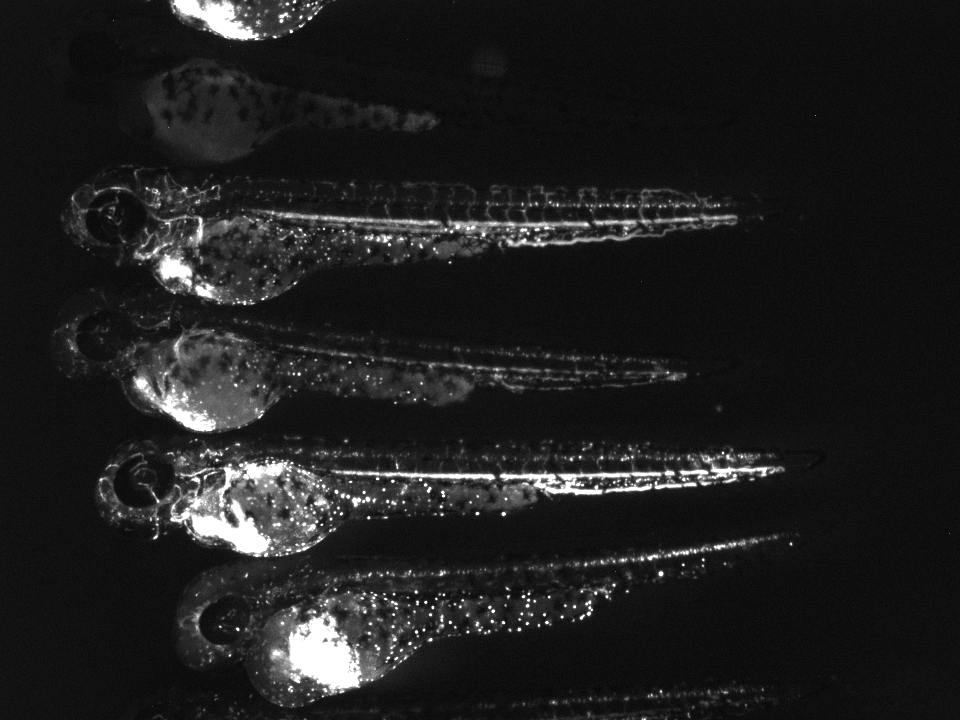

Supplement: Supplementary file 7 — Source data Fig. 3 [file 44321_2025_368_MOESM7_ESM.zip › FIGURE_3/3D/DASATINIB_01uM (8).tif]

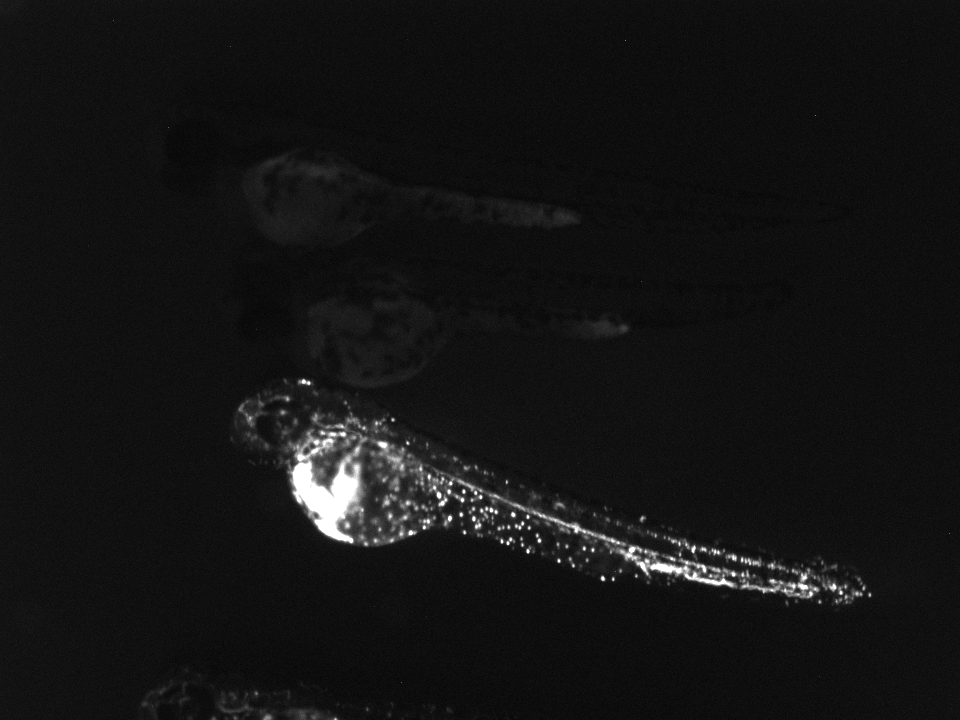

Supplement: Supplementary file 7 — Source data Fig. 3 [file 44321_2025_368_MOESM7_ESM.zip › FIGURE_3/3D/DASATINIB_1uM (1).tif]

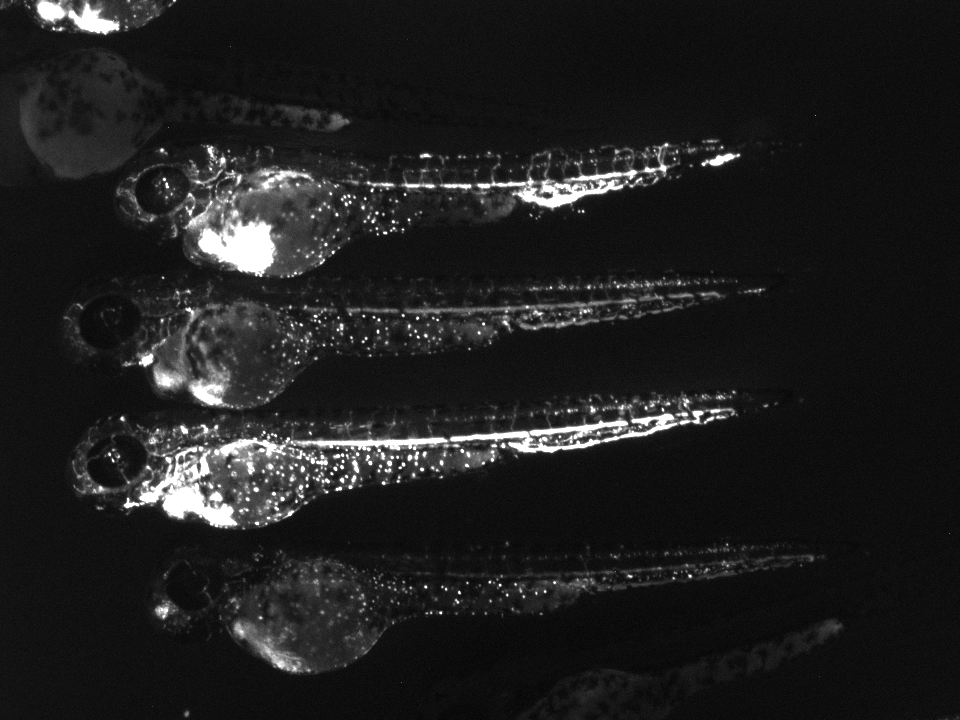

Supplement: Supplementary file 7 — Source data Fig. 3 [file 44321_2025_368_MOESM7_ESM.zip › FIGURE_3/3D/DASATINIB_1uM (10).tif]

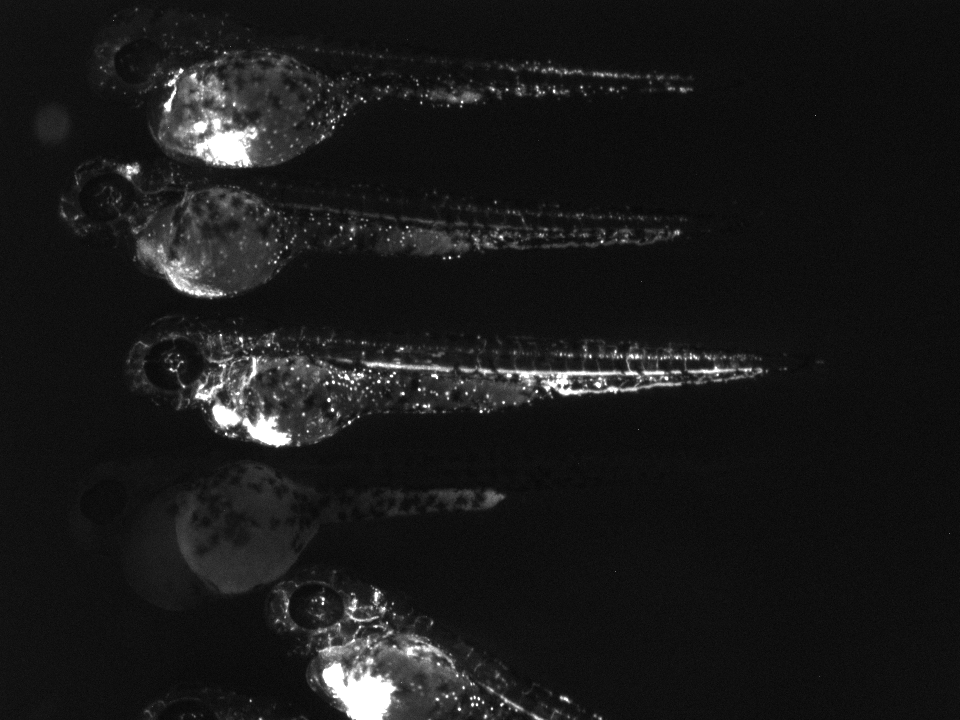

Supplement: Supplementary file 7 — Source data Fig. 3 [file 44321_2025_368_MOESM7_ESM.zip › FIGURE_3/3D/DASATINIB_1uM (12).tif]

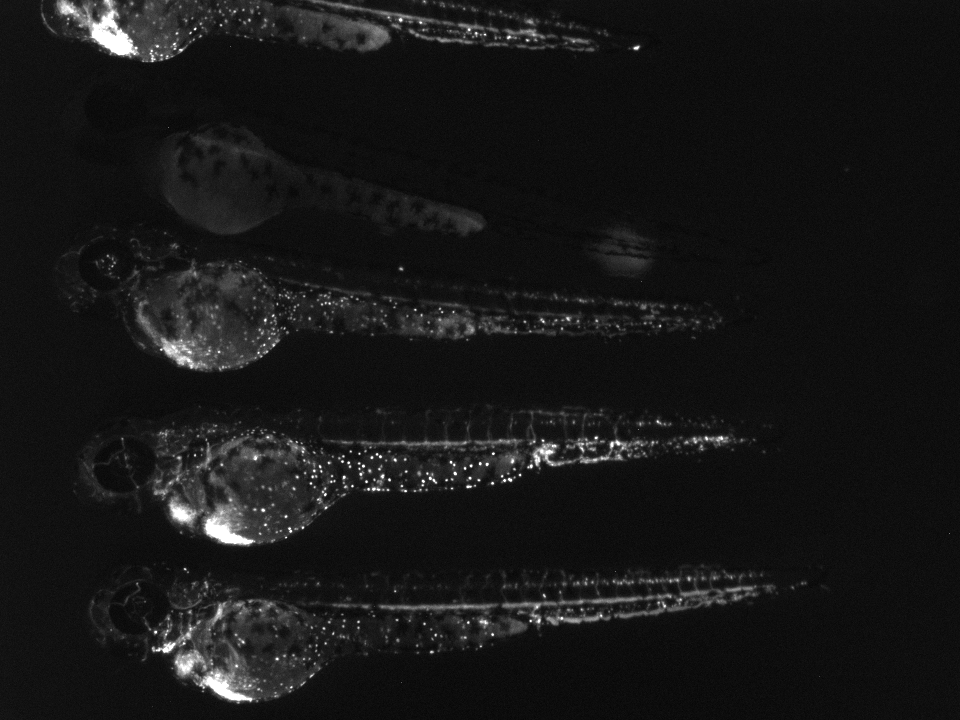

Supplement: Supplementary file 7 — Source data Fig. 3 [file 44321_2025_368_MOESM7_ESM.zip › FIGURE_3/3D/DASATINIB_1uM (14).tif]

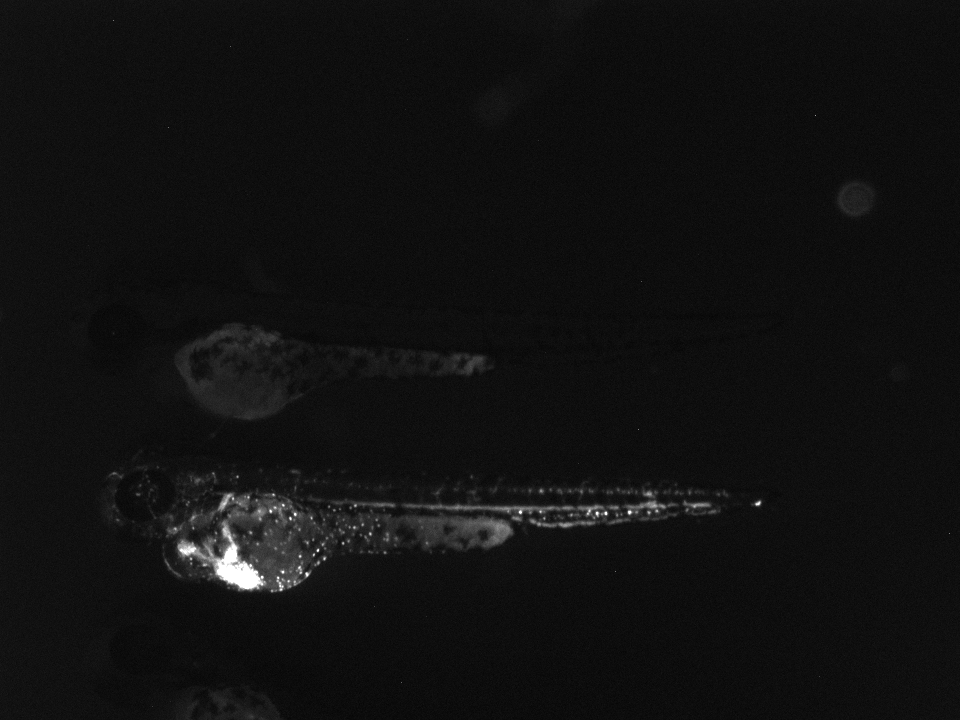

Supplement: Supplementary file 7 — Source data Fig. 3 [file 44321_2025_368_MOESM7_ESM.zip › FIGURE_3/3D/DASATINIB_1uM (15).tif]

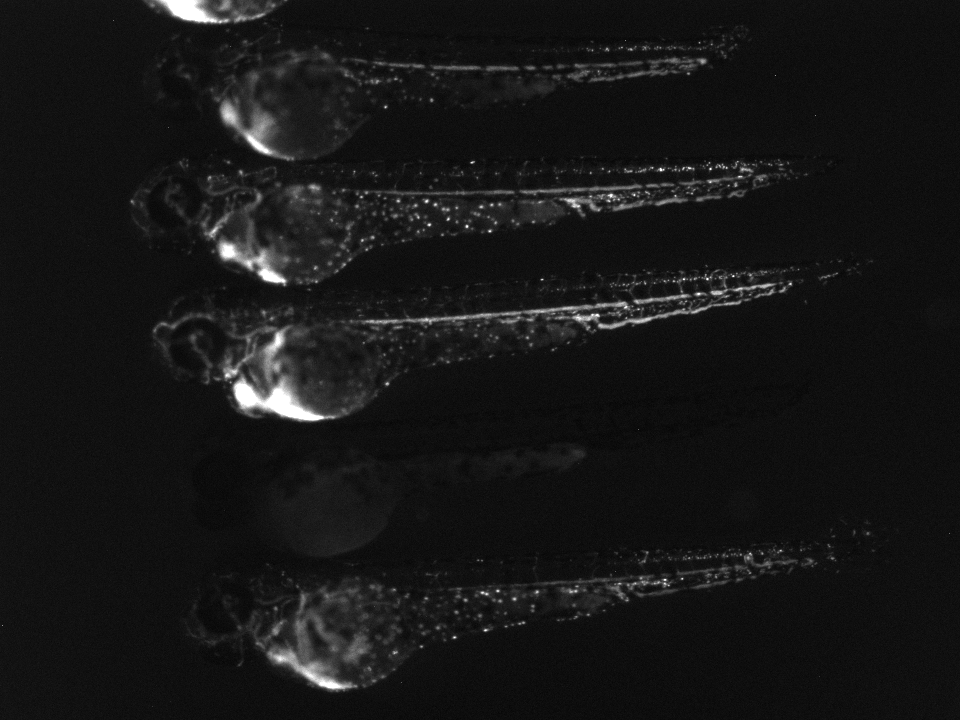

Supplement: Supplementary file 7 — Source data Fig. 3 [file 44321_2025_368_MOESM7_ESM.zip › FIGURE_3/3D/DASATINIB_1uM (16).tif]

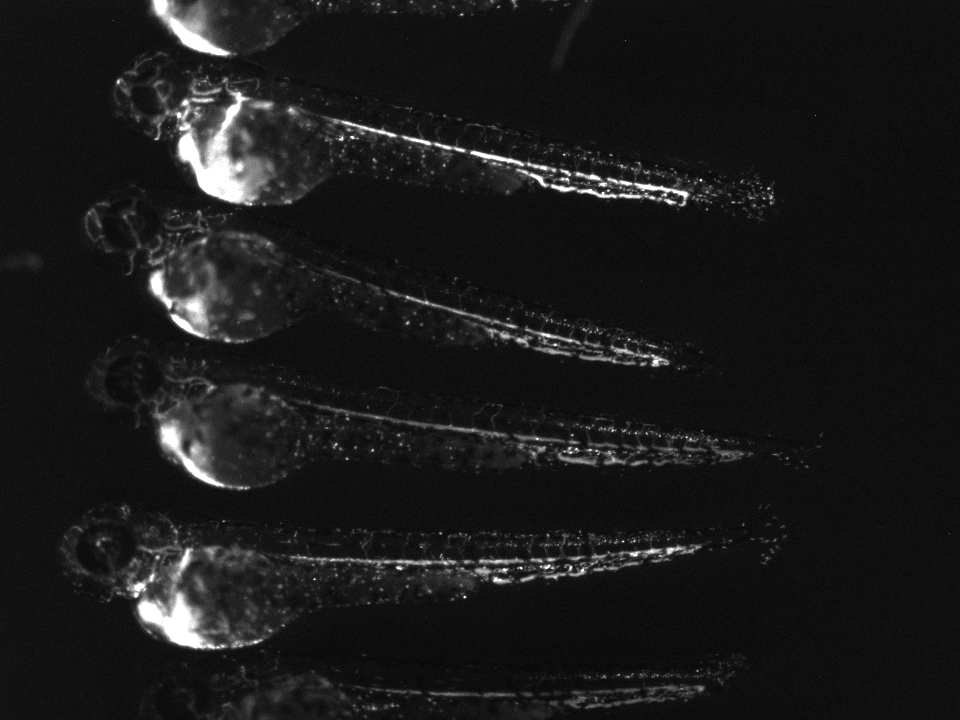

Supplement: Supplementary file 7 — Source data Fig. 3 [file 44321_2025_368_MOESM7_ESM.zip › FIGURE_3/3D/DASATINIB_1uM (17).tif]

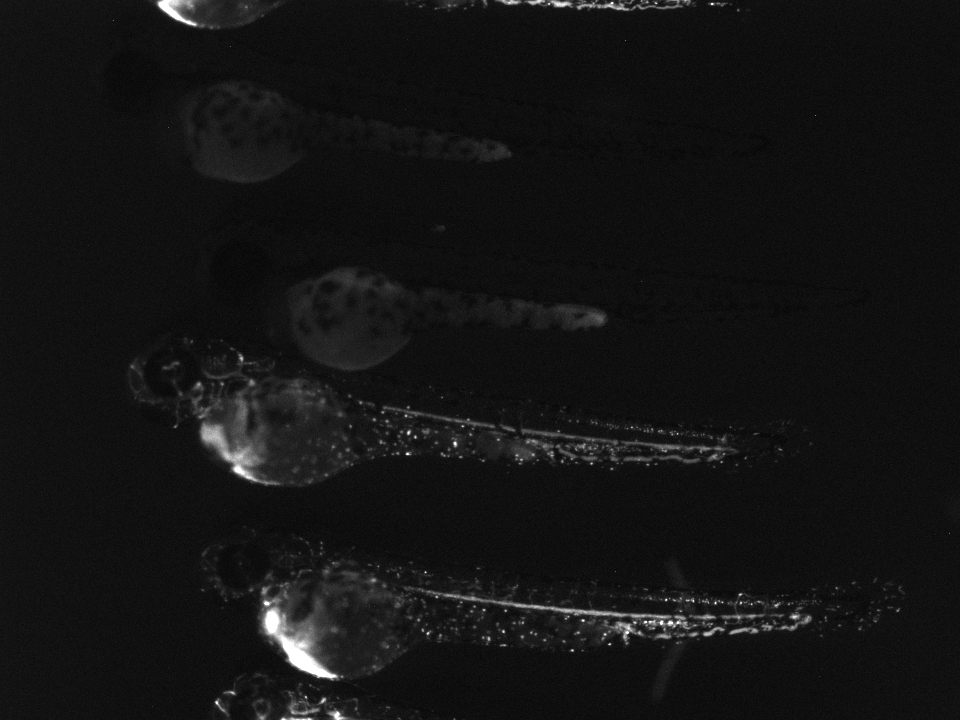

Supplement: Supplementary file 7 — Source data Fig. 3 [file 44321_2025_368_MOESM7_ESM.zip › FIGURE_3/3D/DASATINIB_1uM (18).tif]

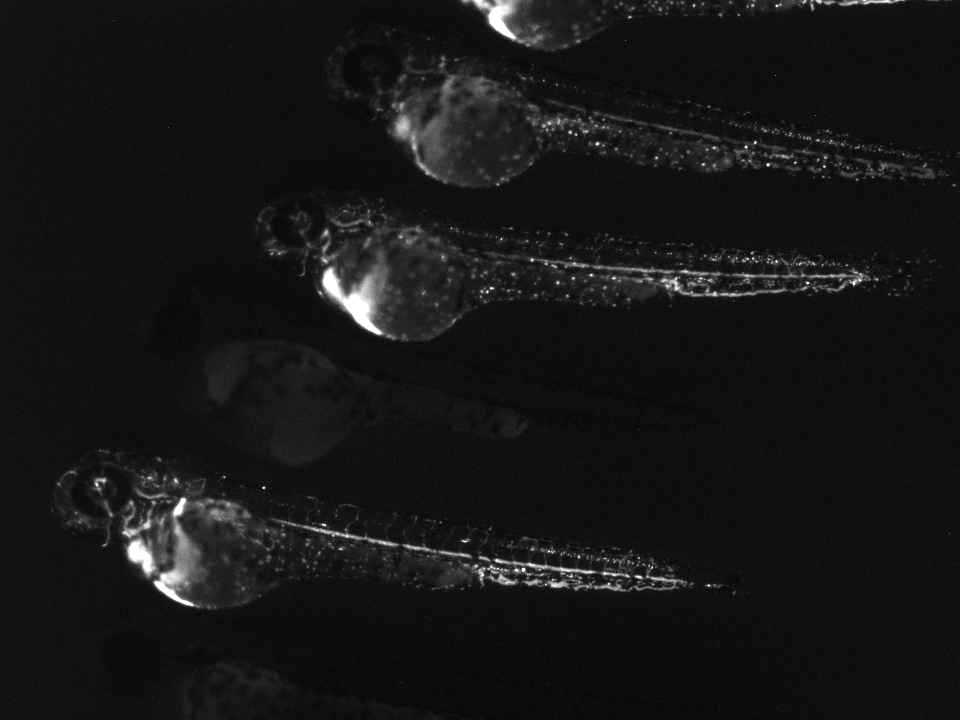

Supplement: Supplementary file 7 — Source data Fig. 3 [file 44321_2025_368_MOESM7_ESM.zip › FIGURE_3/3D/DASATINIB_1uM (19).tif]

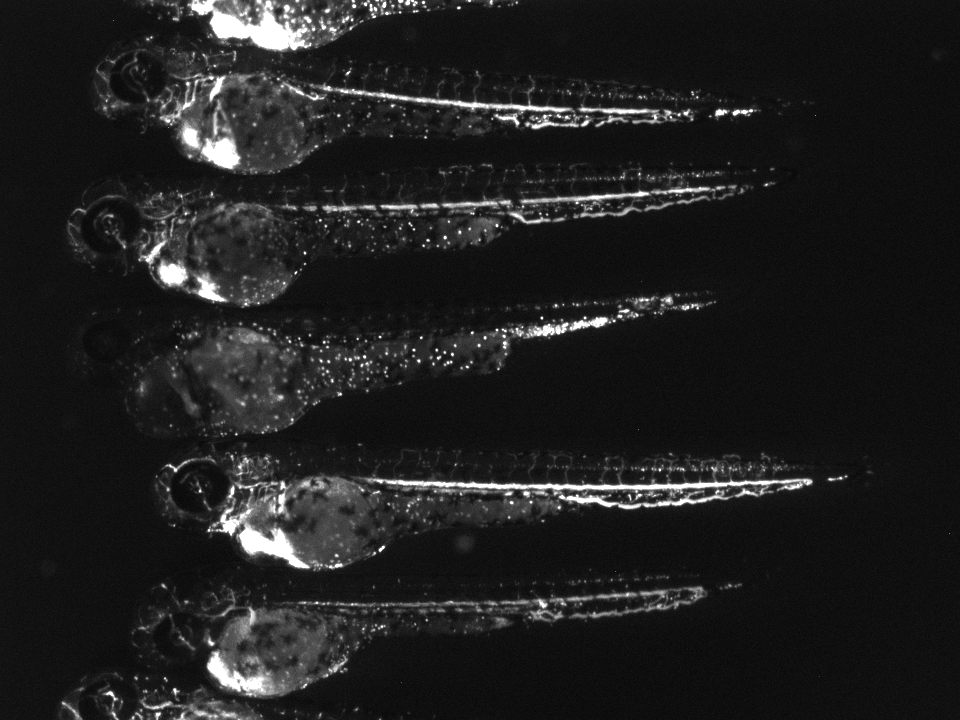

Supplement: Supplementary file 7 — Source data Fig. 3 [file 44321_2025_368_MOESM7_ESM.zip › FIGURE_3/3D/DASATINIB_1uM (2).tif]

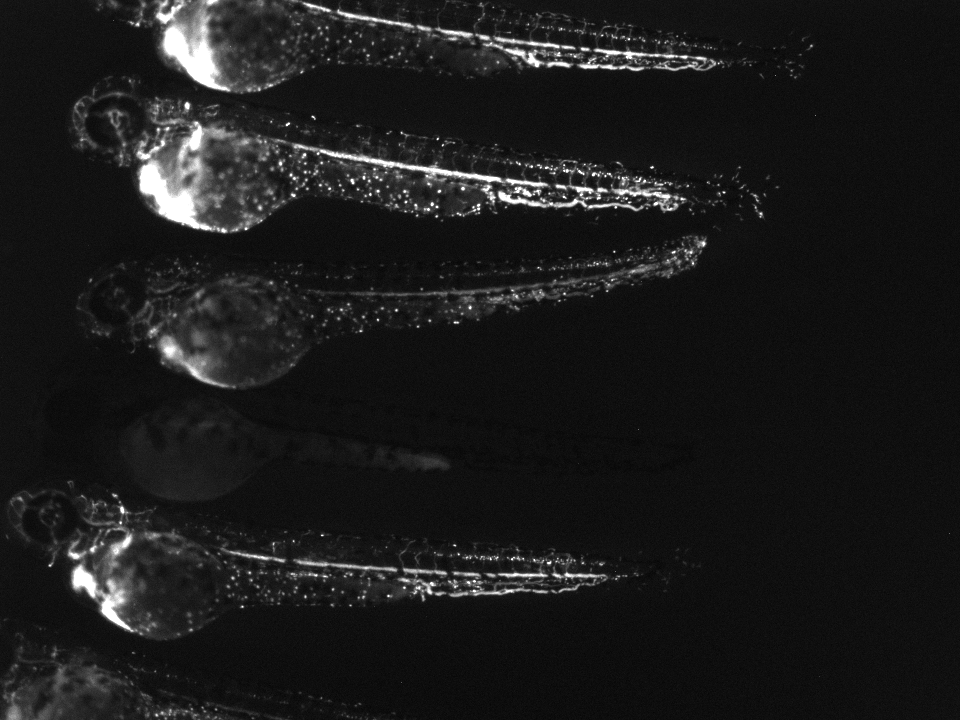

Supplement: Supplementary file 7 — Source data Fig. 3 [file 44321_2025_368_MOESM7_ESM.zip › FIGURE_3/3D/DASATINIB_1uM (20).tif]

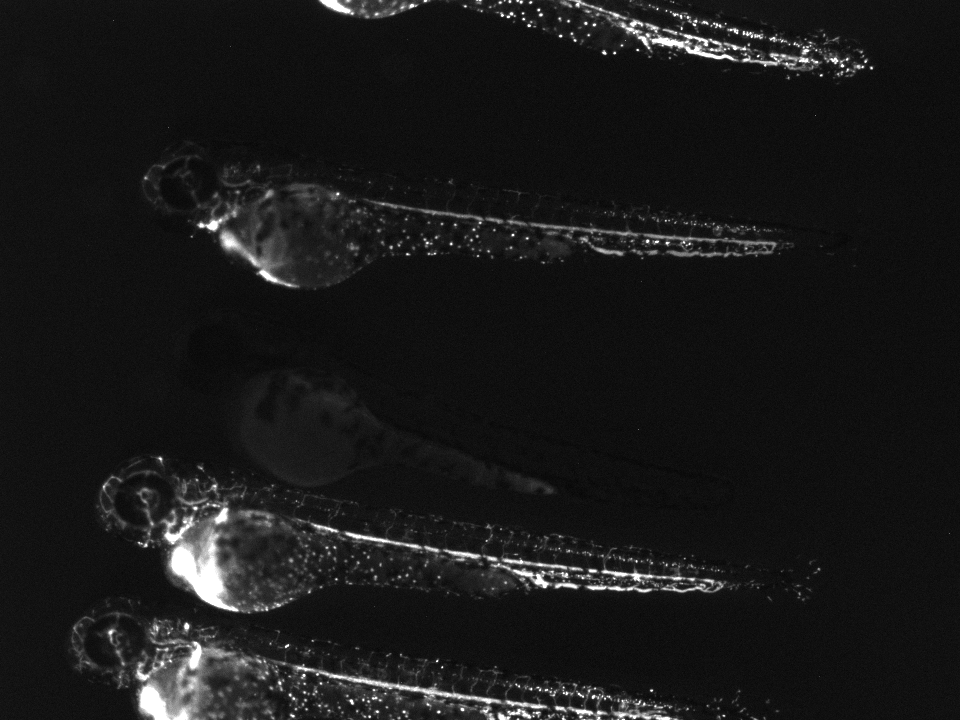

Supplement: Supplementary file 7 — Source data Fig. 3 [file 44321_2025_368_MOESM7_ESM.zip › FIGURE_3/3D/DASATINIB_1uM (21).tif]

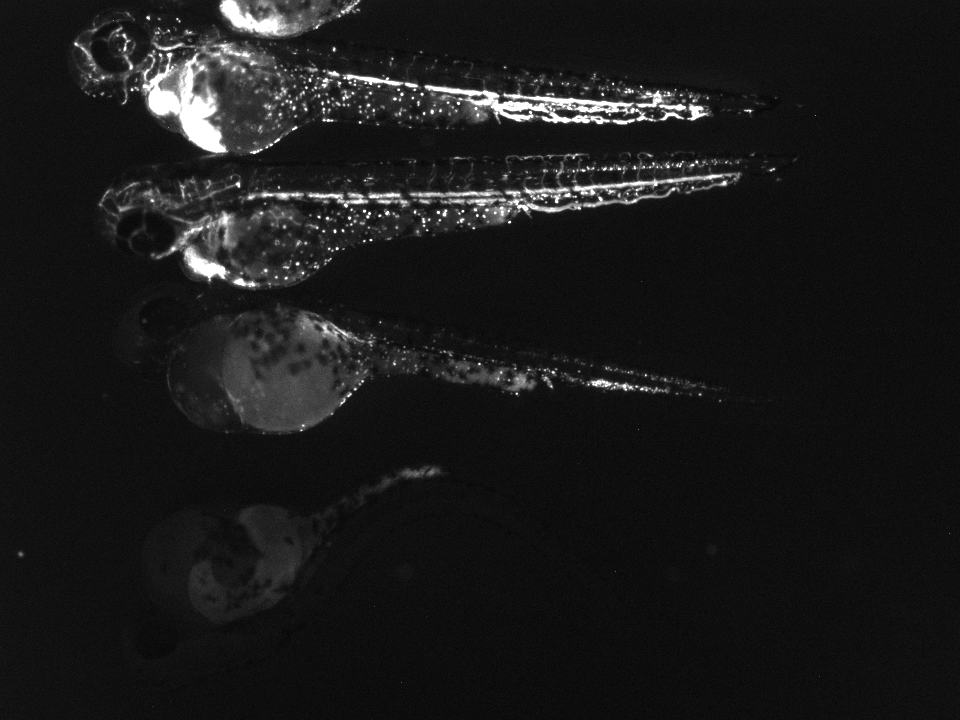

Supplement: Supplementary file 7 — Source data Fig. 3 [file 44321_2025_368_MOESM7_ESM.zip › FIGURE_3/3D/DASATINIB_1uM (22).tif]

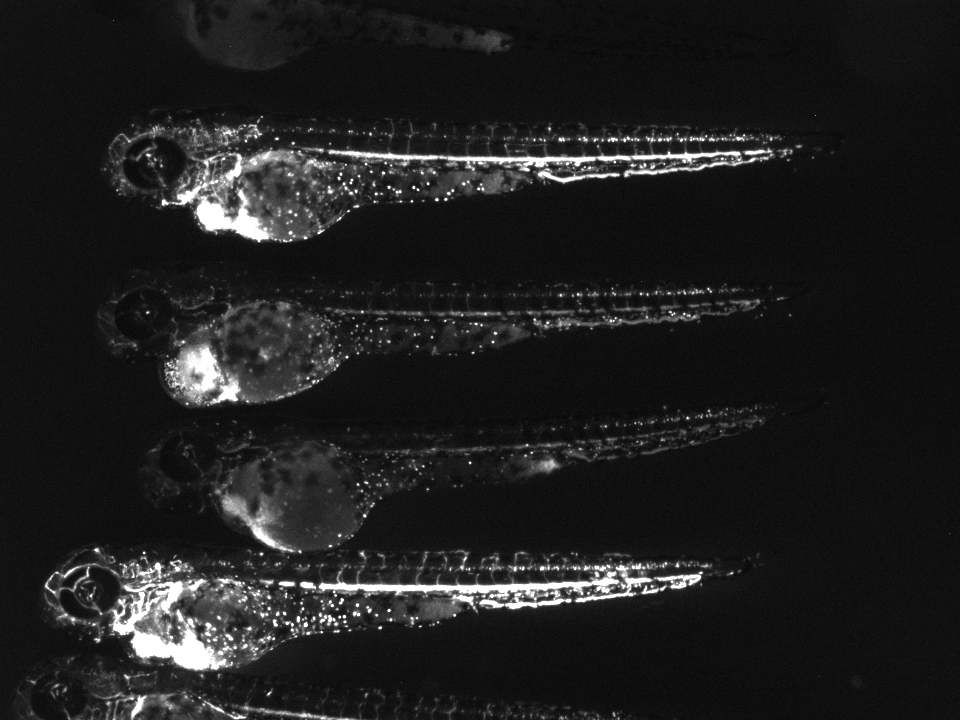

Supplement: Supplementary file 7 — Source data Fig. 3 [file 44321_2025_368_MOESM7_ESM.zip › FIGURE_3/3D/DASATINIB_1uM (4).tif]

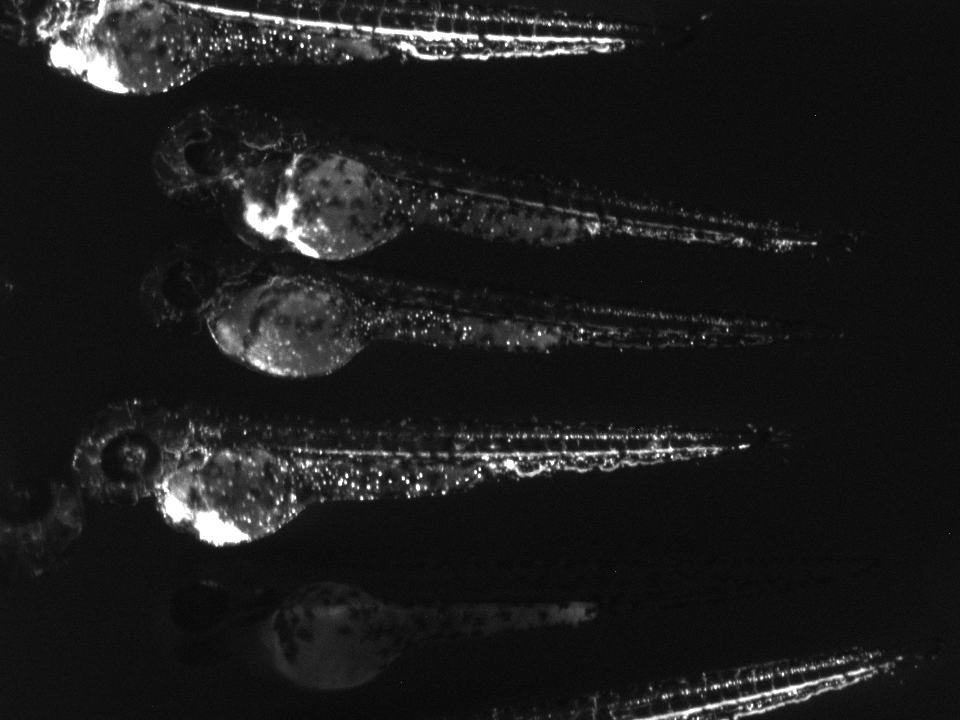

Supplement: Supplementary file 7 — Source data Fig. 3 [file 44321_2025_368_MOESM7_ESM.zip › FIGURE_3/3D/DASATINIB_1uM (6).tif]

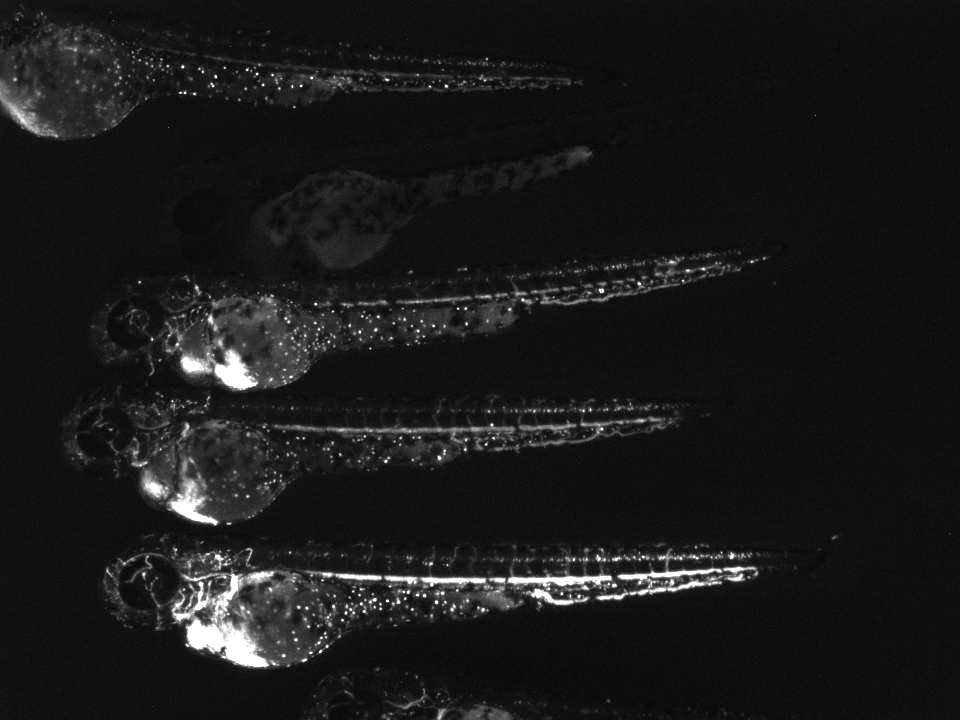

Supplement: Supplementary file 7 — Source data Fig. 3 [file 44321_2025_368_MOESM7_ESM.zip › FIGURE_3/3D/DASATINIB_1uM (8).tif]

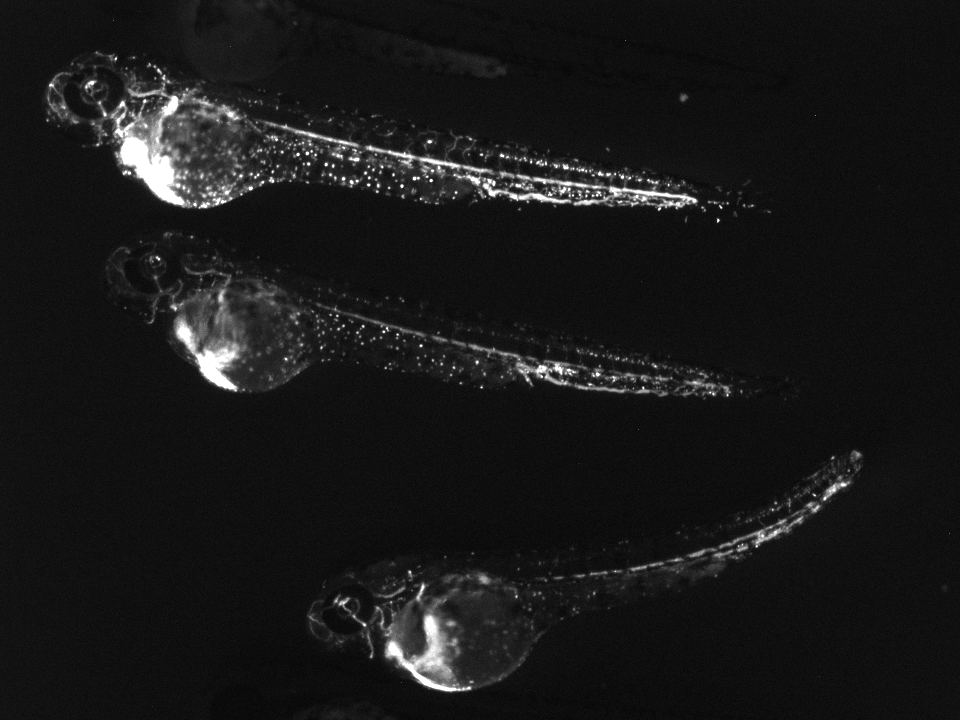

Supplement: Supplementary file 7 — Source data Fig. 3 [file 44321_2025_368_MOESM7_ESM.zip › FIGURE_3/3D/DMSO (1).tif]

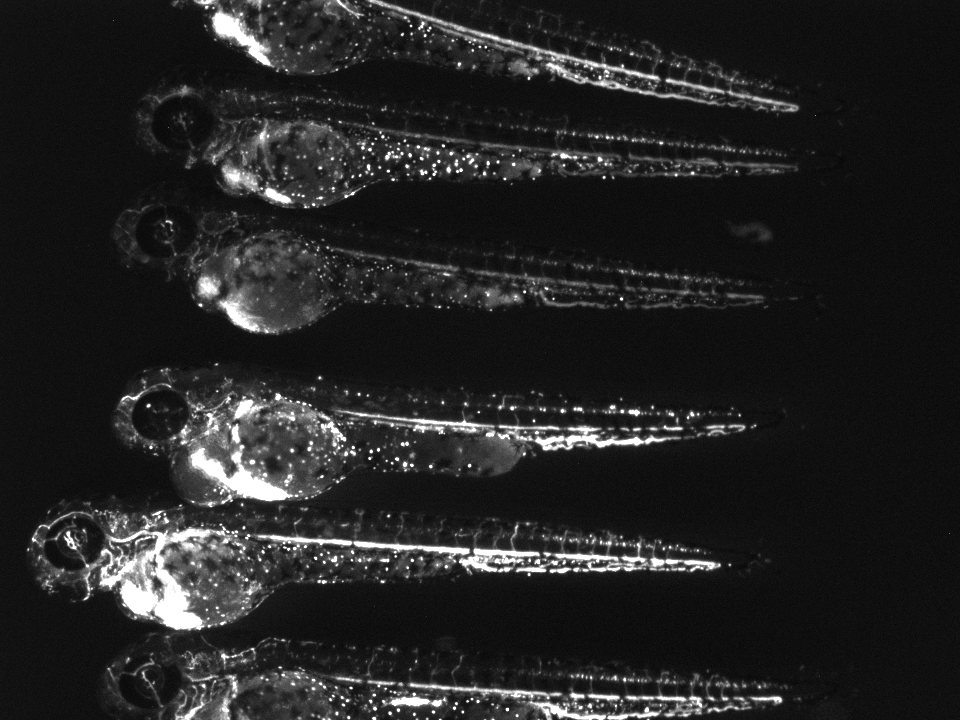

Supplement: Supplementary file 7 — Source data Fig. 3 [file 44321_2025_368_MOESM7_ESM.zip › FIGURE_3/3D/DMSO (10).tif]

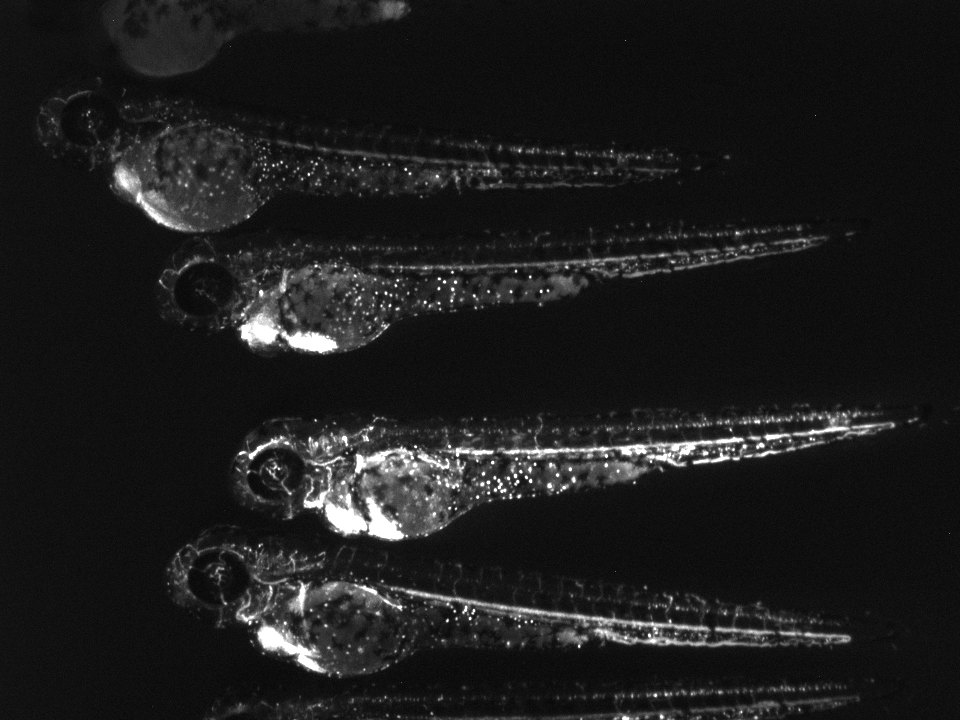

Supplement: Supplementary file 7 — Source data Fig. 3 [file 44321_2025_368_MOESM7_ESM.zip › FIGURE_3/3D/DMSO (11).tif]

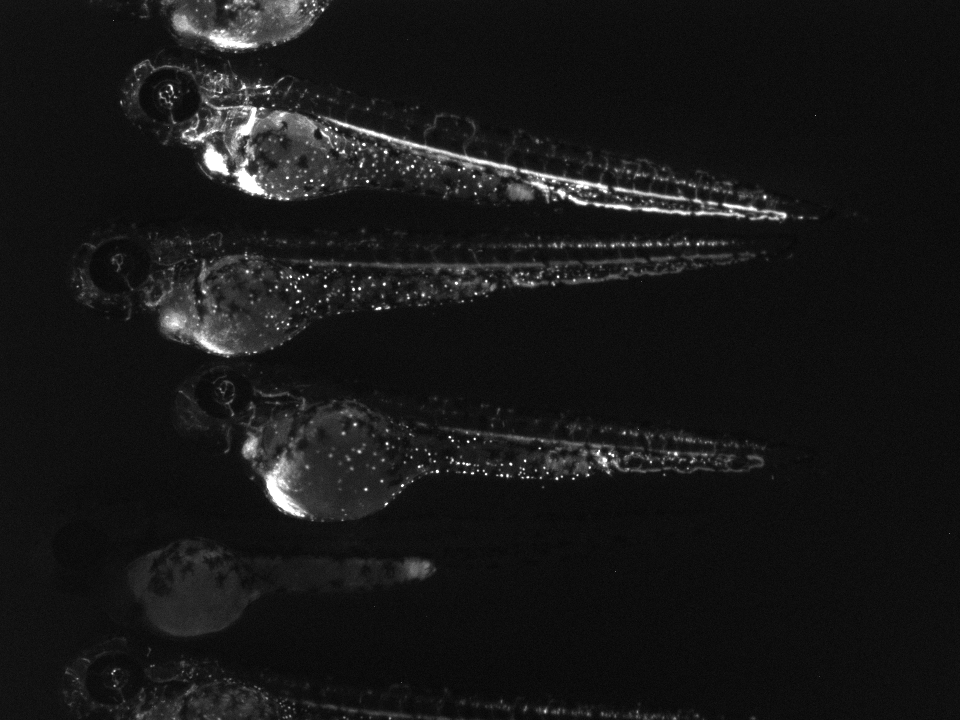

Supplement: Supplementary file 7 — Source data Fig. 3 [file 44321_2025_368_MOESM7_ESM.zip › FIGURE_3/3D/DMSO (12).tif]

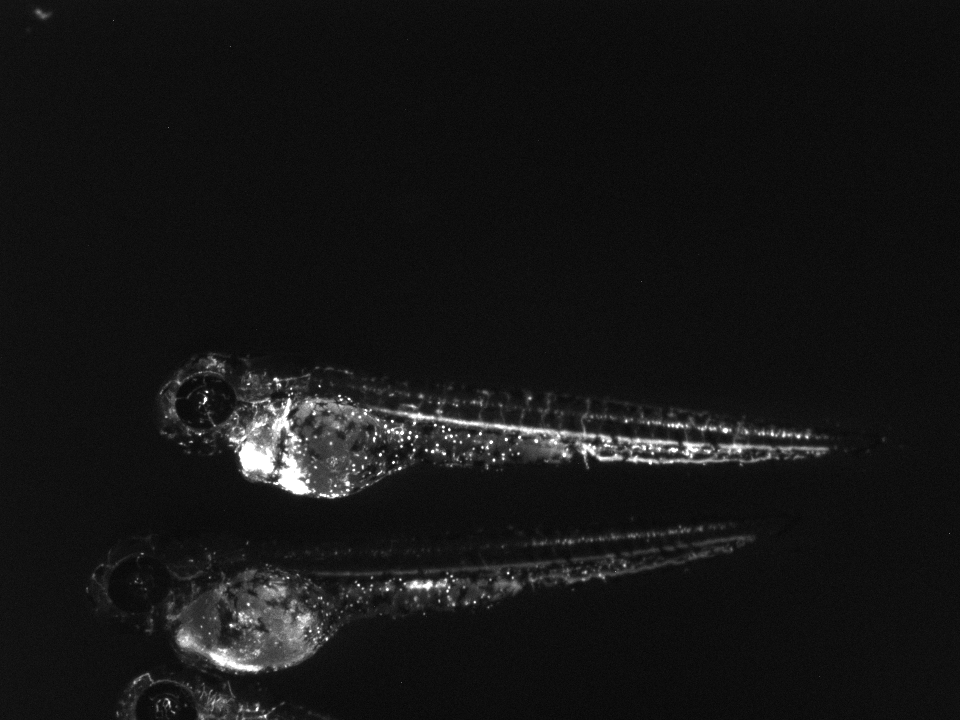

Supplement: Supplementary file 7 — Source data Fig. 3 [file 44321_2025_368_MOESM7_ESM.zip › FIGURE_3/3D/DMSO (13).tif]

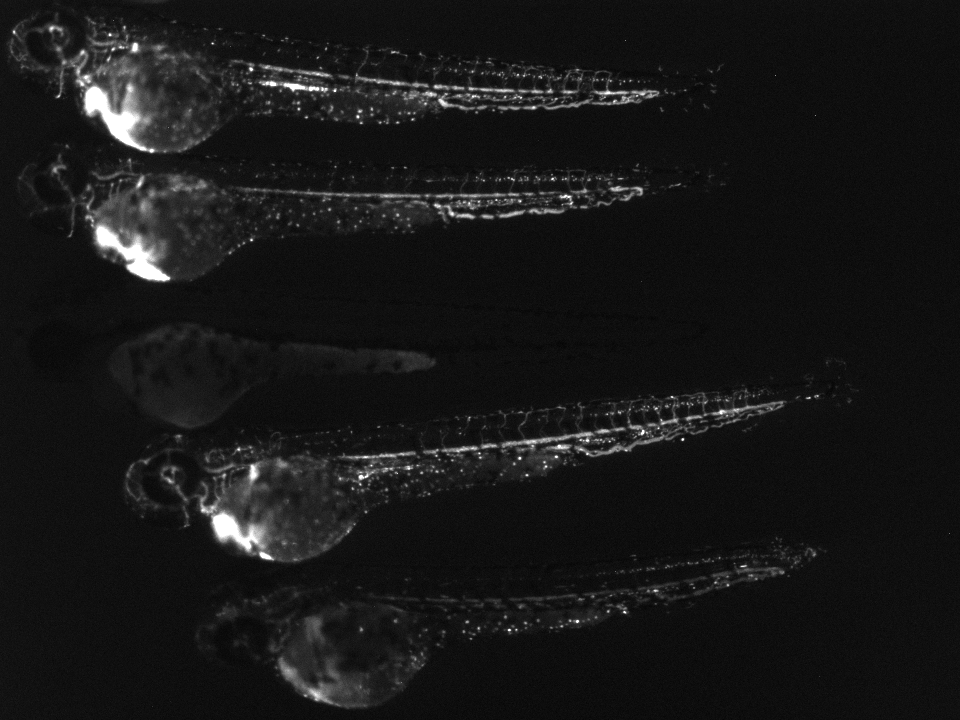

Supplement: Supplementary file 7 — Source data Fig. 3 [file 44321_2025_368_MOESM7_ESM.zip › FIGURE_3/3D/DMSO (2).tif]

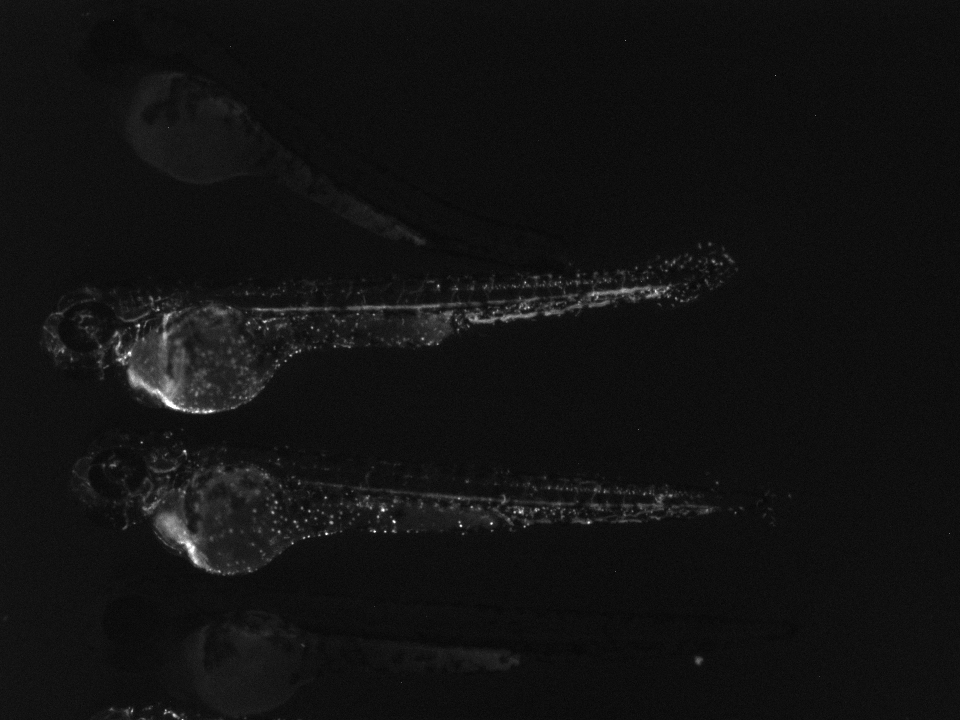

Supplement: Supplementary file 7 — Source data Fig. 3 [file 44321_2025_368_MOESM7_ESM.zip › FIGURE_3/3D/DMSO (3).tif]

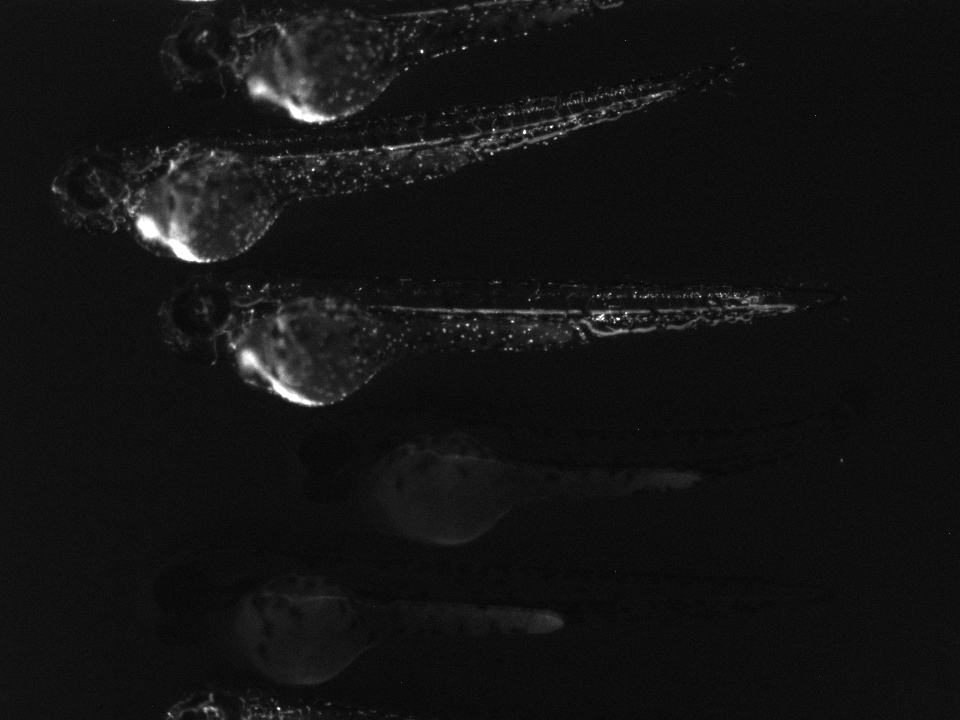

Supplement: Supplementary file 7 — Source data Fig. 3 [file 44321_2025_368_MOESM7_ESM.zip › FIGURE_3/3D/DMSO (4).tif]

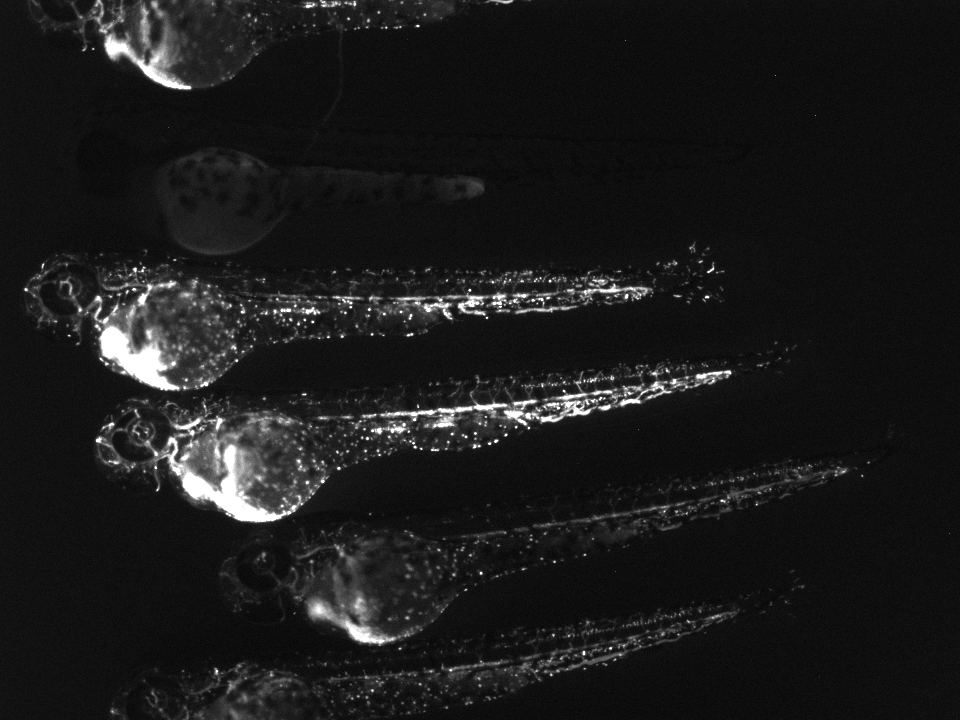

Supplement: Supplementary file 7 — Source data Fig. 3 [file 44321_2025_368_MOESM7_ESM.zip › FIGURE_3/3D/DMSO (5).tif]

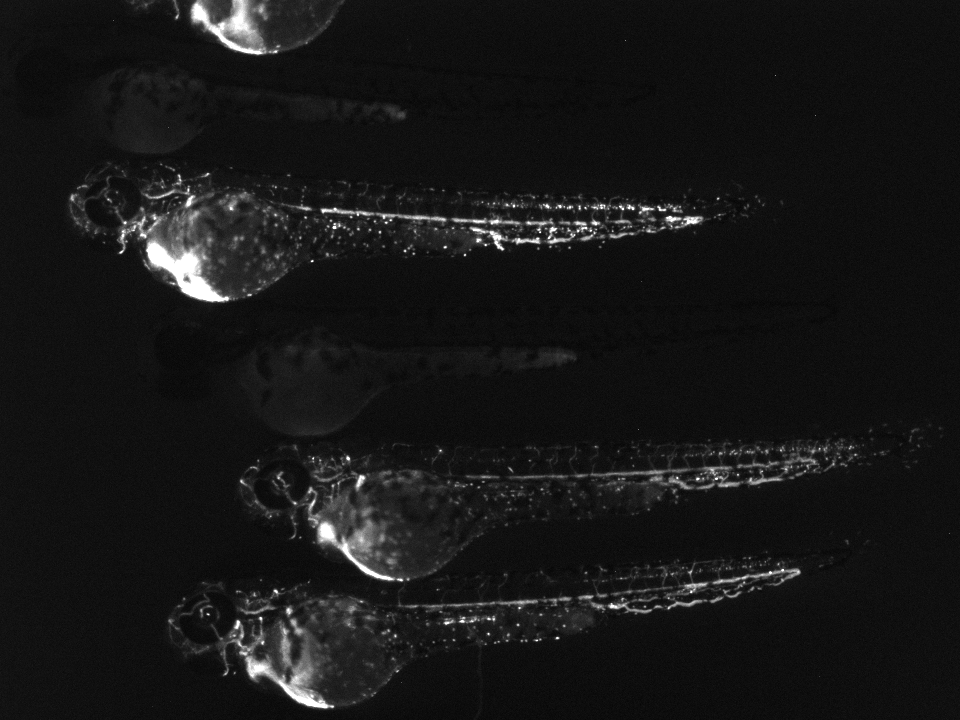

Supplement: Supplementary file 7 — Source data Fig. 3 [file 44321_2025_368_MOESM7_ESM.zip › FIGURE_3/3D/DMSO (6).tif]

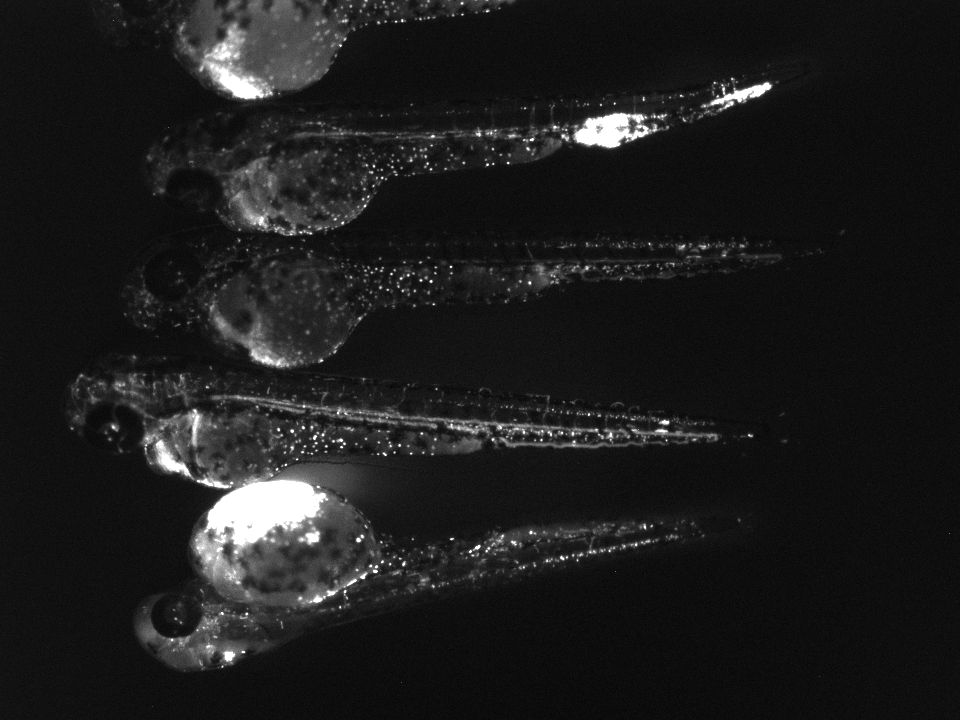

Supplement: Supplementary file 7 — Source data Fig. 3 [file 44321_2025_368_MOESM7_ESM.zip › FIGURE_3/3D/DMSO (7).tif]

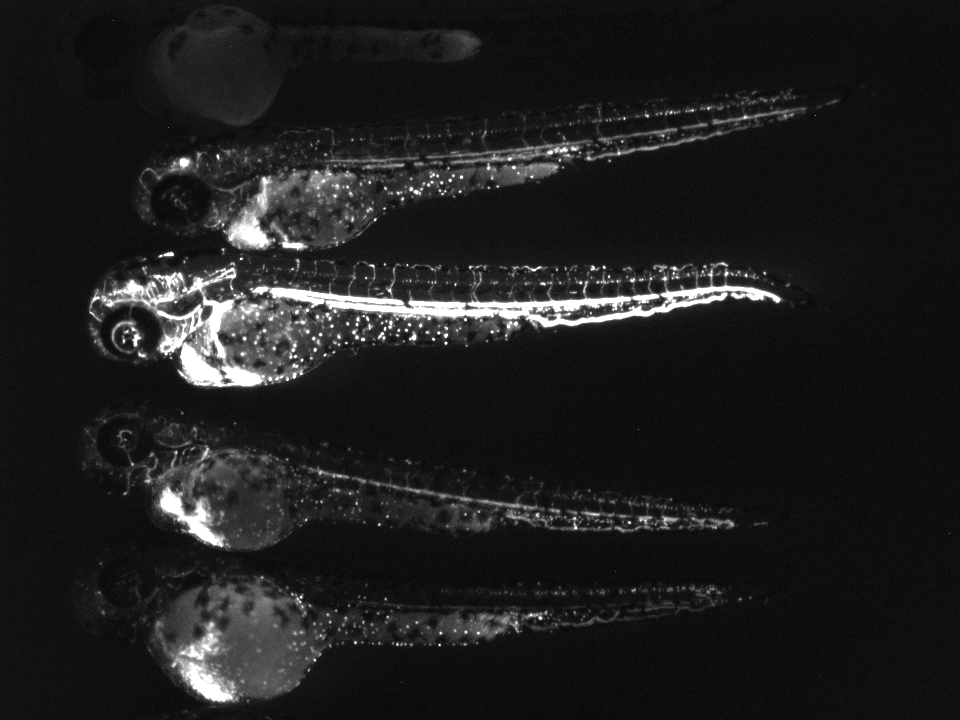

Supplement: Supplementary file 7 — Source data Fig. 3 [file 44321_2025_368_MOESM7_ESM.zip › FIGURE_3/3D/DMSO (8).tif]

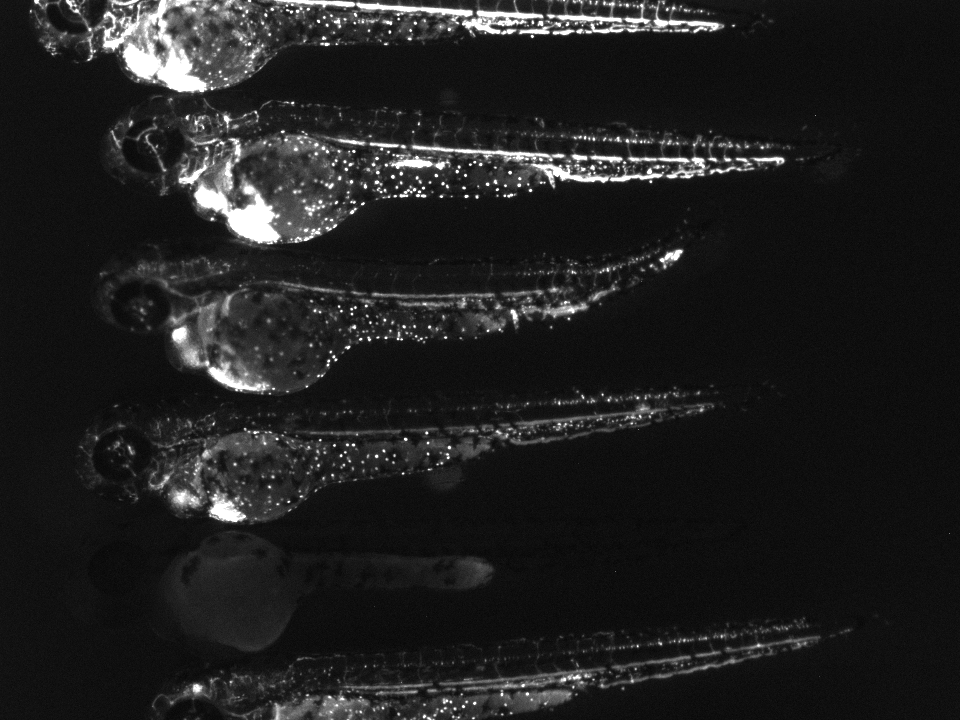

Supplement: Supplementary file 7 — Source data Fig. 3 [file 44321_2025_368_MOESM7_ESM.zip › FIGURE_3/3D/DMSO (9).tif]

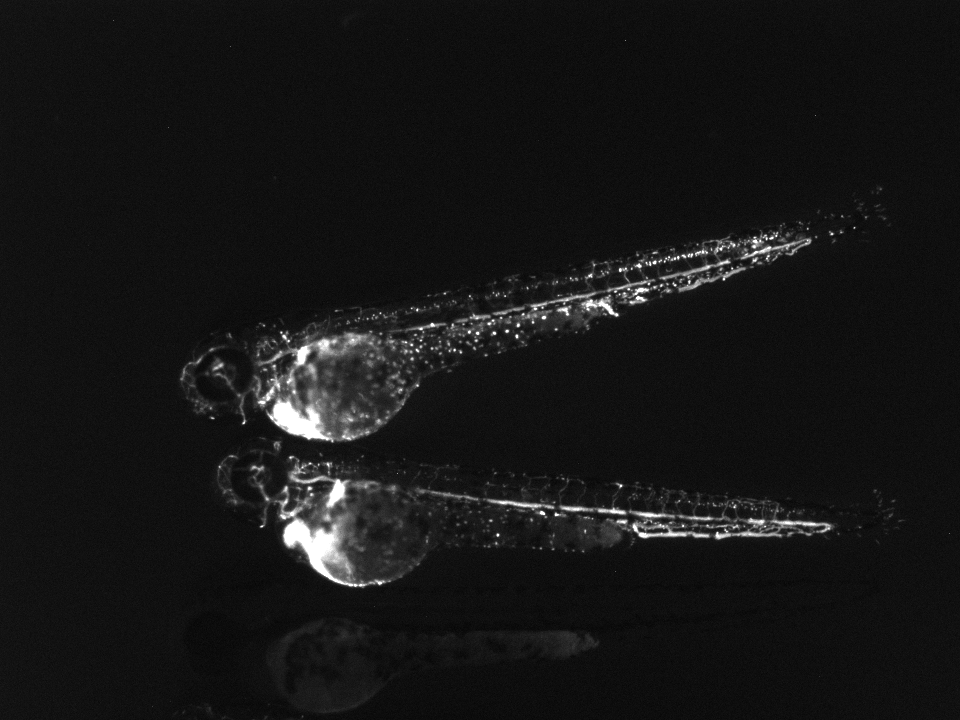

Supplement: Supplementary file 7 — Source data Fig. 3 [file 44321_2025_368_MOESM7_ESM.zip › FIGURE_3/3D/IMATINIB_10uM (1).tif]

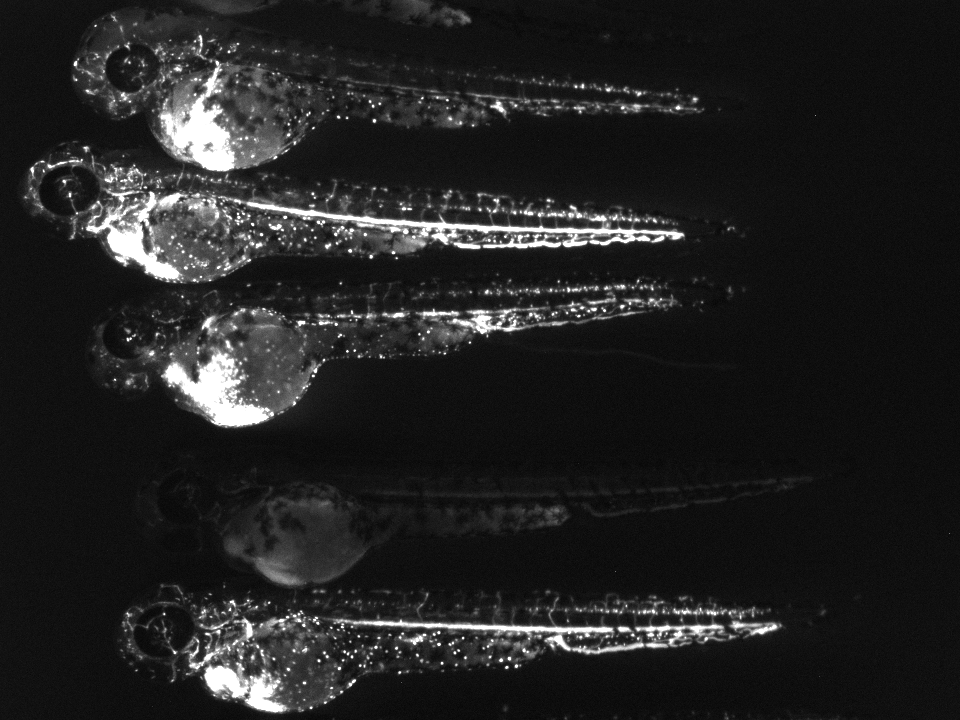

Supplement: Supplementary file 7 — Source data Fig. 3 [file 44321_2025_368_MOESM7_ESM.zip › FIGURE_3/3D/IMATINIB_10uM (10).tif]

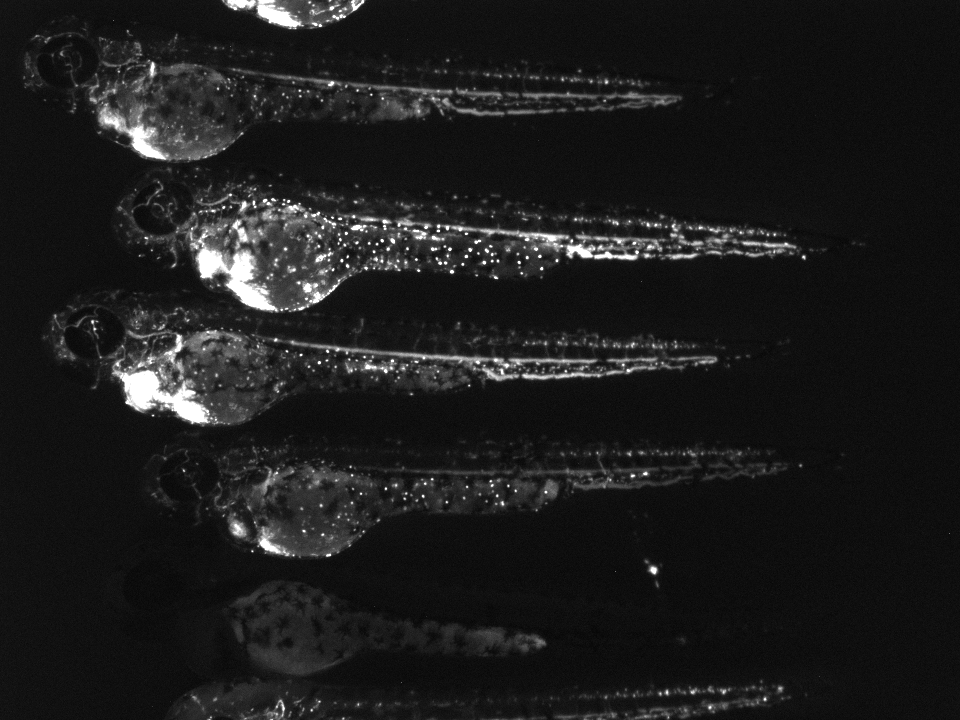

Supplement: Supplementary file 7 — Source data Fig. 3 [file 44321_2025_368_MOESM7_ESM.zip › FIGURE_3/3D/IMATINIB_10uM (11).tif]

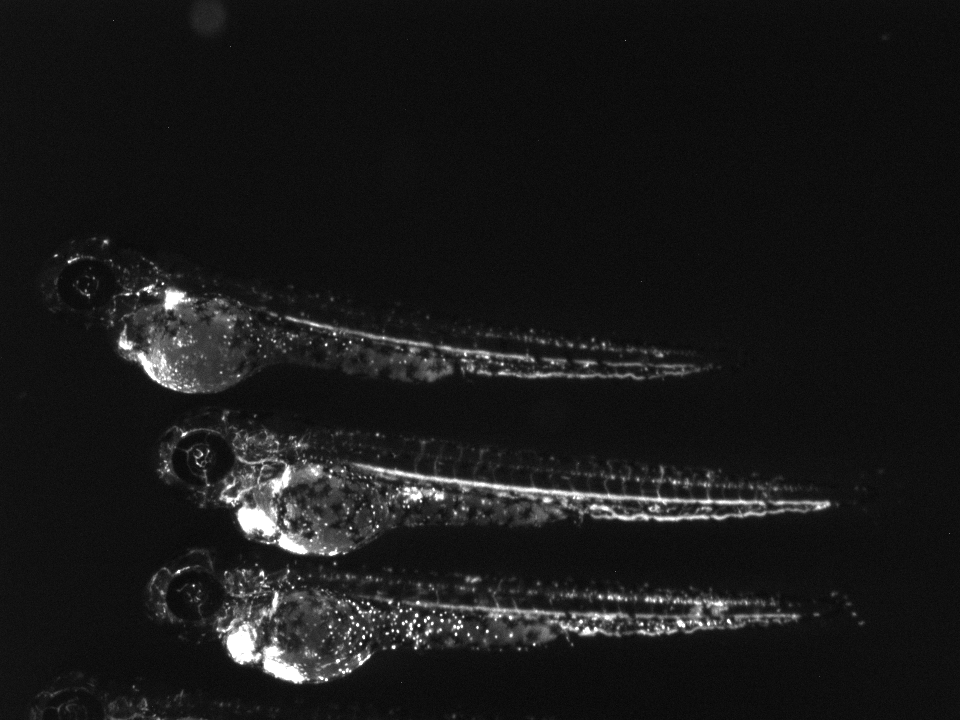

Supplement: Supplementary file 7 — Source data Fig. 3 [file 44321_2025_368_MOESM7_ESM.zip › FIGURE_3/3D/IMATINIB_10uM (12).tif]

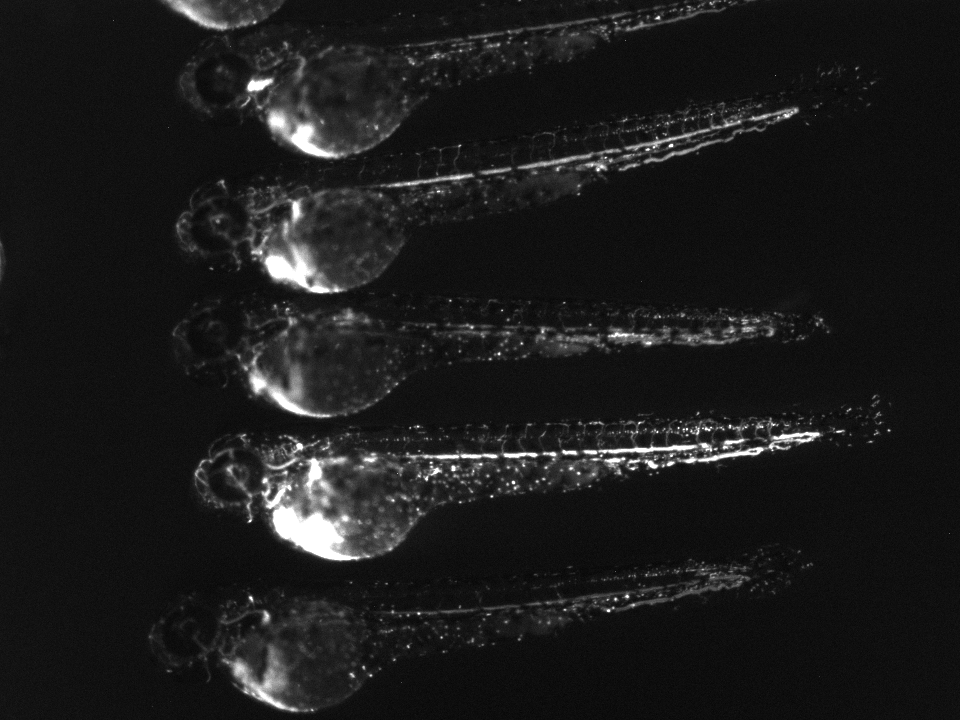

Supplement: Supplementary file 7 — Source data Fig. 3 [file 44321_2025_368_MOESM7_ESM.zip › FIGURE_3/3D/IMATINIB_10uM (13).tif]

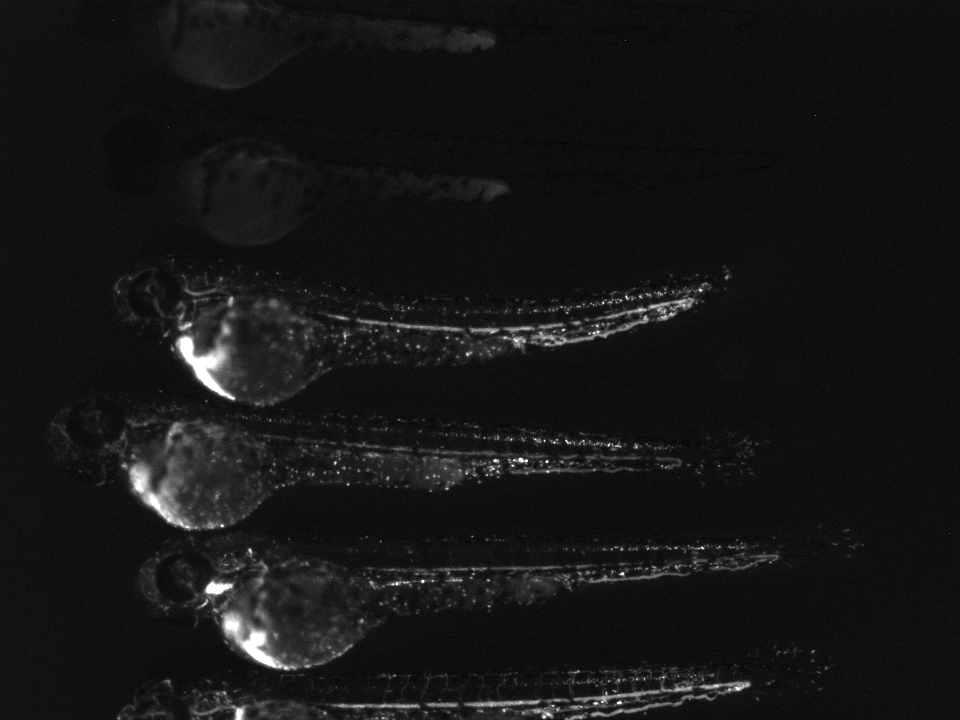

Supplement: Supplementary file 7 — Source data Fig. 3 [file 44321_2025_368_MOESM7_ESM.zip › FIGURE_3/3D/IMATINIB_10uM (14).tif]

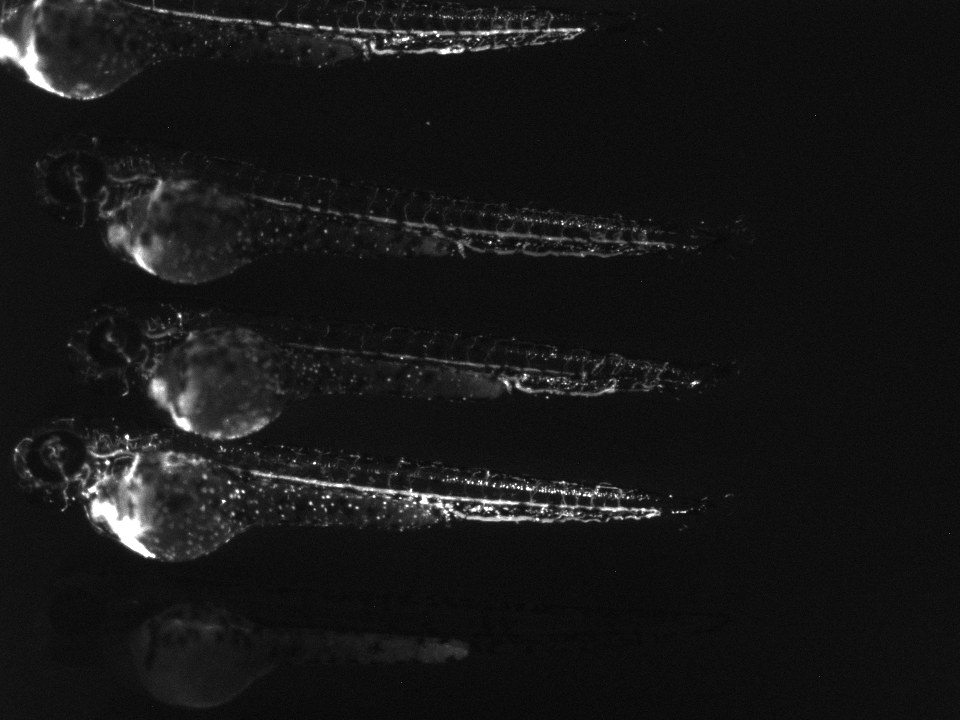

Supplement: Supplementary file 7 — Source data Fig. 3 [file 44321_2025_368_MOESM7_ESM.zip › FIGURE_3/3D/IMATINIB_10uM (15).tif]

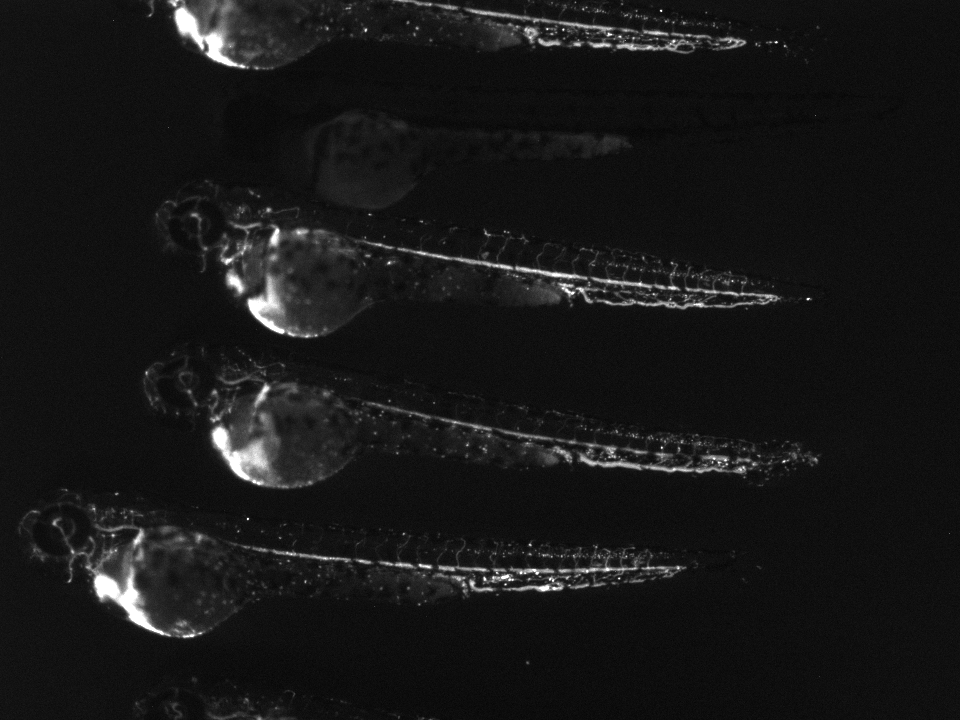

Supplement: Supplementary file 7 — Source data Fig. 3 [file 44321_2025_368_MOESM7_ESM.zip › FIGURE_3/3D/IMATINIB_10uM (16).tif]

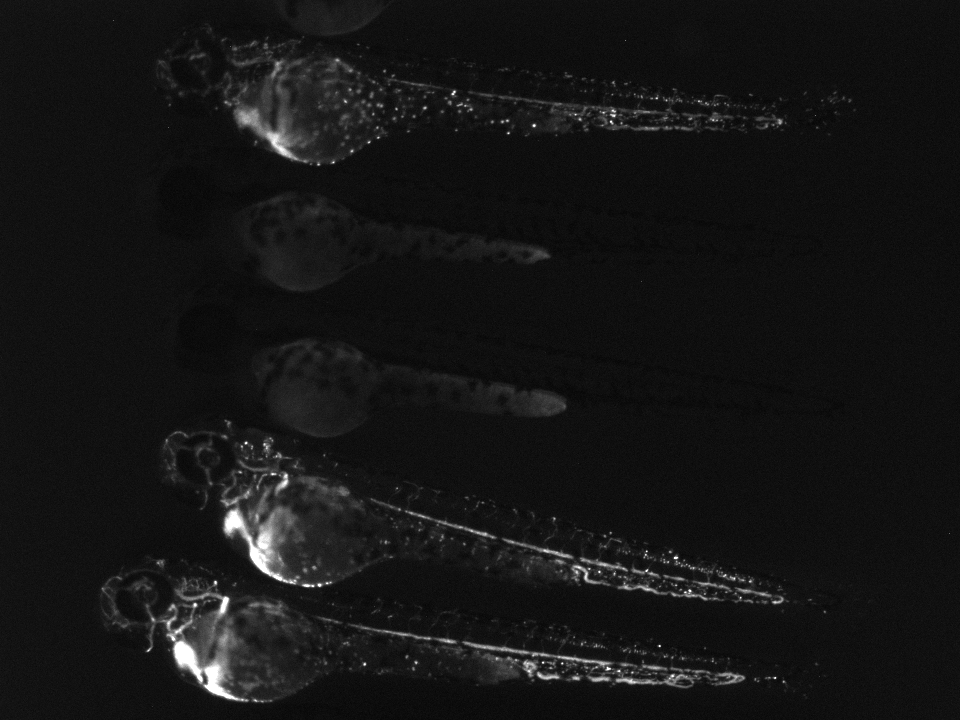

Supplement: Supplementary file 7 — Source data Fig. 3 [file 44321_2025_368_MOESM7_ESM.zip › FIGURE_3/3D/IMATINIB_10uM (2).tif]

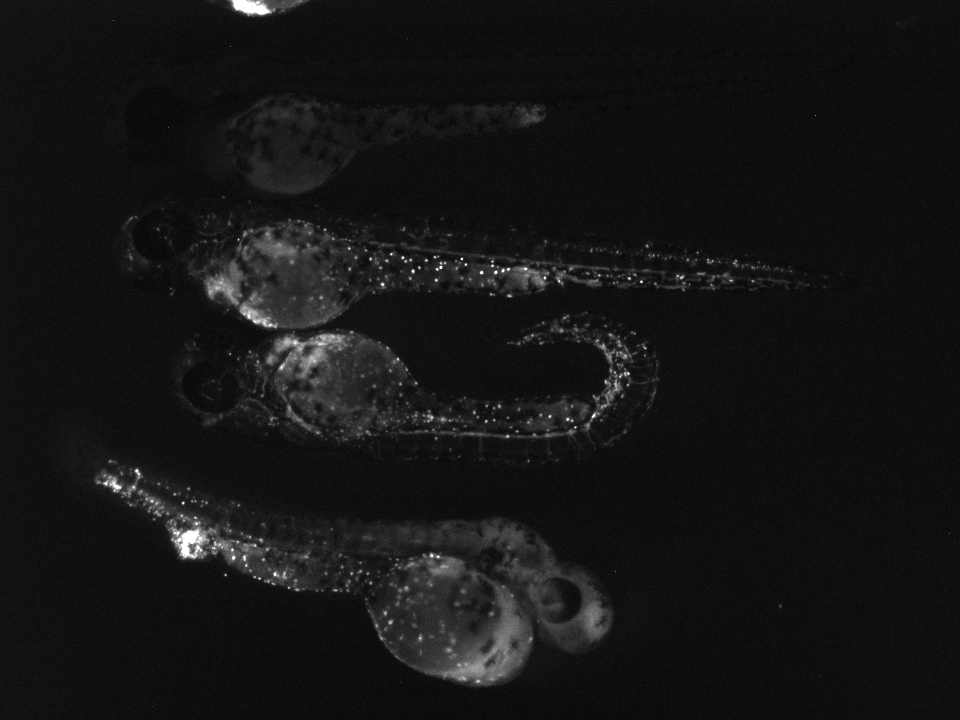

Supplement: Supplementary file 7 — Source data Fig. 3 [file 44321_2025_368_MOESM7_ESM.zip › FIGURE_3/3D/IMATINIB_10uM (4).tif]

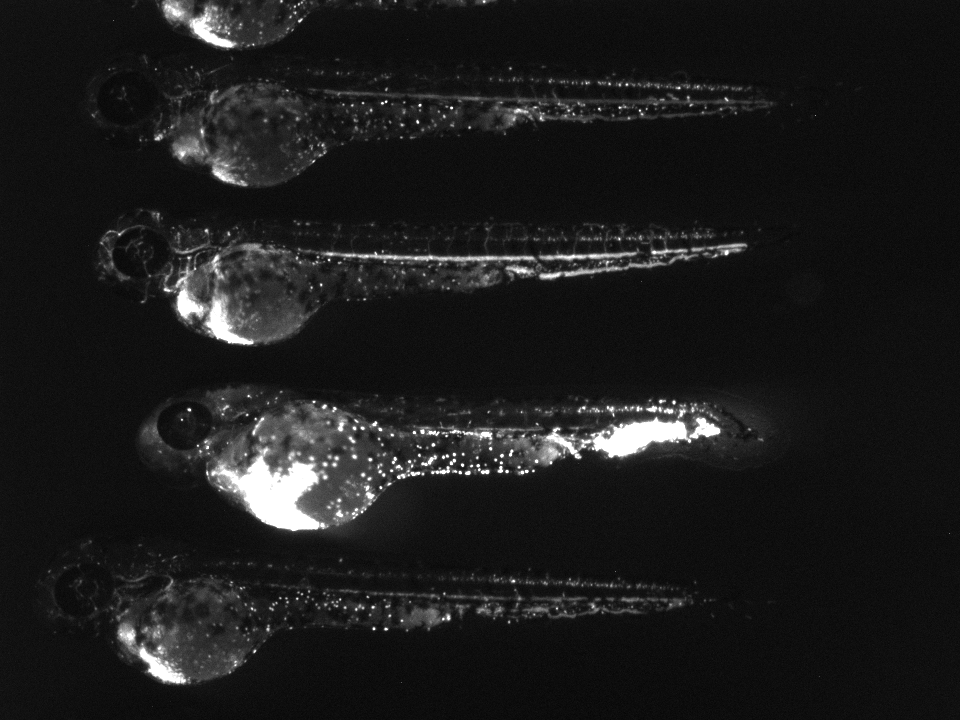

Supplement: Supplementary file 7 — Source data Fig. 3 [file 44321_2025_368_MOESM7_ESM.zip › FIGURE_3/3D/IMATINIB_10uM (6).tif]

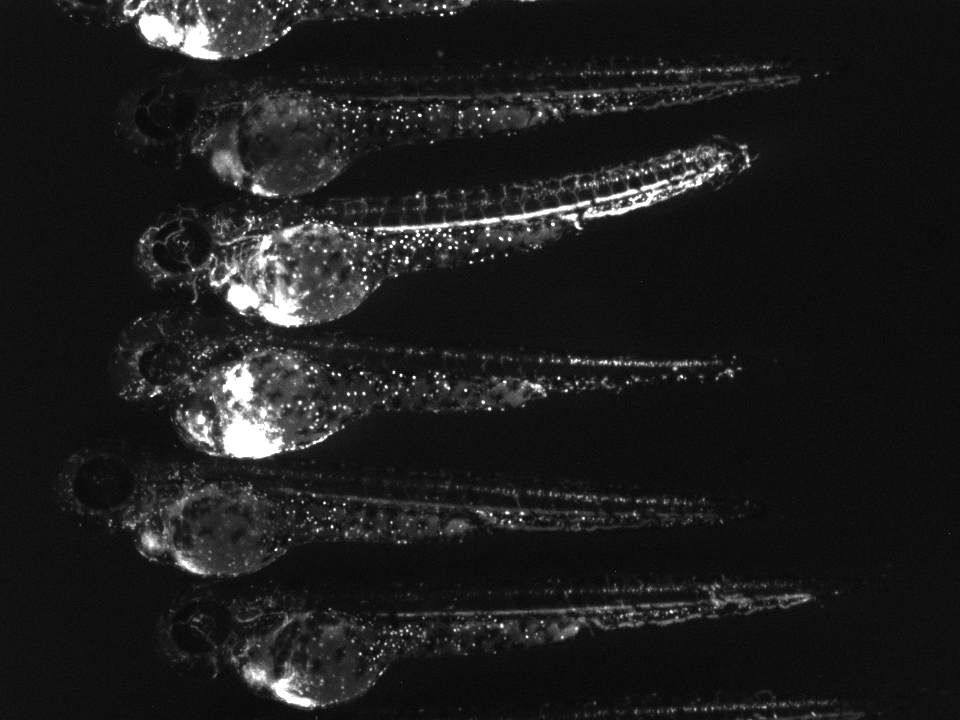

Supplement: Supplementary file 7 — Source data Fig. 3 [file 44321_2025_368_MOESM7_ESM.zip › FIGURE_3/3D/IMATINIB_10uM (8).tif]

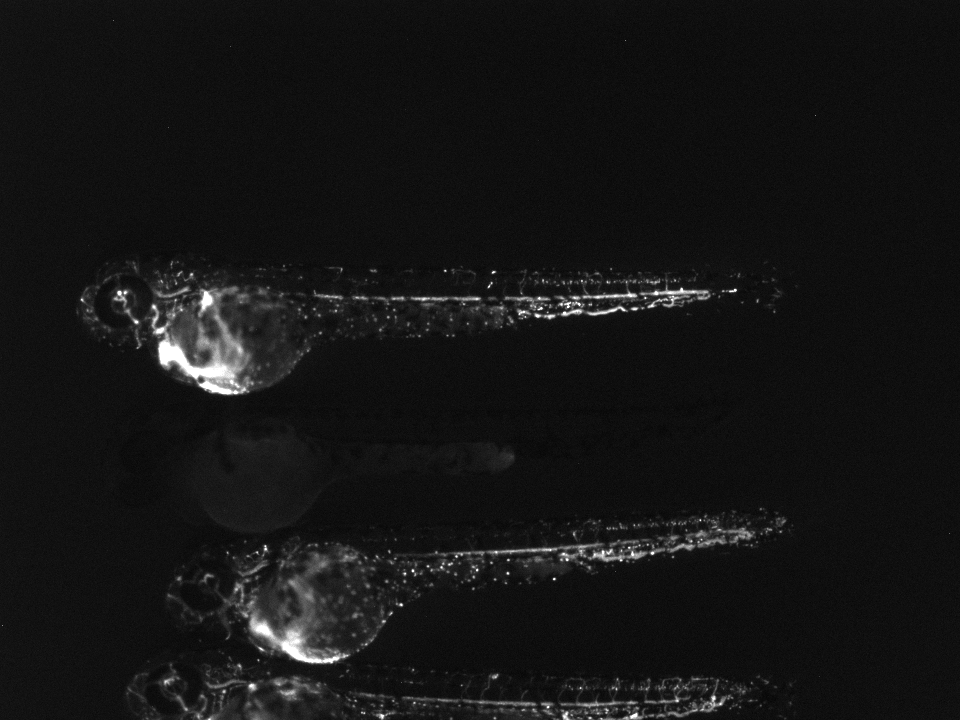

Supplement: Supplementary file 7 — Source data Fig. 3 [file 44321_2025_368_MOESM7_ESM.zip › FIGURE_3/3D/IMATINIB_1uM (1).tif]

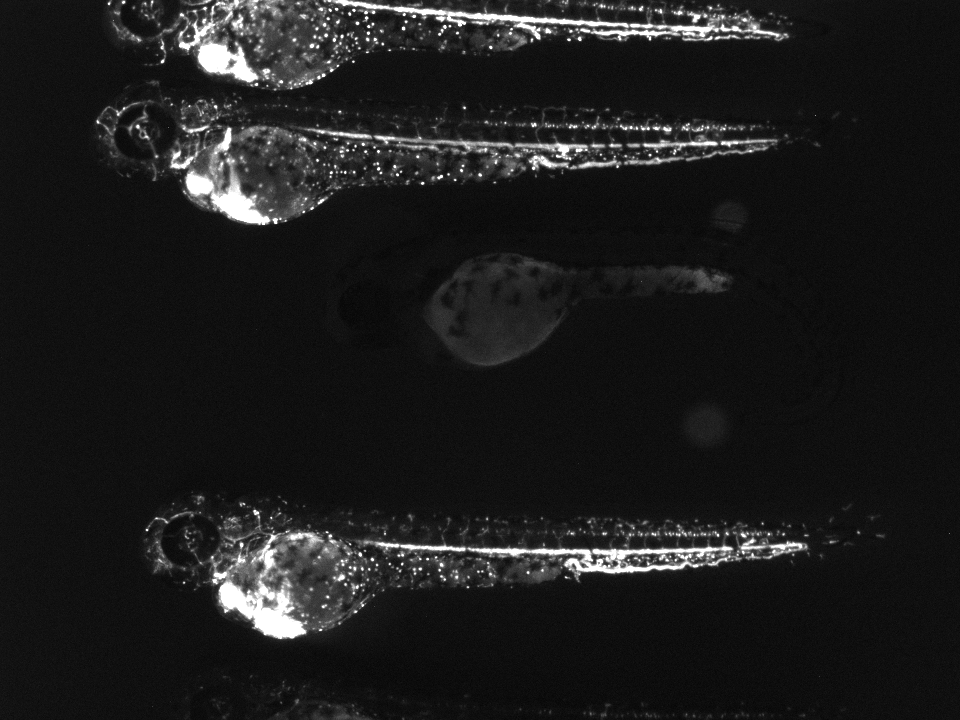

Supplement: Supplementary file 7 — Source data Fig. 3 [file 44321_2025_368_MOESM7_ESM.zip › FIGURE_3/3D/IMATINIB_1uM (10).tif]

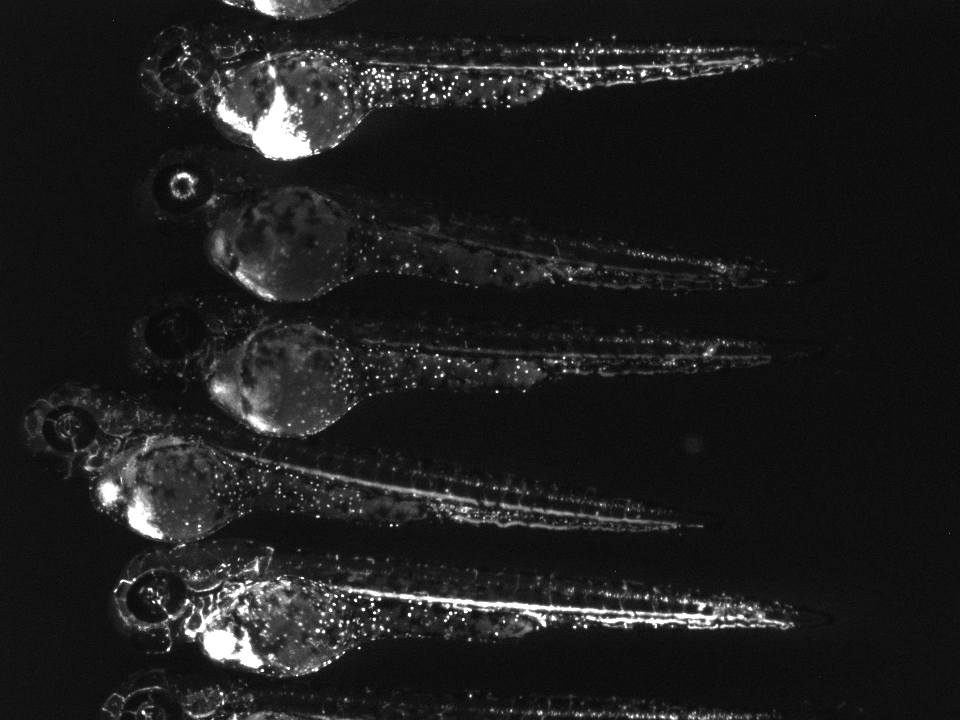

Supplement: Supplementary file 7 — Source data Fig. 3 [file 44321_2025_368_MOESM7_ESM.zip › FIGURE_3/3D/IMATINIB_1uM (11).tif]

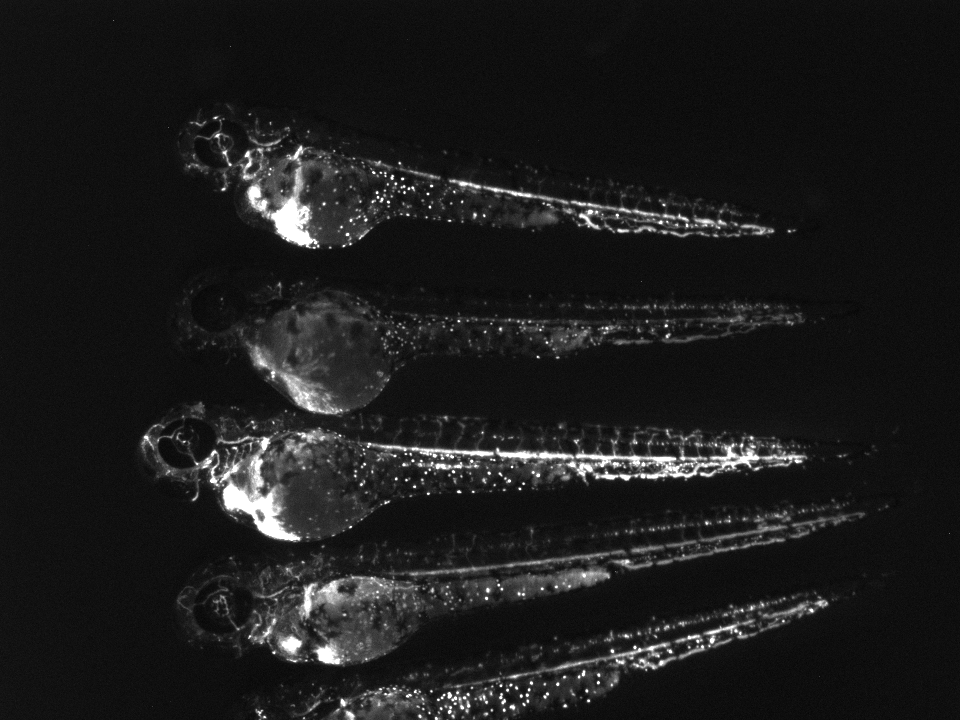

Supplement: Supplementary file 7 — Source data Fig. 3 [file 44321_2025_368_MOESM7_ESM.zip › FIGURE_3/3D/IMATINIB_1uM (12).tif]

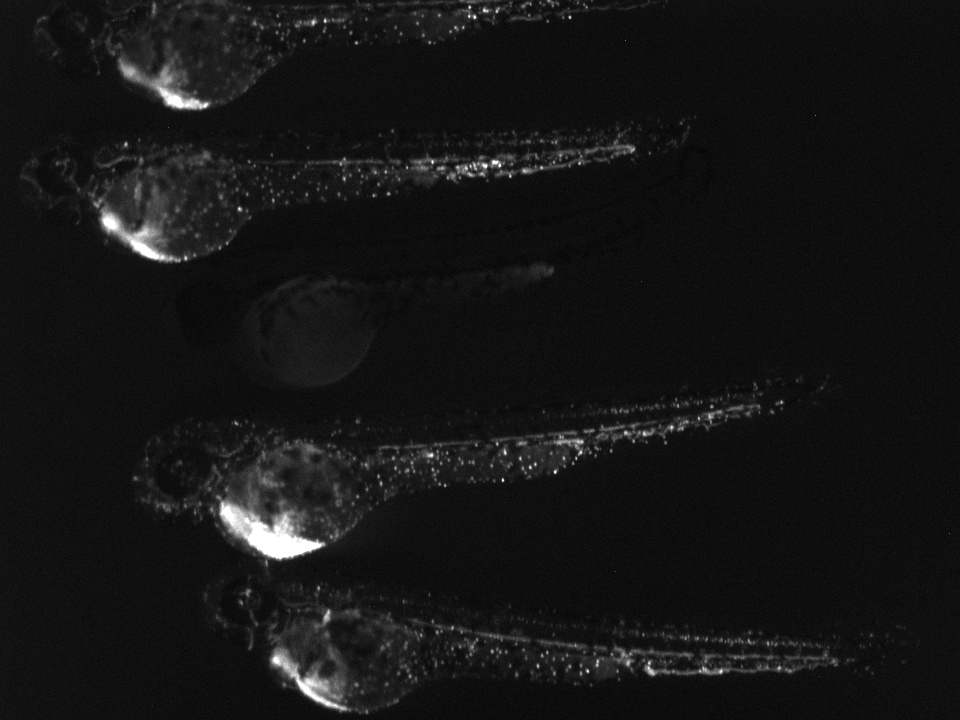

Supplement: Supplementary file 7 — Source data Fig. 3 [file 44321_2025_368_MOESM7_ESM.zip › FIGURE_3/3D/IMATINIB_1uM (13).tif]

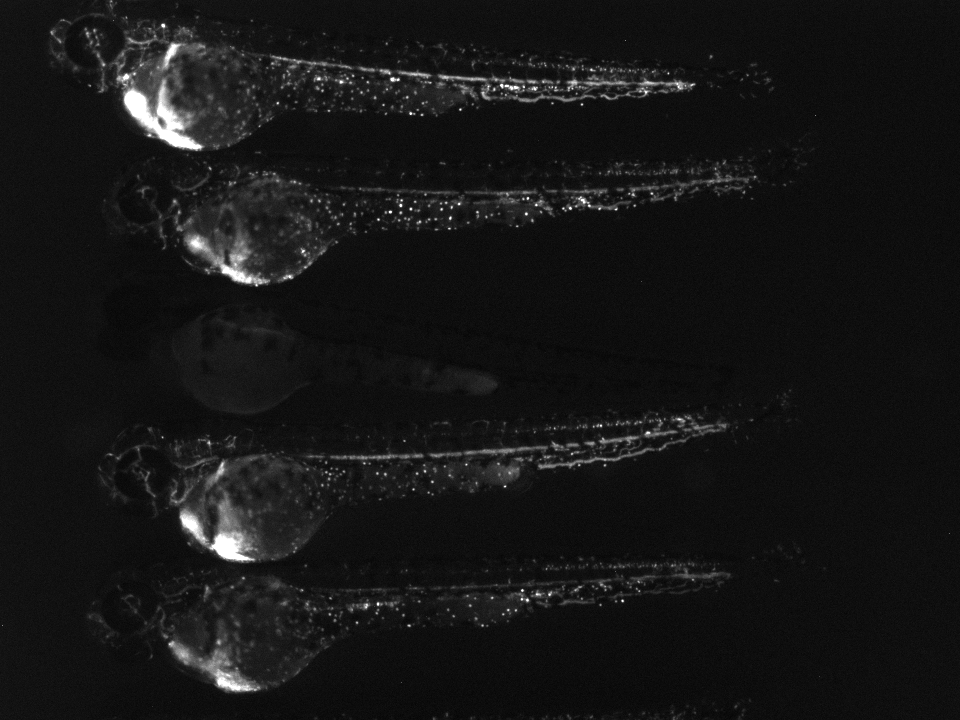

Supplement: Supplementary file 7 — Source data Fig. 3 [file 44321_2025_368_MOESM7_ESM.zip › FIGURE_3/3D/IMATINIB_1uM (14).tif]

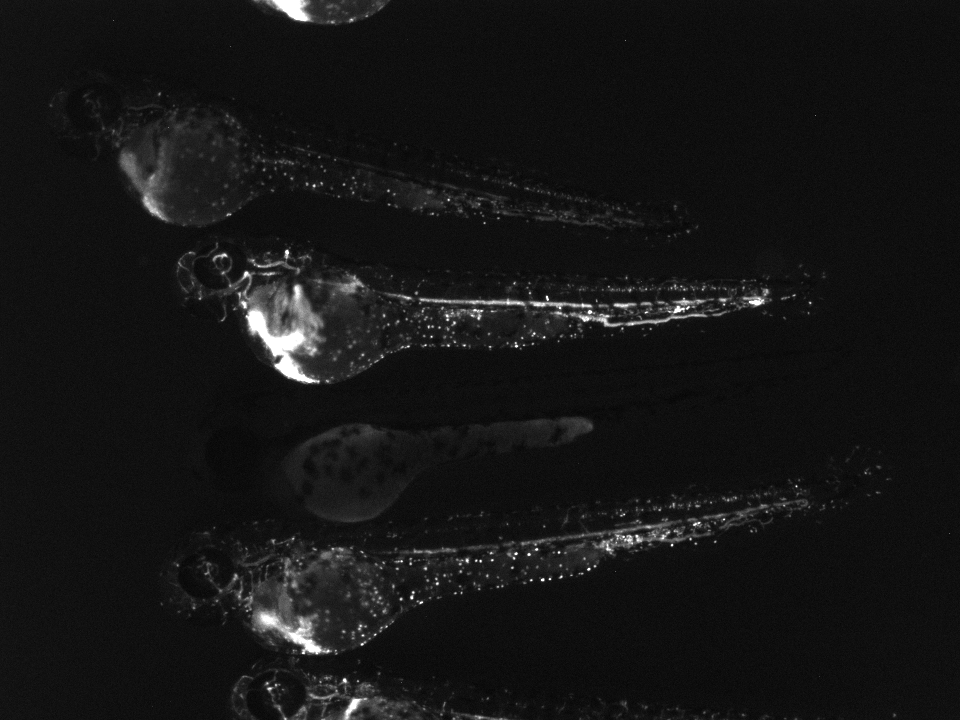

Supplement: Supplementary file 7 — Source data Fig. 3 [file 44321_2025_368_MOESM7_ESM.zip › FIGURE_3/3D/IMATINIB_1uM (15).tif]

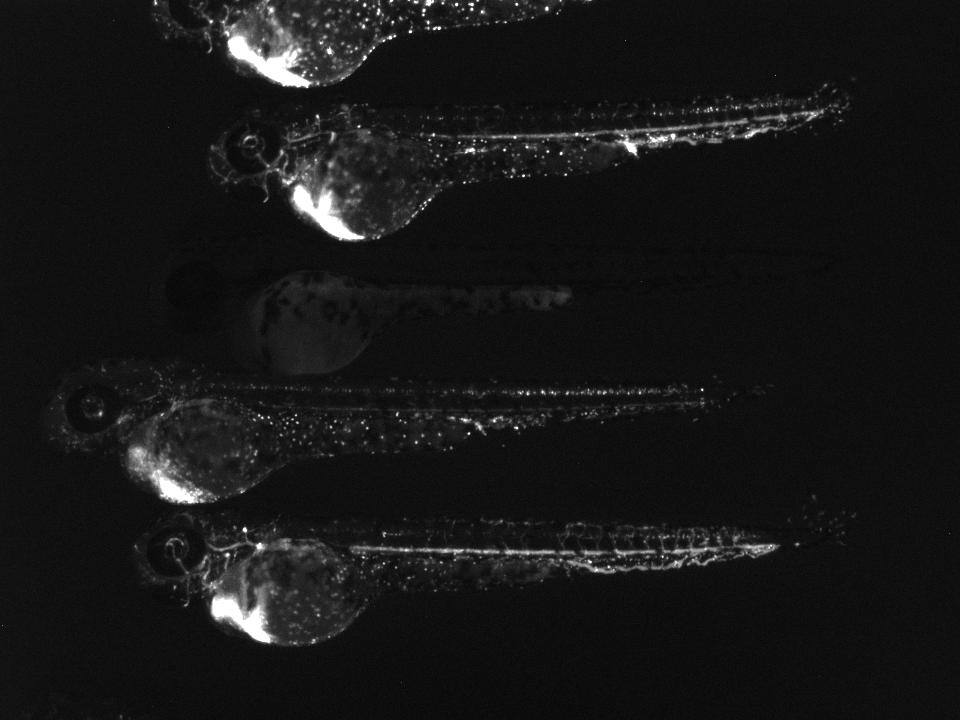

Supplement: Supplementary file 7 — Source data Fig. 3 [file 44321_2025_368_MOESM7_ESM.zip › FIGURE_3/3D/IMATINIB_1uM (16).tif]

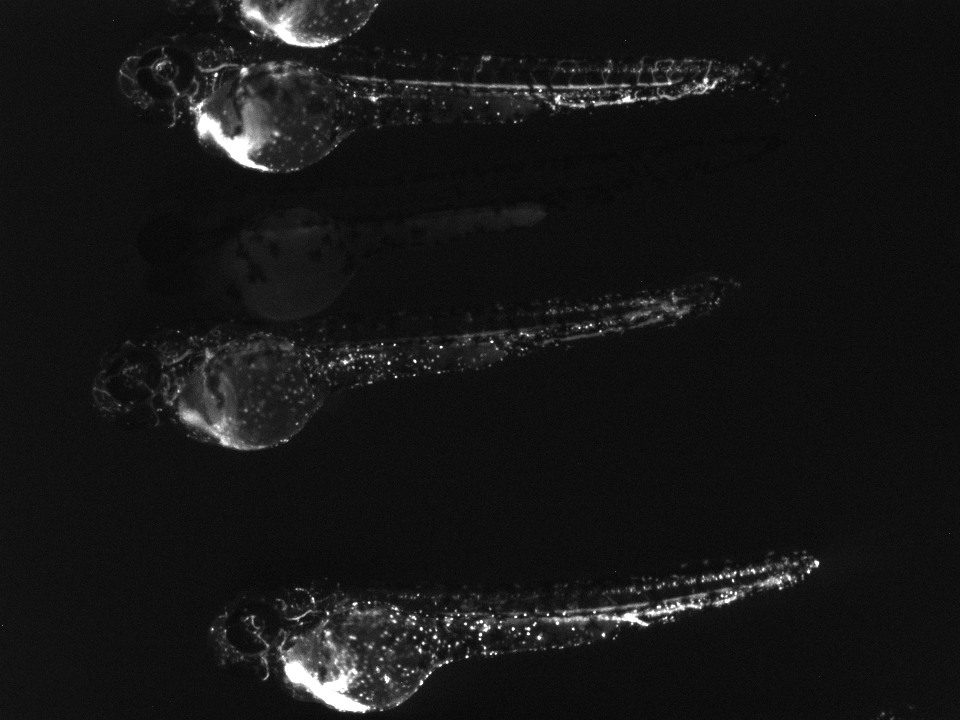

Supplement: Supplementary file 7 — Source data Fig. 3 [file 44321_2025_368_MOESM7_ESM.zip › FIGURE_3/3D/IMATINIB_1uM (2).tif]

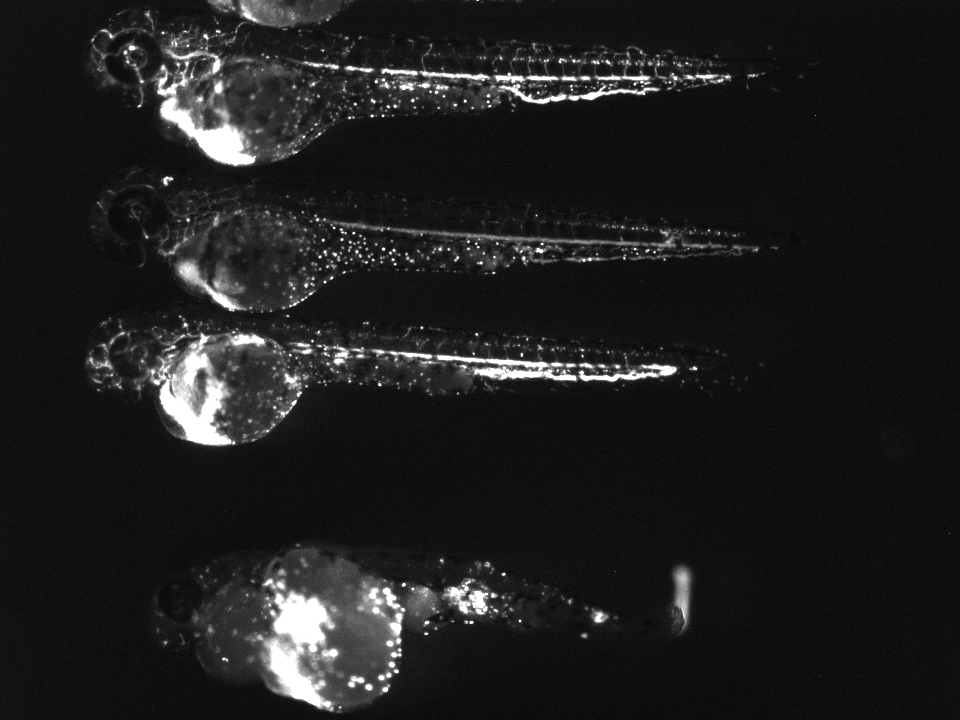

Supplement: Supplementary file 7 — Source data Fig. 3 [file 44321_2025_368_MOESM7_ESM.zip › FIGURE_3/3D/IMATINIB_1uM (4).tif]

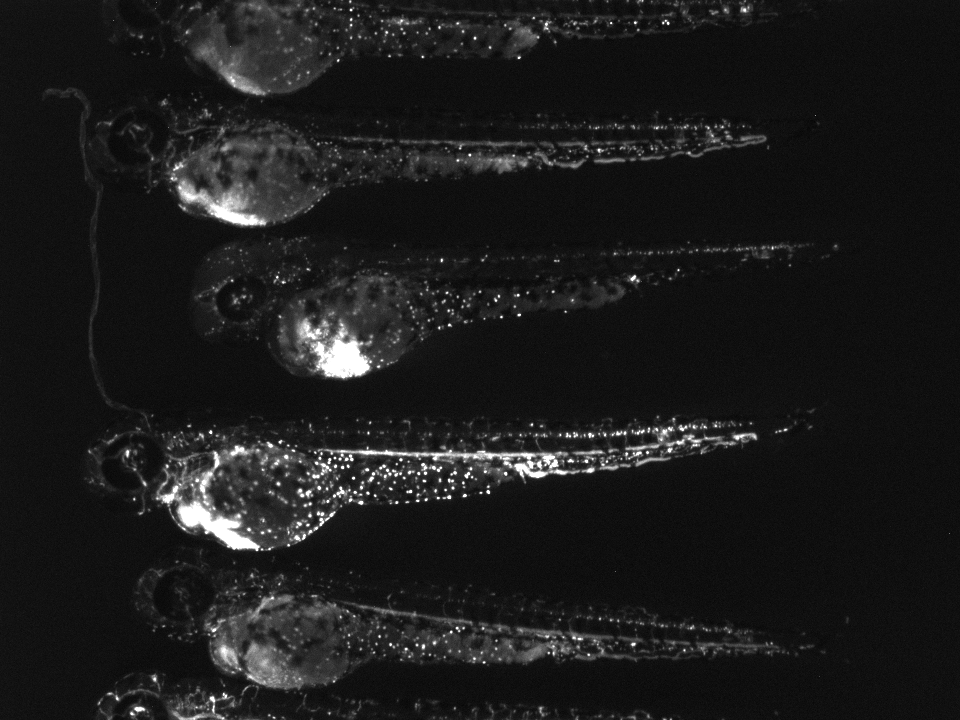

Supplement: Supplementary file 7 — Source data Fig. 3 [file 44321_2025_368_MOESM7_ESM.zip › FIGURE_3/3D/IMATINIB_1uM (6).tif]

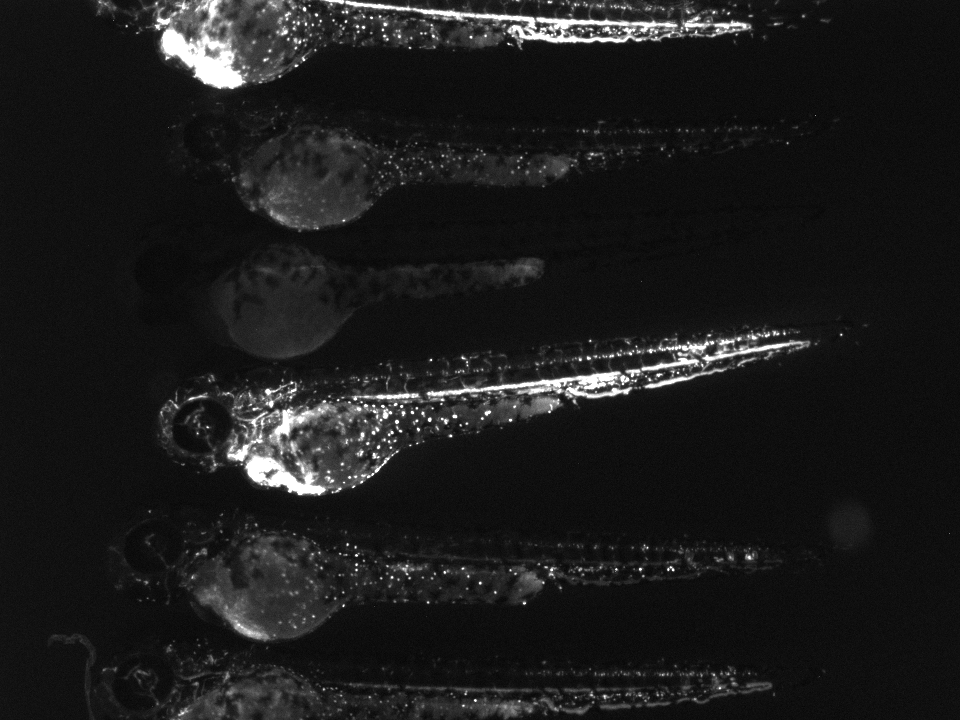

Supplement: Supplementary file 7 — Source data Fig. 3 [file 44321_2025_368_MOESM7_ESM.zip › FIGURE_3/3D/IMATINIB_1uM (8).tif]

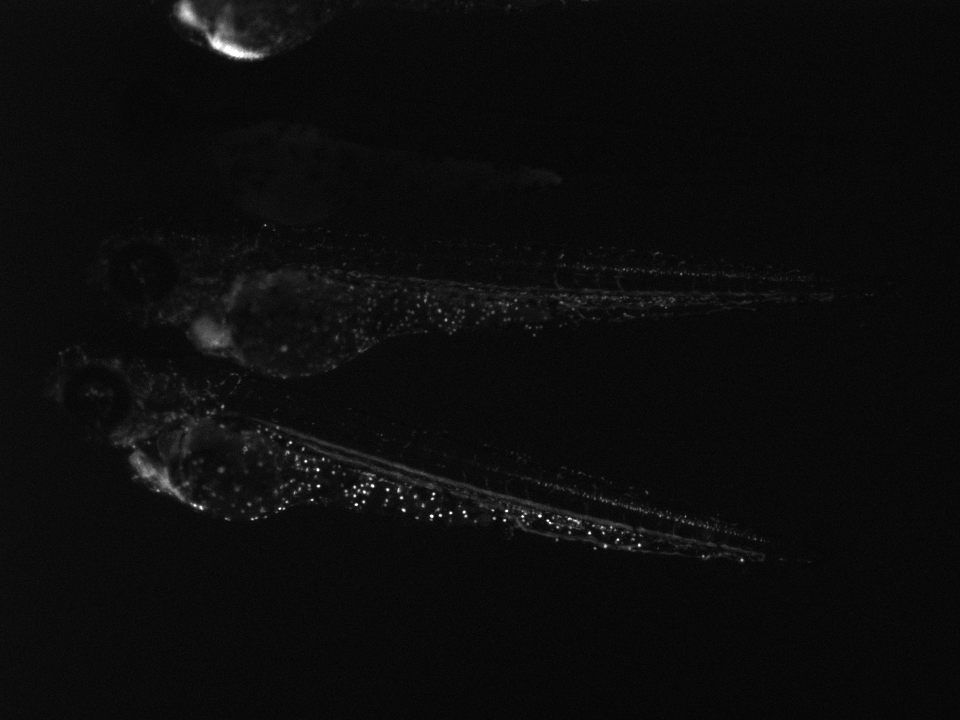

Supplement: Supplementary file 7 — Source data Fig. 3 [file 44321_2025_368_MOESM7_ESM.zip › FIGURE_3/3E/BOSUTINIB_01uM (1).tif]

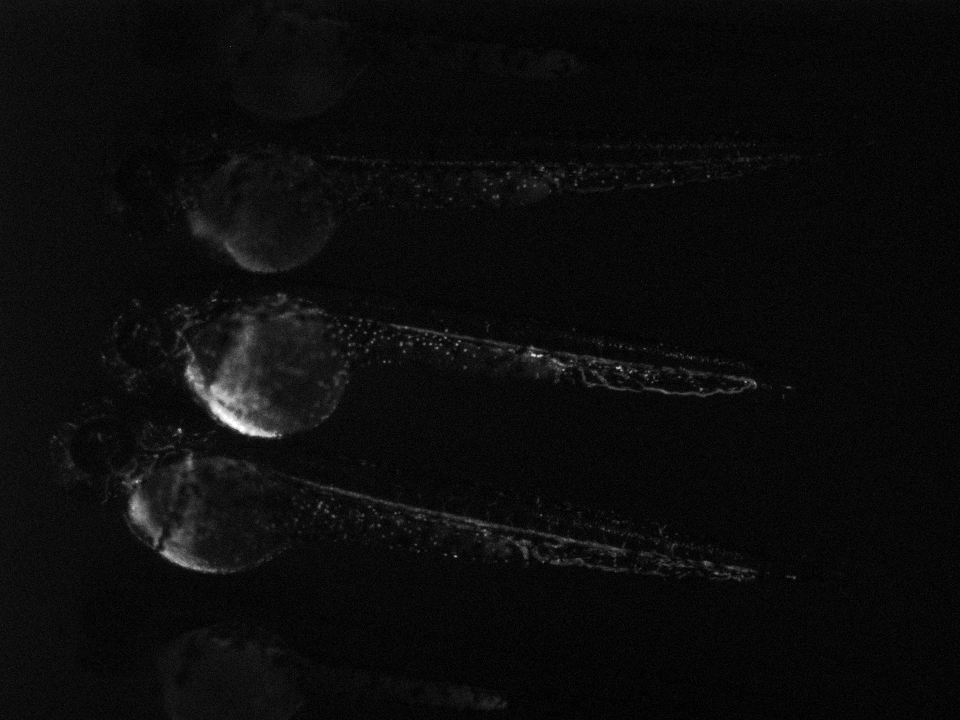

Supplement: Supplementary file 7 — Source data Fig. 3 [file 44321_2025_368_MOESM7_ESM.zip › FIGURE_3/3E/BOSUTINIB_01uM (10).tif]

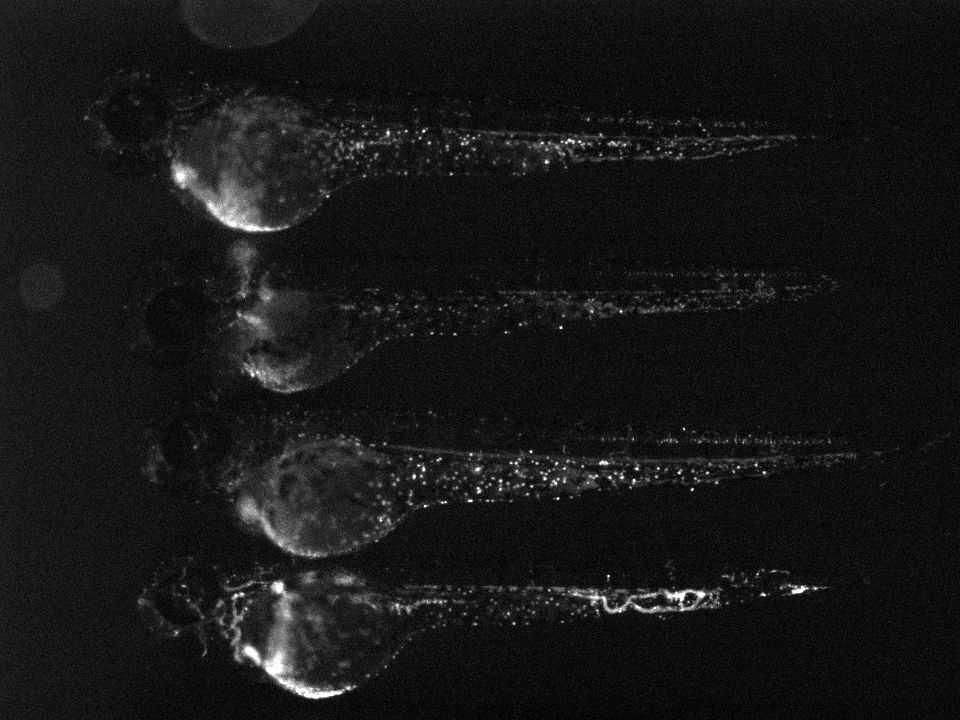

Supplement: Supplementary file 7 — Source data Fig. 3 [file 44321_2025_368_MOESM7_ESM.zip › FIGURE_3/3E/BOSUTINIB_01uM (11).tif]

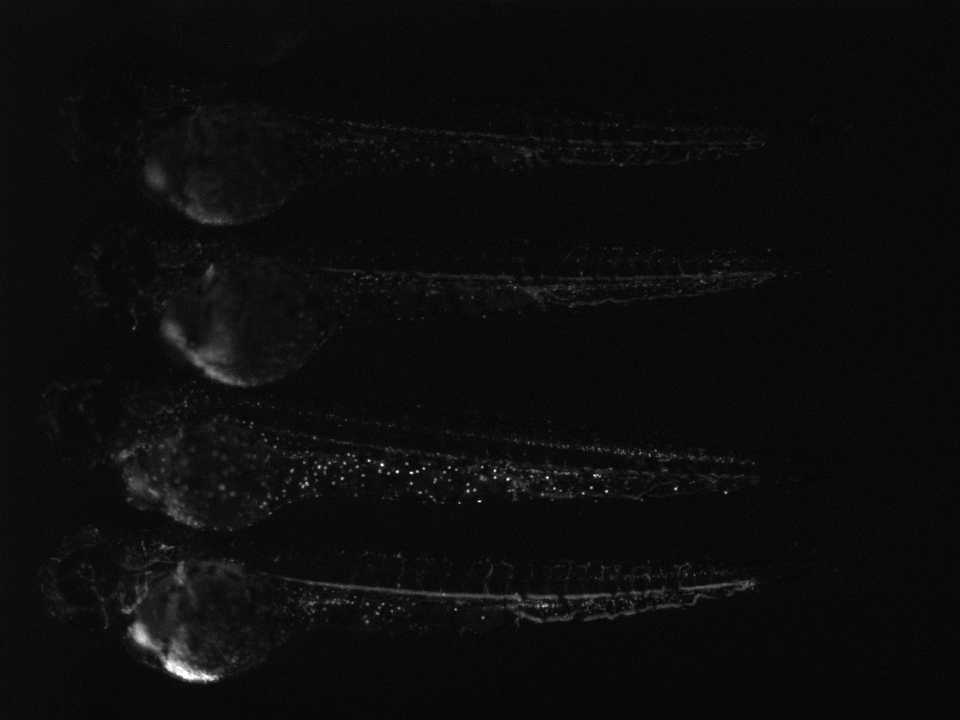

Supplement: Supplementary file 7 — Source data Fig. 3 [file 44321_2025_368_MOESM7_ESM.zip › FIGURE_3/3E/BOSUTINIB_01uM (12).tif]

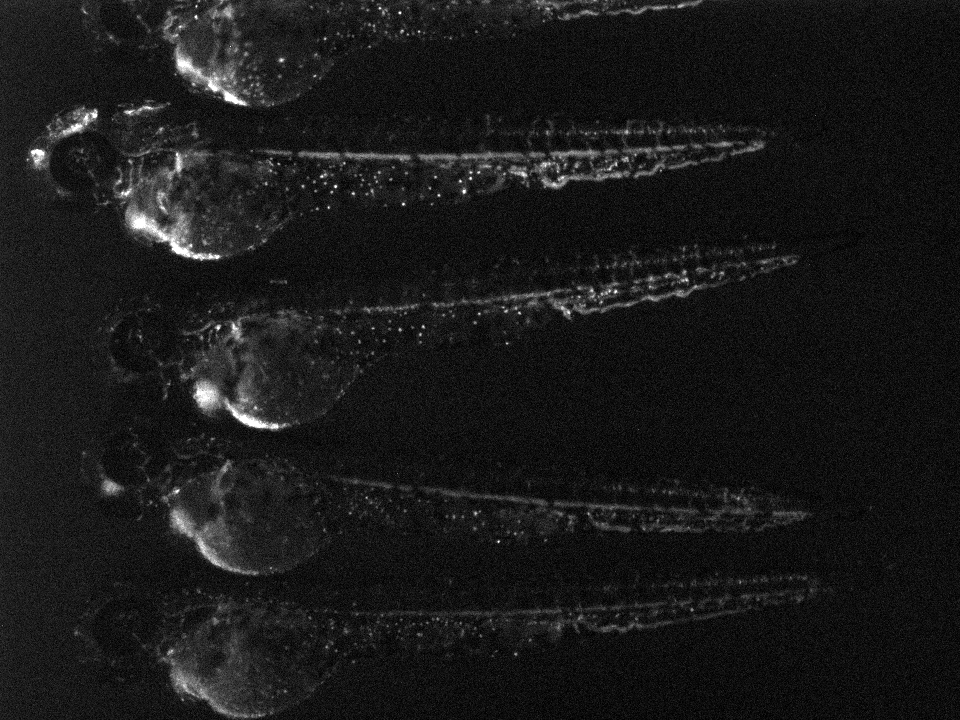

Supplement: Supplementary file 7 — Source data Fig. 3 [file 44321_2025_368_MOESM7_ESM.zip › FIGURE_3/3E/BOSUTINIB_01uM (2).tif]

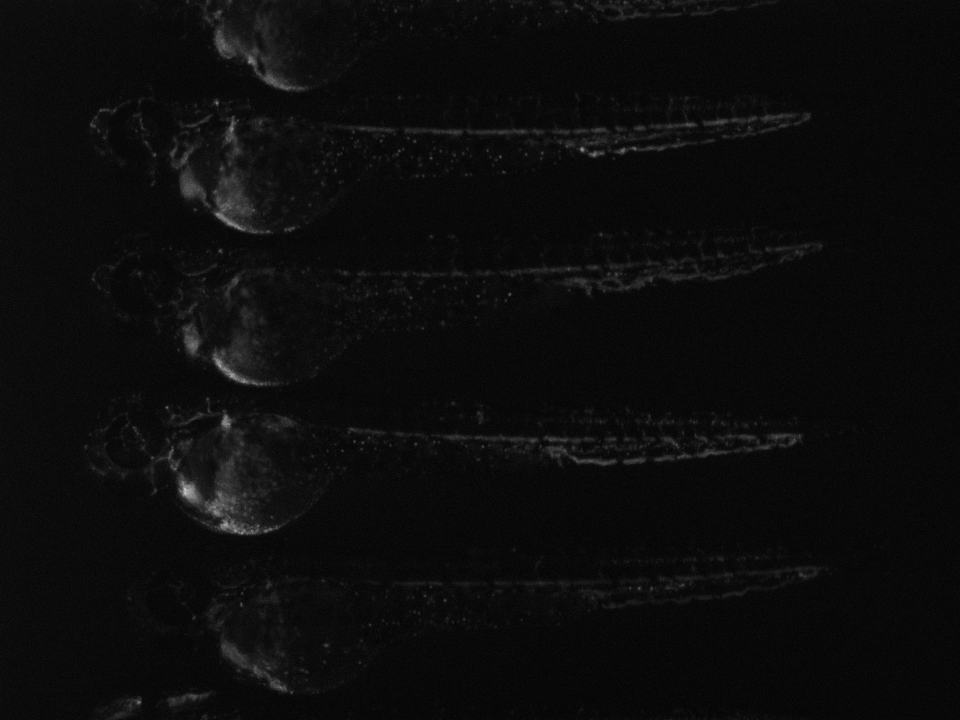

Supplement: Supplementary file 7 — Source data Fig. 3 [file 44321_2025_368_MOESM7_ESM.zip › FIGURE_3/3E/BOSUTINIB_01uM (3).tif]

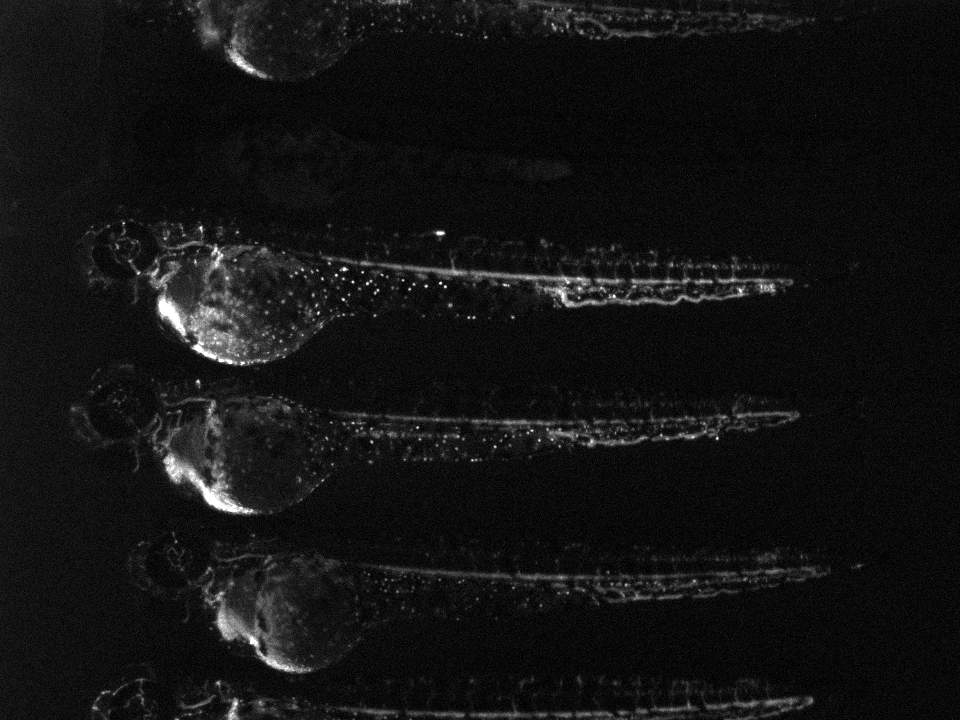

Supplement: Supplementary file 7 — Source data Fig. 3 [file 44321_2025_368_MOESM7_ESM.zip › FIGURE_3/3E/BOSUTINIB_01uM (4).tif]

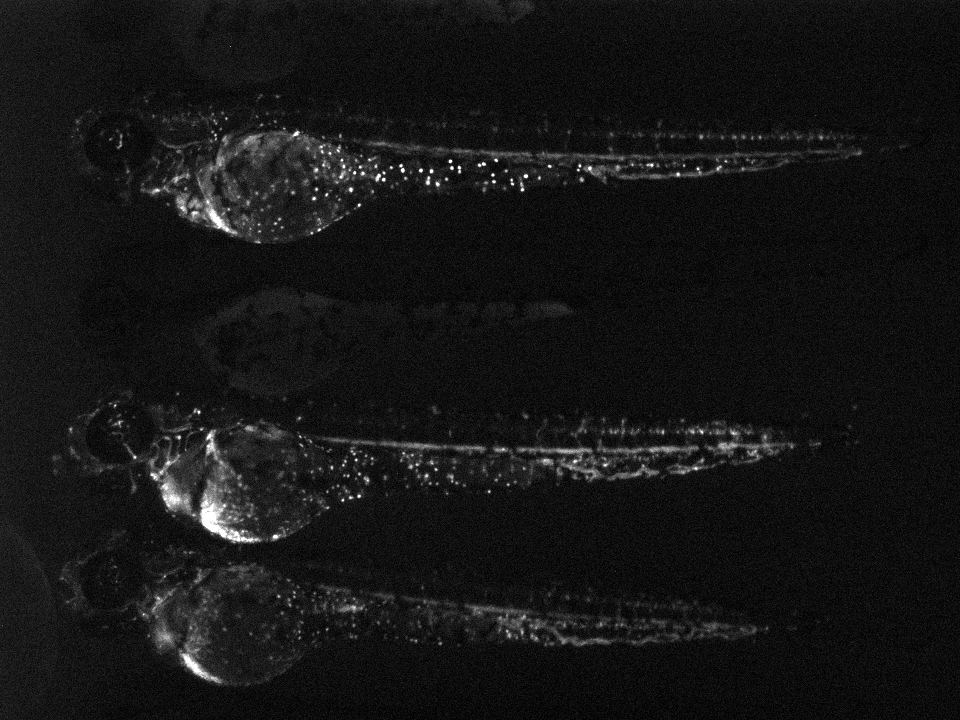

Supplement: Supplementary file 7 — Source data Fig. 3 [file 44321_2025_368_MOESM7_ESM.zip › FIGURE_3/3E/BOSUTINIB_01uM (5).tif]

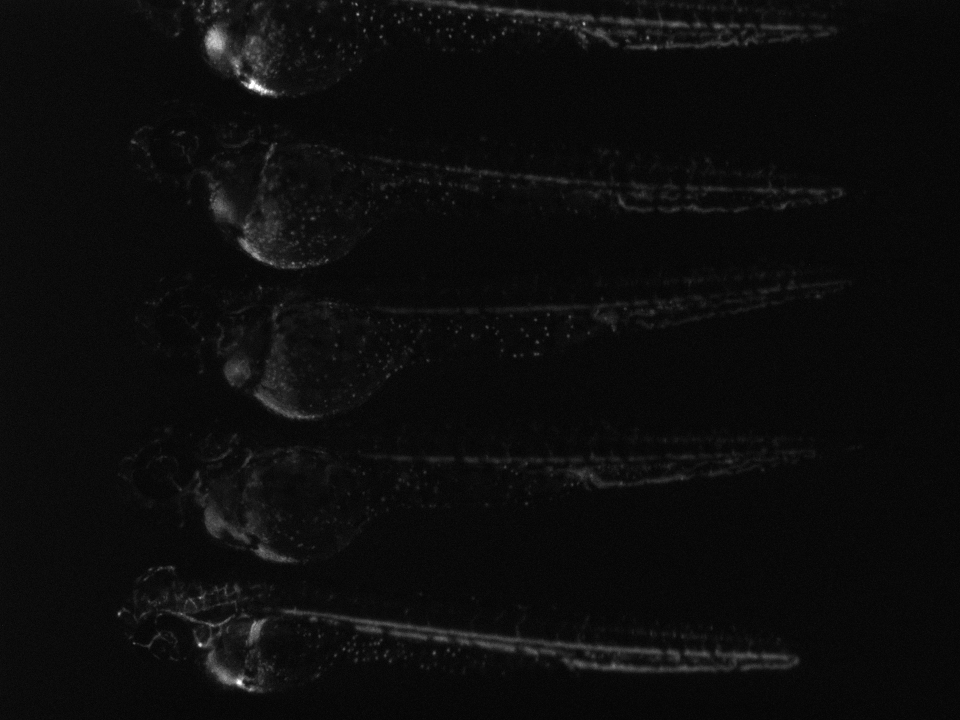

Supplement: Supplementary file 7 — Source data Fig. 3 [file 44321_2025_368_MOESM7_ESM.zip › FIGURE_3/3E/BOSUTINIB_01uM (6).tif]

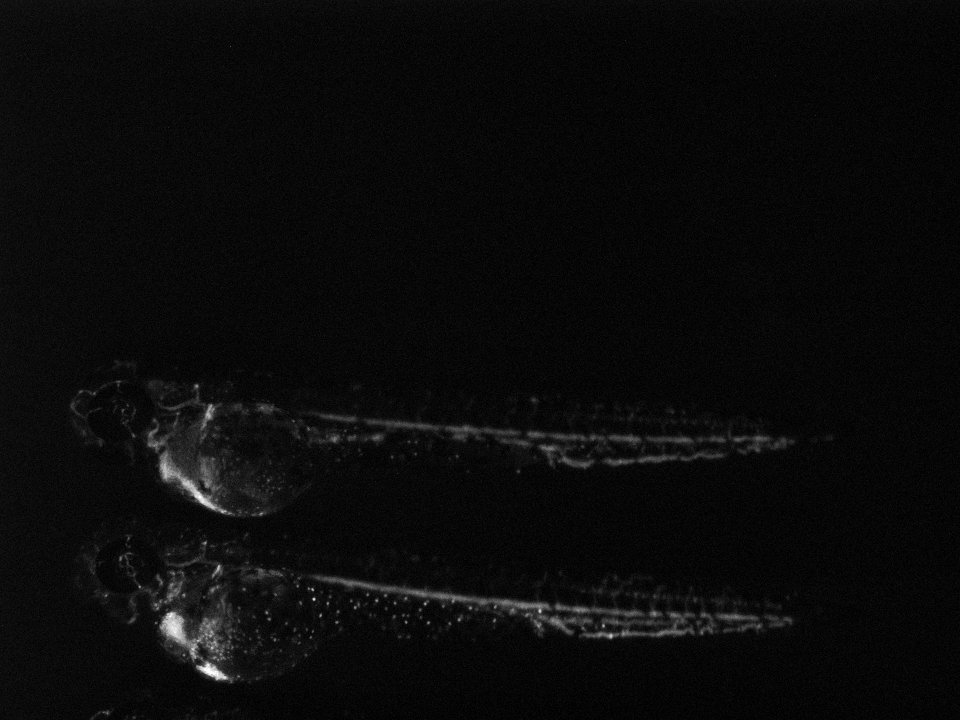

Supplement: Supplementary file 7 — Source data Fig. 3 [file 44321_2025_368_MOESM7_ESM.zip › FIGURE_3/3E/BOSUTINIB_01uM (7).tif]

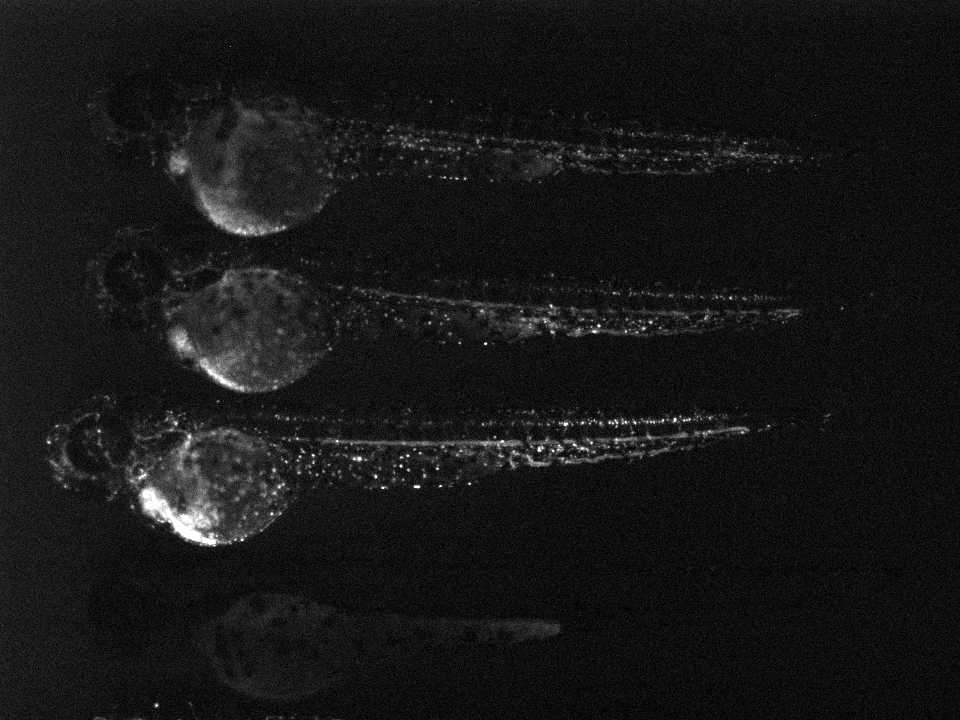

Supplement: Supplementary file 7 — Source data Fig. 3 [file 44321_2025_368_MOESM7_ESM.zip › FIGURE_3/3E/BOSUTINIB_01uM (8).tif]

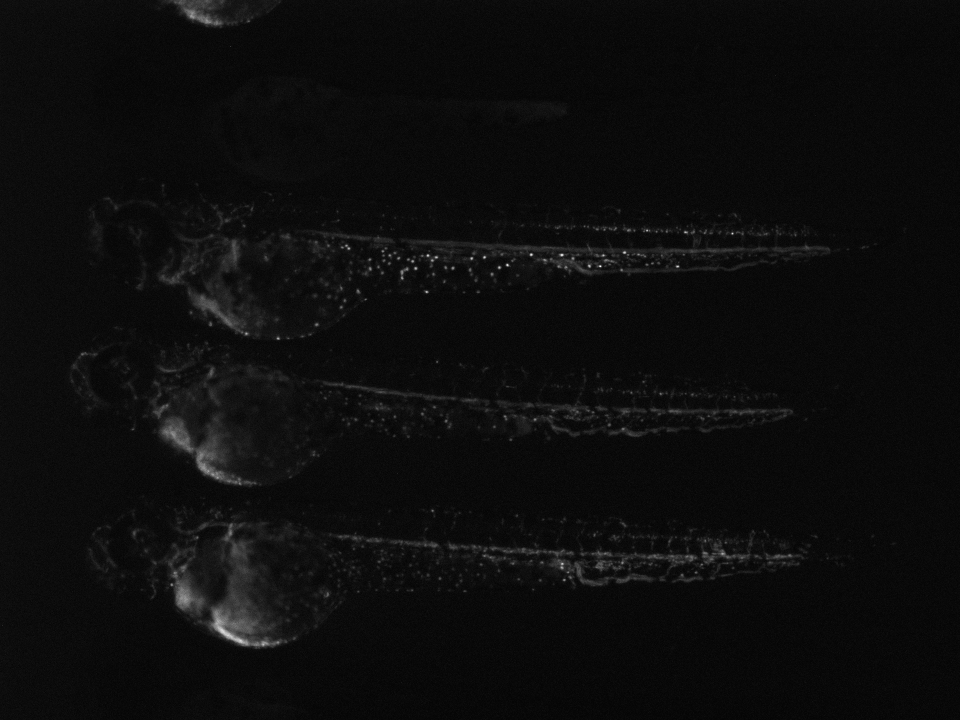

Supplement: Supplementary file 7 — Source data Fig. 3 [file 44321_2025_368_MOESM7_ESM.zip › FIGURE_3/3E/BOSUTINIB_01uM (9).tif]

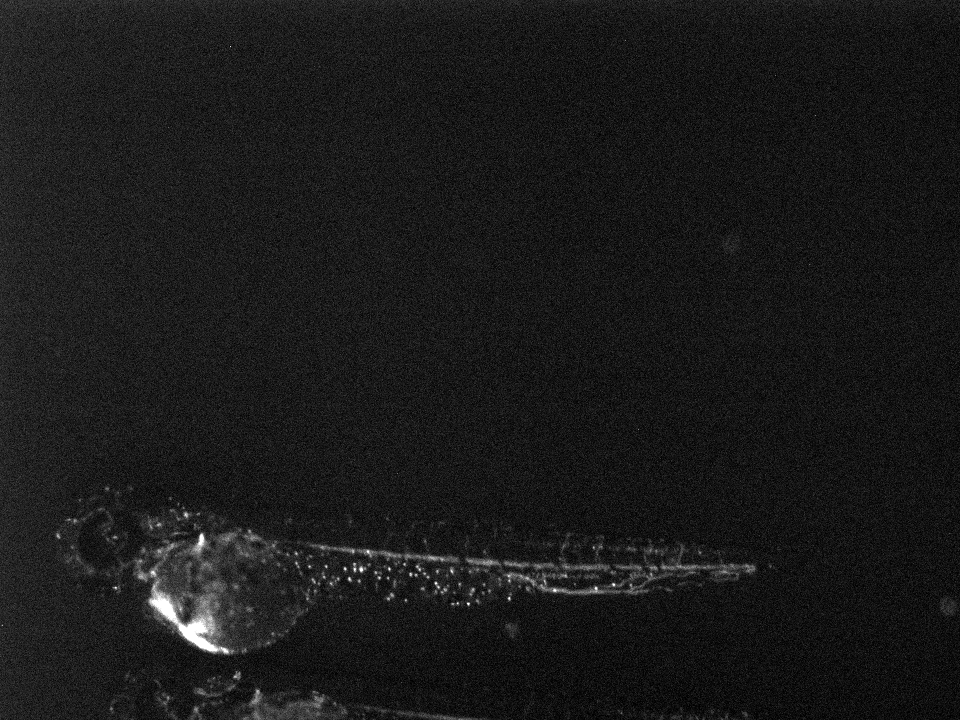

Supplement: Supplementary file 7 — Source data Fig. 3 [file 44321_2025_368_MOESM7_ESM.zip › FIGURE_3/3E/BOSUTINIB_1uM (1).tif]

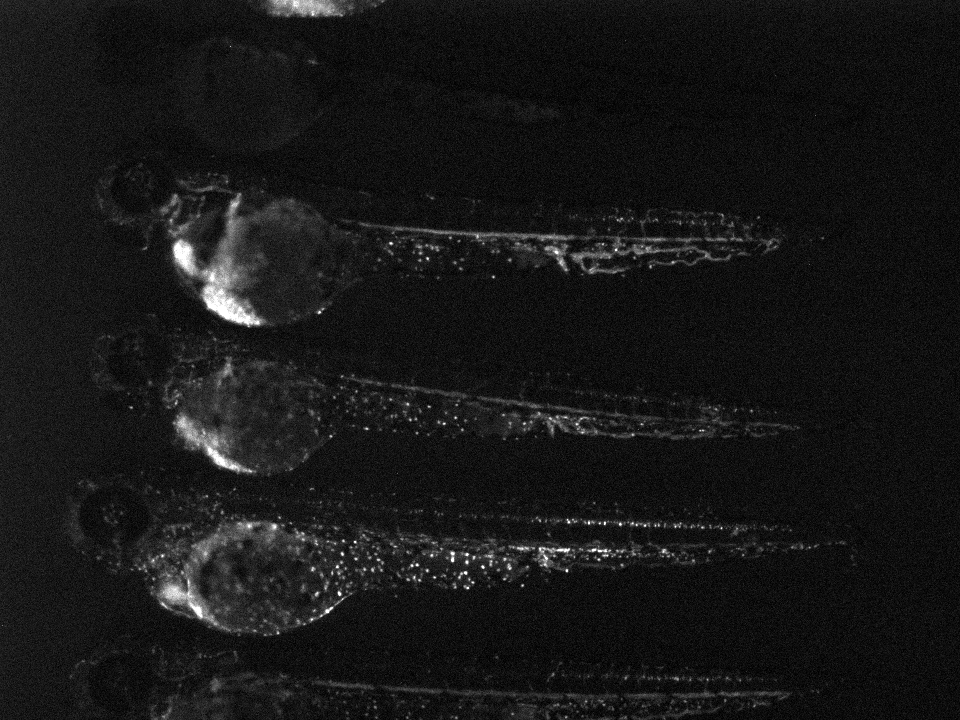

Supplement: Supplementary file 7 — Source data Fig. 3 [file 44321_2025_368_MOESM7_ESM.zip › FIGURE_3/3E/BOSUTINIB_1uM (10).tif]

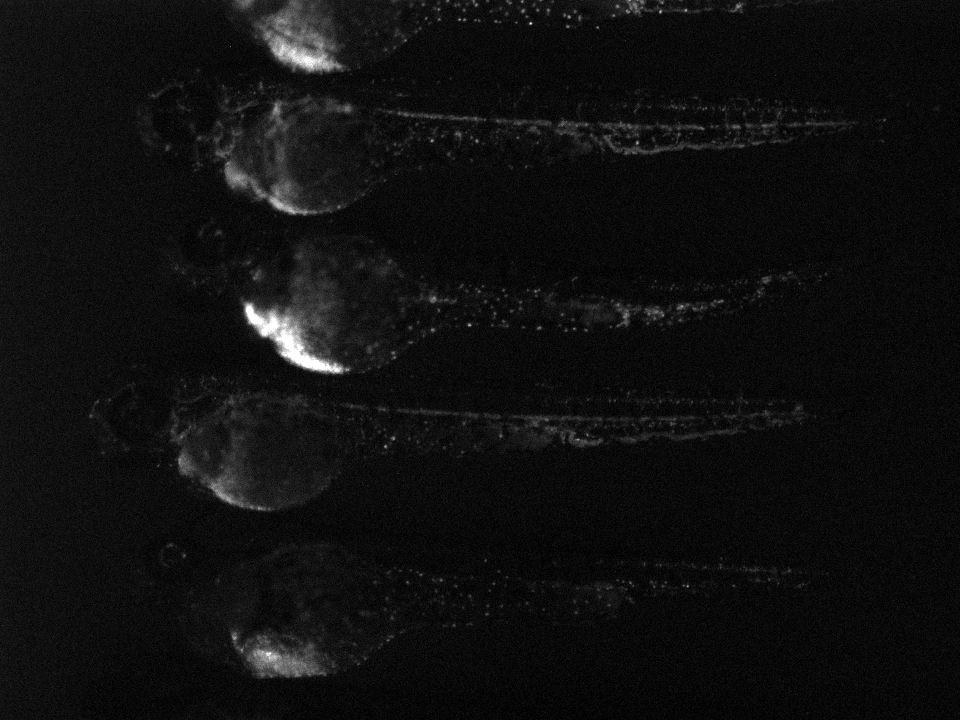

Supplement: Supplementary file 7 — Source data Fig. 3 [file 44321_2025_368_MOESM7_ESM.zip › FIGURE_3/3E/BOSUTINIB_1uM (11).tif]

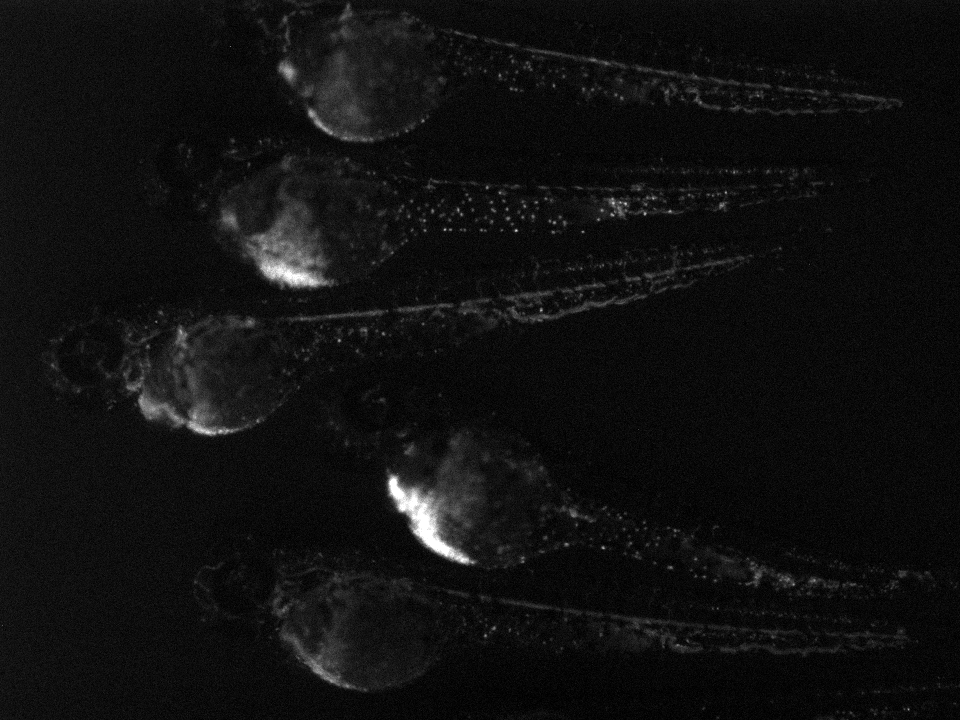

Supplement: Supplementary file 7 — Source data Fig. 3 [file 44321_2025_368_MOESM7_ESM.zip › FIGURE_3/3E/BOSUTINIB_1uM (12).tif]

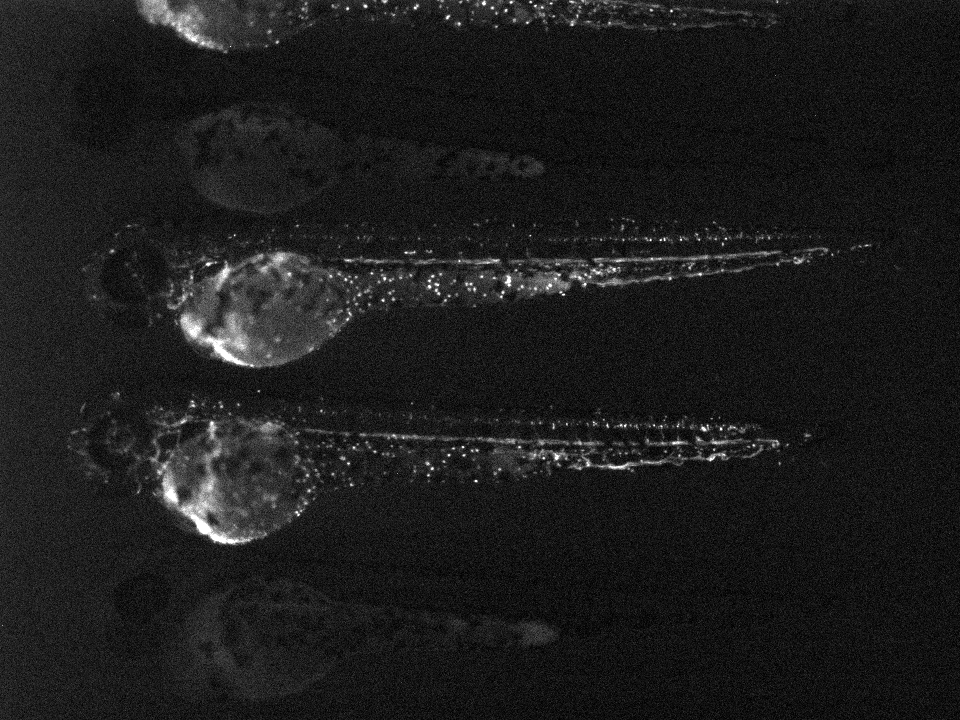

Supplement: Supplementary file 7 — Source data Fig. 3 [file 44321_2025_368_MOESM7_ESM.zip › FIGURE_3/3E/BOSUTINIB_1uM (2).tif]

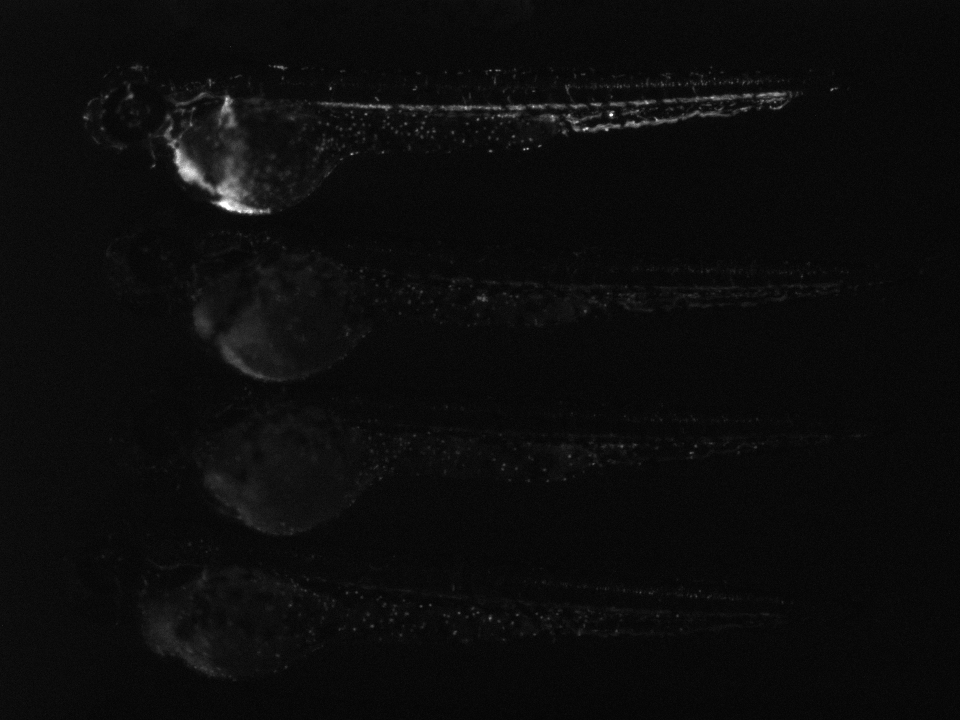

Supplement: Supplementary file 7 — Source data Fig. 3 [file 44321_2025_368_MOESM7_ESM.zip › FIGURE_3/3E/BOSUTINIB_1uM (3).tif]

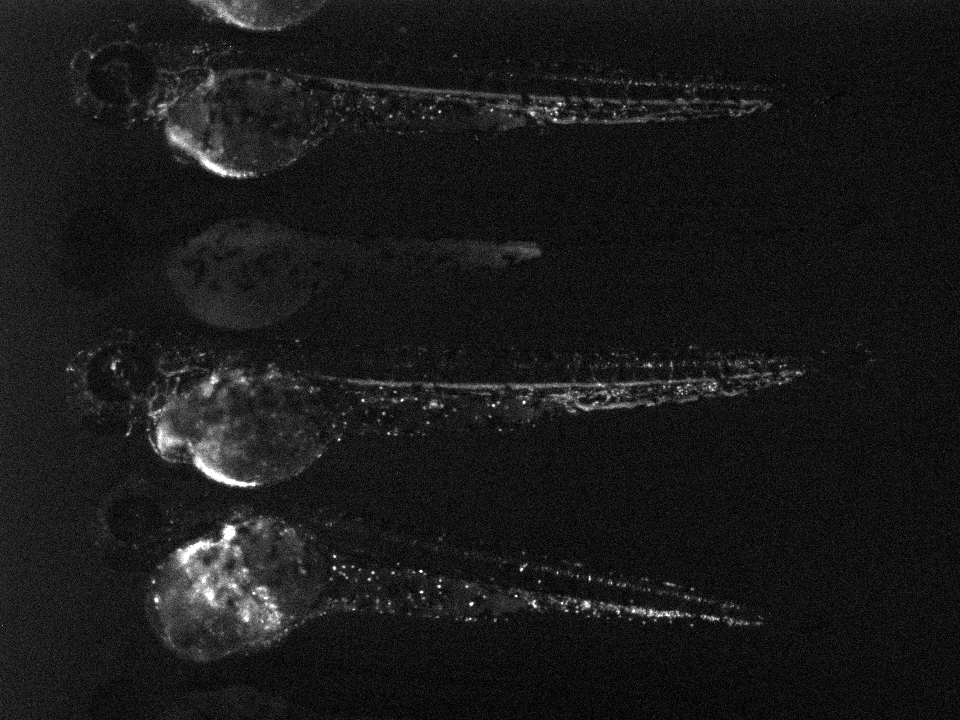

Supplement: Supplementary file 7 — Source data Fig. 3 [file 44321_2025_368_MOESM7_ESM.zip › FIGURE_3/3E/BOSUTINIB_1uM (4).tif]

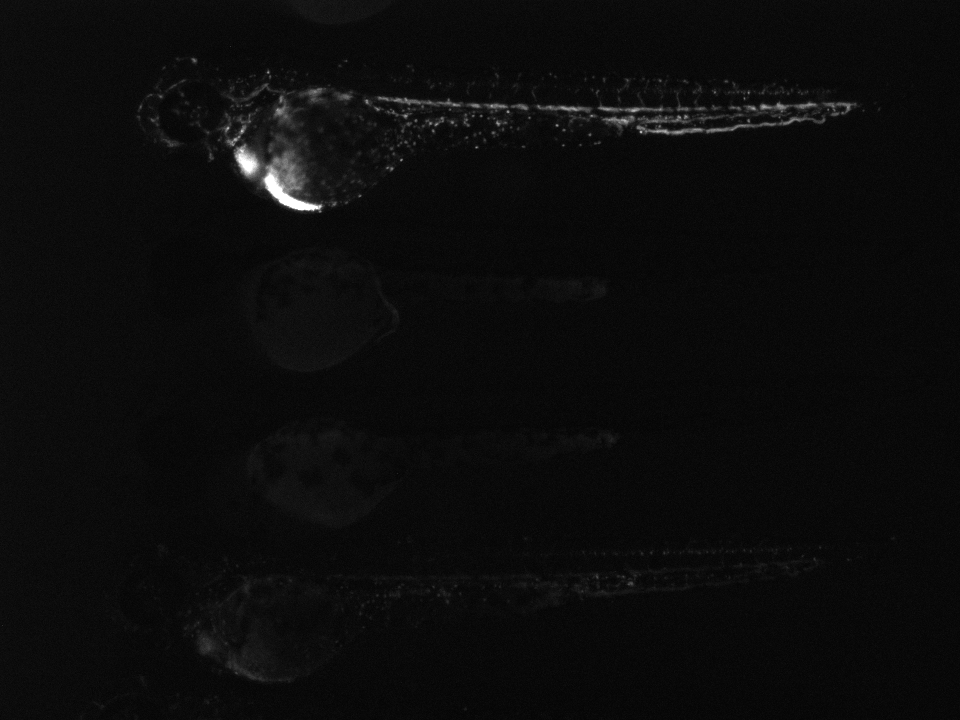

Supplement: Supplementary file 7 — Source data Fig. 3 [file 44321_2025_368_MOESM7_ESM.zip › FIGURE_3/3E/BOSUTINIB_1uM (5).tif]

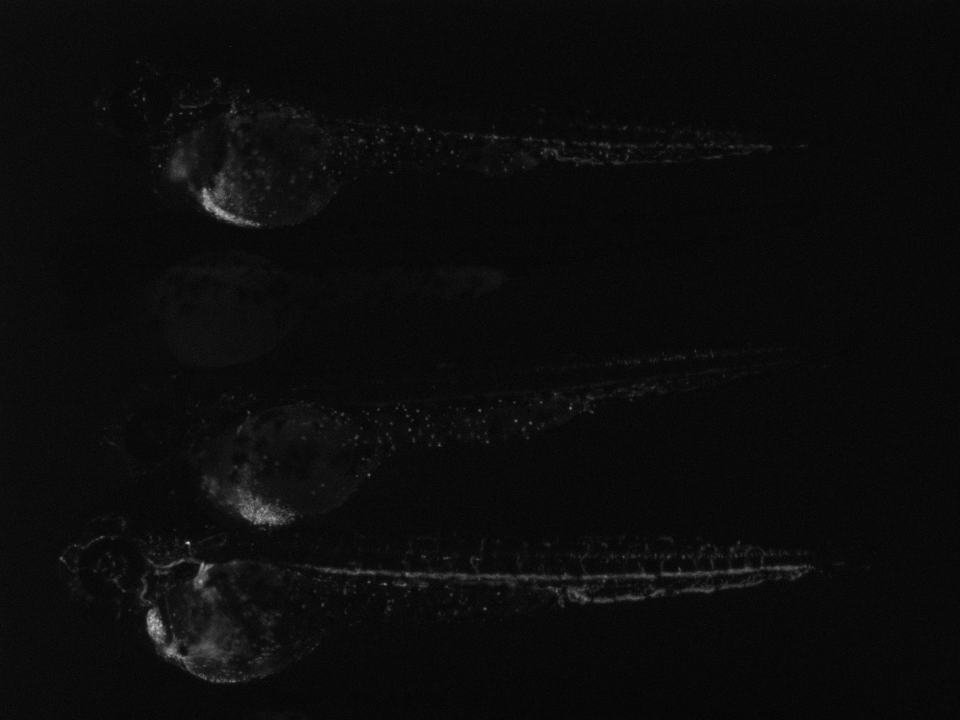

Supplement: Supplementary file 7 — Source data Fig. 3 [file 44321_2025_368_MOESM7_ESM.zip › FIGURE_3/3E/BOSUTINIB_1uM (6).tif]

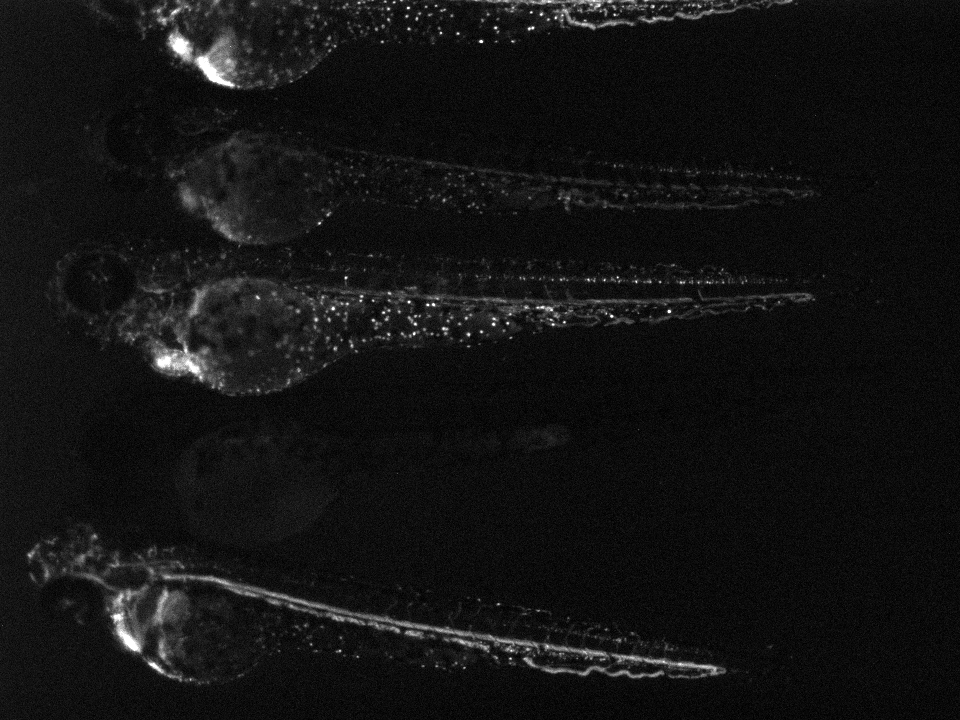

Supplement: Supplementary file 7 — Source data Fig. 3 [file 44321_2025_368_MOESM7_ESM.zip › FIGURE_3/3E/BOSUTINIB_1uM (7).tif]

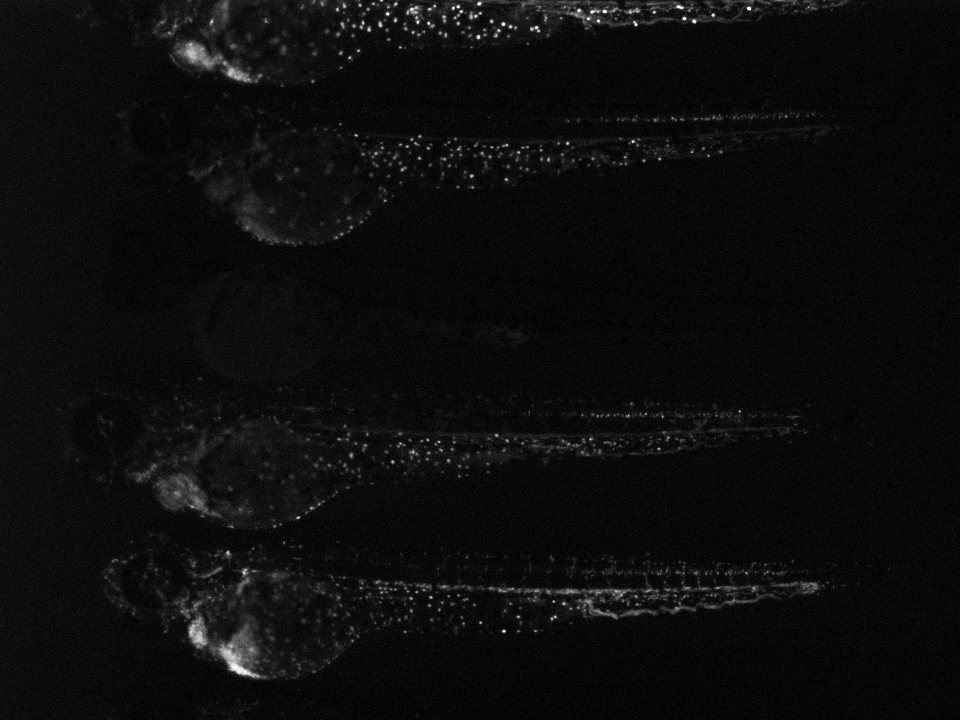

Supplement: Supplementary file 7 — Source data Fig. 3 [file 44321_2025_368_MOESM7_ESM.zip › FIGURE_3/3E/BOSUTINIB_1uM (8).tif]

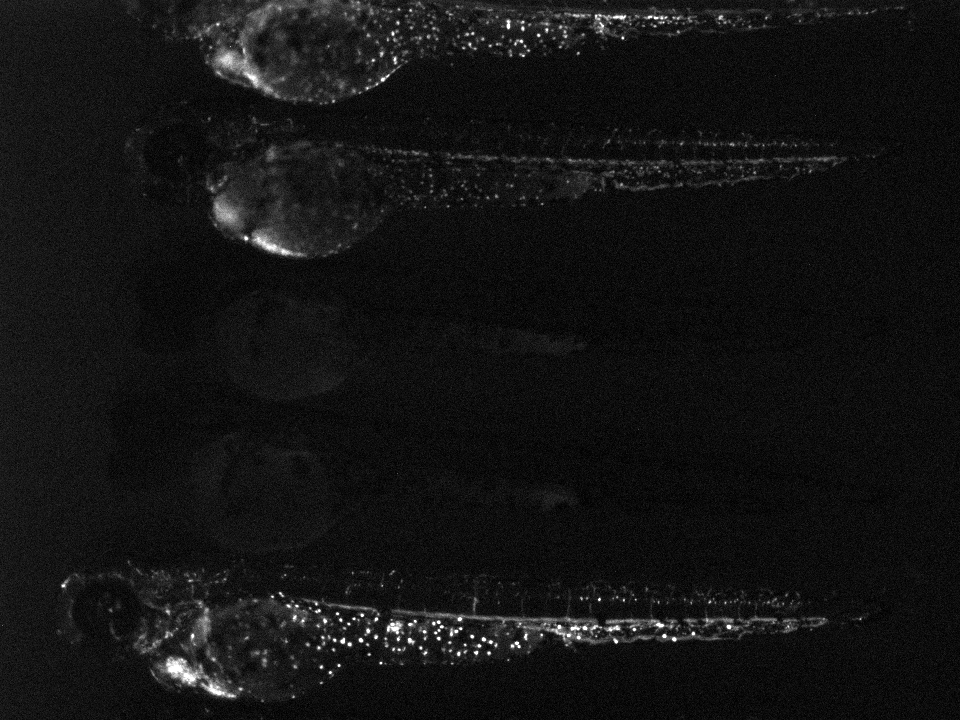

Supplement: Supplementary file 7 — Source data Fig. 3 [file 44321_2025_368_MOESM7_ESM.zip › FIGURE_3/3E/BOSUTINIB_1uM (9).tif]

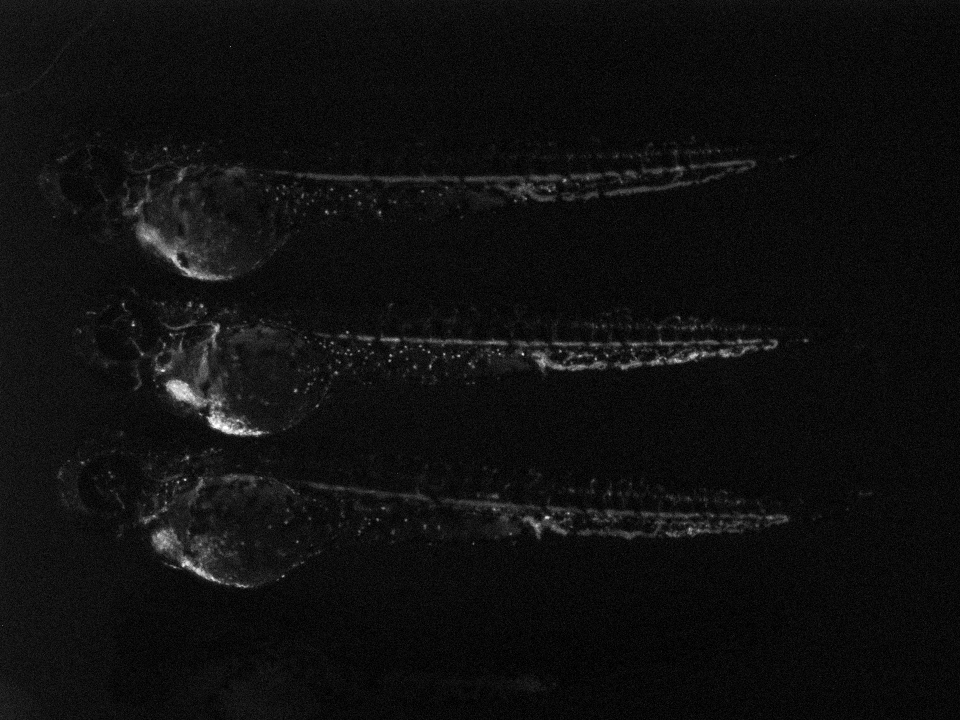

Supplement: Supplementary file 7 — Source data Fig. 3 [file 44321_2025_368_MOESM7_ESM.zip › FIGURE_3/3E/DMSO (1).tif]

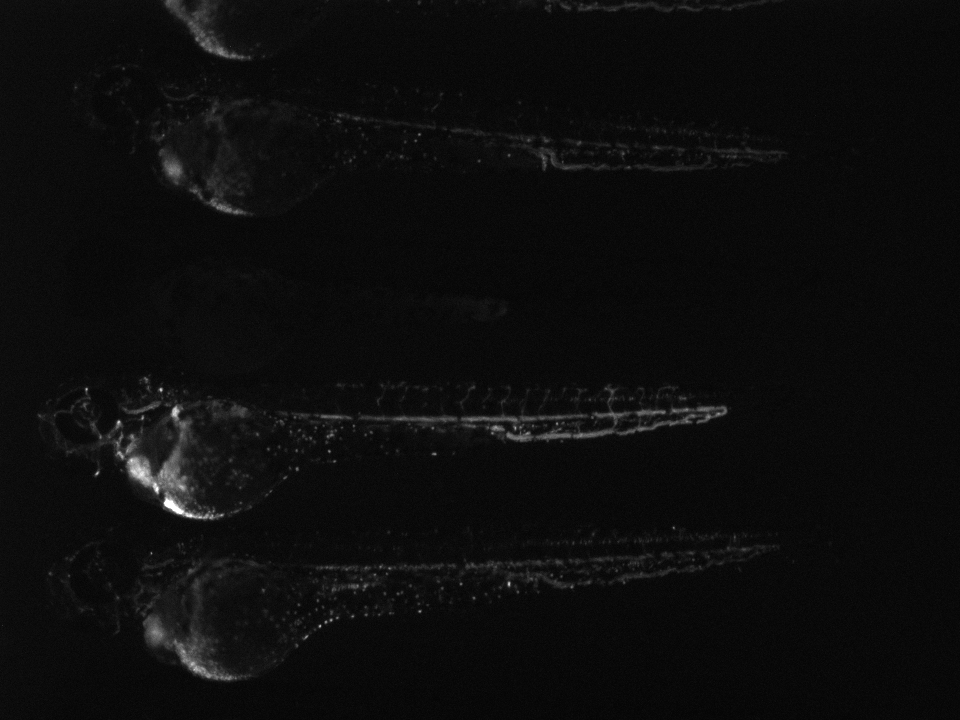

Supplement: Supplementary file 7 — Source data Fig. 3 [file 44321_2025_368_MOESM7_ESM.zip › FIGURE_3/3E/DMSO (10).tif]

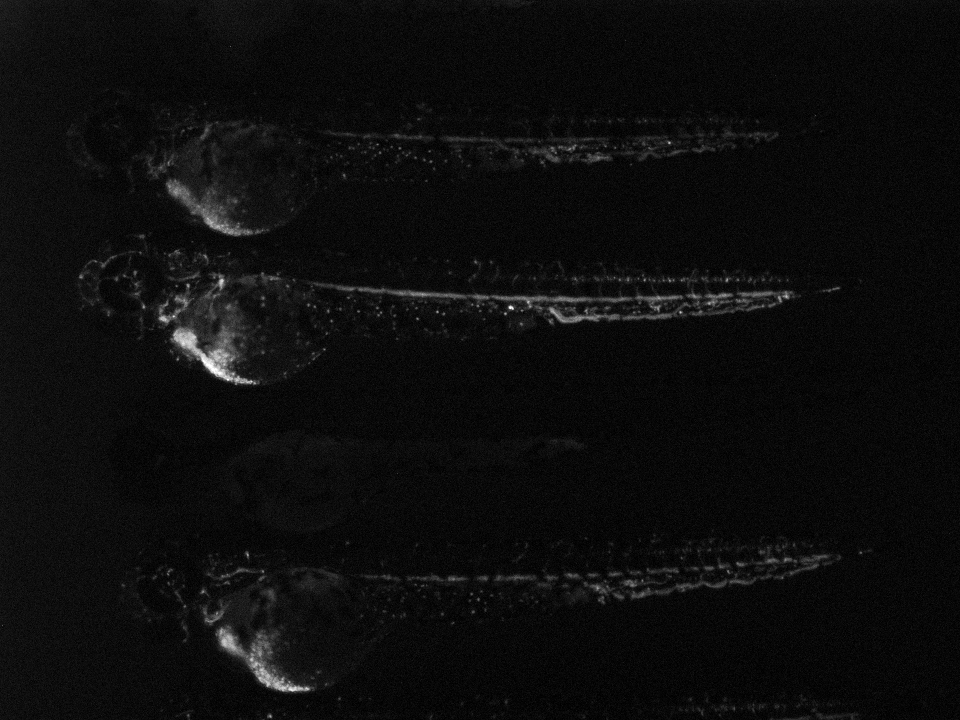

Supplement: Supplementary file 7 — Source data Fig. 3 [file 44321_2025_368_MOESM7_ESM.zip › FIGURE_3/3E/DMSO (11).tif]

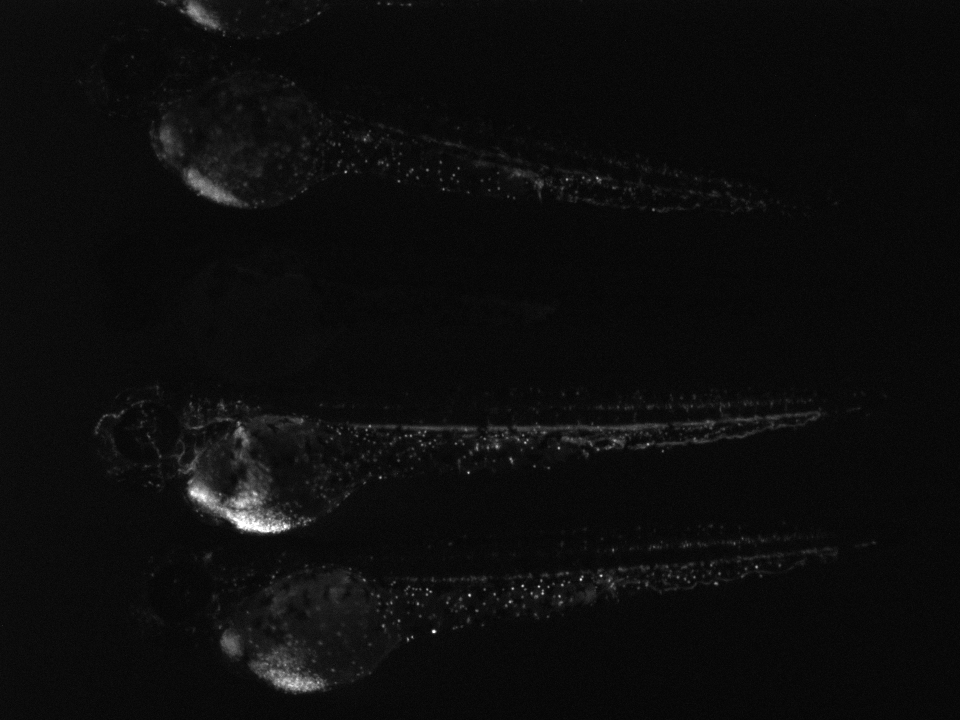

Supplement: Supplementary file 7 — Source data Fig. 3 [file 44321_2025_368_MOESM7_ESM.zip › FIGURE_3/3E/DMSO (2).tif]

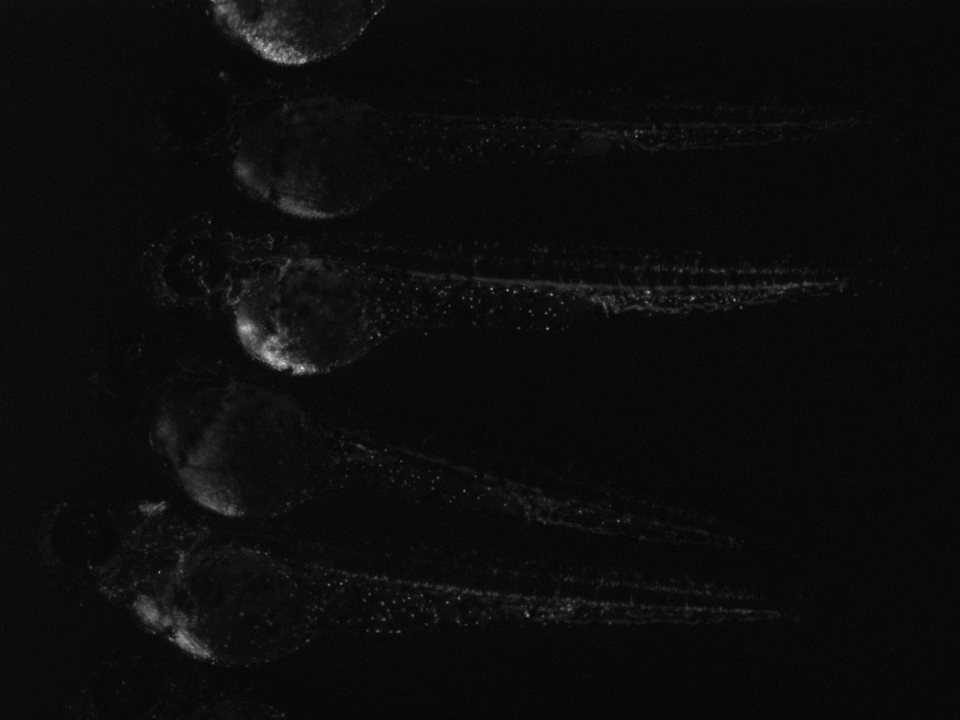

Supplement: Supplementary file 7 — Source data Fig. 3 [file 44321_2025_368_MOESM7_ESM.zip › FIGURE_3/3E/DMSO (3).tif]

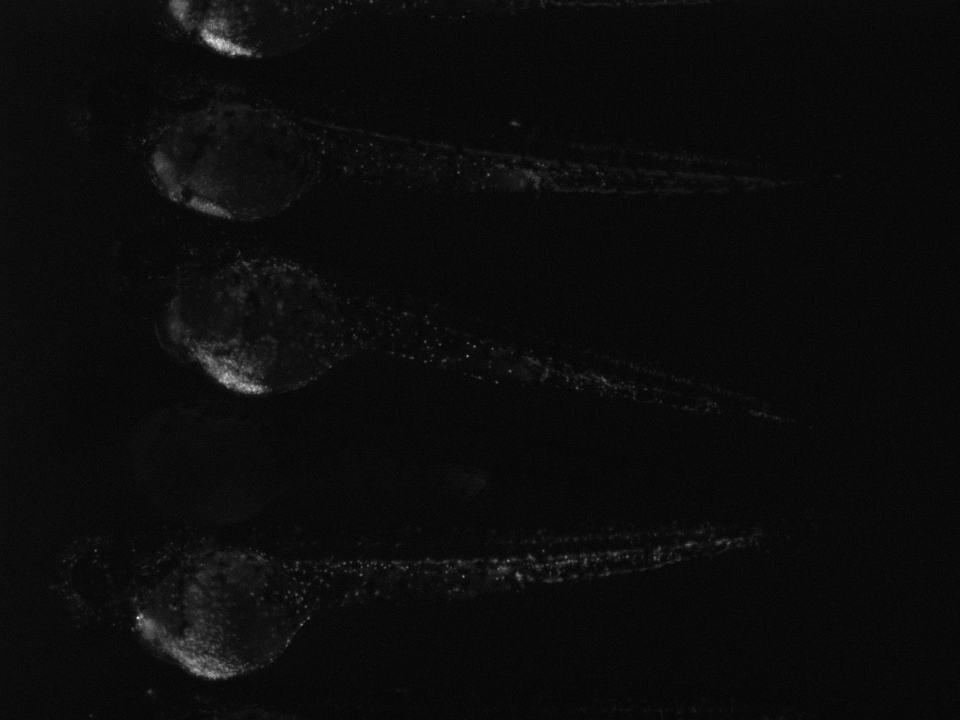

Supplement: Supplementary file 7 — Source data Fig. 3 [file 44321_2025_368_MOESM7_ESM.zip › FIGURE_3/3E/DMSO (4).tif]

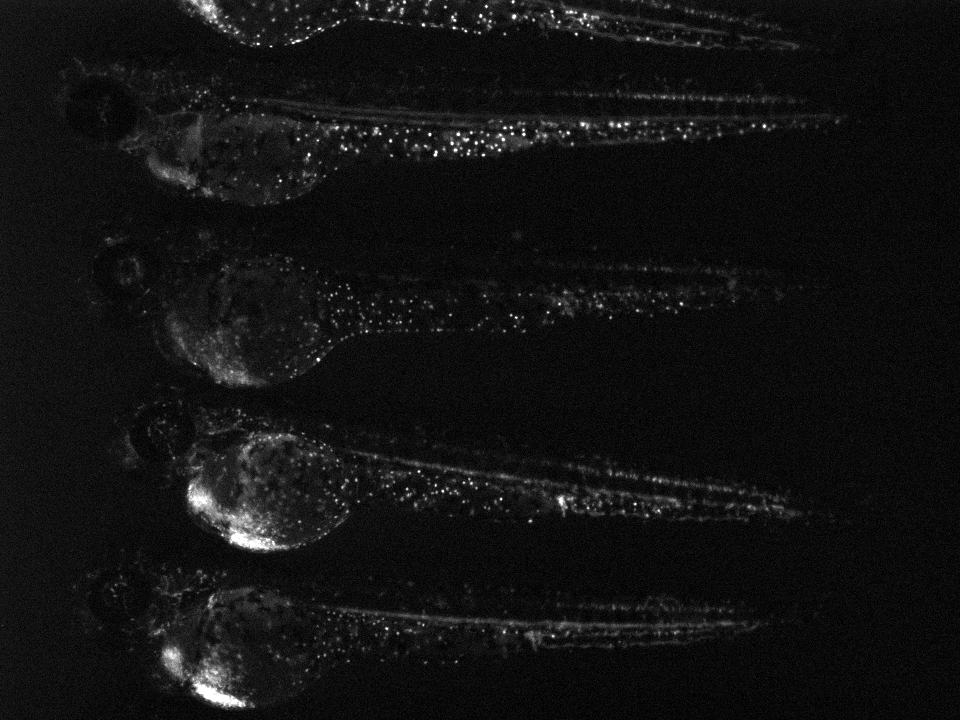

Supplement: Supplementary file 7 — Source data Fig. 3 [file 44321_2025_368_MOESM7_ESM.zip › FIGURE_3/3E/DMSO (5).tif]

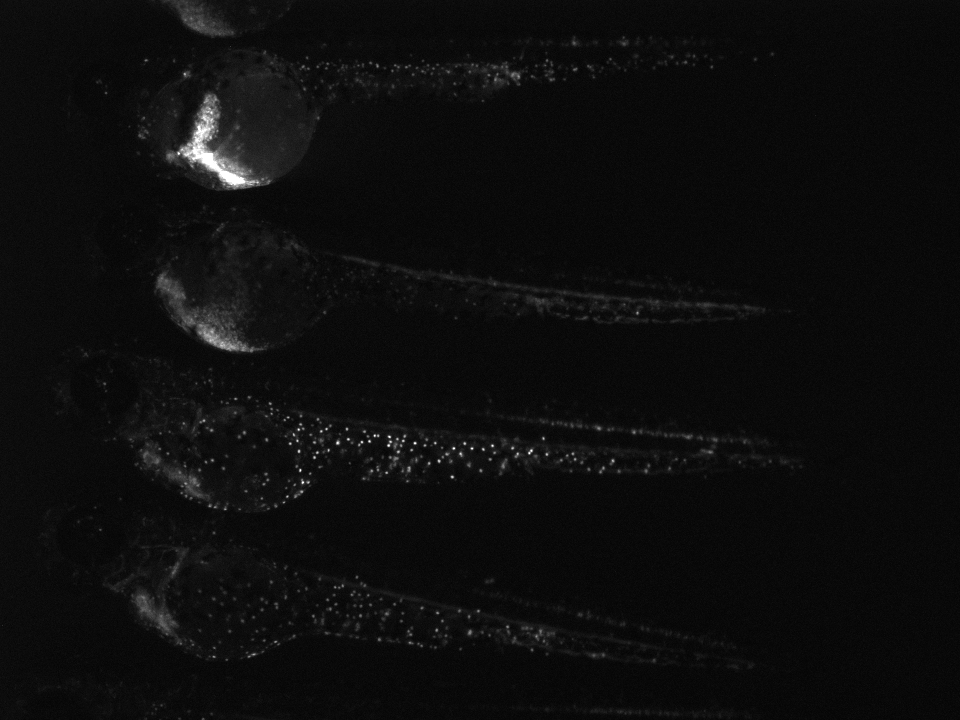

Supplement: Supplementary file 7 — Source data Fig. 3 [file 44321_2025_368_MOESM7_ESM.zip › FIGURE_3/3E/DMSO (6).tif]

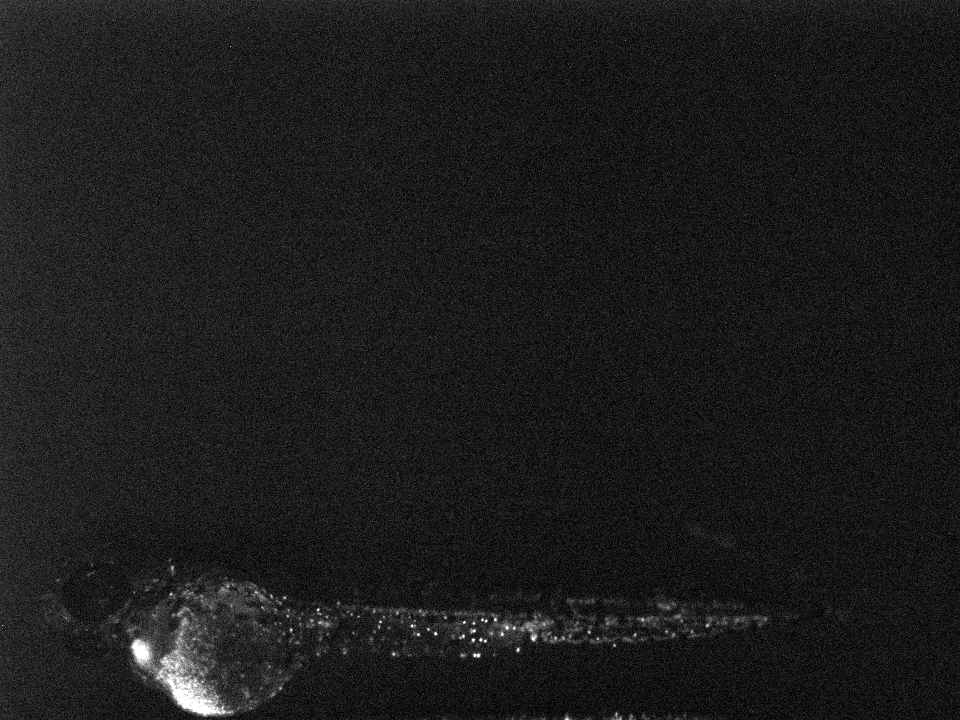

Supplement: Supplementary file 7 — Source data Fig. 3 [file 44321_2025_368_MOESM7_ESM.zip › FIGURE_3/3E/DMSO (7).tif]

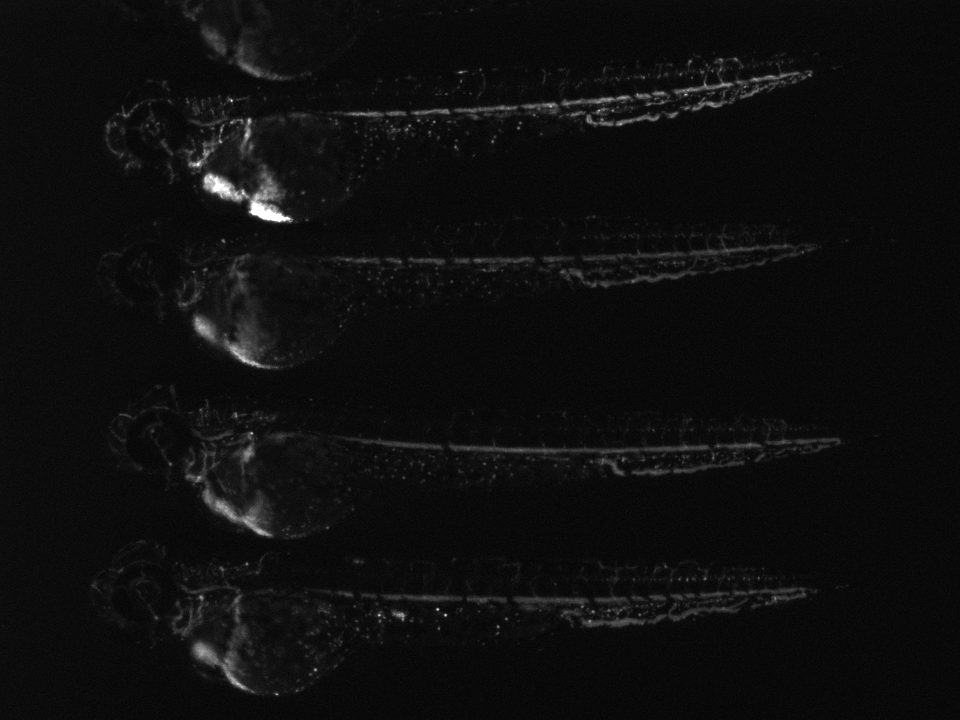

Supplement: Supplementary file 7 — Source data Fig. 3 [file 44321_2025_368_MOESM7_ESM.zip › FIGURE_3/3E/DMSO (8).tif]
