# Supplementary figures and images for: TKI-mediated inhibition of NLRP1 inflammasome restores erythropoiesis in DBA syndrome (part 4 of 4)
Source: EMBO Mol Med. 2026 Jan 9;18(2):702–24. doi: 10.1038/s44321-025-00368-3 (PMC12905221; doi:10.1038/s44321-025-00368-3)

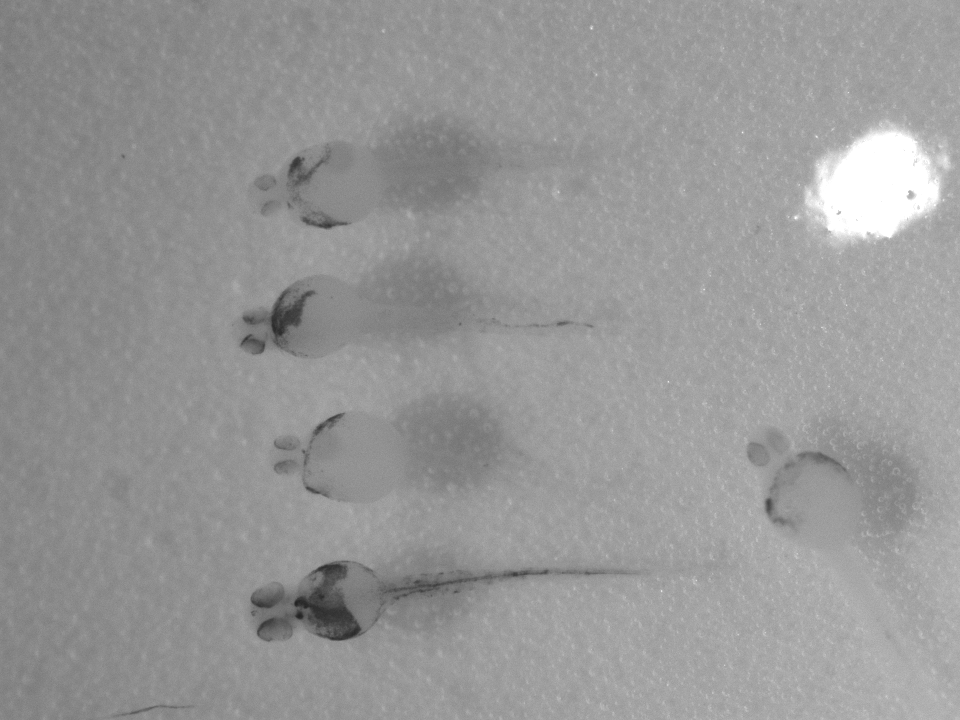

Supplement: Supplementary file 8 — Source data Fig. 4 [file 44321_2025_368_MOESM8_ESM.zip › FIGURE_4/4G/crRNA_rps19_nilotinib_1uM (3).tif]

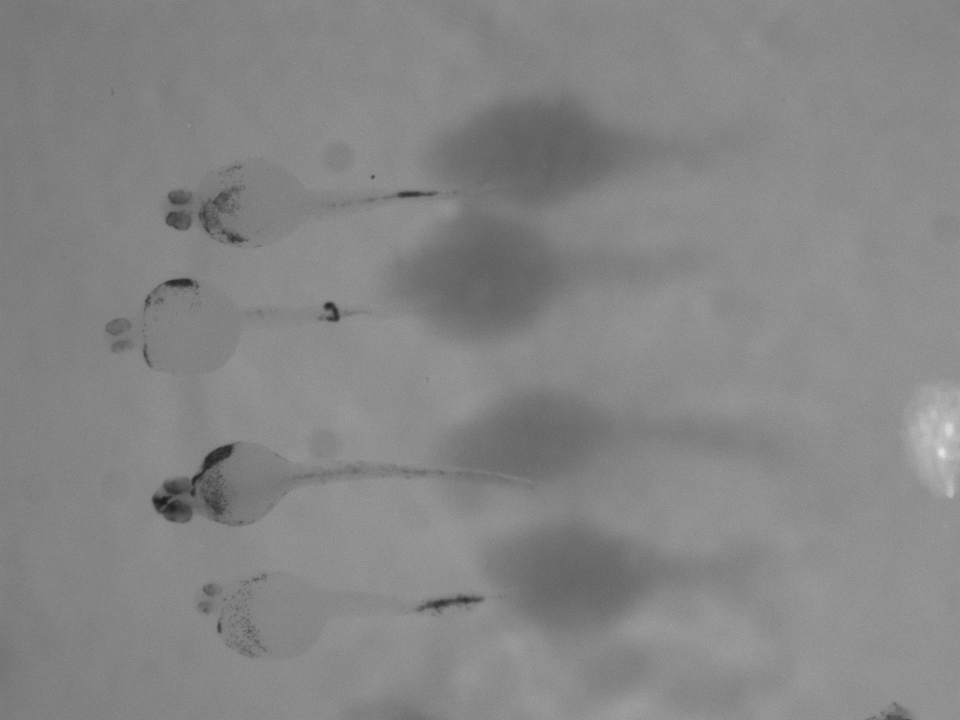

Supplement: Supplementary file 8 — Source data Fig. 4 [file 44321_2025_368_MOESM8_ESM.zip › FIGURE_4/4G/crRNA_rps19_nilotinib_1uM (4).tif]

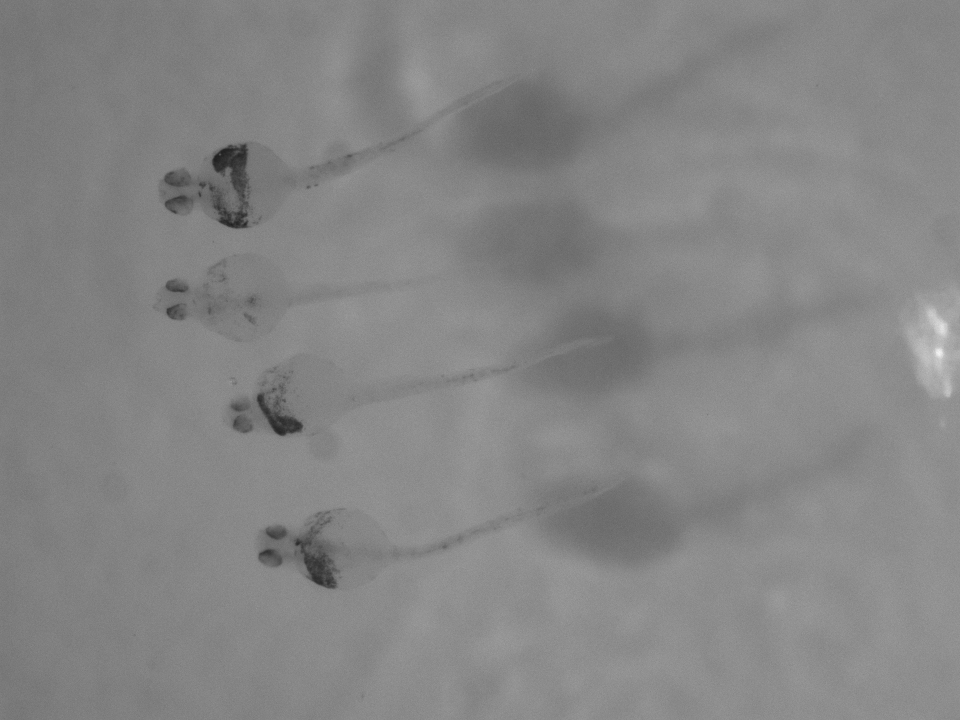

Supplement: Supplementary file 8 — Source data Fig. 4 [file 44321_2025_368_MOESM8_ESM.zip › FIGURE_4/4G/crRNA_rps19_nilotinib_1uM (5).tif]

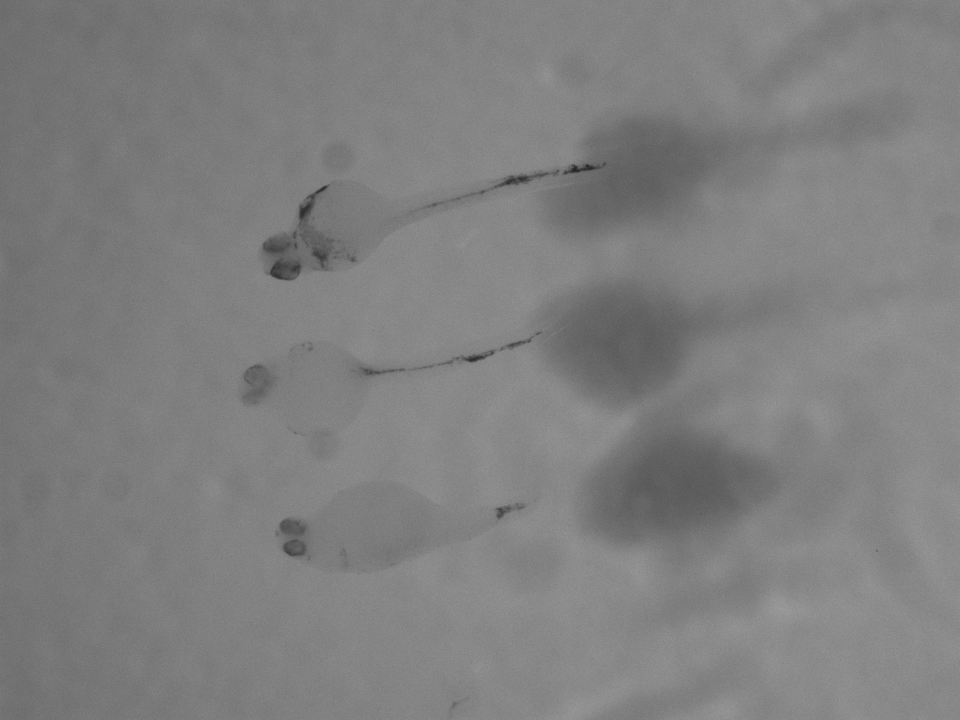

Supplement: Supplementary file 8 — Source data Fig. 4 [file 44321_2025_368_MOESM8_ESM.zip › FIGURE_4/4G/crRNA_rps19_ponatinib_1uM (1).tif]

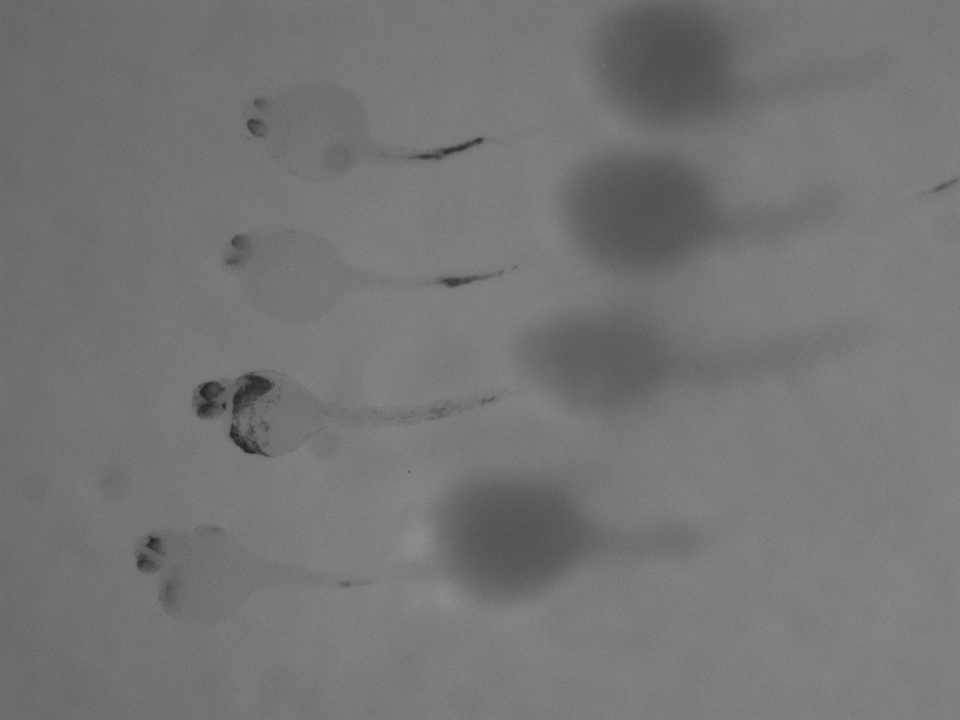

Supplement: Supplementary file 8 — Source data Fig. 4 [file 44321_2025_368_MOESM8_ESM.zip › FIGURE_4/4G/crRNA_rps19_ponatinib_1uM (2).tif]

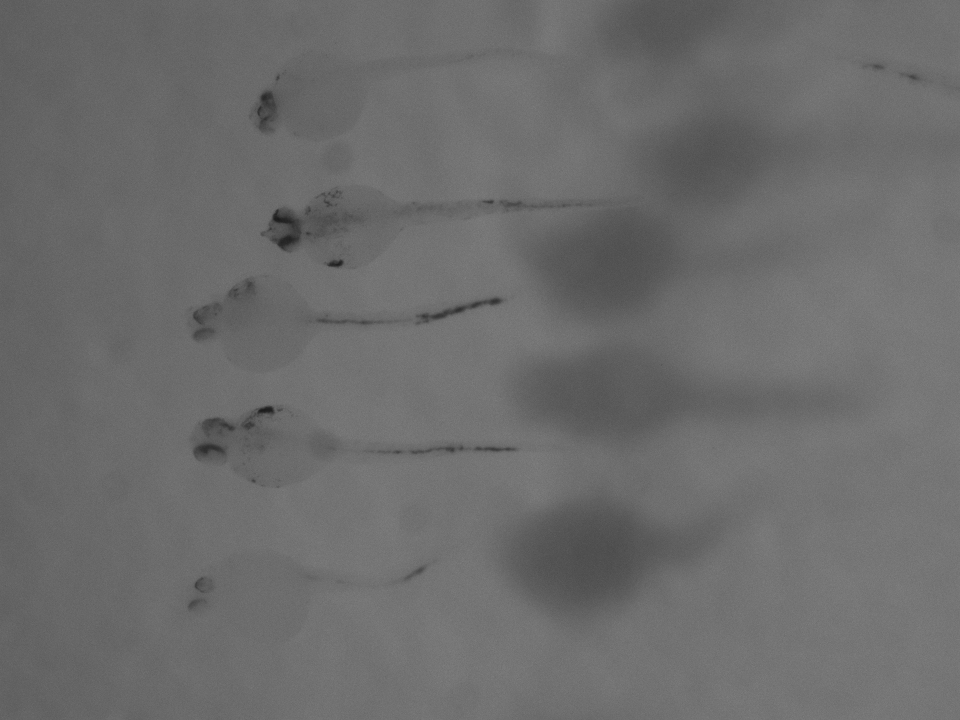

Supplement: Supplementary file 8 — Source data Fig. 4 [file 44321_2025_368_MOESM8_ESM.zip › FIGURE_4/4G/crRNA_rps19_ponatinib_1uM (3).tif]

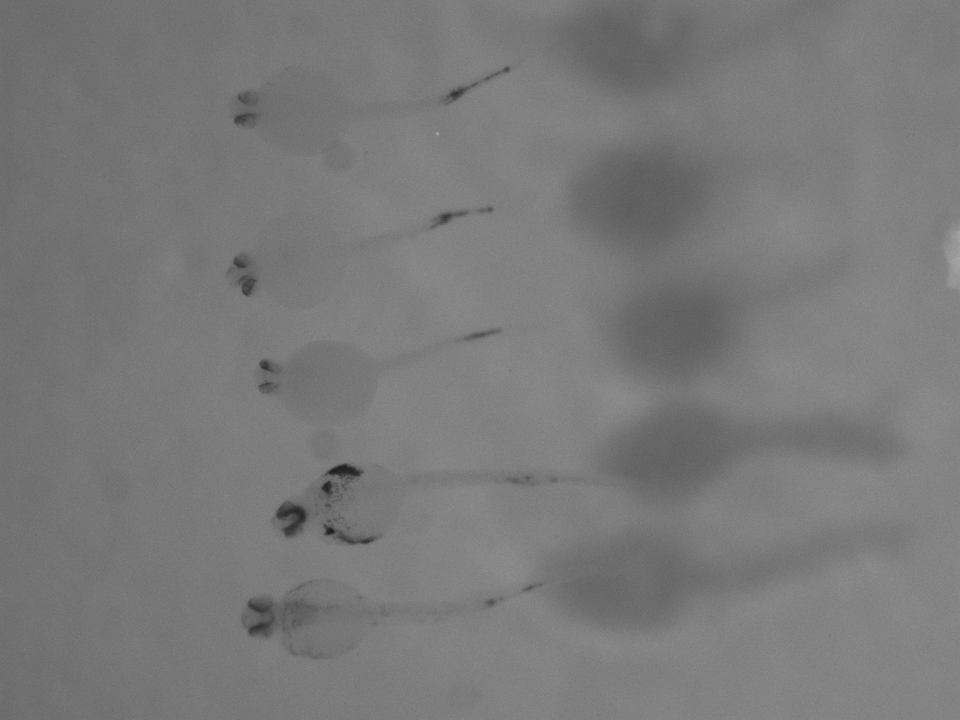

Supplement: Supplementary file 8 — Source data Fig. 4 [file 44321_2025_368_MOESM8_ESM.zip › FIGURE_4/4G/crRNA_rps19_ponatinib_1uM (4).tif]

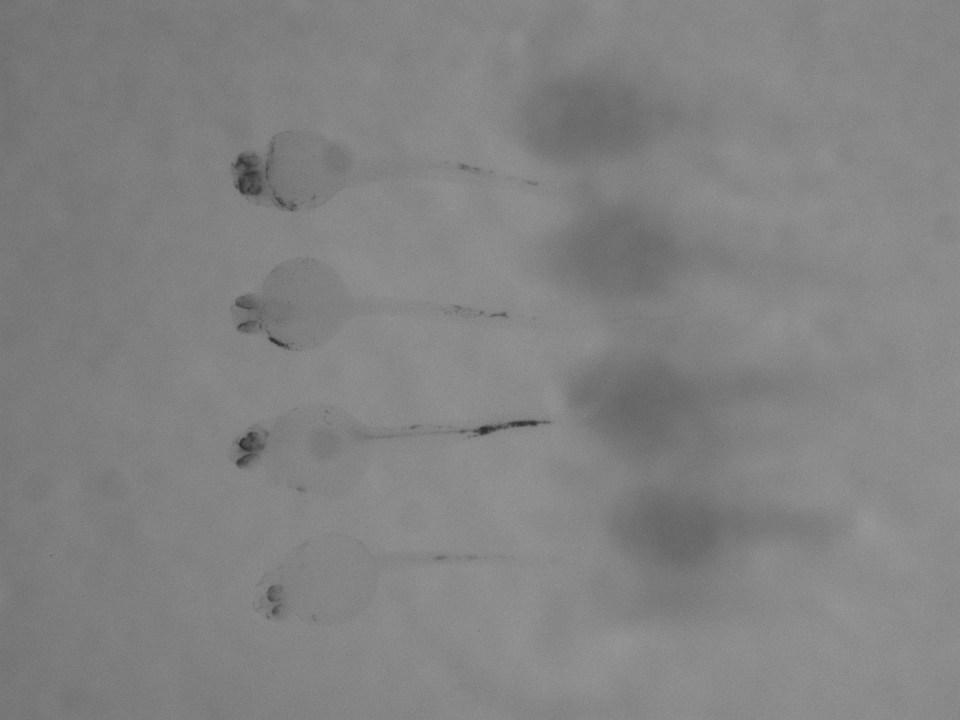

Supplement: Supplementary file 8 — Source data Fig. 4 [file 44321_2025_368_MOESM8_ESM.zip › FIGURE_4/4G/crRNA_rps19_ponatinib_1uM (5).tif]

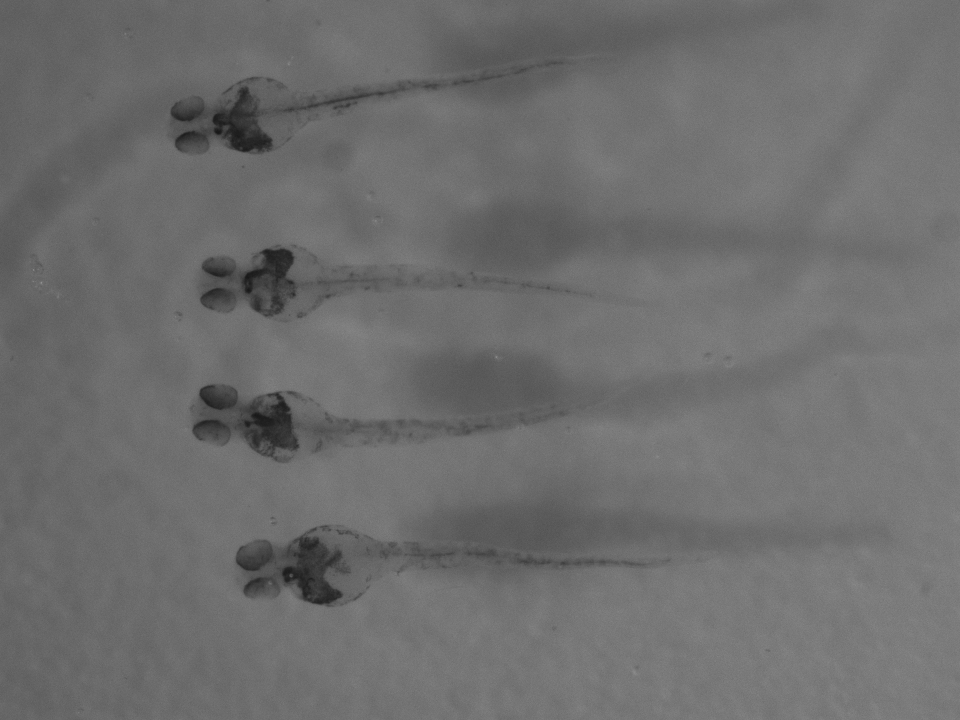

Supplement: Supplementary file 8 — Source data Fig. 4 [file 44321_2025_368_MOESM8_ESM.zip › FIGURE_4/4G/crRNA_std_DMSO (1).tif]

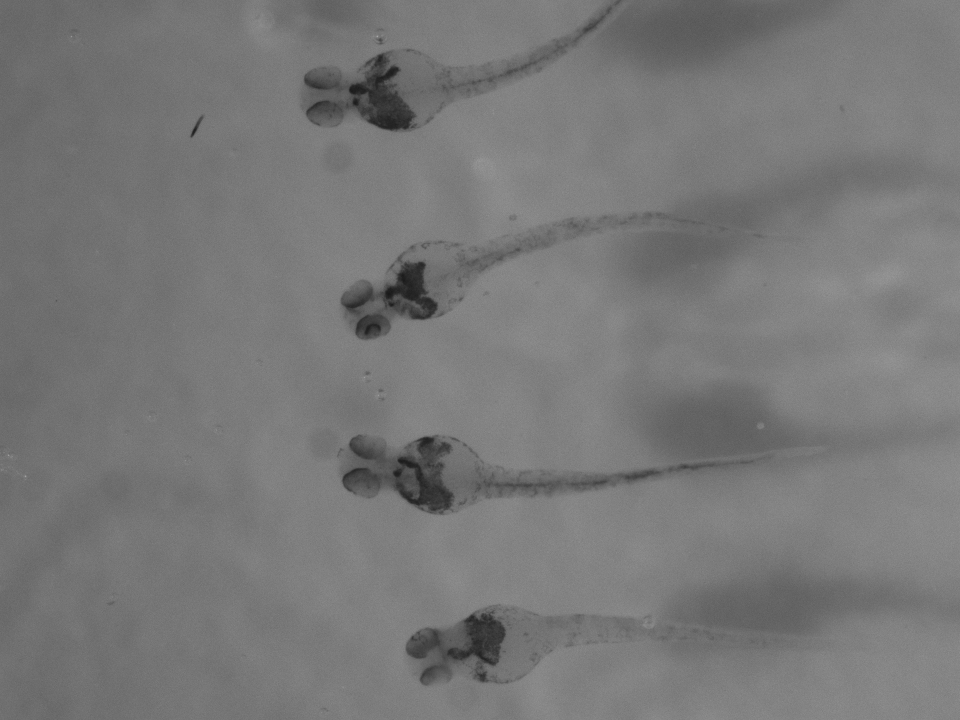

Supplement: Supplementary file 8 — Source data Fig. 4 [file 44321_2025_368_MOESM8_ESM.zip › FIGURE_4/4G/crRNA_std_DMSO (2).tif]

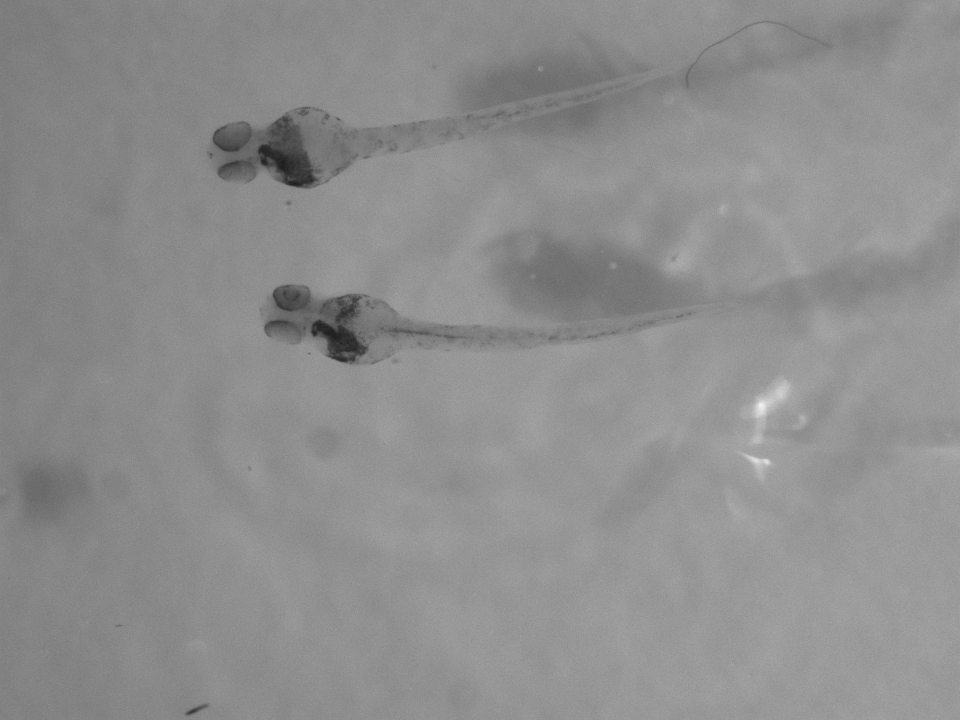

Supplement: Supplementary file 8 — Source data Fig. 4 [file 44321_2025_368_MOESM8_ESM.zip › FIGURE_4/4G/crRNA_std_DMSO (3).tif]

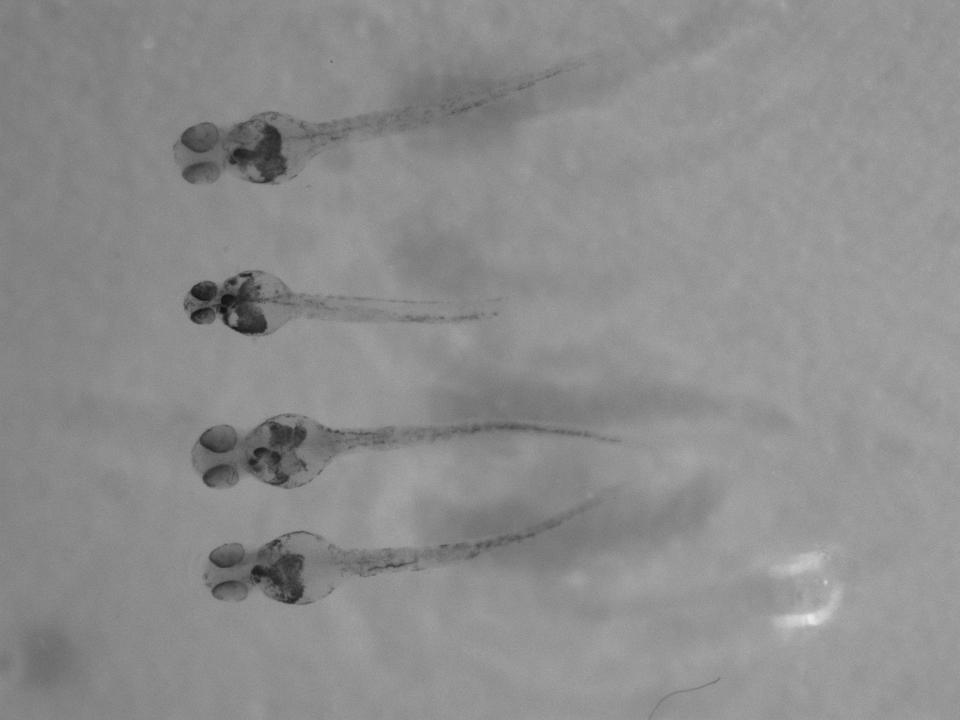

Supplement: Supplementary file 8 — Source data Fig. 4 [file 44321_2025_368_MOESM8_ESM.zip › FIGURE_4/4G/crRNA_std_DMSO (4).tif]

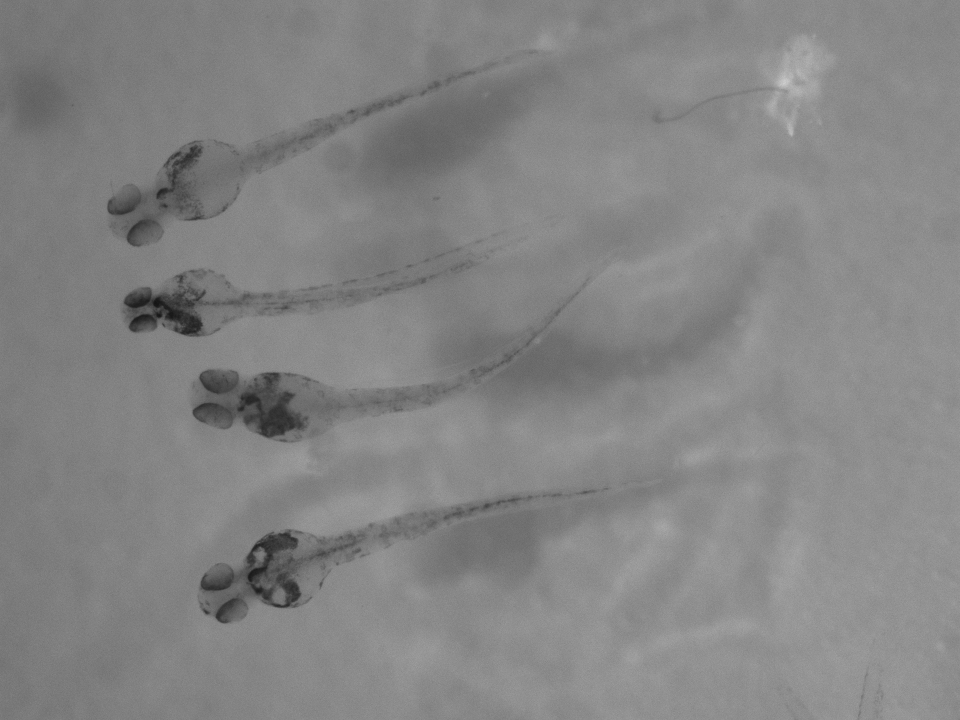

Supplement: Supplementary file 8 — Source data Fig. 4 [file 44321_2025_368_MOESM8_ESM.zip › FIGURE_4/4G/crRNA_std_DMSO (5).tif]

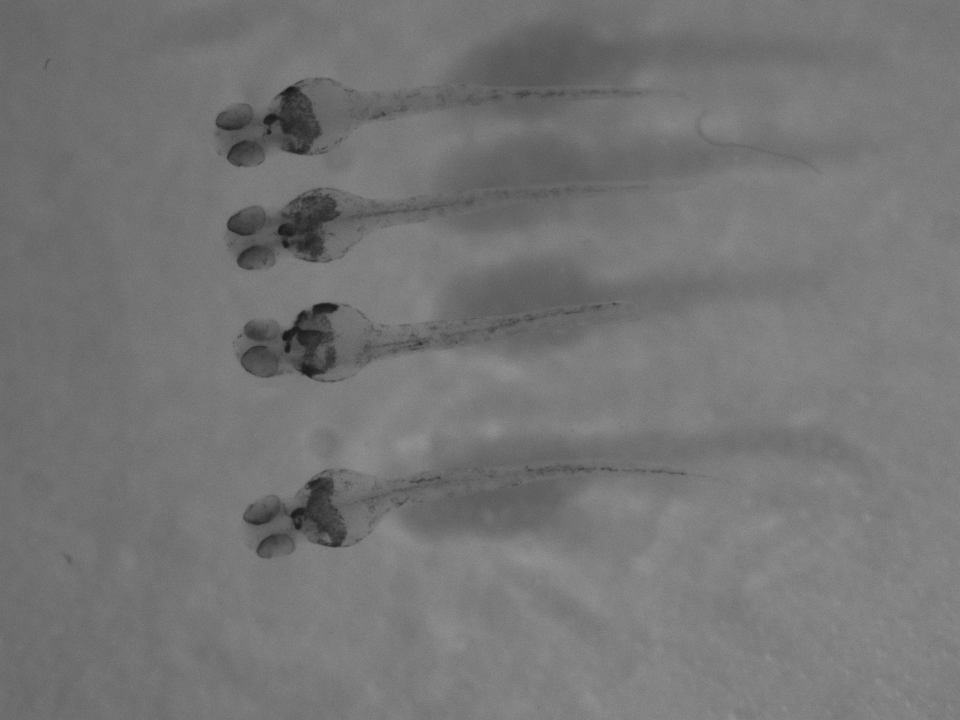

Supplement: Supplementary file 8 — Source data Fig. 4 [file 44321_2025_368_MOESM8_ESM.zip › FIGURE_4/4G/crRNA_std_DMSO (6).tif]

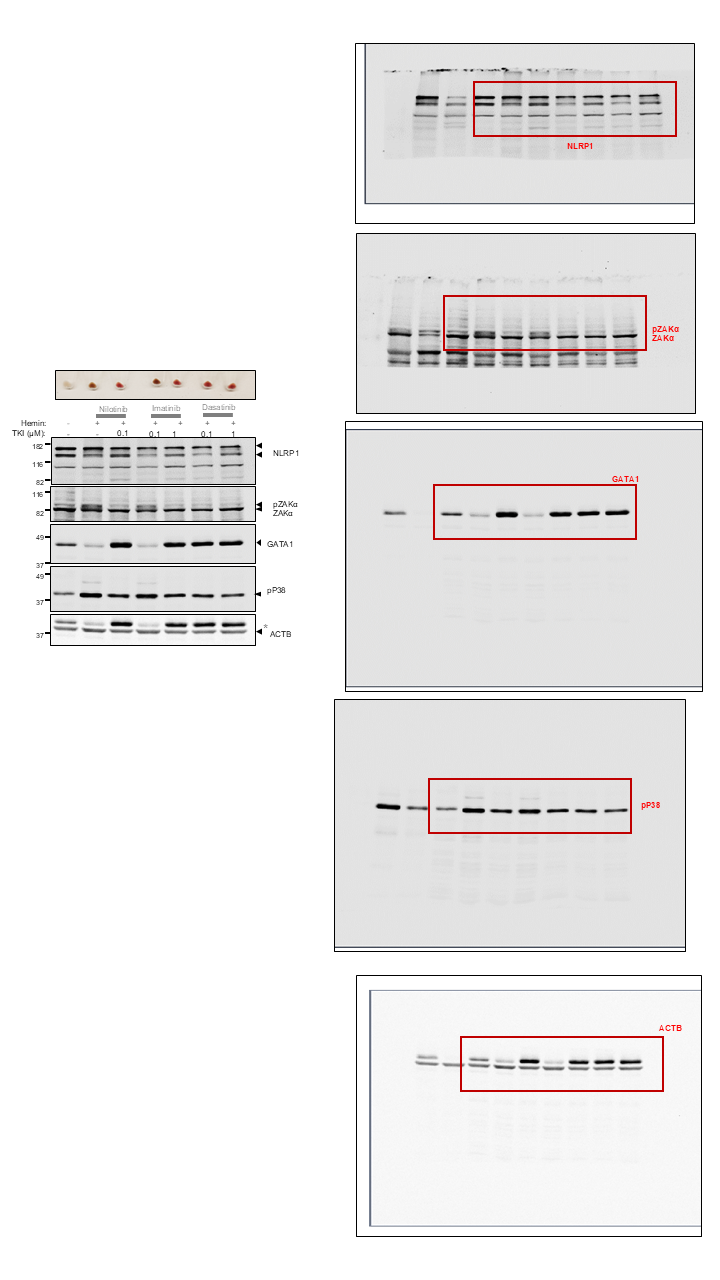

Supplement: Supplementary file 9 — Source data Fig. 5 [file 44321_2025_368_MOESM9_ESM.zip › FIGURE_5/5B.png]

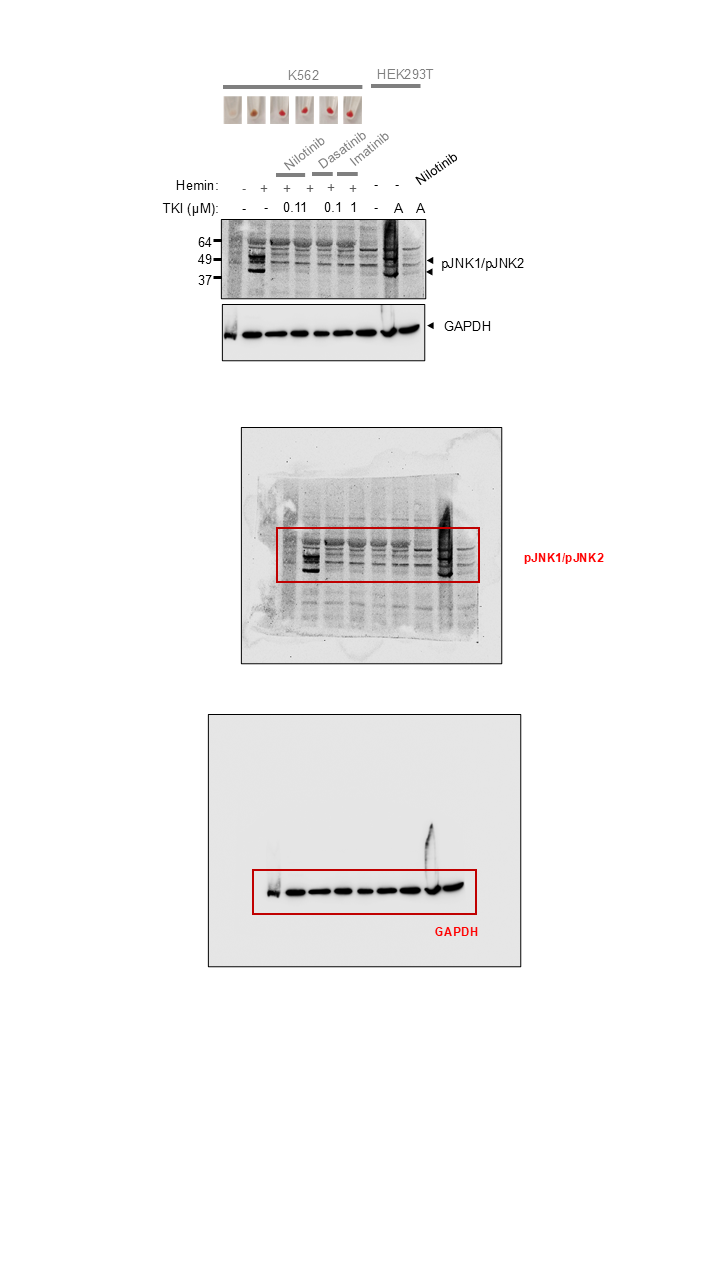

Supplement: Supplementary file 9 — Source data Fig. 5 [file 44321_2025_368_MOESM9_ESM.zip › FIGURE_5/5C.png]

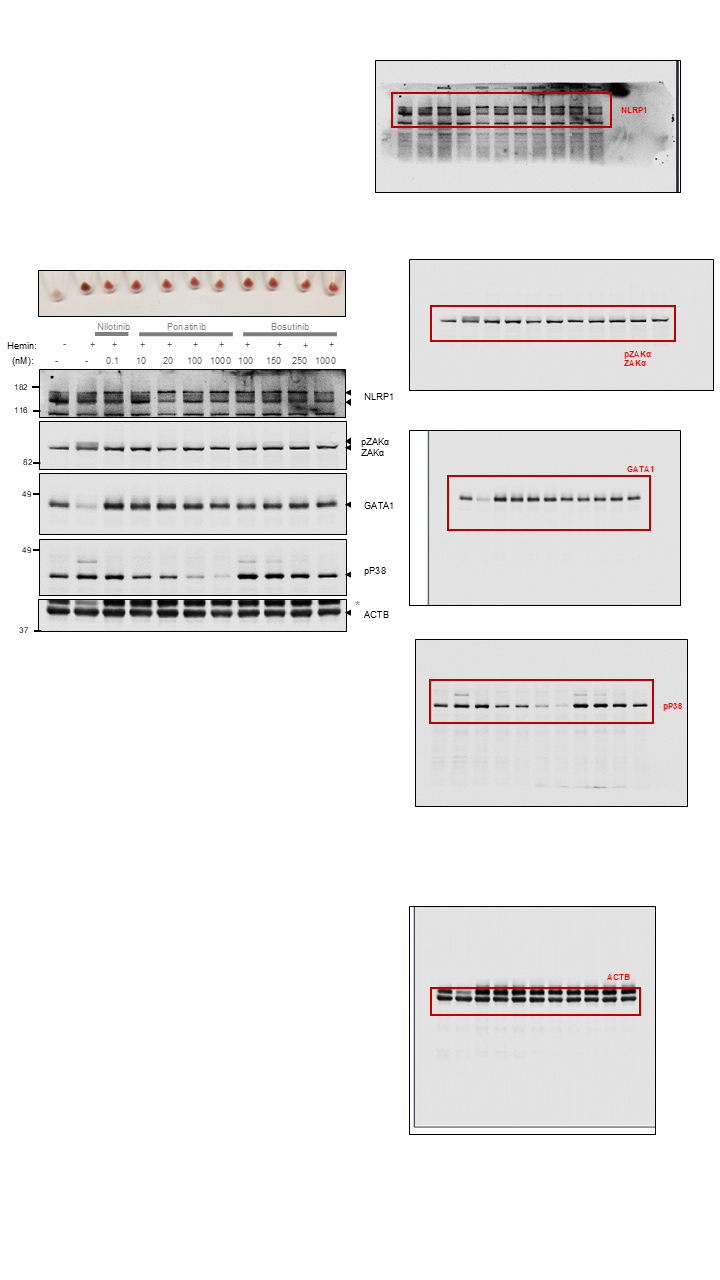

Supplement: Supplementary file 9 — Source data Fig. 5 [file 44321_2025_368_MOESM9_ESM.zip › FIGURE_5/5D.png]
