# Supplementary figures and images for: TKI-mediated inhibition of NLRP1 inflammasome restores erythropoiesis in DBA syndrome (part 3 of 4)
Source: EMBO Mol Med. 2026 Jan 9;18(2):702–24. doi: 10.1038/s44321-025-00368-3 (PMC12905221; doi:10.1038/s44321-025-00368-3)

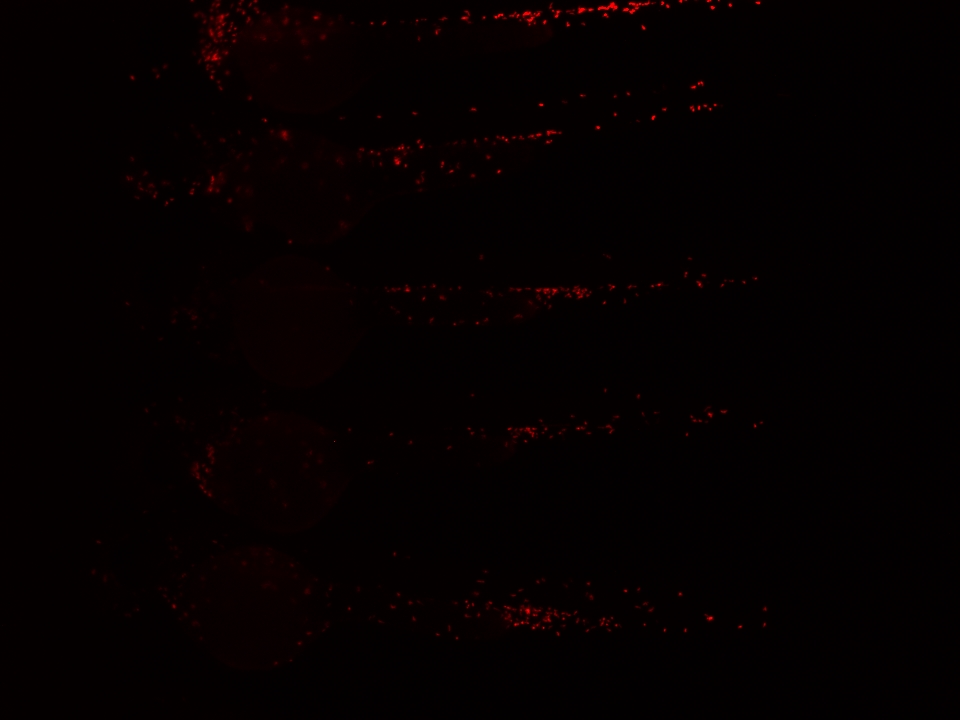

Supplement: Supplementary file 8 — Source data Fig. 4 [file 44321_2025_368_MOESM8_ESM.zip › FIGURE_4/4B/PONATINIB_1uM (11).jpg]

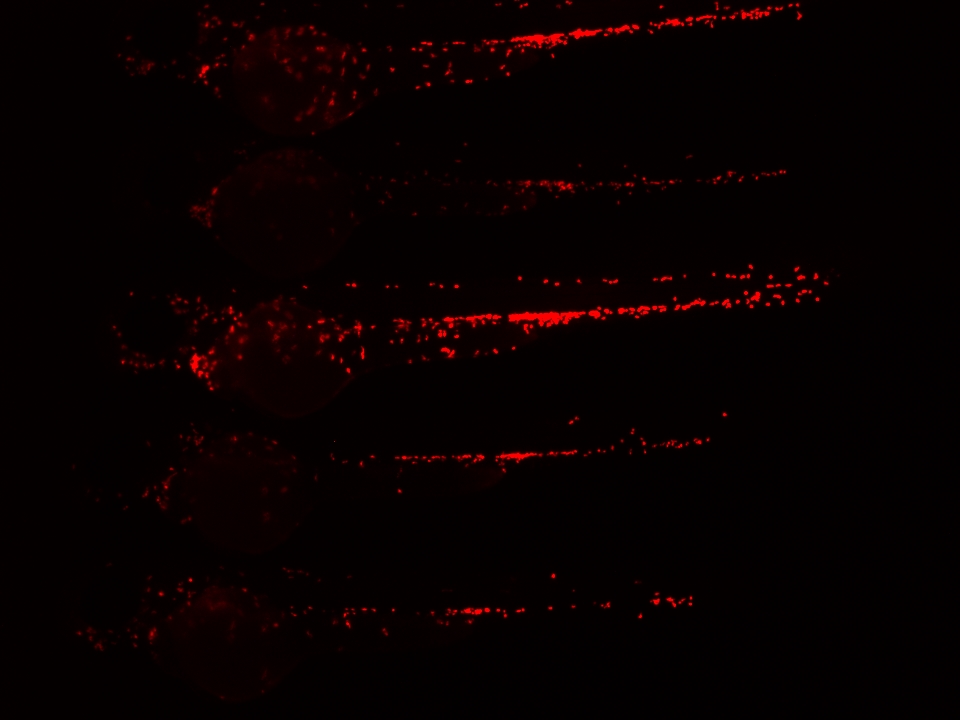

Supplement: Supplementary file 8 — Source data Fig. 4 [file 44321_2025_368_MOESM8_ESM.zip › FIGURE_4/4B/PONATINIB_1uM (2).jpg]

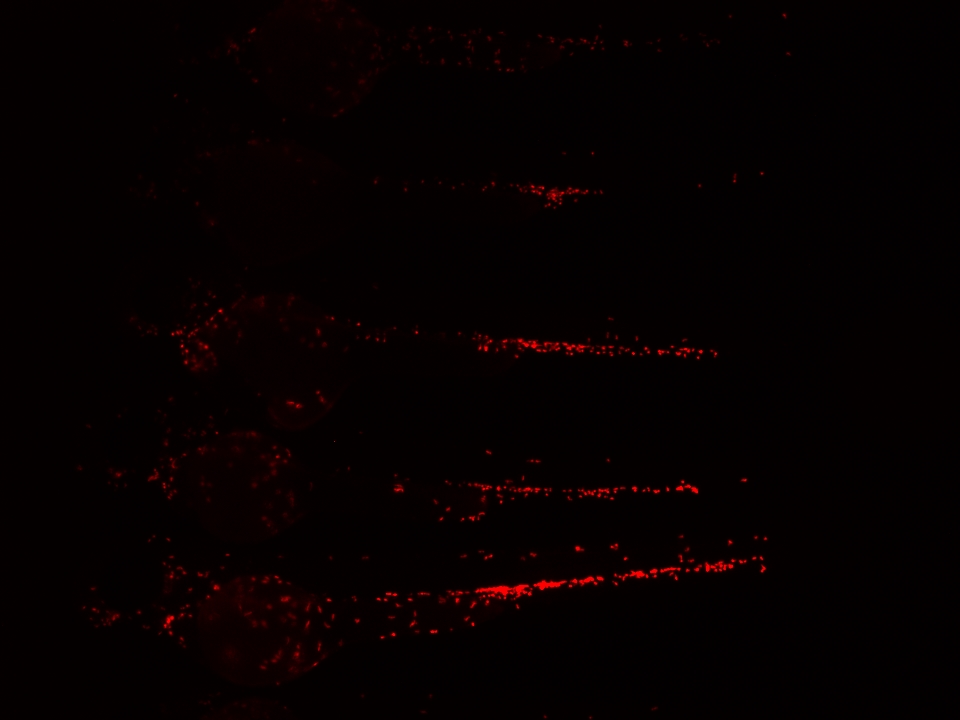

Supplement: Supplementary file 8 — Source data Fig. 4 [file 44321_2025_368_MOESM8_ESM.zip › FIGURE_4/4B/PONATINIB_1uM (3).jpg]

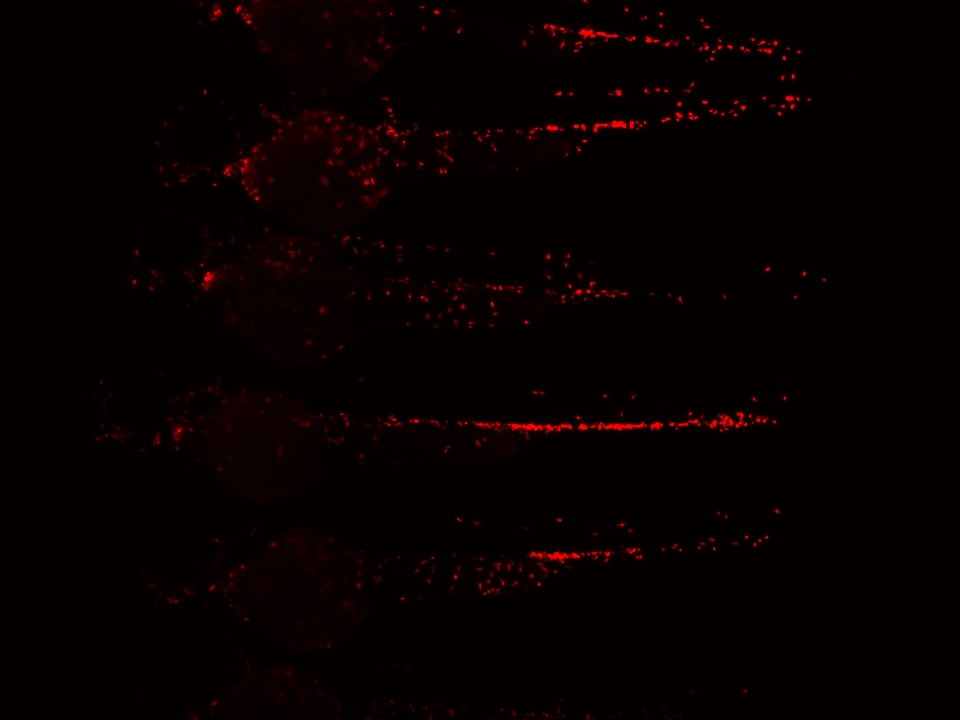

Supplement: Supplementary file 8 — Source data Fig. 4 [file 44321_2025_368_MOESM8_ESM.zip › FIGURE_4/4B/PONATINIB_1uM (4).jpg]

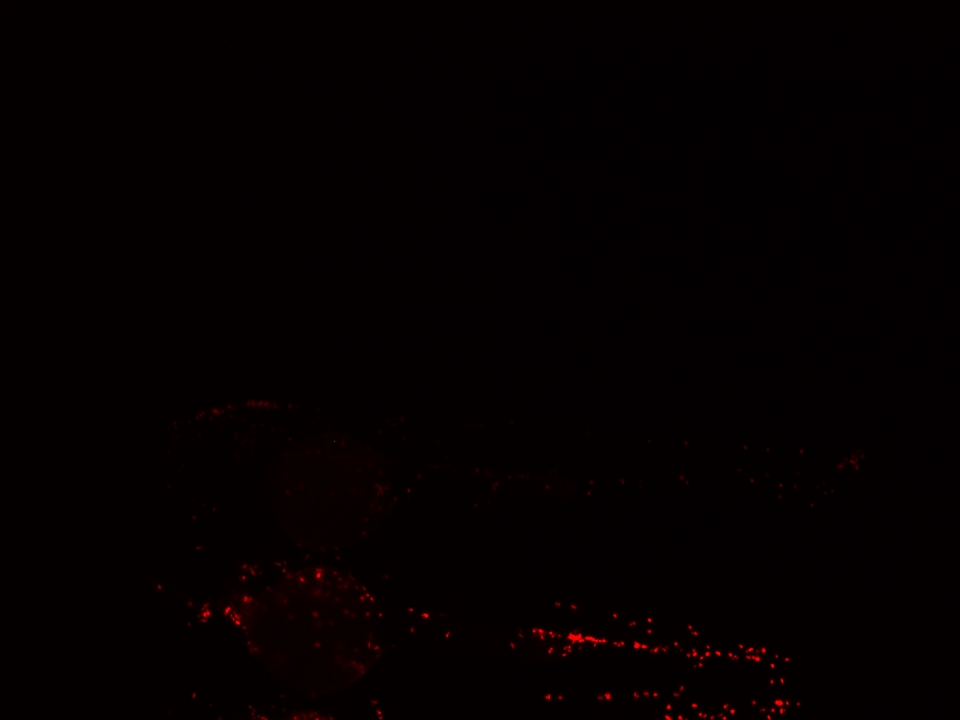

Supplement: Supplementary file 8 — Source data Fig. 4 [file 44321_2025_368_MOESM8_ESM.zip › FIGURE_4/4B/PONATINIB_1uM (5).jpg]

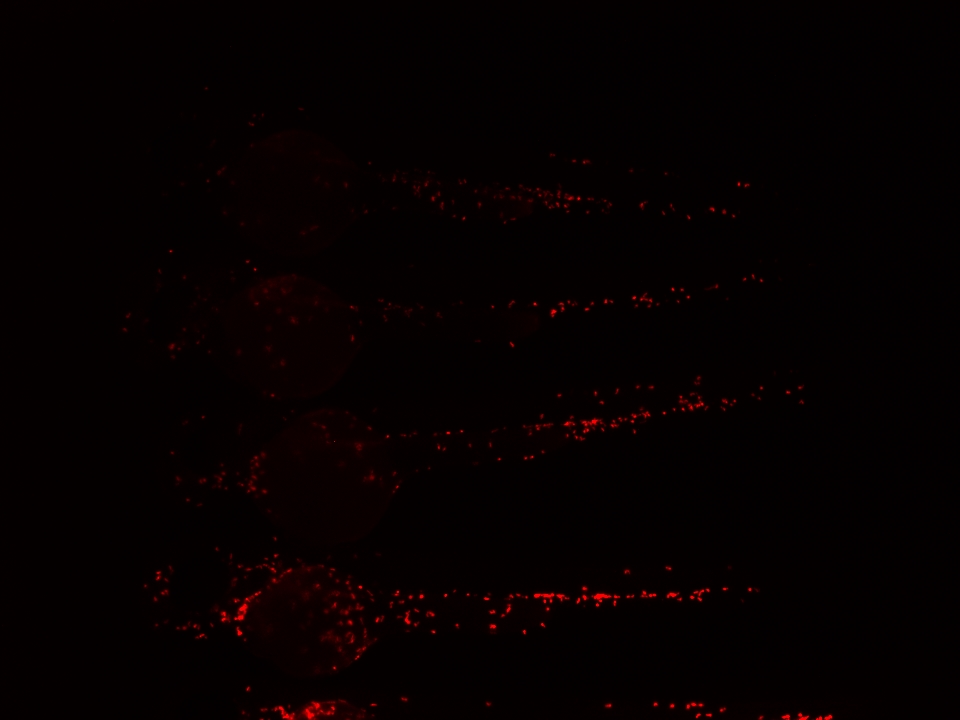

Supplement: Supplementary file 8 — Source data Fig. 4 [file 44321_2025_368_MOESM8_ESM.zip › FIGURE_4/4B/PONATINIB_1uM (6).jpg]

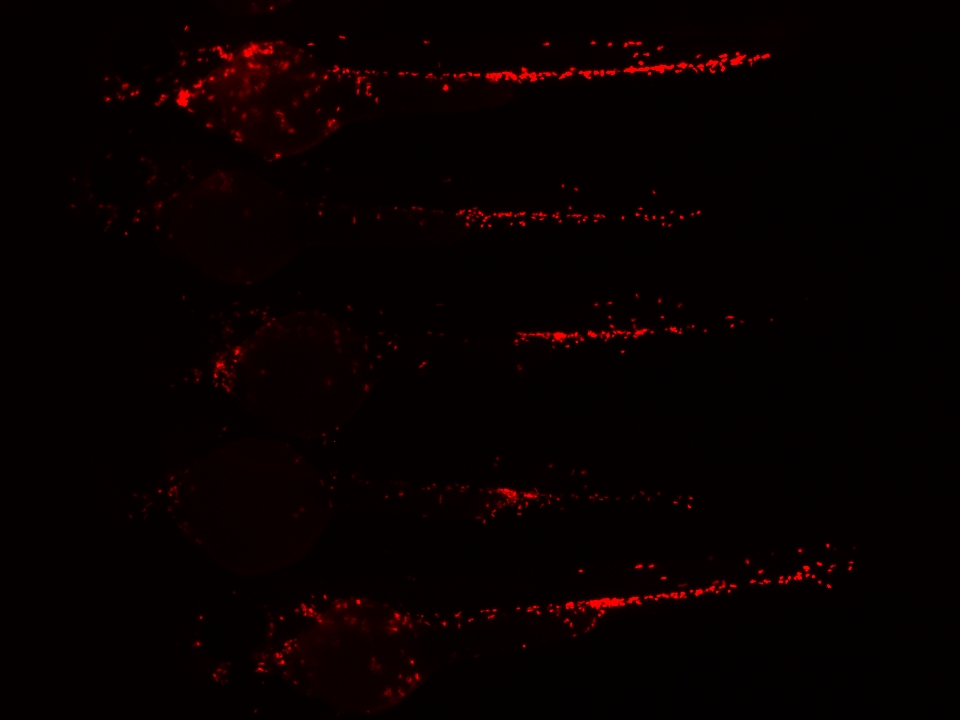

Supplement: Supplementary file 8 — Source data Fig. 4 [file 44321_2025_368_MOESM8_ESM.zip › FIGURE_4/4B/PONATINIB_1uM (7).jpg]

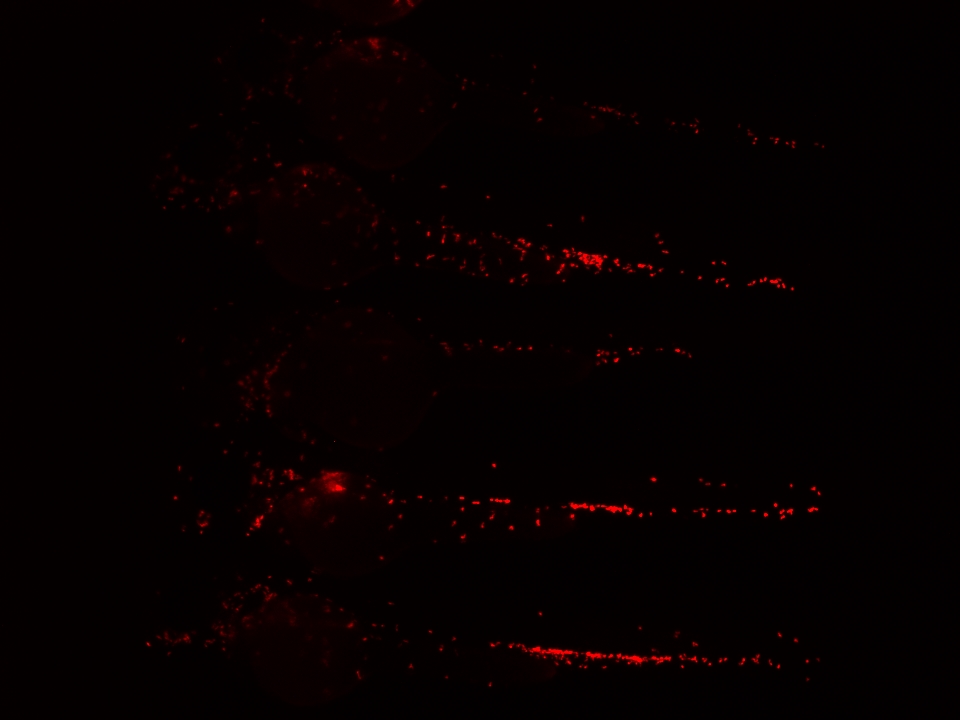

Supplement: Supplementary file 8 — Source data Fig. 4 [file 44321_2025_368_MOESM8_ESM.zip › FIGURE_4/4B/PONATINIB_1uM (8).jpg]

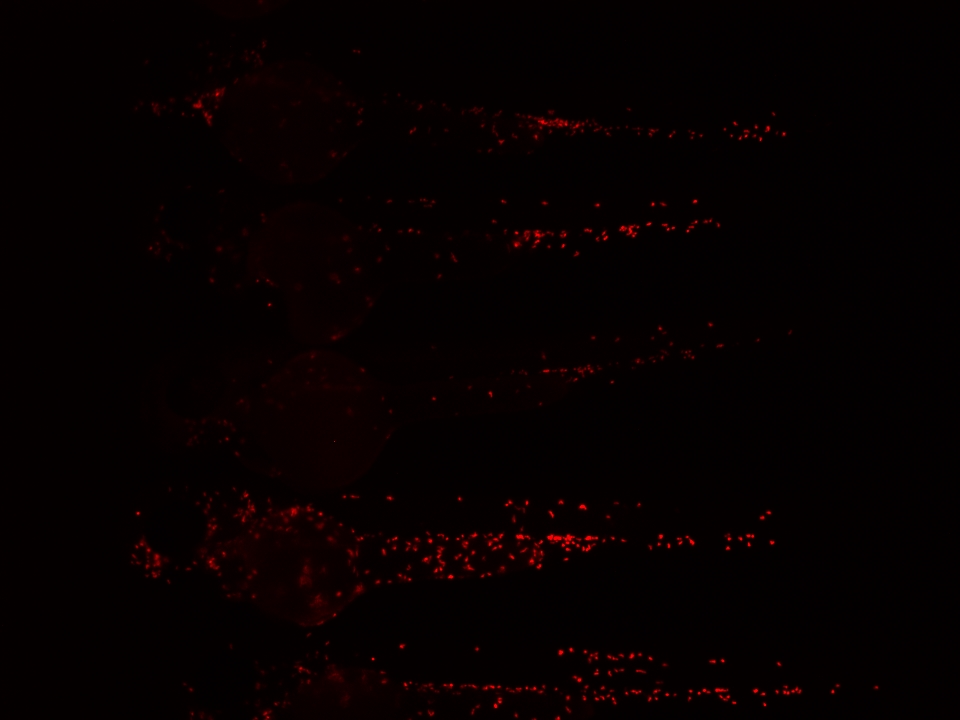

Supplement: Supplementary file 8 — Source data Fig. 4 [file 44321_2025_368_MOESM8_ESM.zip › FIGURE_4/4B/PONATINIB_1uM (9).jpg]

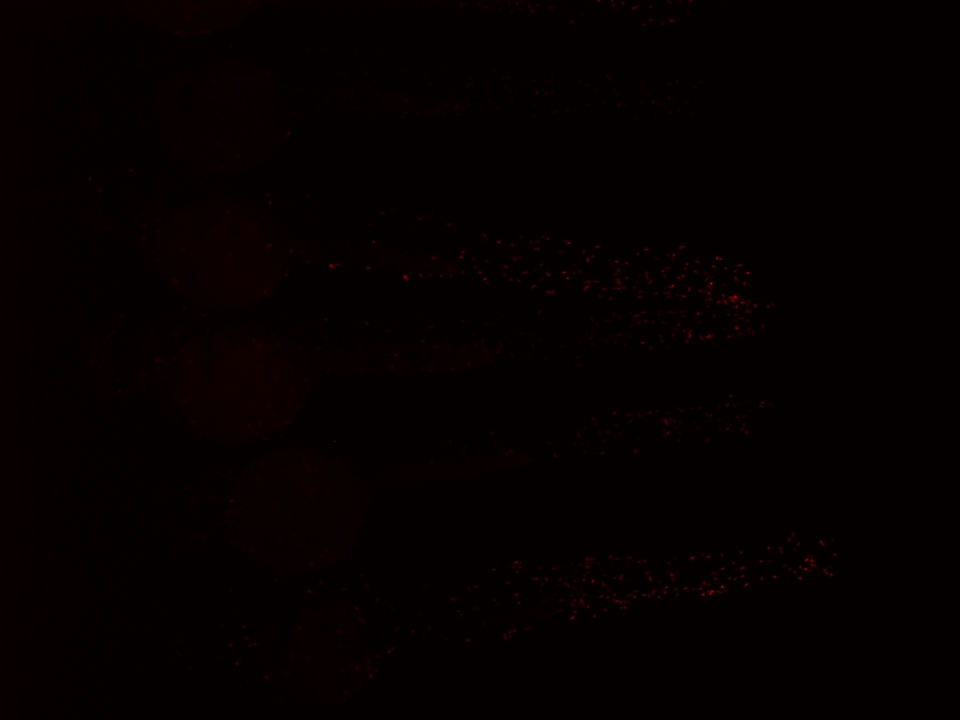

Supplement: Supplementary file 8 — Source data Fig. 4 [file 44321_2025_368_MOESM8_ESM.zip › FIGURE_4/4D/BOSUTINIB_01uM (1).jpg]

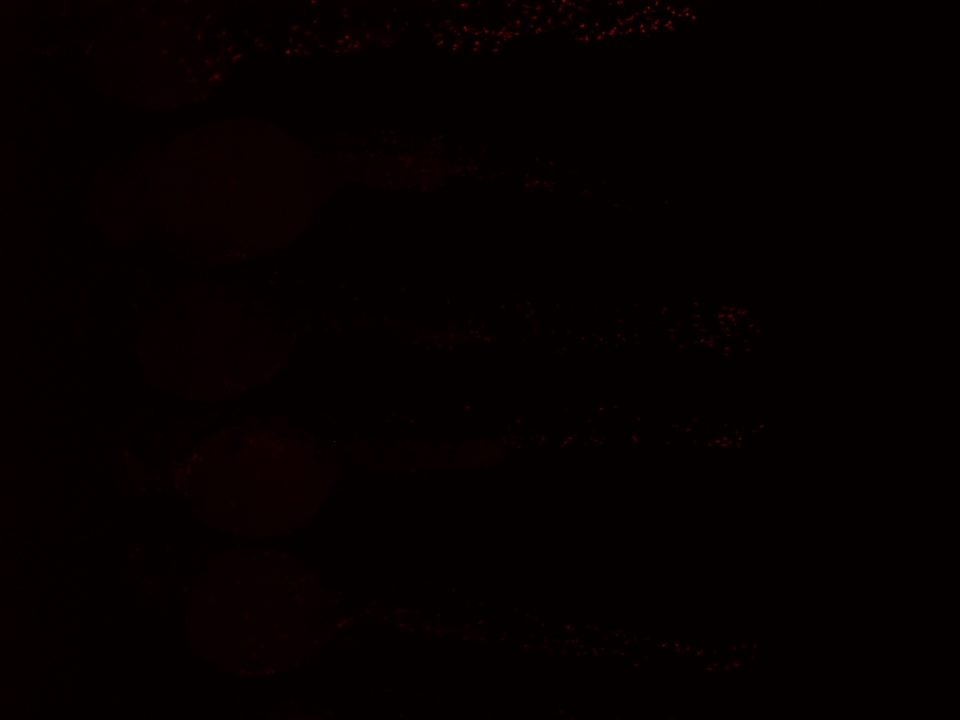

Supplement: Supplementary file 8 — Source data Fig. 4 [file 44321_2025_368_MOESM8_ESM.zip › FIGURE_4/4D/BOSUTINIB_01uM (2).jpg]

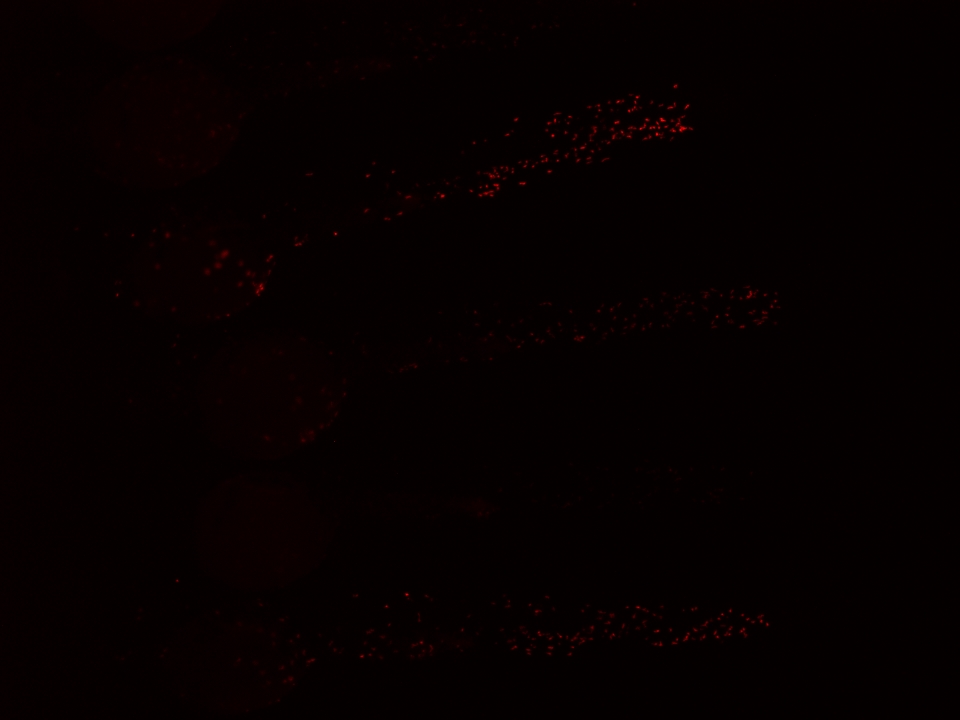

Supplement: Supplementary file 8 — Source data Fig. 4 [file 44321_2025_368_MOESM8_ESM.zip › FIGURE_4/4D/BOSUTINIB_01uM (3).jpg]

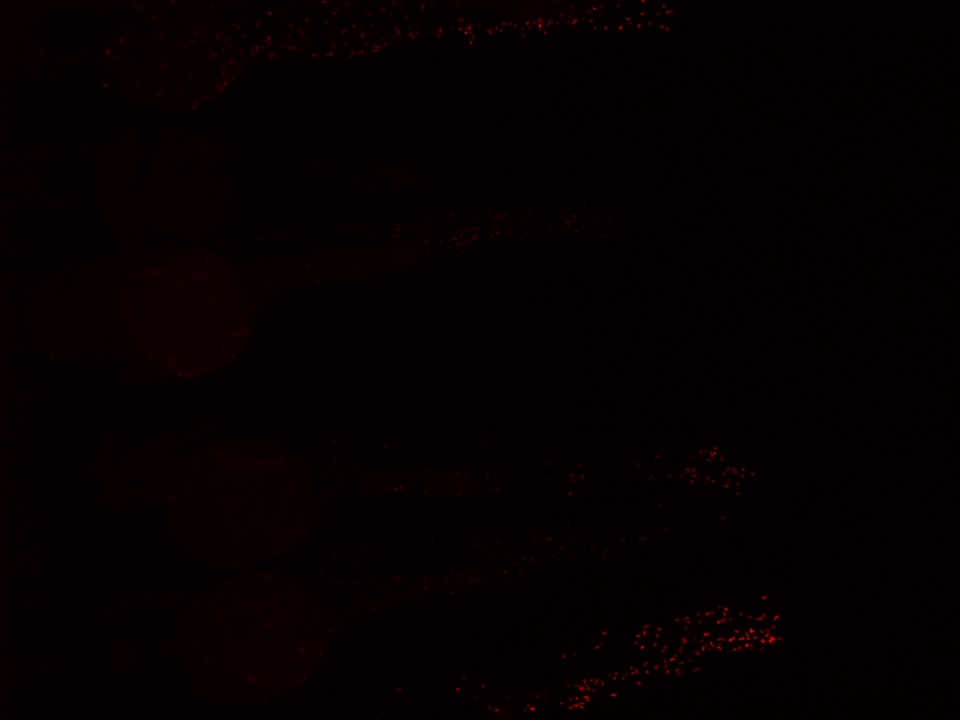

Supplement: Supplementary file 8 — Source data Fig. 4 [file 44321_2025_368_MOESM8_ESM.zip › FIGURE_4/4D/BOSUTINIB_01uM (4).jpg]

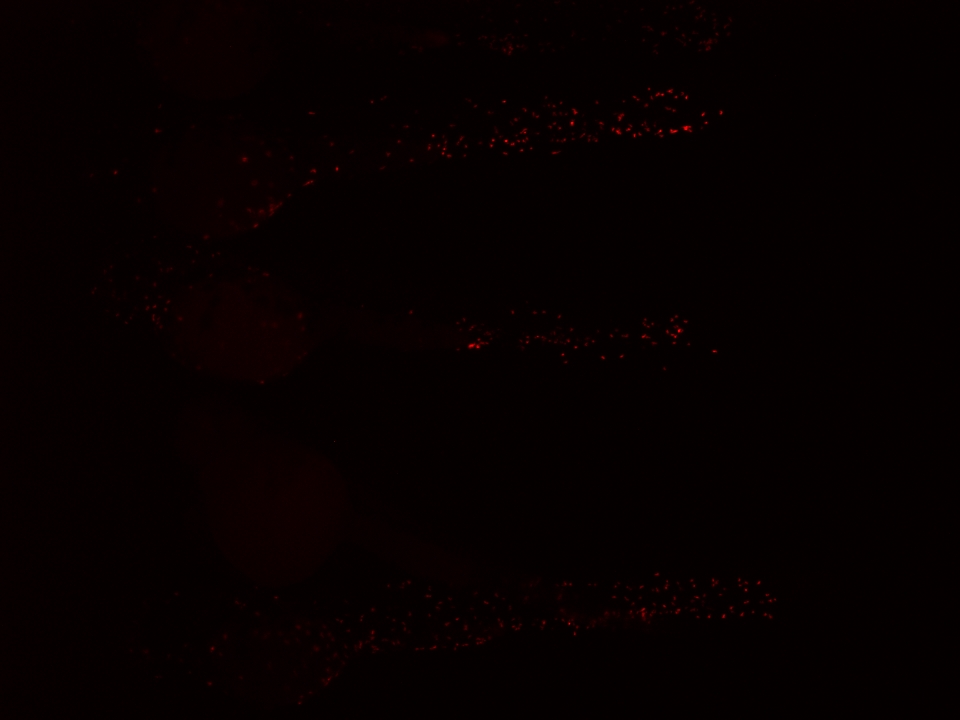

Supplement: Supplementary file 8 — Source data Fig. 4 [file 44321_2025_368_MOESM8_ESM.zip › FIGURE_4/4D/BOSUTINIB_01uM (5).jpg]

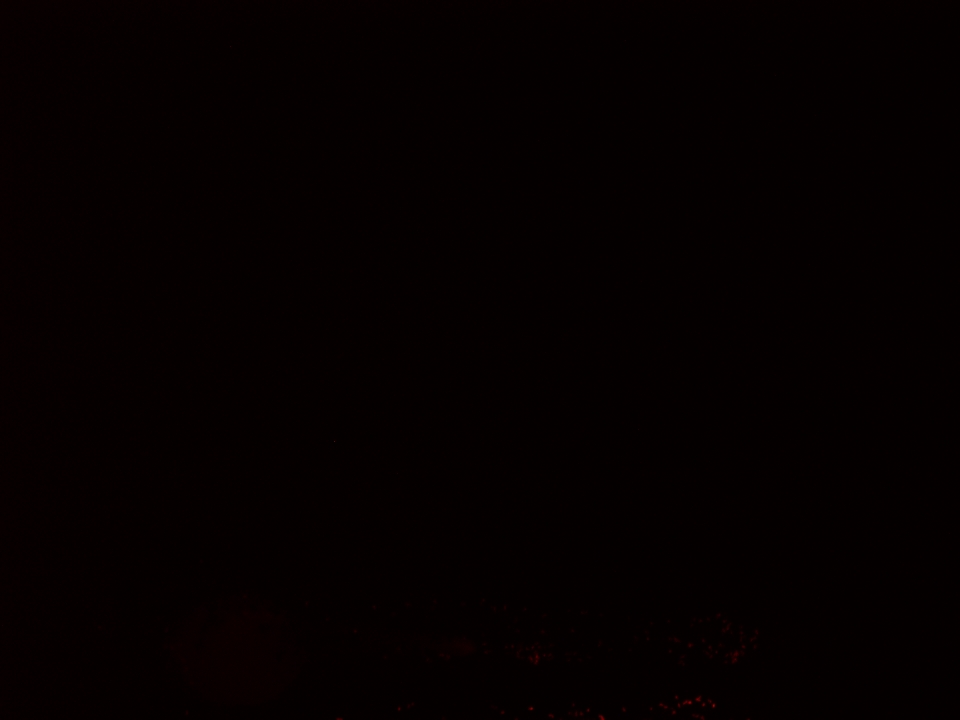

Supplement: Supplementary file 8 — Source data Fig. 4 [file 44321_2025_368_MOESM8_ESM.zip › FIGURE_4/4D/BOSUTINIB_01uM (6).jpg]

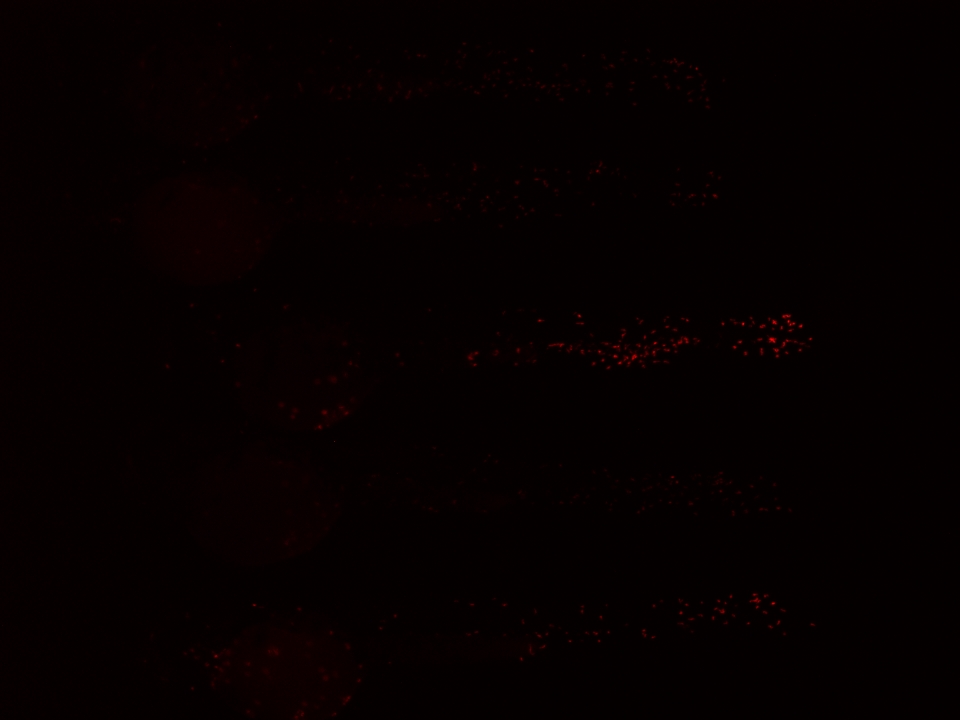

Supplement: Supplementary file 8 — Source data Fig. 4 [file 44321_2025_368_MOESM8_ESM.zip › FIGURE_4/4D/BOSUTINIB_01uM (7).jpg]

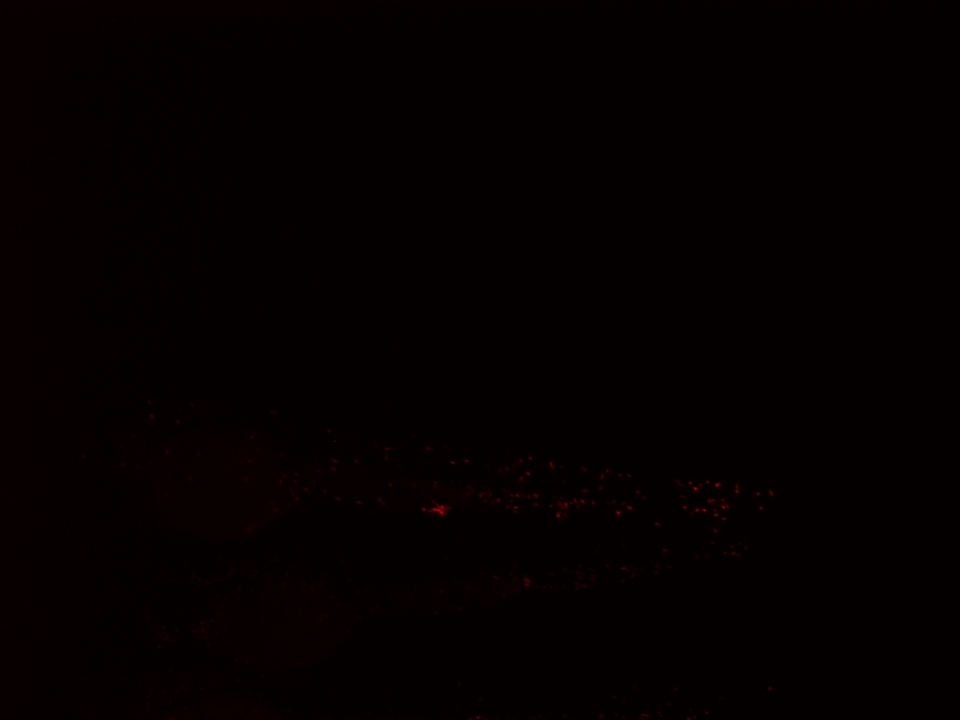

Supplement: Supplementary file 8 — Source data Fig. 4 [file 44321_2025_368_MOESM8_ESM.zip › FIGURE_4/4D/BOSUTINIB_1uM (1).jpg]

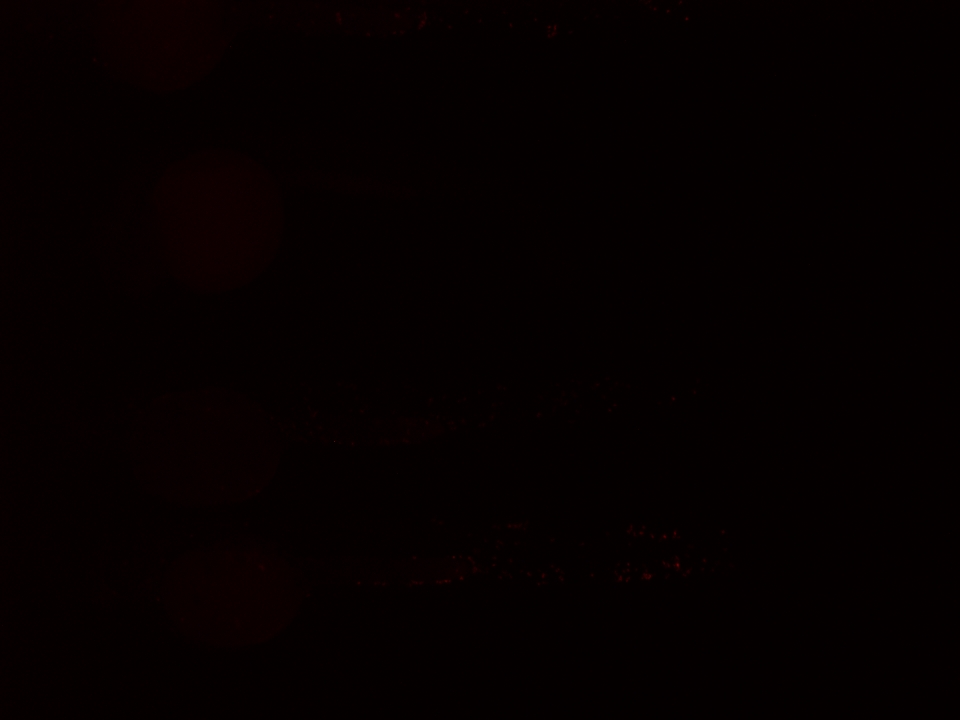

Supplement: Supplementary file 8 — Source data Fig. 4 [file 44321_2025_368_MOESM8_ESM.zip › FIGURE_4/4D/BOSUTINIB_1uM (2).jpg]

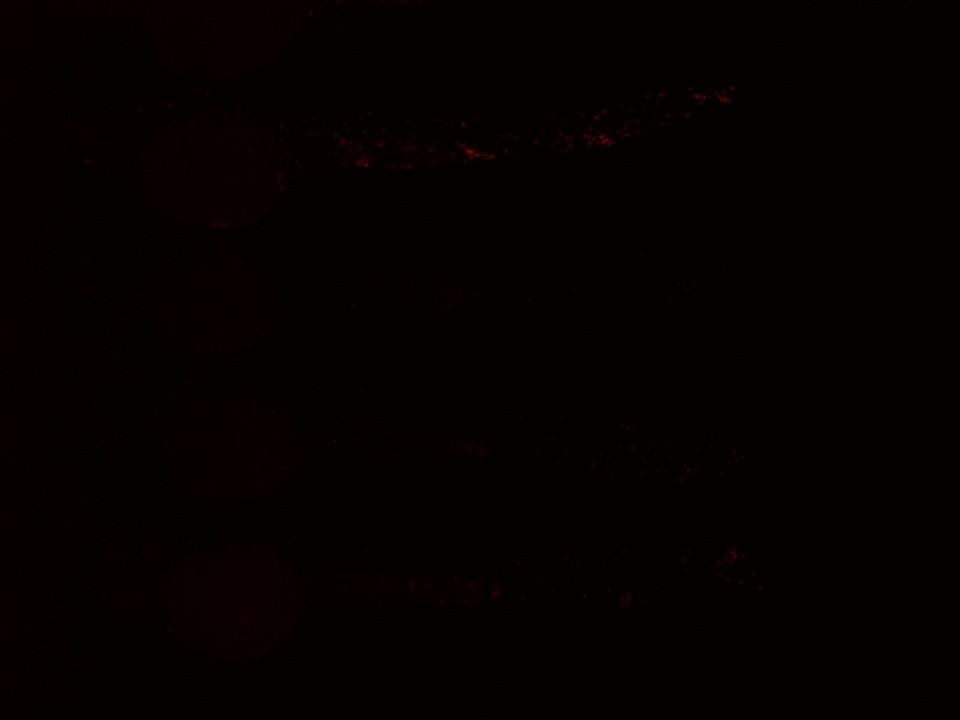

Supplement: Supplementary file 8 — Source data Fig. 4 [file 44321_2025_368_MOESM8_ESM.zip › FIGURE_4/4D/BOSUTINIB_1uM (3).jpg]

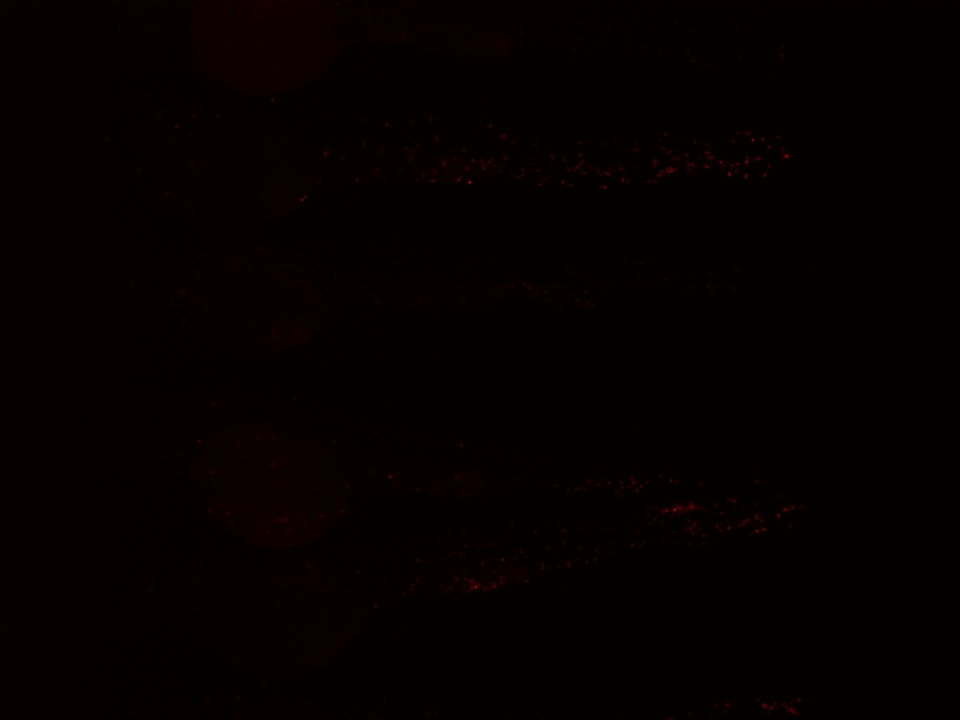

Supplement: Supplementary file 8 — Source data Fig. 4 [file 44321_2025_368_MOESM8_ESM.zip › FIGURE_4/4D/BOSUTINIB_1uM (4).jpg]

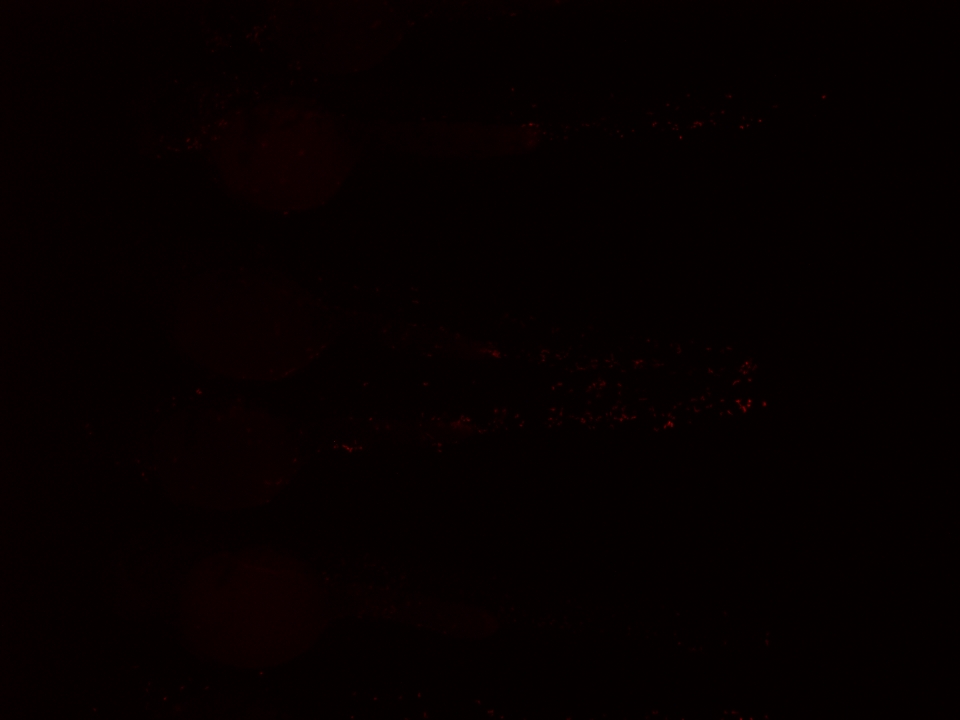

Supplement: Supplementary file 8 — Source data Fig. 4 [file 44321_2025_368_MOESM8_ESM.zip › FIGURE_4/4D/BOSUTINIB_1uM (5).jpg]

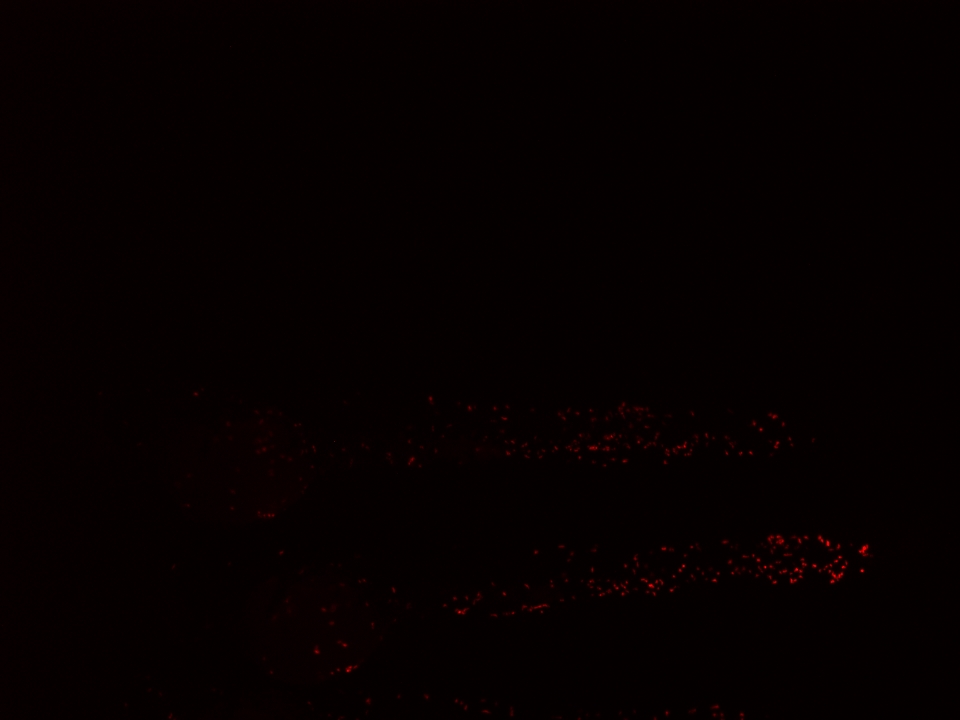

Supplement: Supplementary file 8 — Source data Fig. 4 [file 44321_2025_368_MOESM8_ESM.zip › FIGURE_4/4D/DASATINIB_01uM (1).jpg]

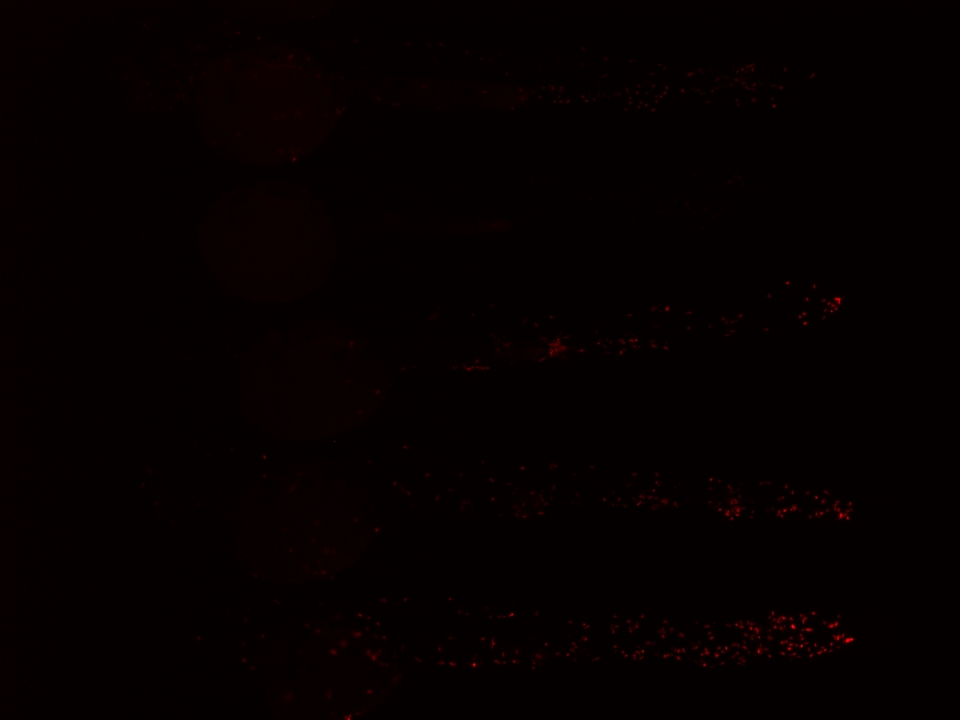

Supplement: Supplementary file 8 — Source data Fig. 4 [file 44321_2025_368_MOESM8_ESM.zip › FIGURE_4/4D/DASATINIB_01uM (2).jpg]

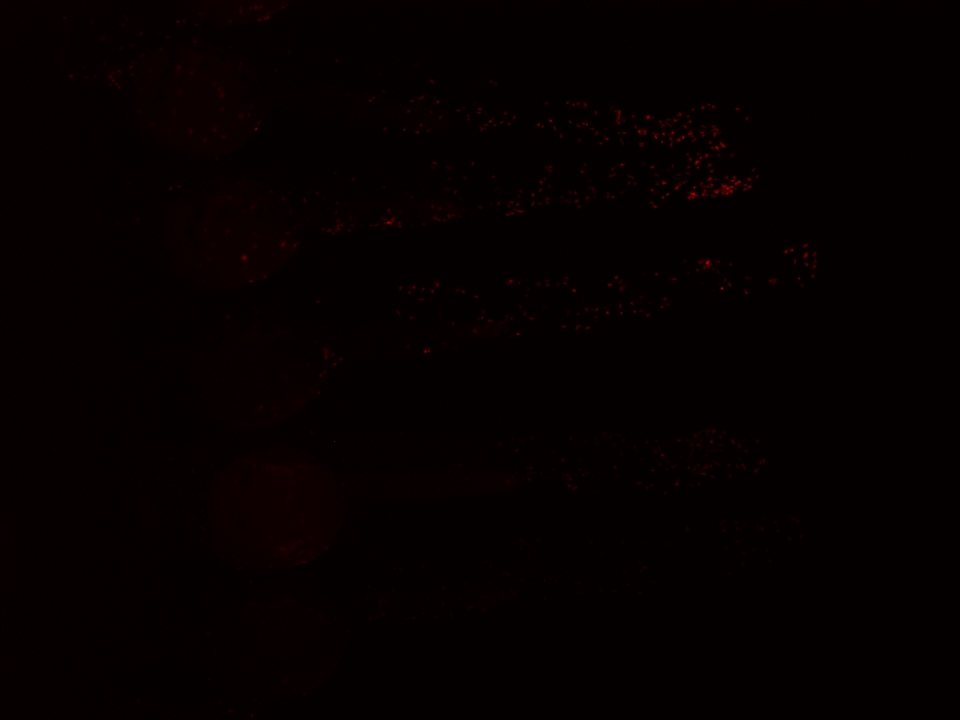

Supplement: Supplementary file 8 — Source data Fig. 4 [file 44321_2025_368_MOESM8_ESM.zip › FIGURE_4/4D/DASATINIB_01uM (3).jpg]

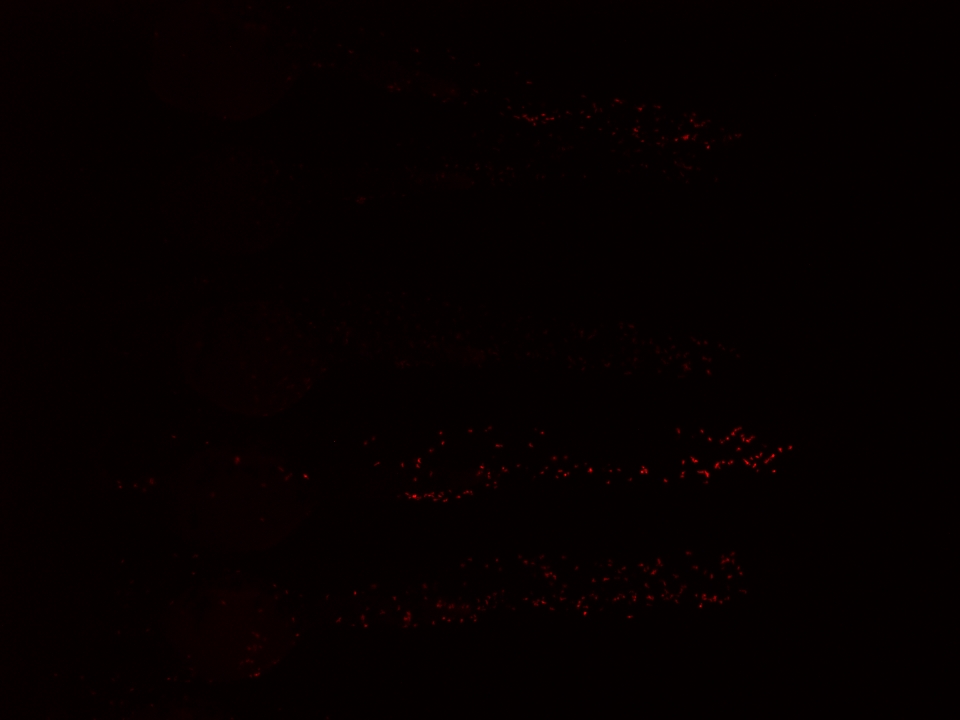

Supplement: Supplementary file 8 — Source data Fig. 4 [file 44321_2025_368_MOESM8_ESM.zip › FIGURE_4/4D/DASATINIB_01uM (4).jpg]

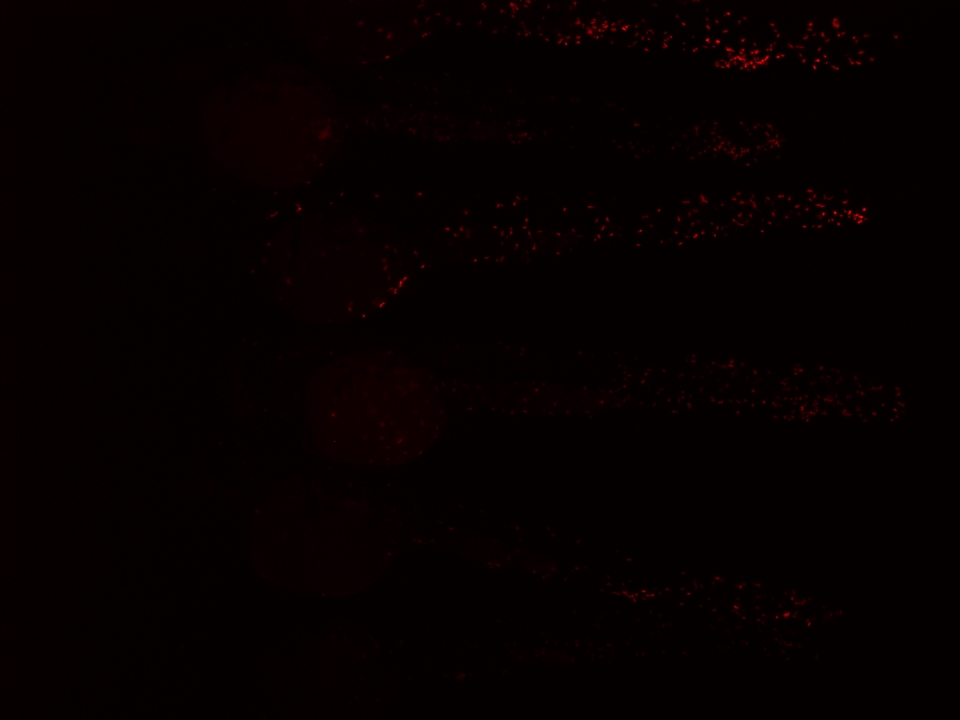

Supplement: Supplementary file 8 — Source data Fig. 4 [file 44321_2025_368_MOESM8_ESM.zip › FIGURE_4/4D/DASATINIB_01uM (5).jpg]

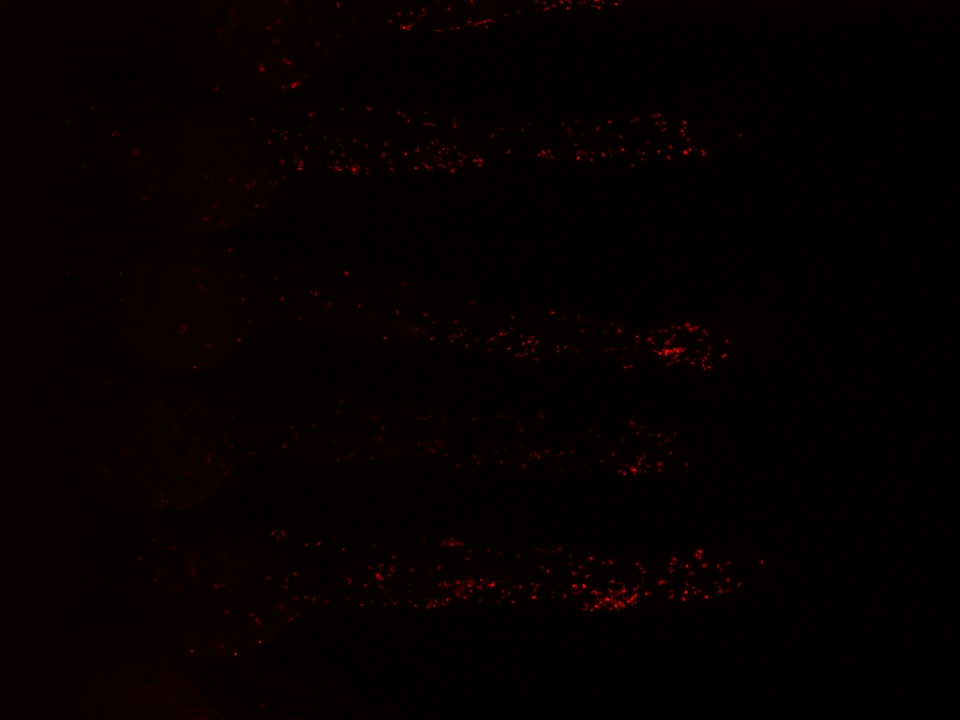

Supplement: Supplementary file 8 — Source data Fig. 4 [file 44321_2025_368_MOESM8_ESM.zip › FIGURE_4/4D/DASATINIB_01uM (6).jpg]

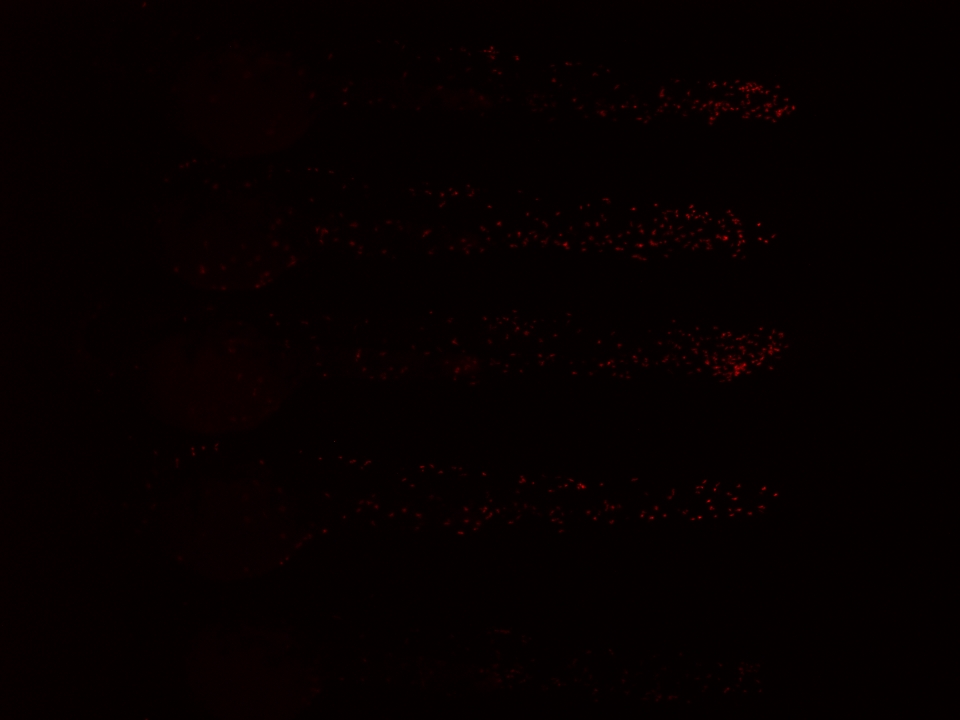

Supplement: Supplementary file 8 — Source data Fig. 4 [file 44321_2025_368_MOESM8_ESM.zip › FIGURE_4/4D/DASATINIB_1uM (1).jpg]

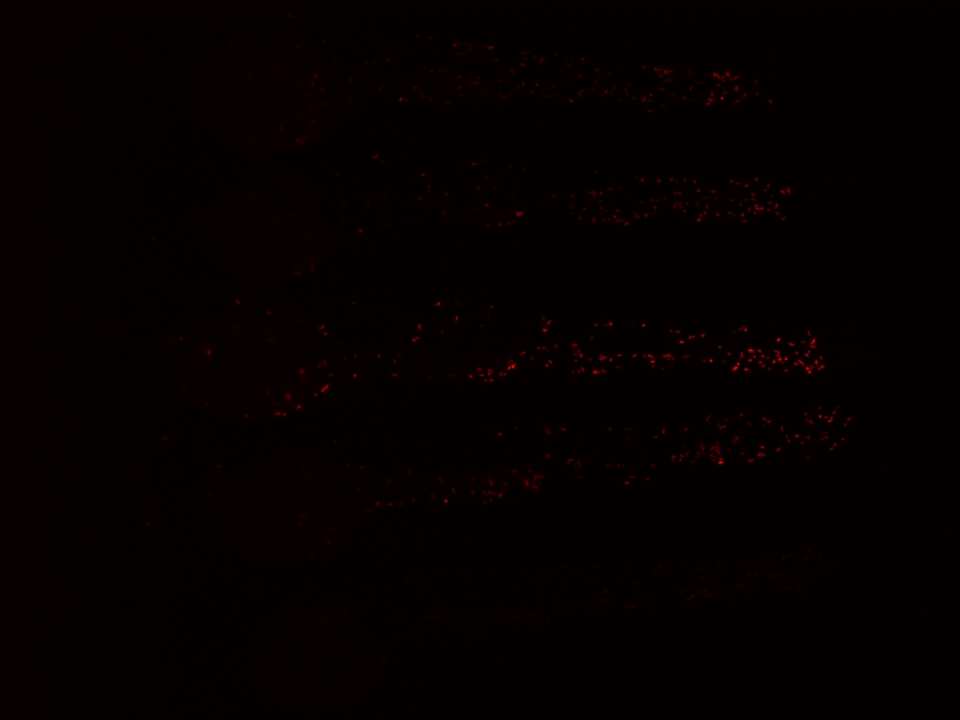

Supplement: Supplementary file 8 — Source data Fig. 4 [file 44321_2025_368_MOESM8_ESM.zip › FIGURE_4/4D/DASATINIB_1uM (2).jpg]

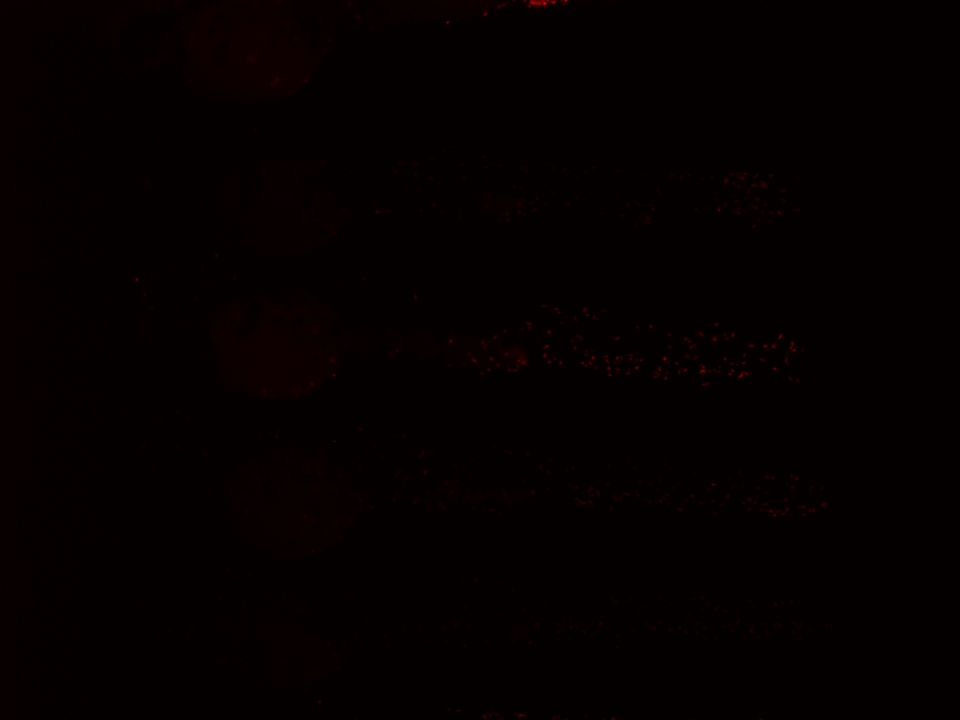

Supplement: Supplementary file 8 — Source data Fig. 4 [file 44321_2025_368_MOESM8_ESM.zip › FIGURE_4/4D/DASATINIB_1uM (3).jpg]

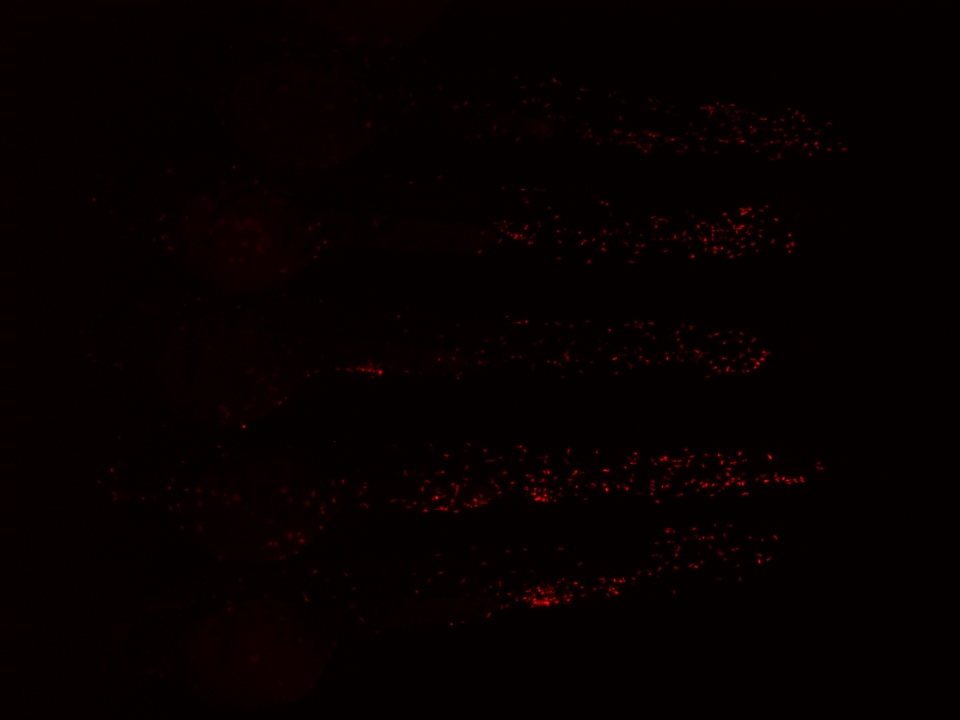

Supplement: Supplementary file 8 — Source data Fig. 4 [file 44321_2025_368_MOESM8_ESM.zip › FIGURE_4/4D/DASATINIB_1uM (4).jpg]

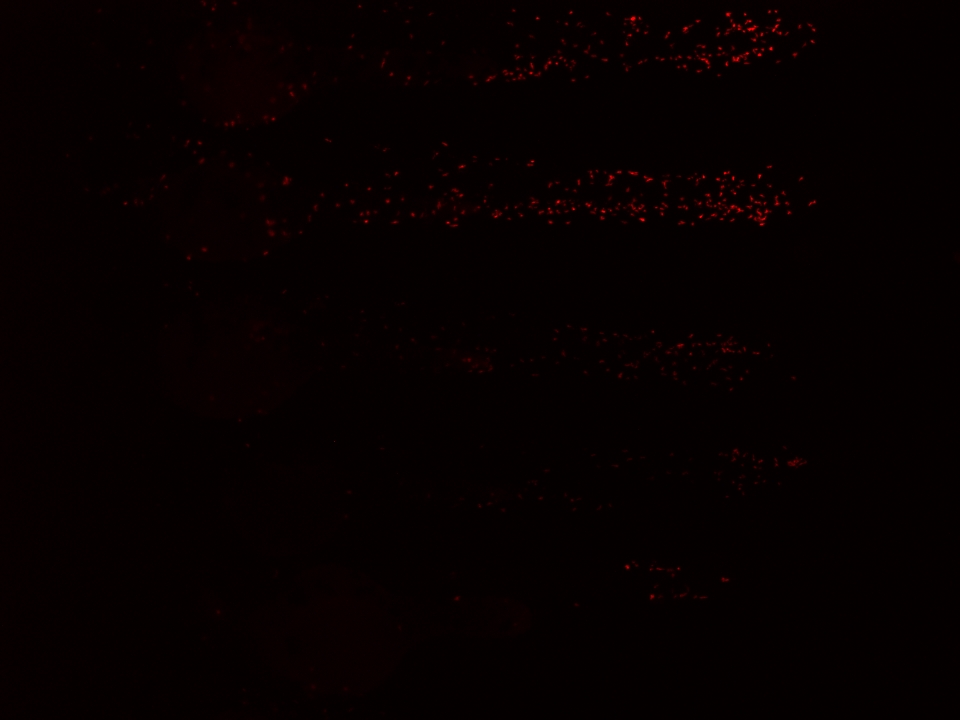

Supplement: Supplementary file 8 — Source data Fig. 4 [file 44321_2025_368_MOESM8_ESM.zip › FIGURE_4/4D/DASATINIB_1uM (5).jpg]

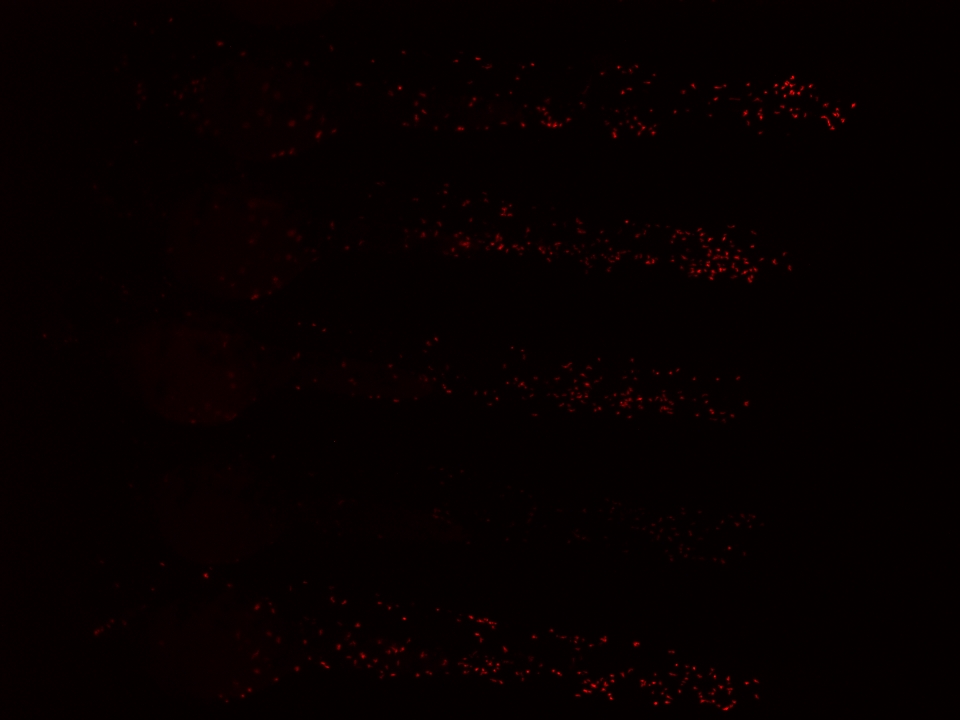

Supplement: Supplementary file 8 — Source data Fig. 4 [file 44321_2025_368_MOESM8_ESM.zip › FIGURE_4/4D/DASATINIB_1uM (6).jpg]

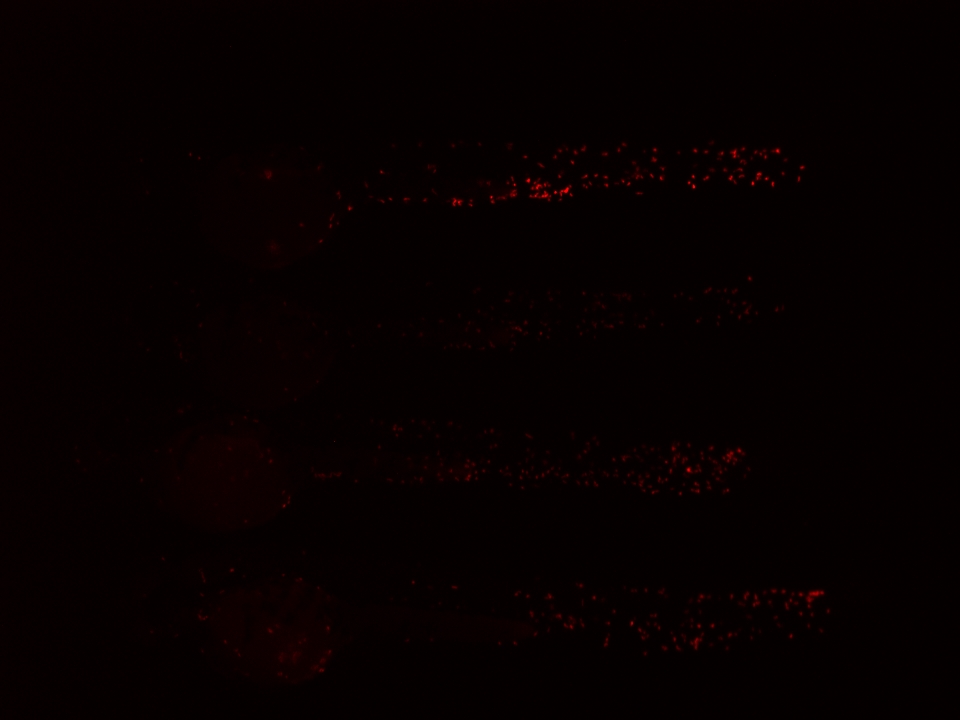

Supplement: Supplementary file 8 — Source data Fig. 4 [file 44321_2025_368_MOESM8_ESM.zip › FIGURE_4/4D/DMSO (1).jpg]

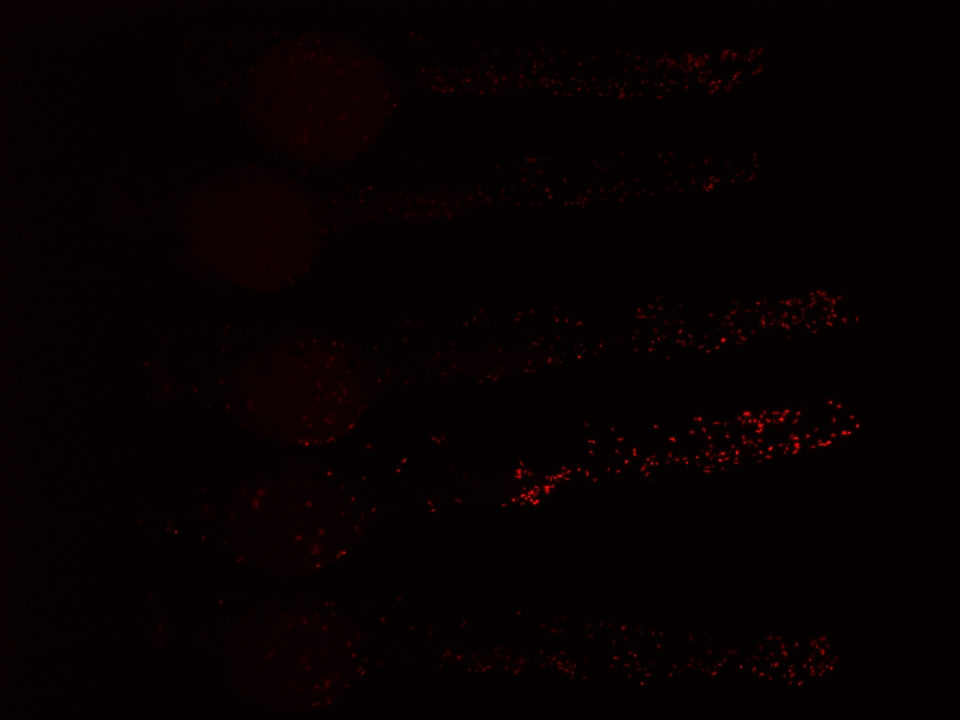

Supplement: Supplementary file 8 — Source data Fig. 4 [file 44321_2025_368_MOESM8_ESM.zip › FIGURE_4/4D/DMSO (2).jpg]

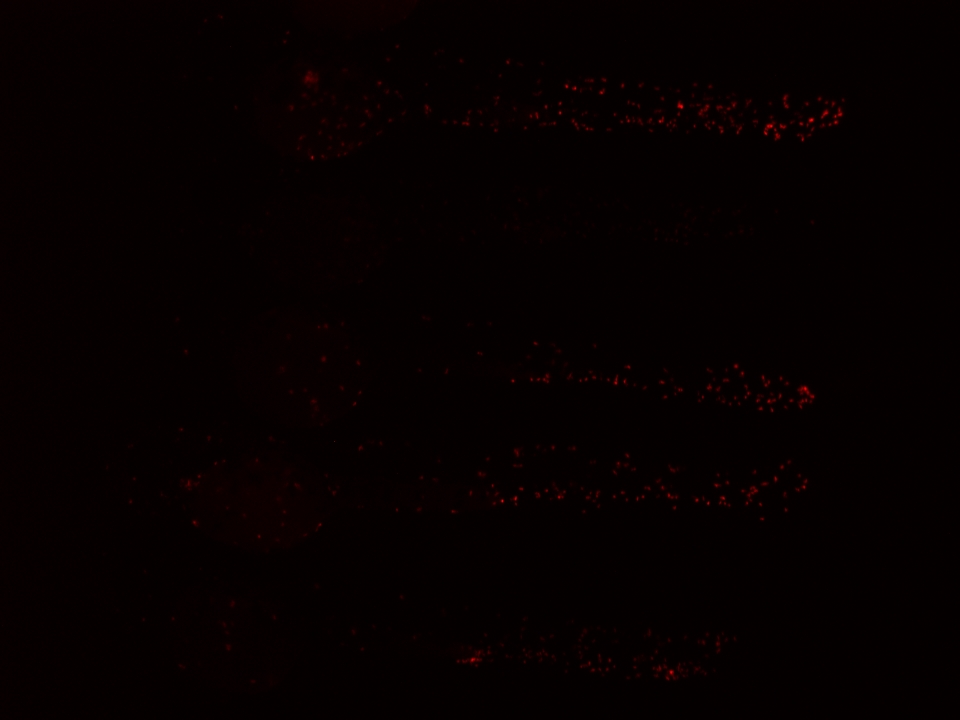

Supplement: Supplementary file 8 — Source data Fig. 4 [file 44321_2025_368_MOESM8_ESM.zip › FIGURE_4/4D/DMSO (3).jpg]

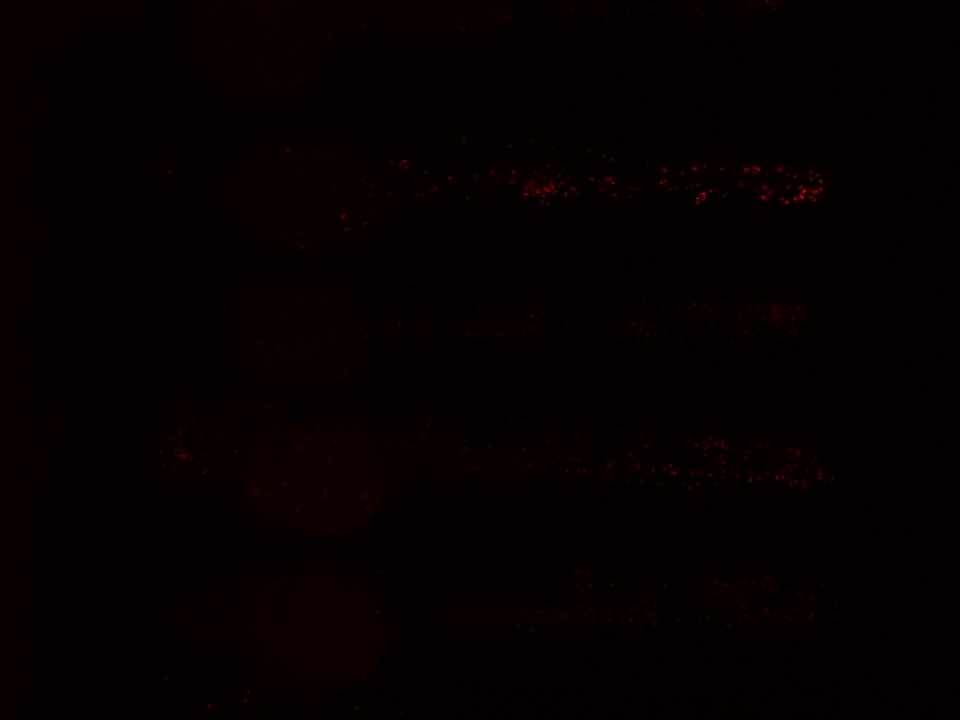

Supplement: Supplementary file 8 — Source data Fig. 4 [file 44321_2025_368_MOESM8_ESM.zip › FIGURE_4/4D/DMSO (4).jpg]

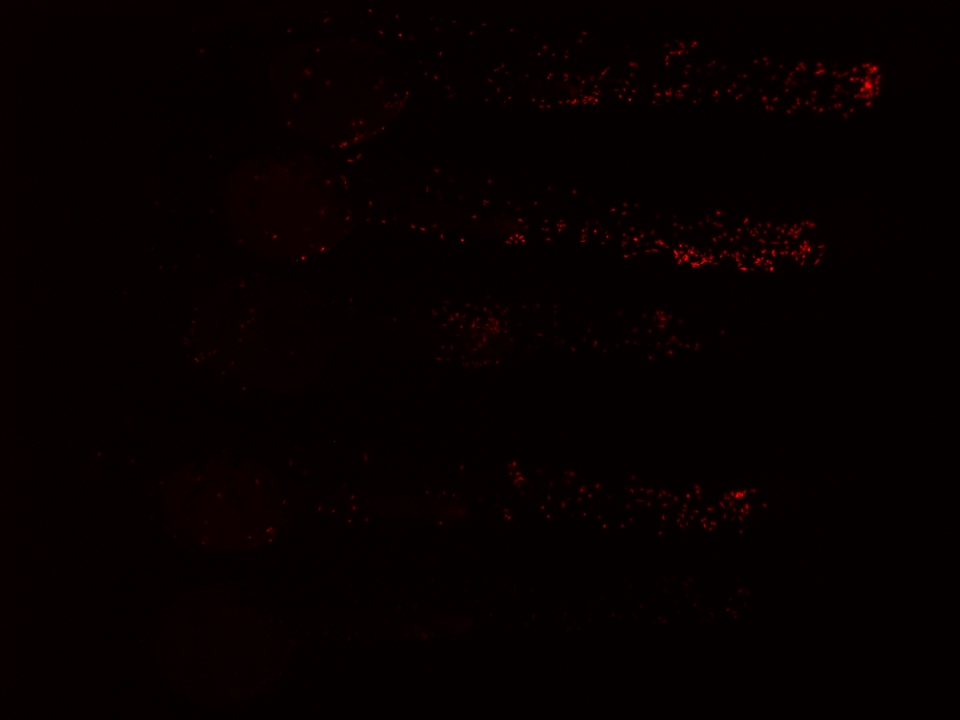

Supplement: Supplementary file 8 — Source data Fig. 4 [file 44321_2025_368_MOESM8_ESM.zip › FIGURE_4/4D/DMSO (5).jpg]

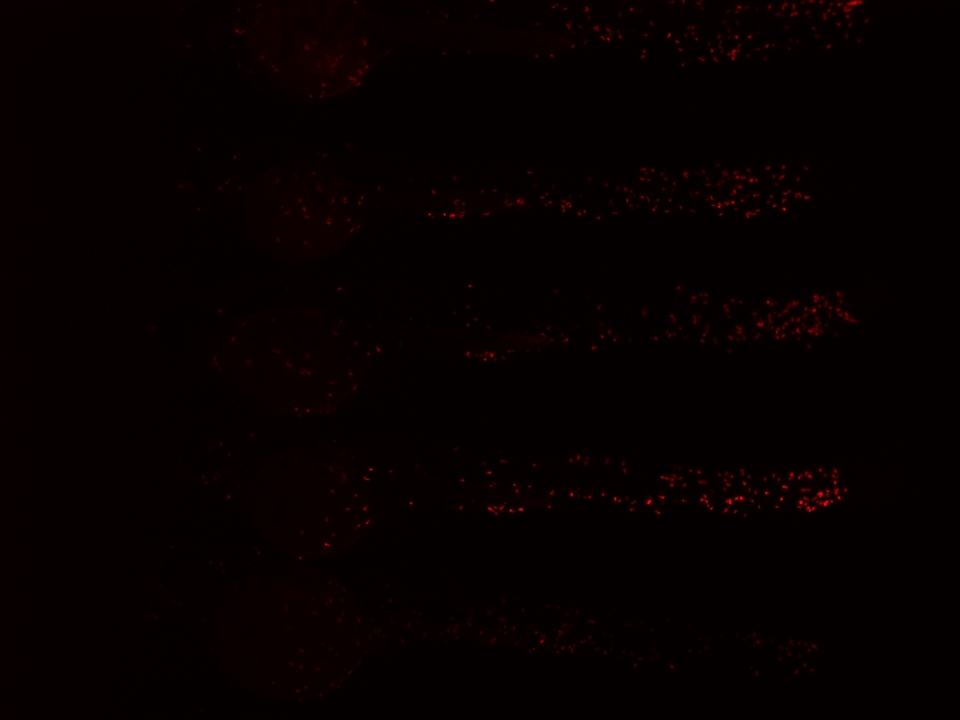

Supplement: Supplementary file 8 — Source data Fig. 4 [file 44321_2025_368_MOESM8_ESM.zip › FIGURE_4/4D/DMSO (6).jpg]

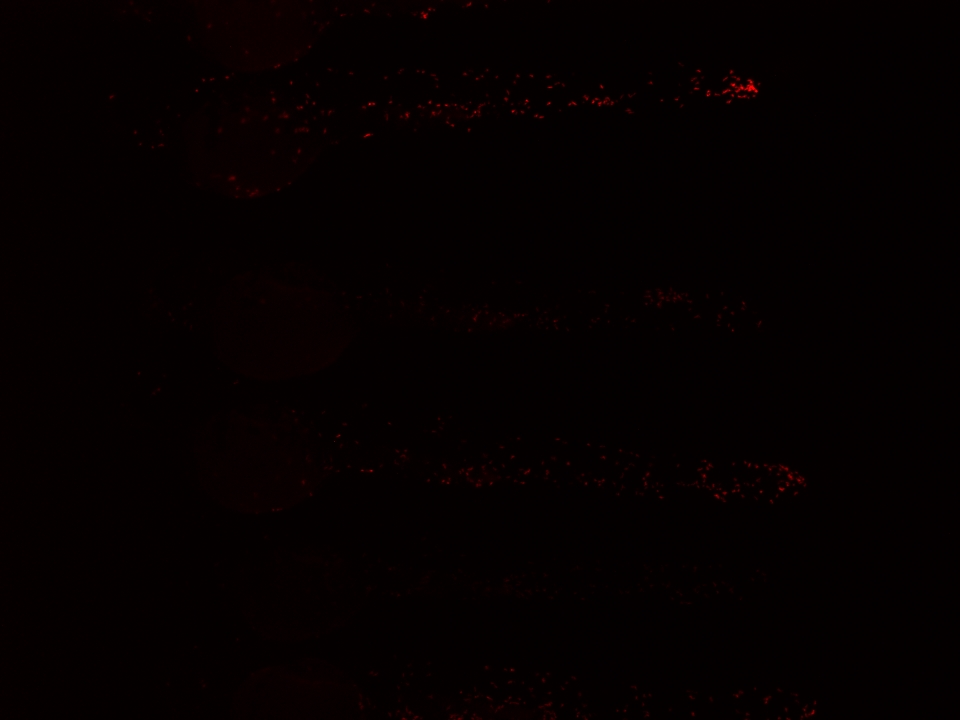

Supplement: Supplementary file 8 — Source data Fig. 4 [file 44321_2025_368_MOESM8_ESM.zip › FIGURE_4/4D/IMATINIB_1uM (1).jpg]

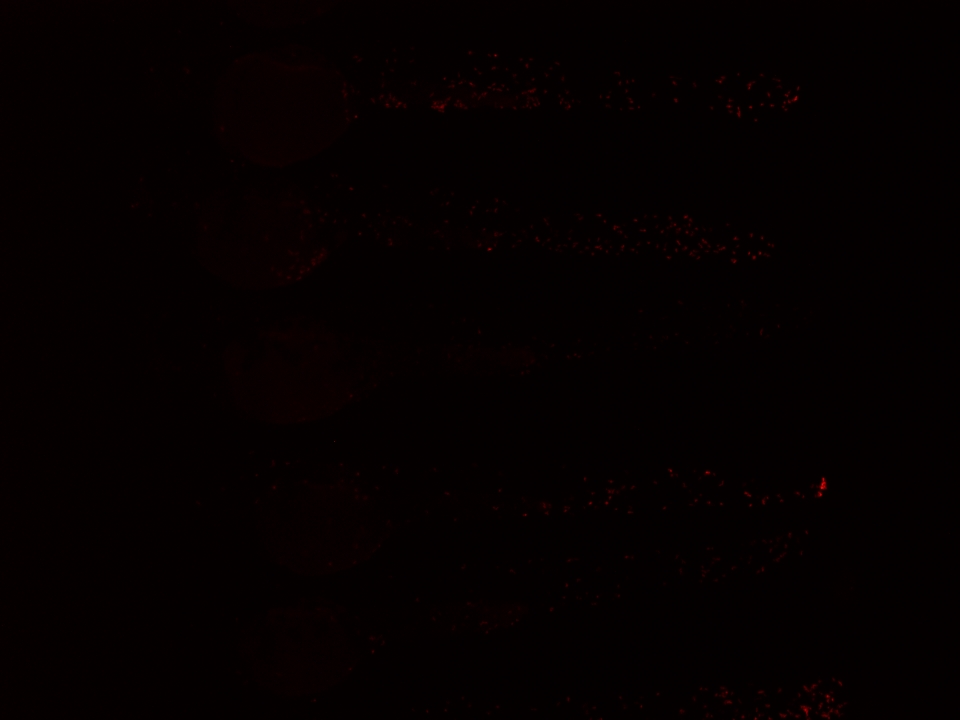

Supplement: Supplementary file 8 — Source data Fig. 4 [file 44321_2025_368_MOESM8_ESM.zip › FIGURE_4/4D/IMATINIB_1uM (2).jpg]

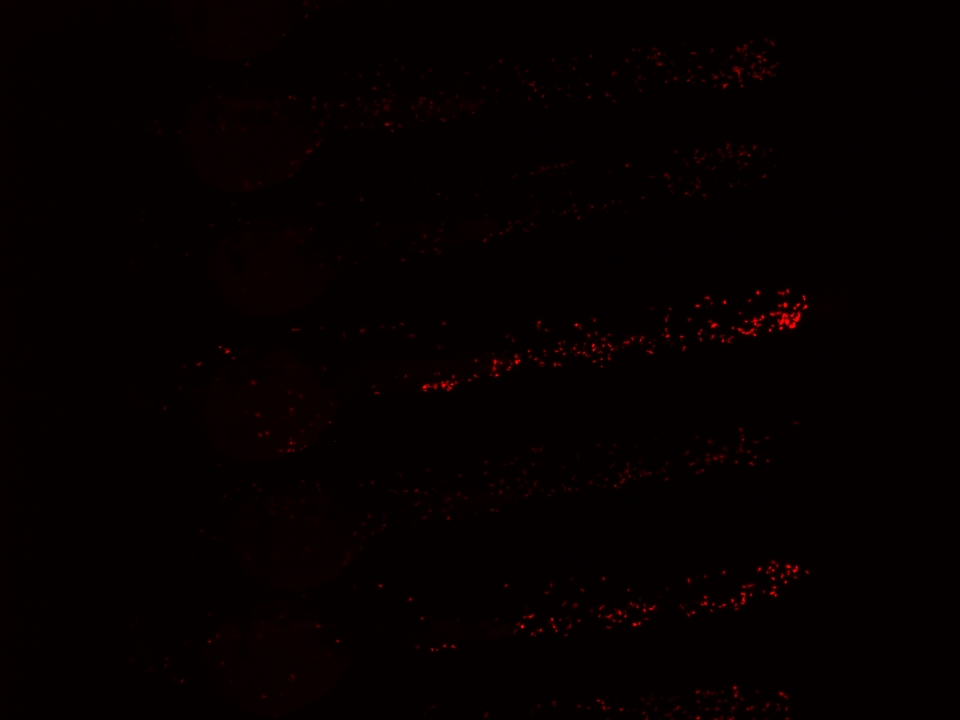

Supplement: Supplementary file 8 — Source data Fig. 4 [file 44321_2025_368_MOESM8_ESM.zip › FIGURE_4/4D/IMATINIB_1uM (3).jpg]

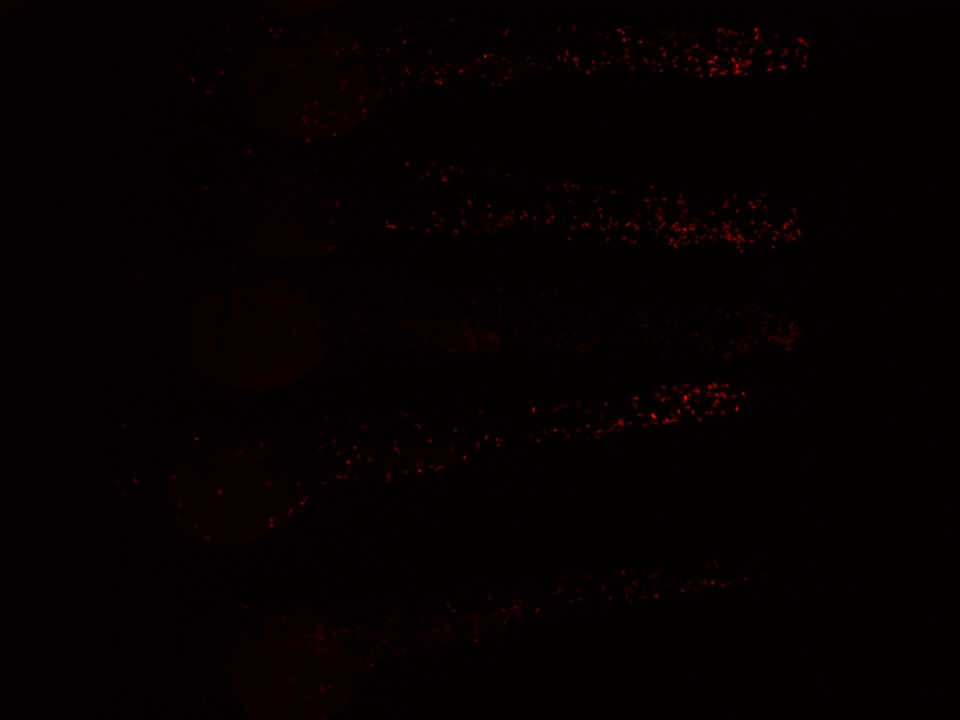

Supplement: Supplementary file 8 — Source data Fig. 4 [file 44321_2025_368_MOESM8_ESM.zip › FIGURE_4/4D/IMATINIB_1uM (4).jpg]

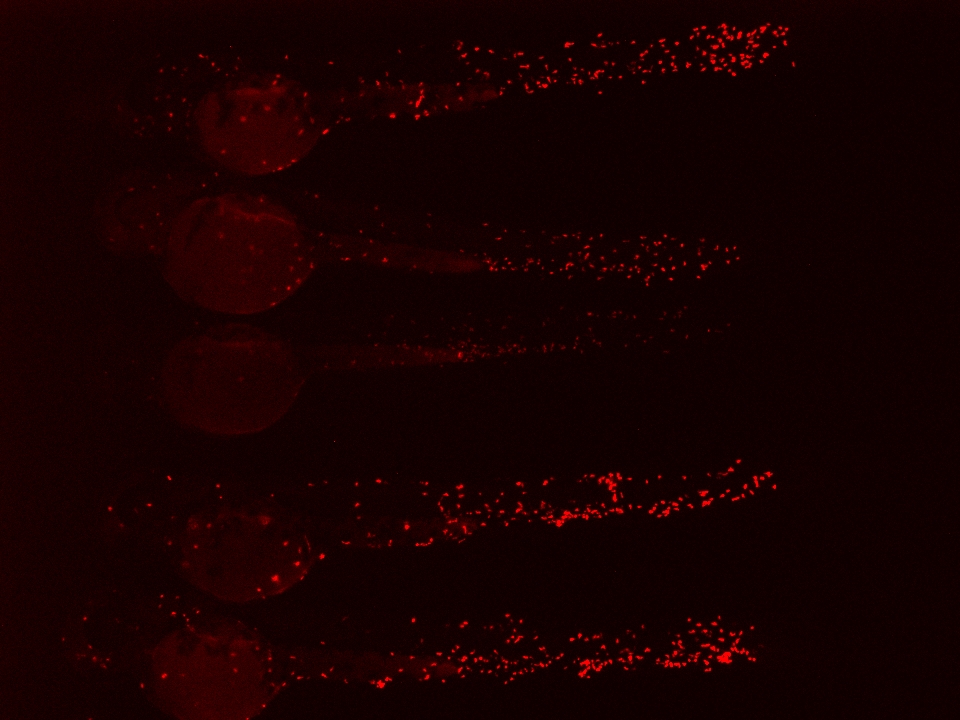

Supplement: Supplementary file 8 — Source data Fig. 4 [file 44321_2025_368_MOESM8_ESM.zip › FIGURE_4/4D/IMATINIB_1uM (5).jpg]

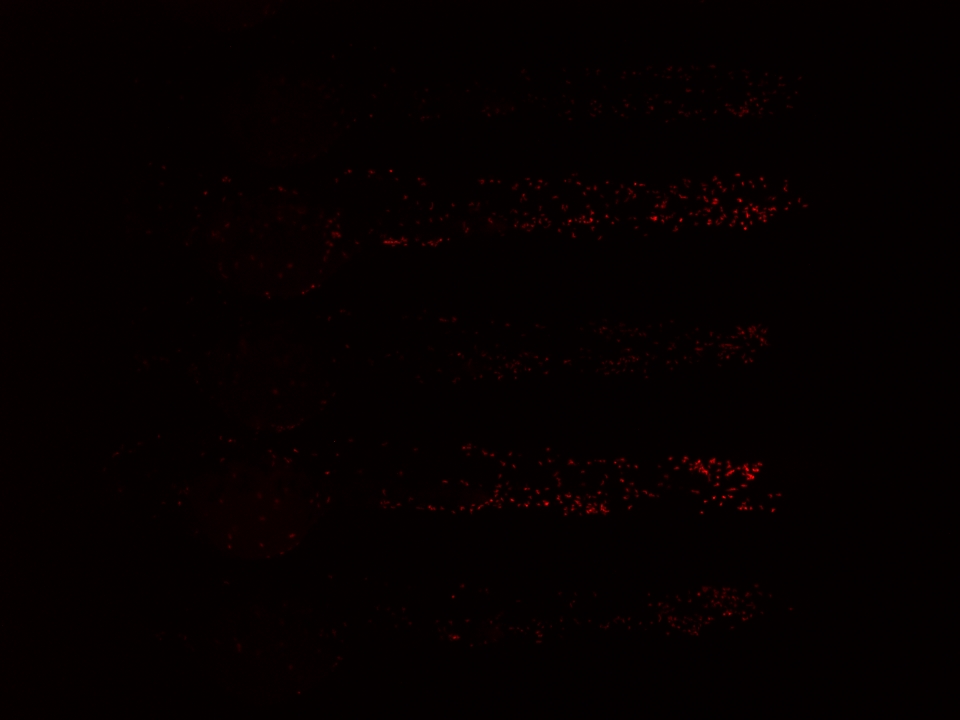

Supplement: Supplementary file 8 — Source data Fig. 4 [file 44321_2025_368_MOESM8_ESM.zip › FIGURE_4/4D/PONATINIB_01uM (1).jpg]

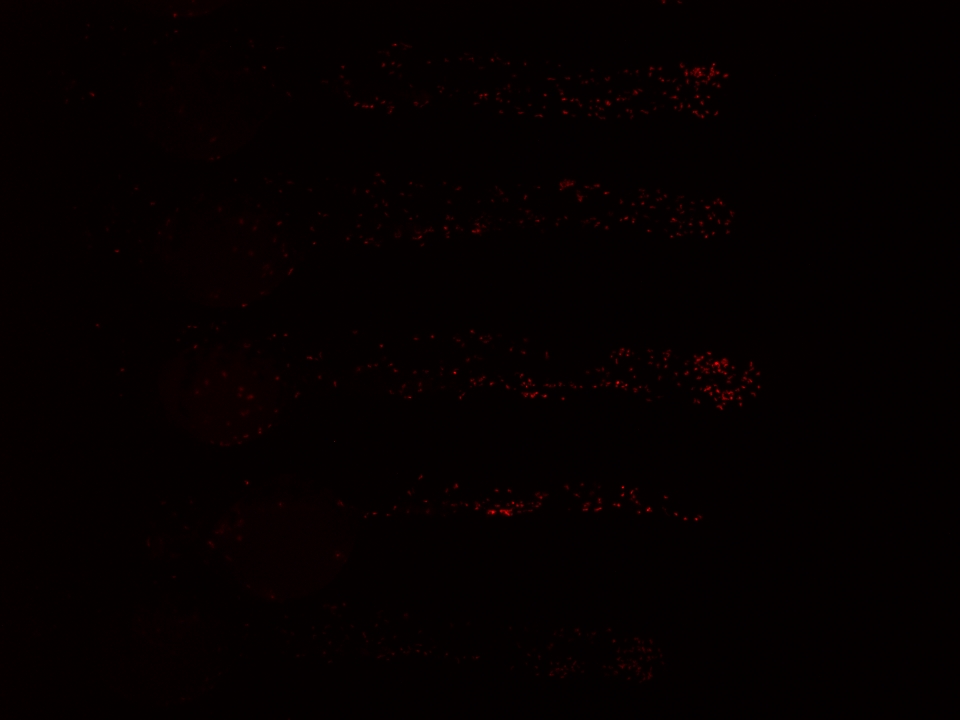

Supplement: Supplementary file 8 — Source data Fig. 4 [file 44321_2025_368_MOESM8_ESM.zip › FIGURE_4/4D/PONATINIB_01uM (2).jpg]

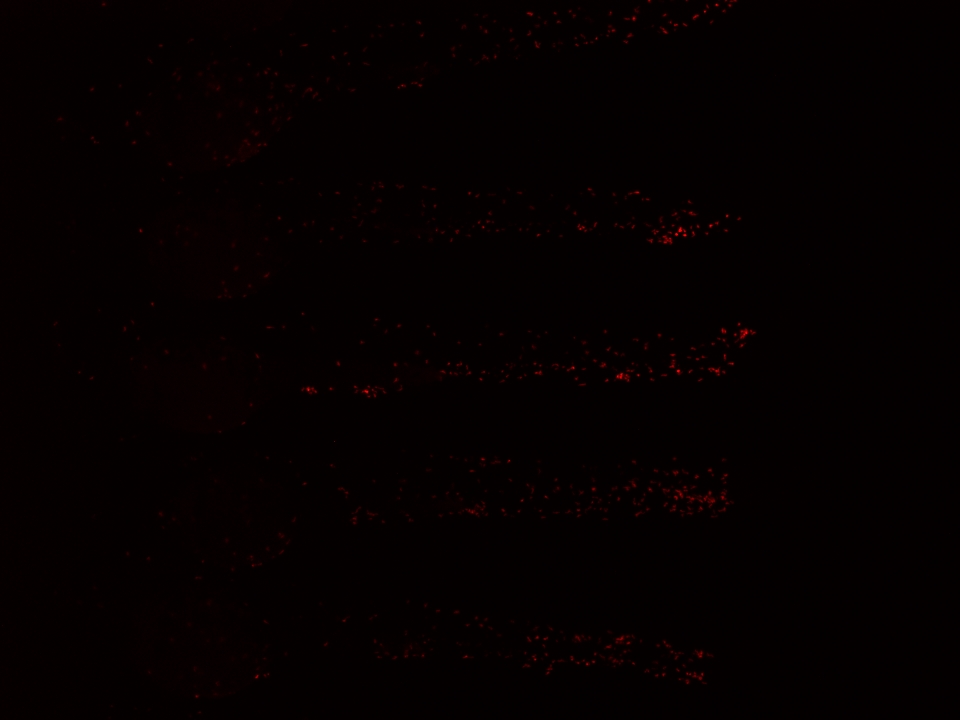

Supplement: Supplementary file 8 — Source data Fig. 4 [file 44321_2025_368_MOESM8_ESM.zip › FIGURE_4/4D/PONATINIB_01uM (3).jpg]

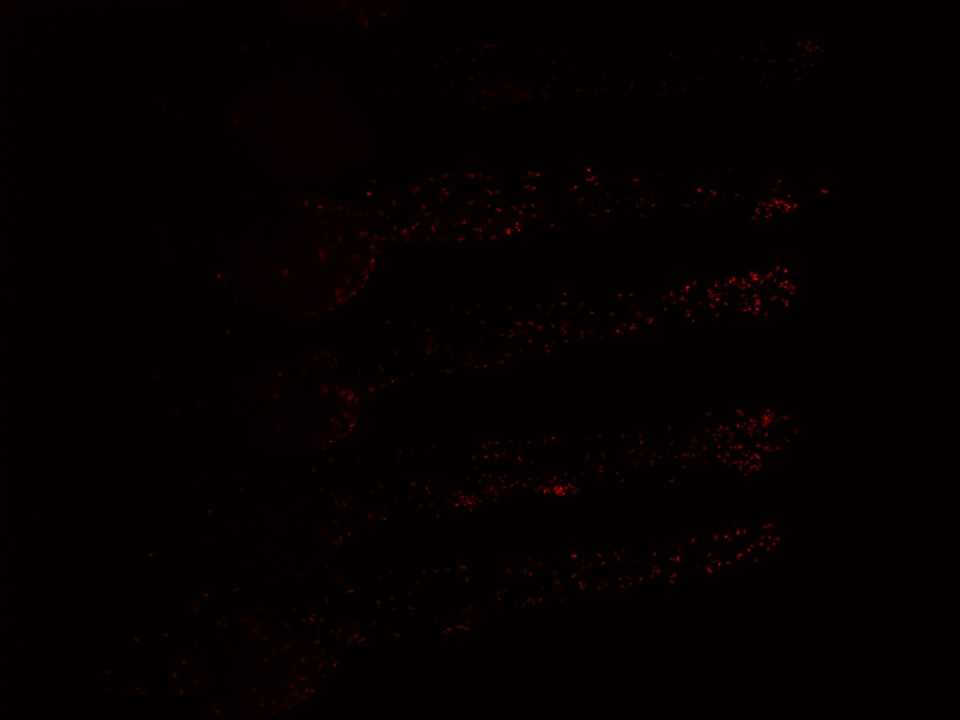

Supplement: Supplementary file 8 — Source data Fig. 4 [file 44321_2025_368_MOESM8_ESM.zip › FIGURE_4/4D/PONATINIB_01uM (4).jpg]

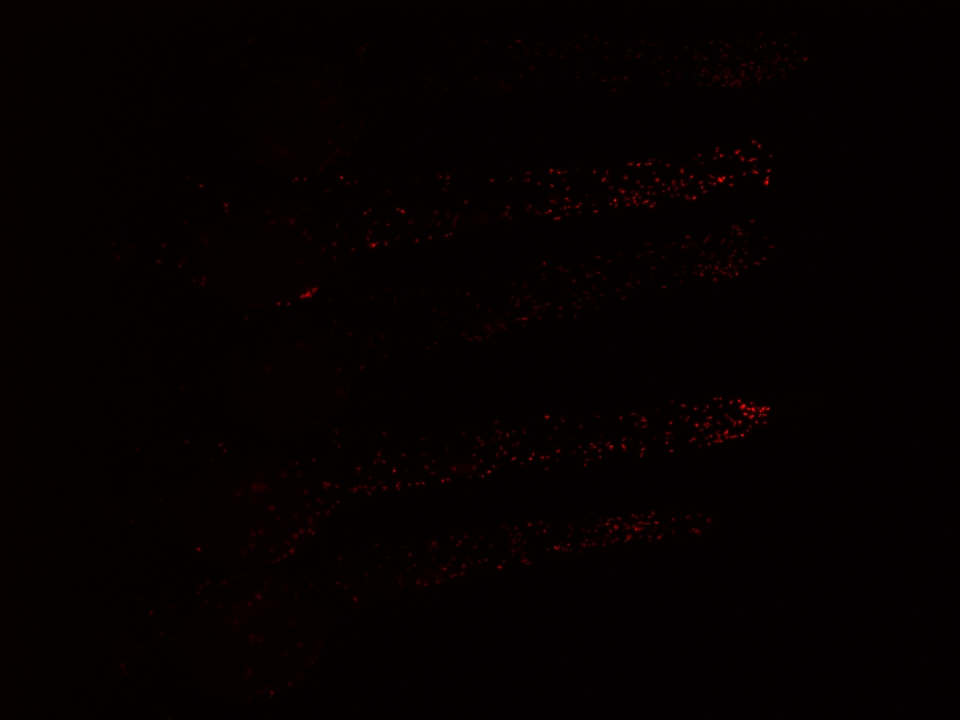

Supplement: Supplementary file 8 — Source data Fig. 4 [file 44321_2025_368_MOESM8_ESM.zip › FIGURE_4/4D/PONATINIB_01uM (5).jpg]

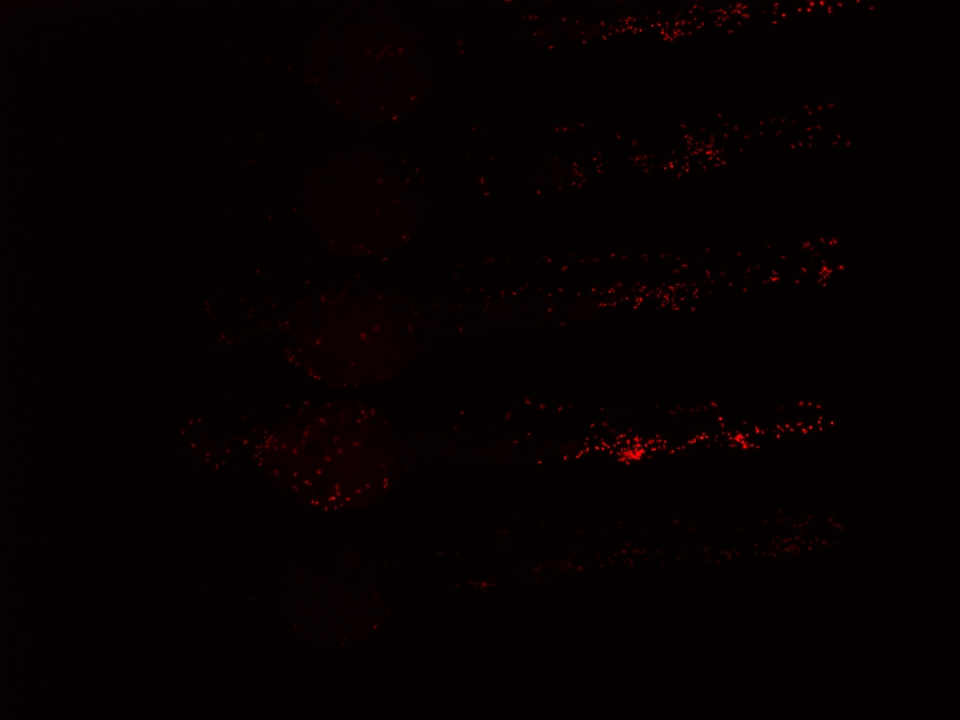

Supplement: Supplementary file 8 — Source data Fig. 4 [file 44321_2025_368_MOESM8_ESM.zip › FIGURE_4/4D/PONATINIB_1uM (1).jpg]

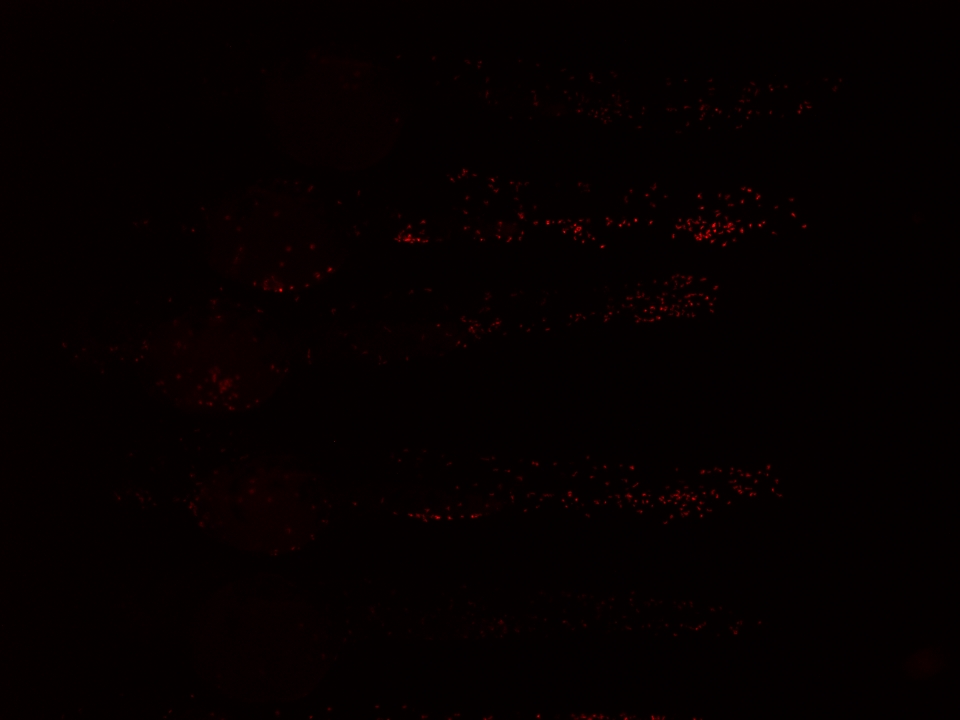

Supplement: Supplementary file 8 — Source data Fig. 4 [file 44321_2025_368_MOESM8_ESM.zip › FIGURE_4/4D/PONATINIB_1uM (2).jpg]

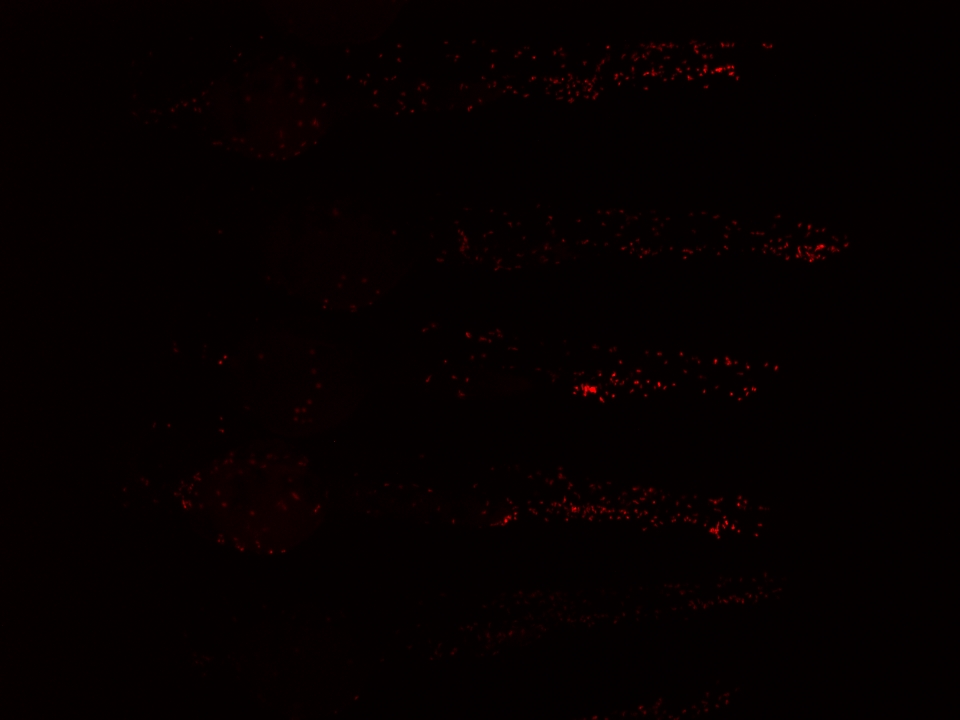

Supplement: Supplementary file 8 — Source data Fig. 4 [file 44321_2025_368_MOESM8_ESM.zip › FIGURE_4/4D/PONATINIB_1uM (3).jpg]

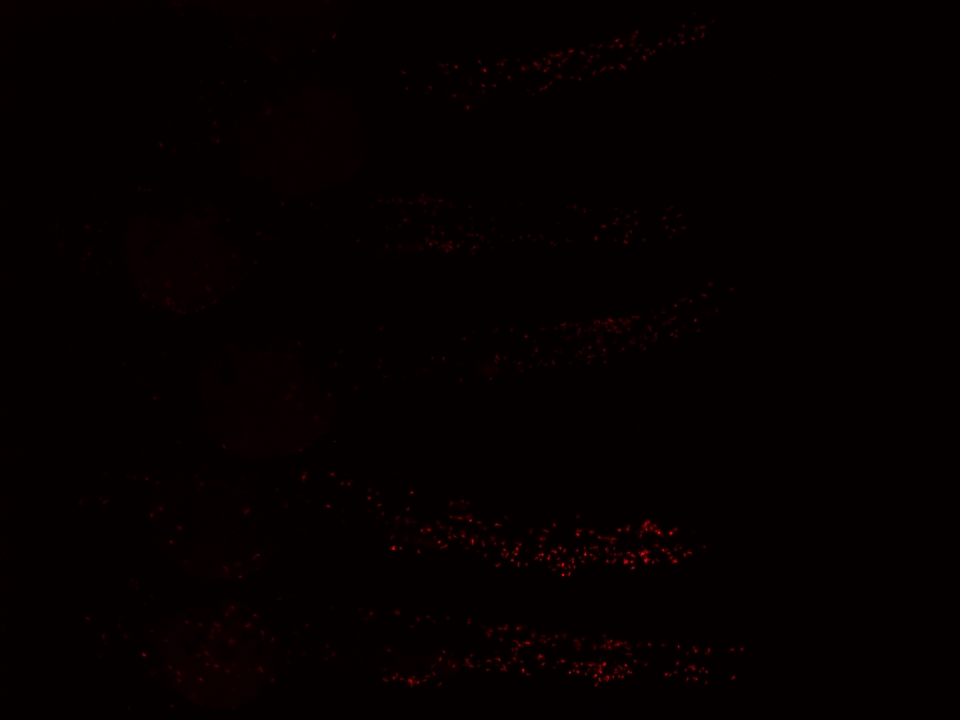

Supplement: Supplementary file 8 — Source data Fig. 4 [file 44321_2025_368_MOESM8_ESM.zip › FIGURE_4/4D/PONATINIB_1uM (4).jpg]

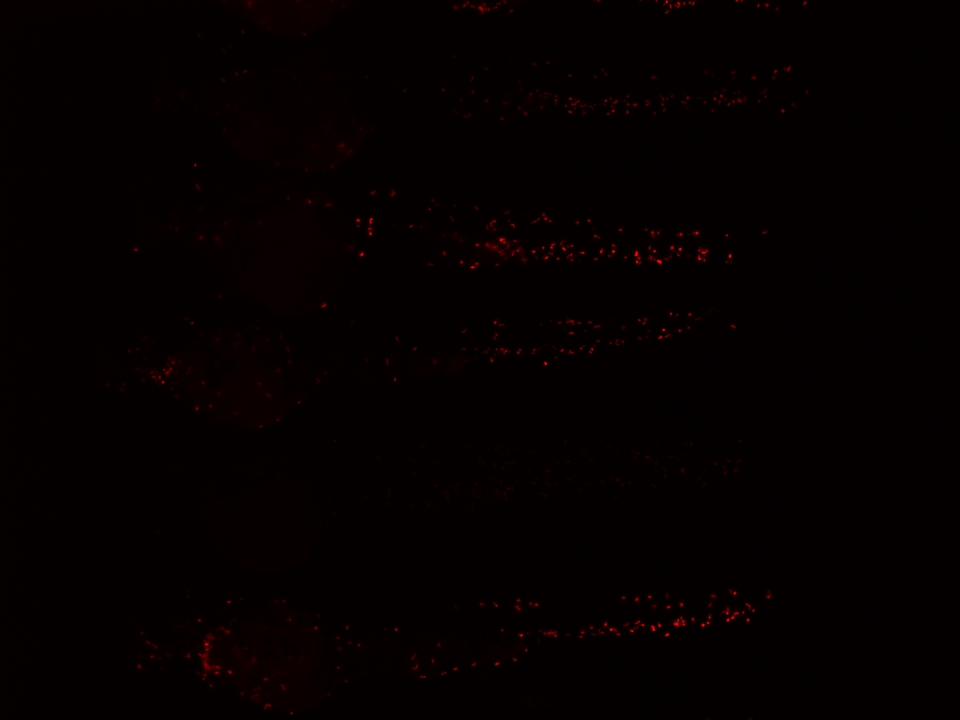

Supplement: Supplementary file 8 — Source data Fig. 4 [file 44321_2025_368_MOESM8_ESM.zip › FIGURE_4/4D/PONATINIB_1uM (5).jpg]

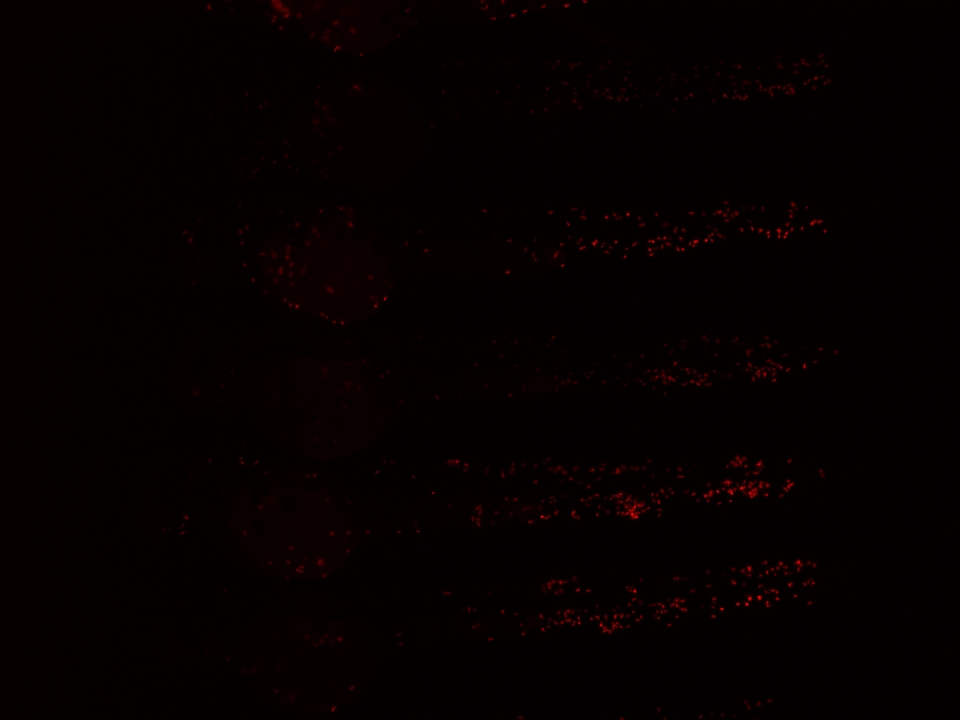

Supplement: Supplementary file 8 — Source data Fig. 4 [file 44321_2025_368_MOESM8_ESM.zip › FIGURE_4/4D/PONATINIB_1uM (6).jpg]

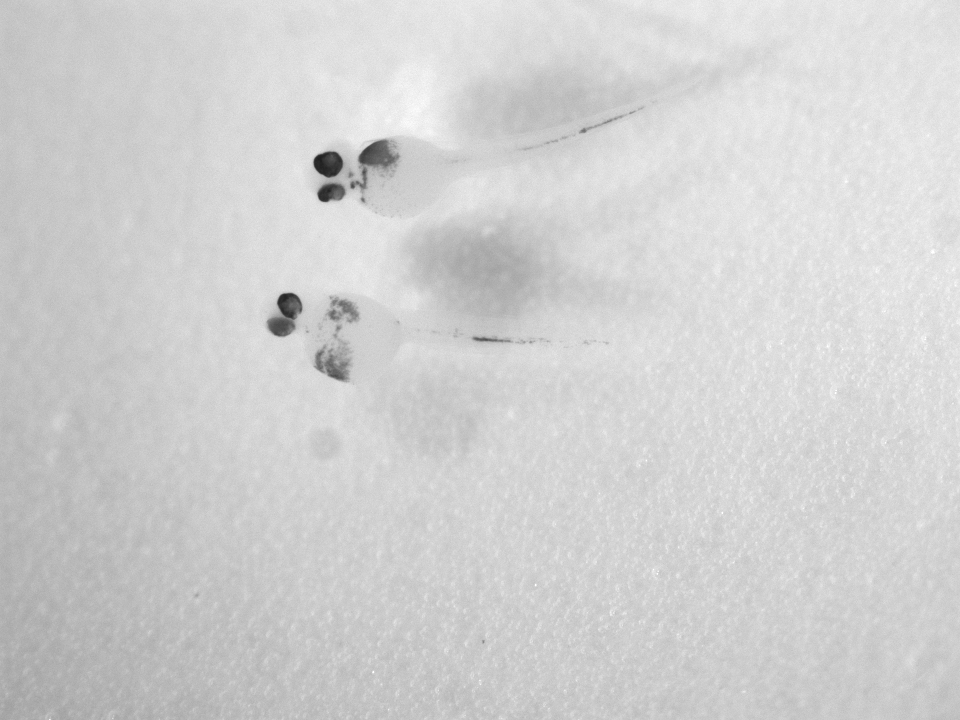

Supplement: Supplementary file 8 — Source data Fig. 4 [file 44321_2025_368_MOESM8_ESM.zip › FIGURE_4/4F/crRNA_rps19_nlrp1 (1).tif]

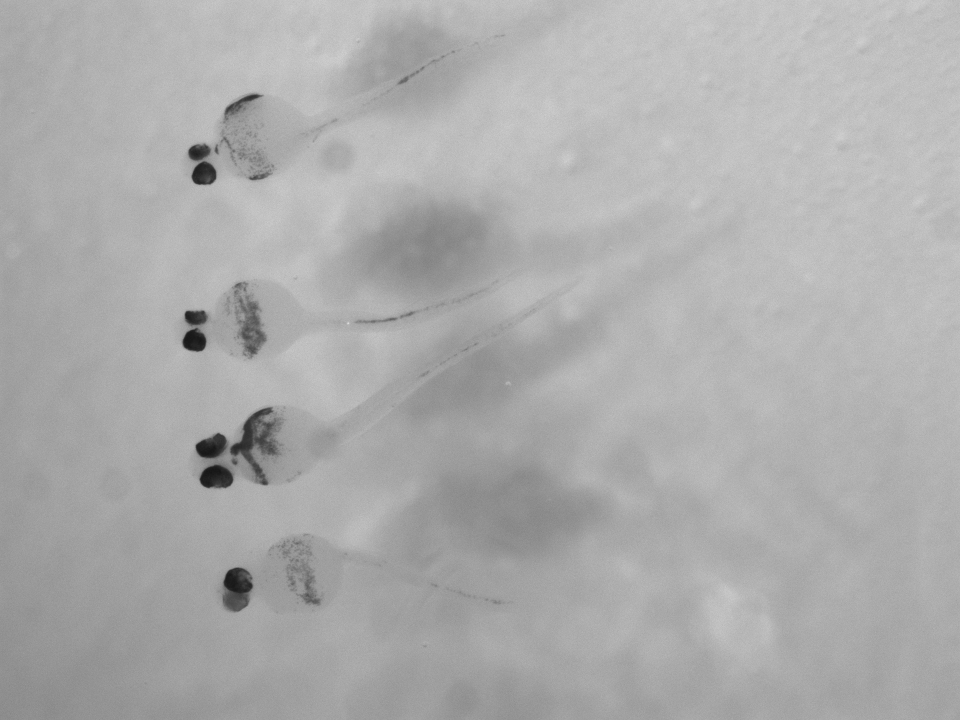

Supplement: Supplementary file 8 — Source data Fig. 4 [file 44321_2025_368_MOESM8_ESM.zip › FIGURE_4/4F/crRNA_rps19_nlrp1 (2).tif]

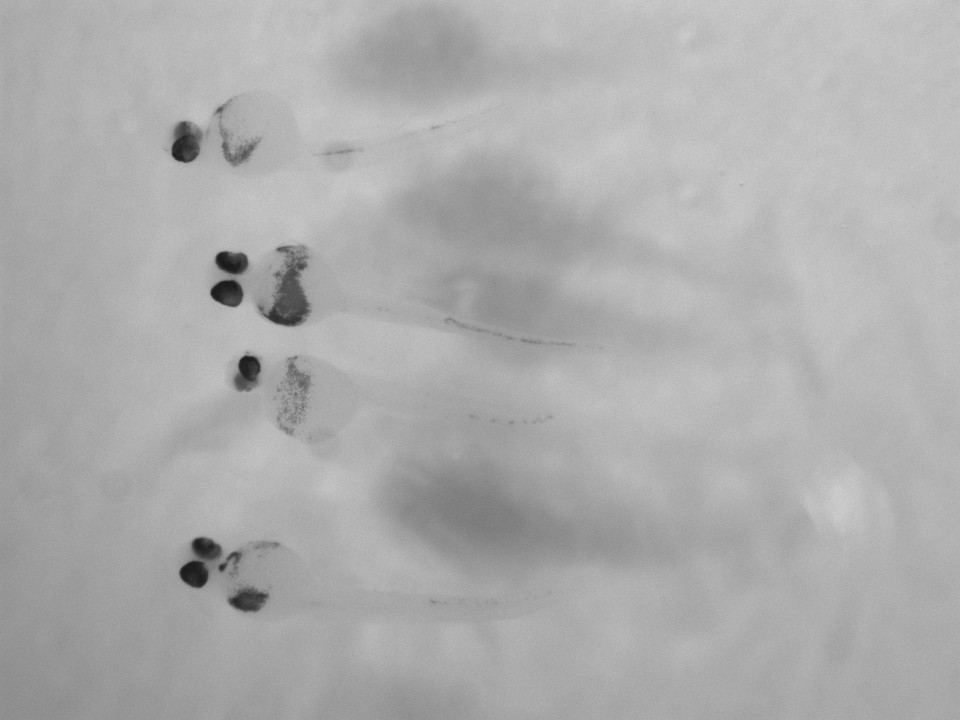

Supplement: Supplementary file 8 — Source data Fig. 4 [file 44321_2025_368_MOESM8_ESM.zip › FIGURE_4/4F/crRNA_rps19_nlrp1 (3).tif]

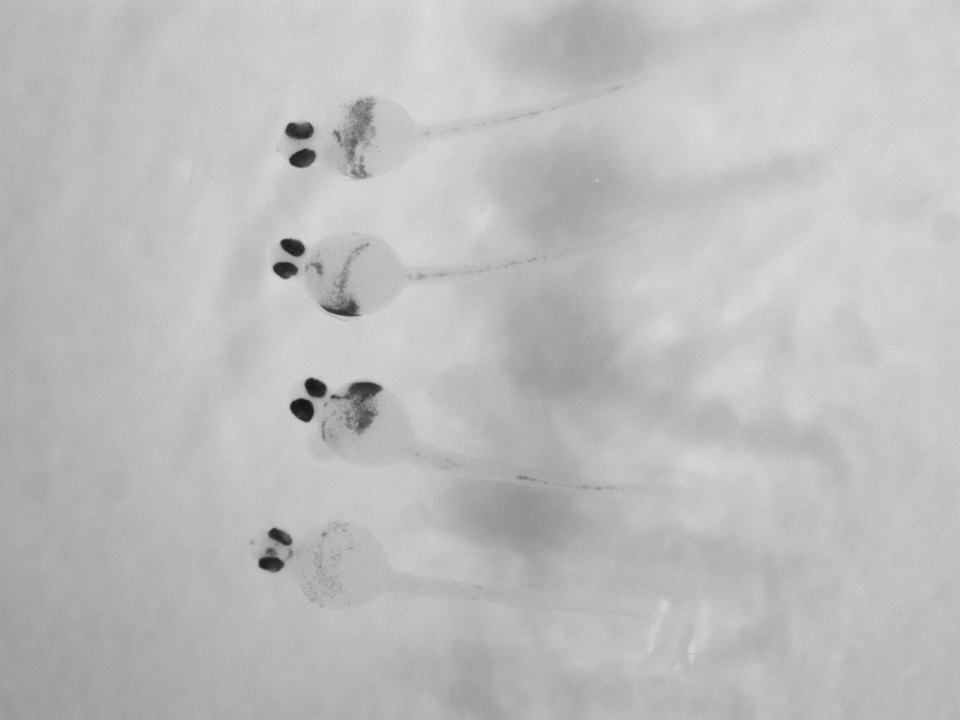

Supplement: Supplementary file 8 — Source data Fig. 4 [file 44321_2025_368_MOESM8_ESM.zip › FIGURE_4/4F/crRNA_rps19_nlrp1 (4).tif]

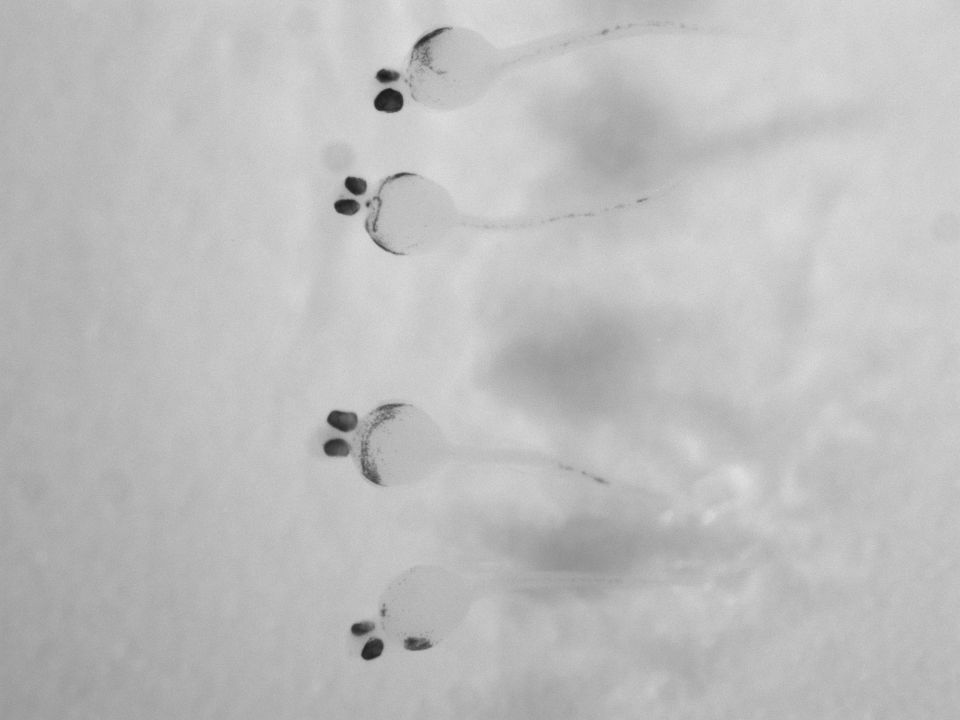

Supplement: Supplementary file 8 — Source data Fig. 4 [file 44321_2025_368_MOESM8_ESM.zip › FIGURE_4/4F/crRNA_rps19_nlrp1 (5).tif]

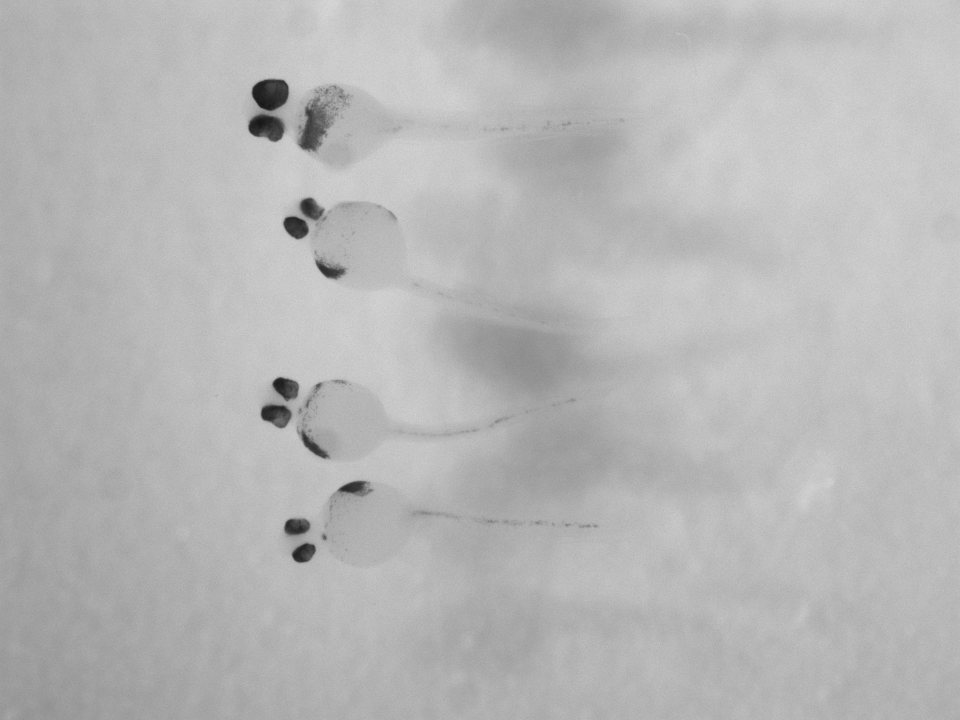

Supplement: Supplementary file 8 — Source data Fig. 4 [file 44321_2025_368_MOESM8_ESM.zip › FIGURE_4/4F/crRNA_rps19_nlrp1 (6).tif]

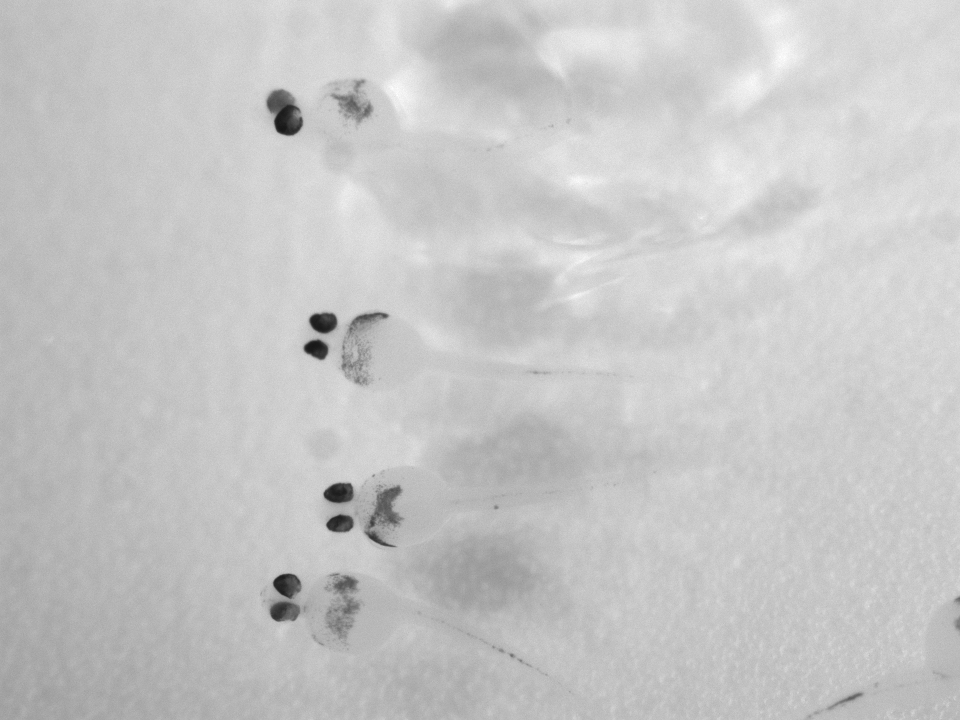

Supplement: Supplementary file 8 — Source data Fig. 4 [file 44321_2025_368_MOESM8_ESM.zip › FIGURE_4/4F/crRNA_rps19_nlrp1 (7).tif]

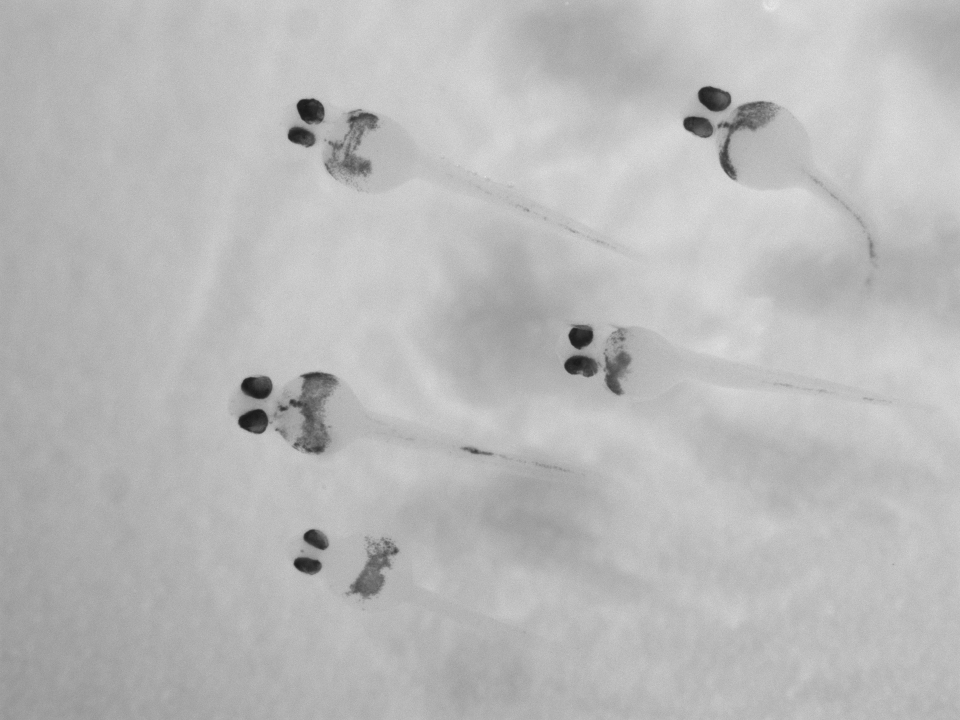

Supplement: Supplementary file 8 — Source data Fig. 4 [file 44321_2025_368_MOESM8_ESM.zip › FIGURE_4/4F/crRNA_rps19_std (1).tif]

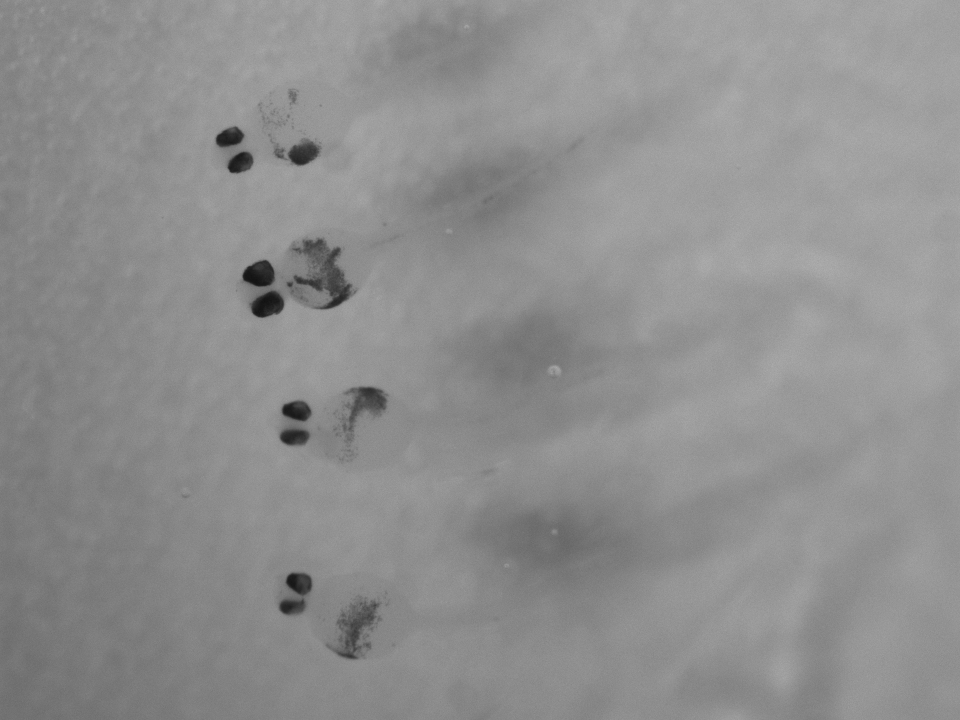

Supplement: Supplementary file 8 — Source data Fig. 4 [file 44321_2025_368_MOESM8_ESM.zip › FIGURE_4/4F/crRNA_rps19_std (2).tif]

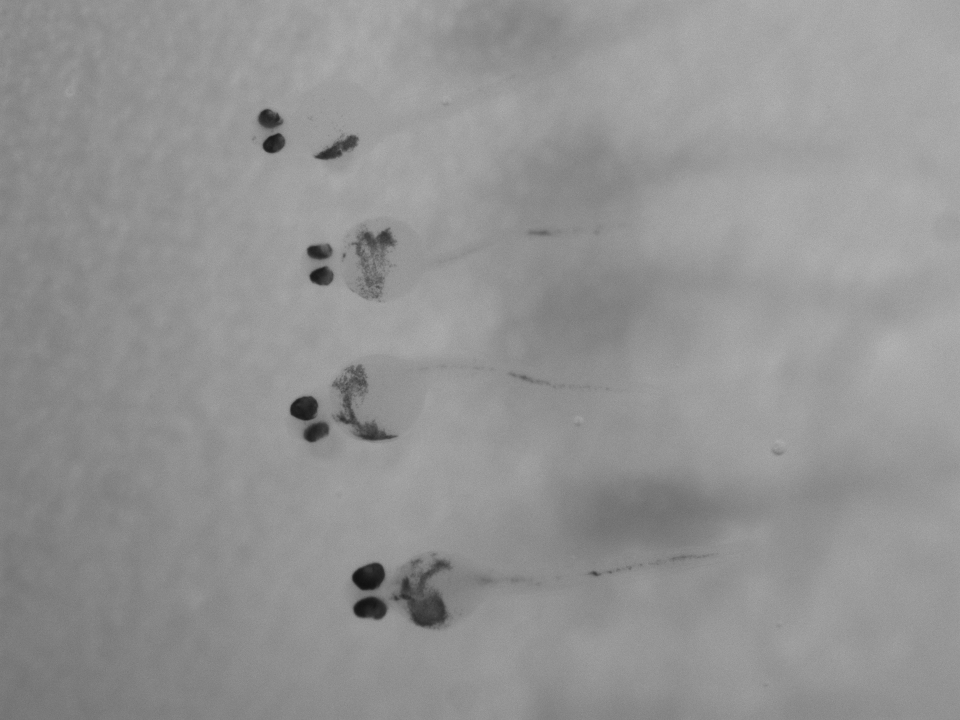

Supplement: Supplementary file 8 — Source data Fig. 4 [file 44321_2025_368_MOESM8_ESM.zip › FIGURE_4/4F/crRNA_rps19_std (3).tif]

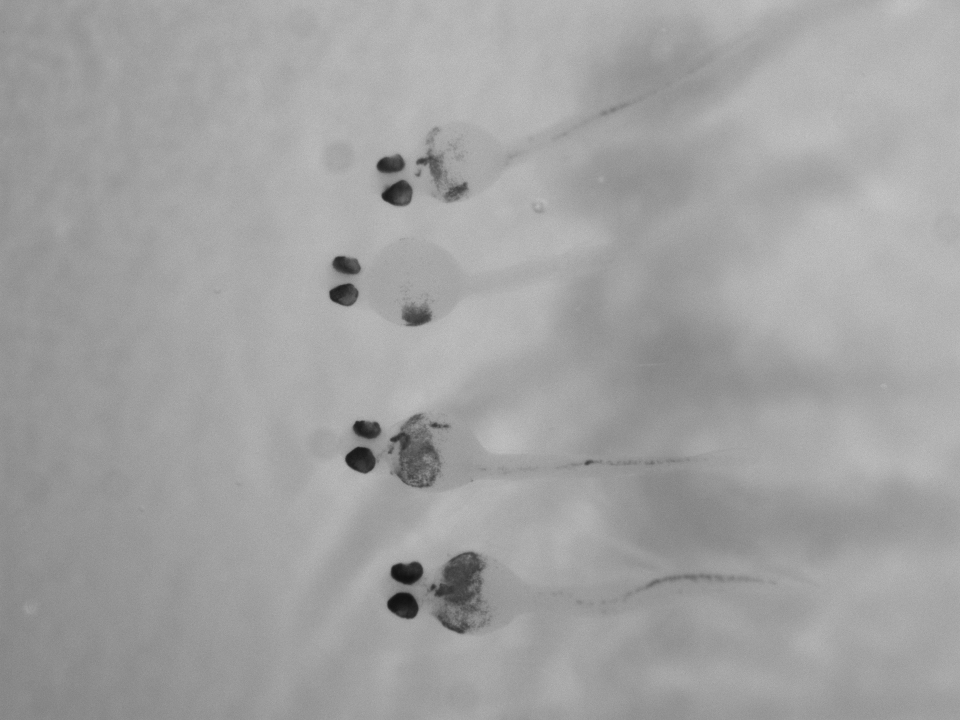

Supplement: Supplementary file 8 — Source data Fig. 4 [file 44321_2025_368_MOESM8_ESM.zip › FIGURE_4/4F/crRNA_rps19_std (4).tif]

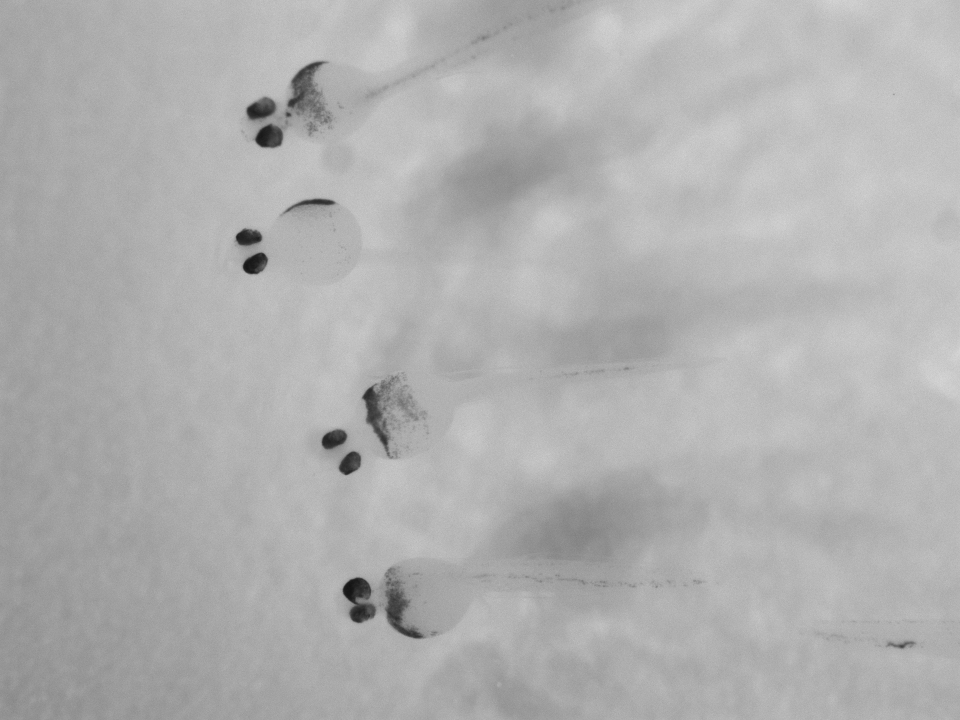

Supplement: Supplementary file 8 — Source data Fig. 4 [file 44321_2025_368_MOESM8_ESM.zip › FIGURE_4/4F/crRNA_rps19_std (5).tif]

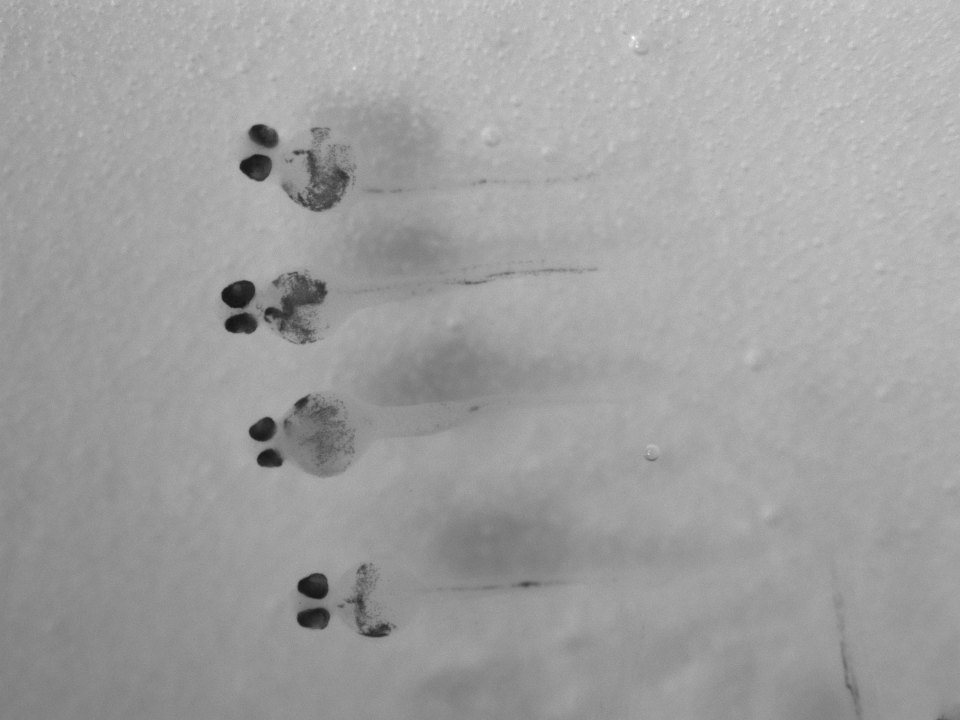

Supplement: Supplementary file 8 — Source data Fig. 4 [file 44321_2025_368_MOESM8_ESM.zip › FIGURE_4/4F/crRNA_rps19_zaka (1).tif]

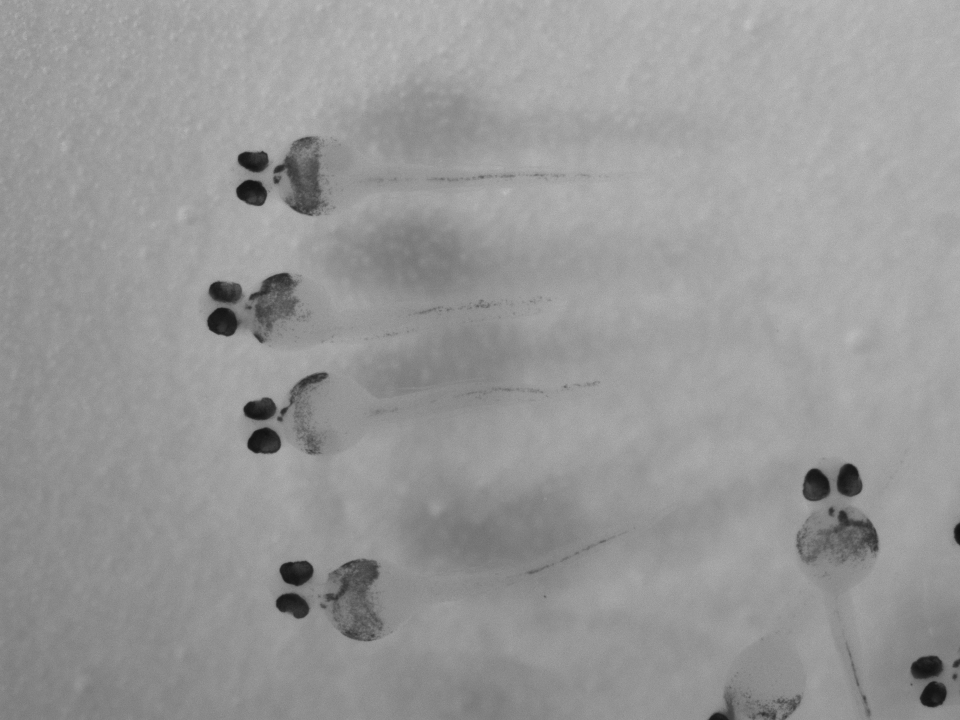

Supplement: Supplementary file 8 — Source data Fig. 4 [file 44321_2025_368_MOESM8_ESM.zip › FIGURE_4/4F/crRNA_rps19_zaka (2).tif]

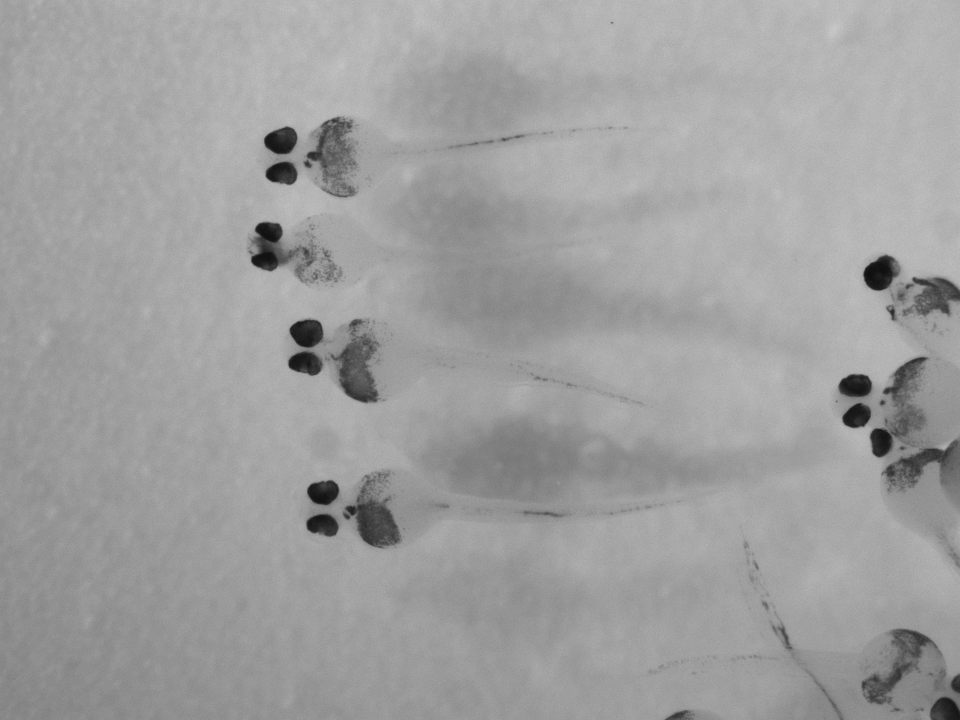

Supplement: Supplementary file 8 — Source data Fig. 4 [file 44321_2025_368_MOESM8_ESM.zip › FIGURE_4/4F/crRNA_rps19_zaka (3).tif]

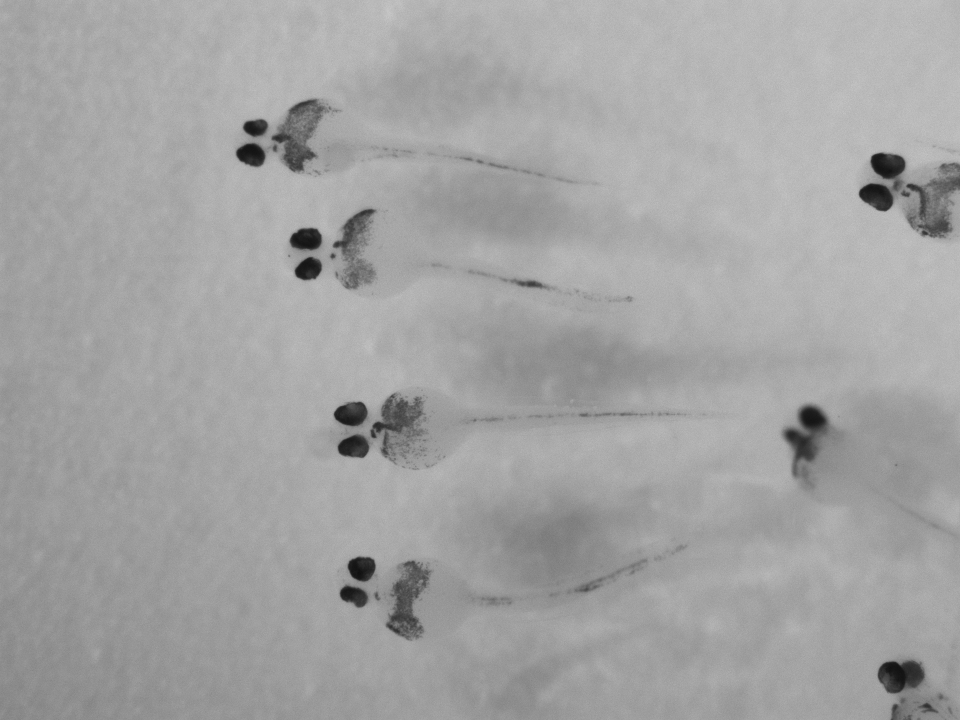

Supplement: Supplementary file 8 — Source data Fig. 4 [file 44321_2025_368_MOESM8_ESM.zip › FIGURE_4/4F/crRNA_rps19_zaka (4).tif]

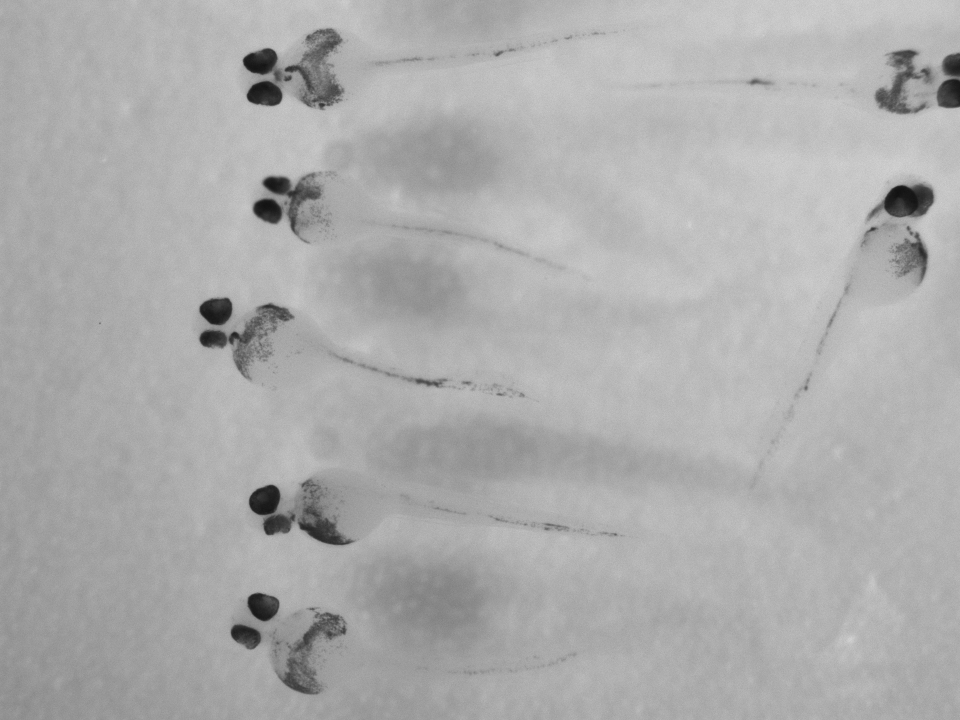

Supplement: Supplementary file 8 — Source data Fig. 4 [file 44321_2025_368_MOESM8_ESM.zip › FIGURE_4/4F/crRNA_rps19_zaka (5).tif]

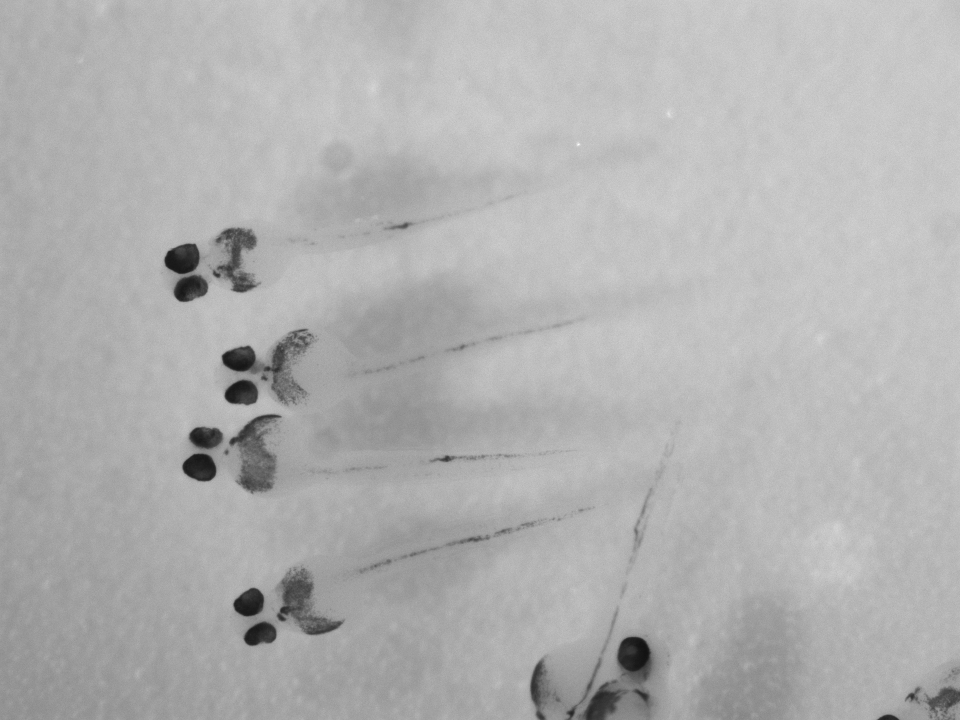

Supplement: Supplementary file 8 — Source data Fig. 4 [file 44321_2025_368_MOESM8_ESM.zip › FIGURE_4/4F/crRNA_rps19_zaka (6).tif]

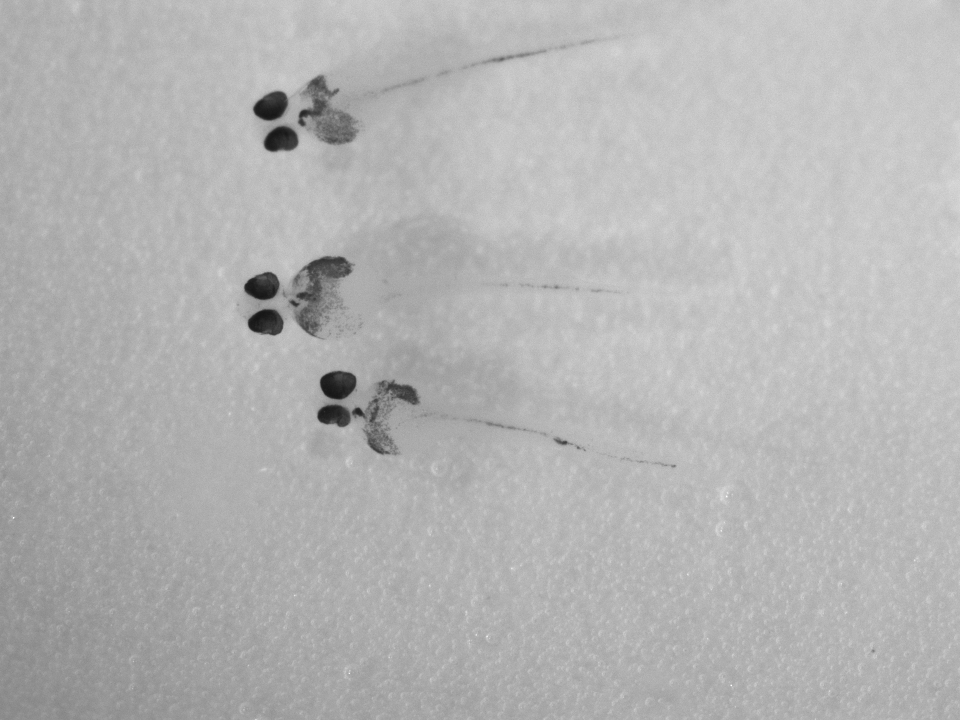

Supplement: Supplementary file 8 — Source data Fig. 4 [file 44321_2025_368_MOESM8_ESM.zip › FIGURE_4/4F/crRNA_rps19_zaka (7).tif]

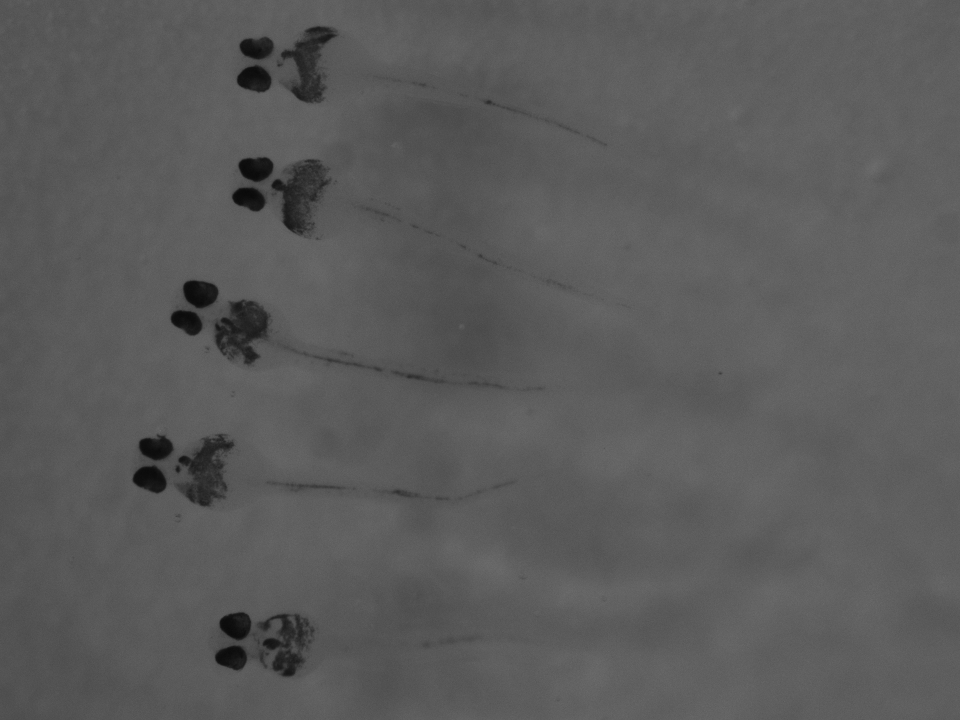

Supplement: Supplementary file 8 — Source data Fig. 4 [file 44321_2025_368_MOESM8_ESM.zip › FIGURE_4/4F/crRNA_std (1).tif]

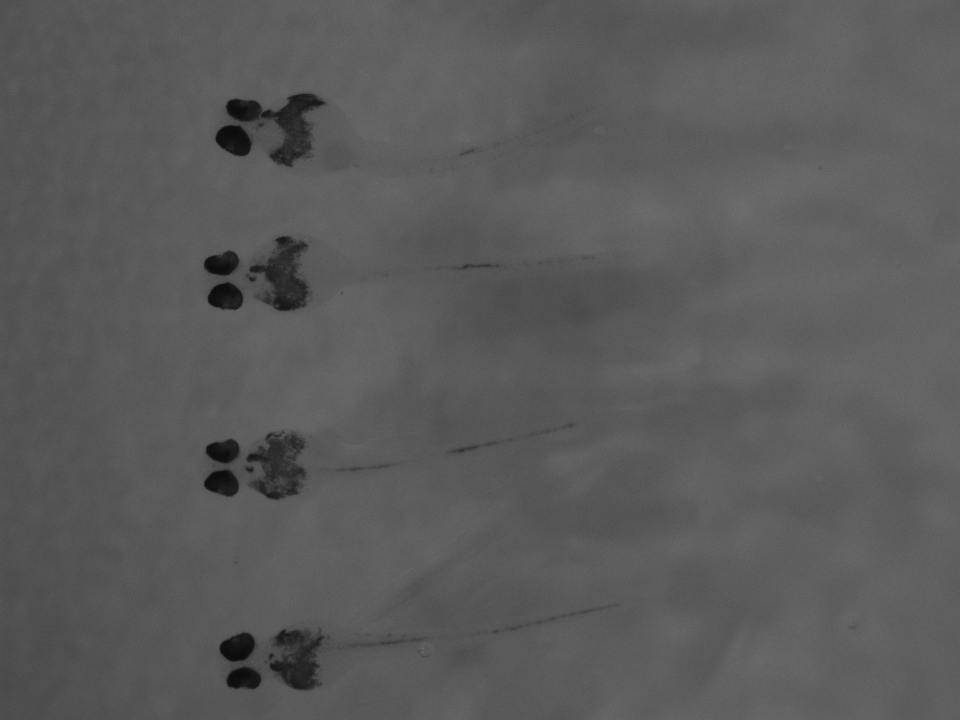

Supplement: Supplementary file 8 — Source data Fig. 4 [file 44321_2025_368_MOESM8_ESM.zip › FIGURE_4/4F/crRNA_std (2).tif]

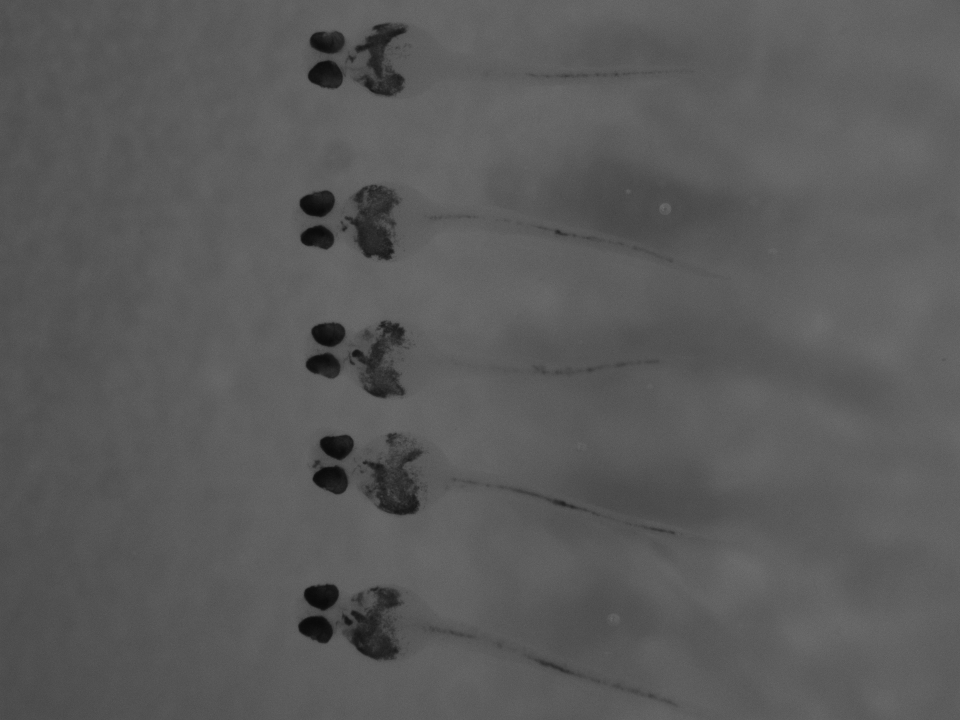

Supplement: Supplementary file 8 — Source data Fig. 4 [file 44321_2025_368_MOESM8_ESM.zip › FIGURE_4/4F/crRNA_std (3).tif]

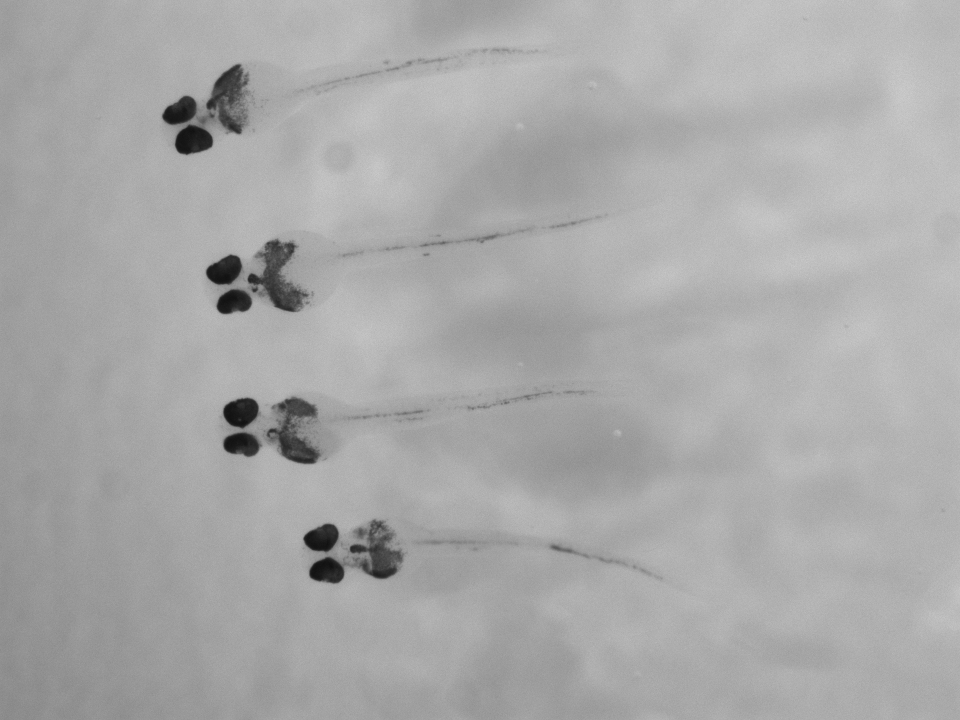

Supplement: Supplementary file 8 — Source data Fig. 4 [file 44321_2025_368_MOESM8_ESM.zip › FIGURE_4/4F/crRNA_std (4).tif]

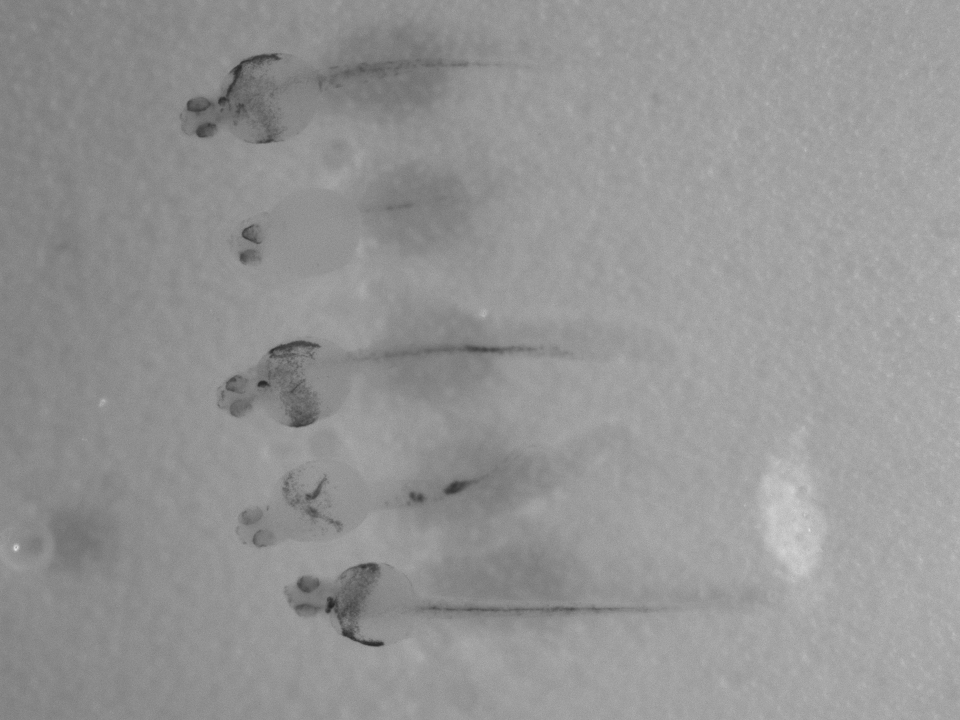

Supplement: Supplementary file 8 — Source data Fig. 4 [file 44321_2025_368_MOESM8_ESM.zip › FIGURE_4/4G/cRNA_rps19_DMSO (1).tif]

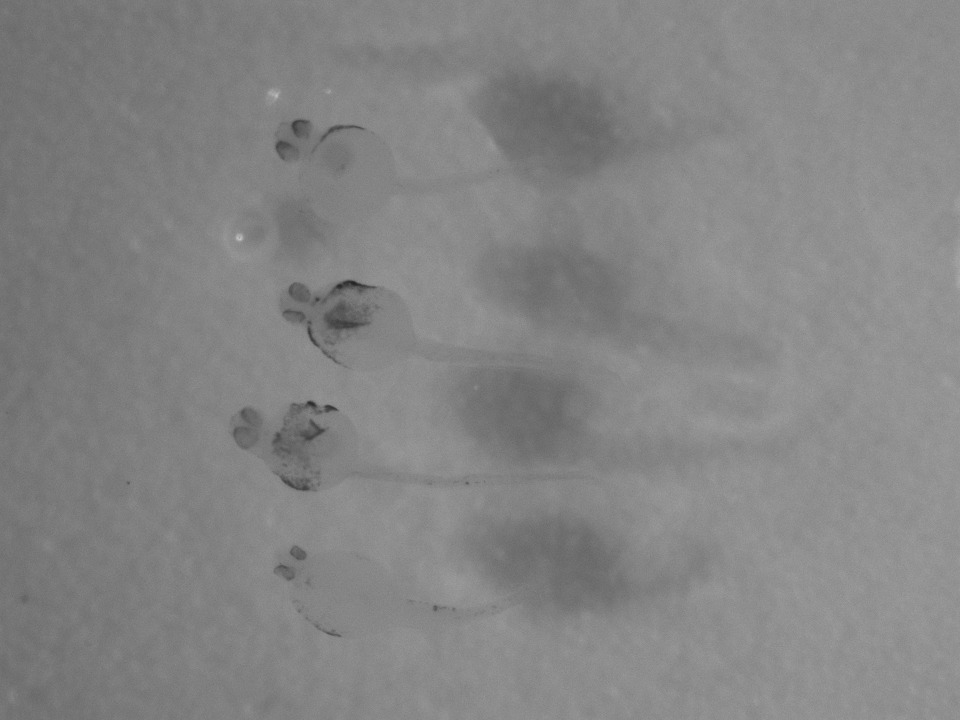

Supplement: Supplementary file 8 — Source data Fig. 4 [file 44321_2025_368_MOESM8_ESM.zip › FIGURE_4/4G/cRNA_rps19_DMSO (2).tif]

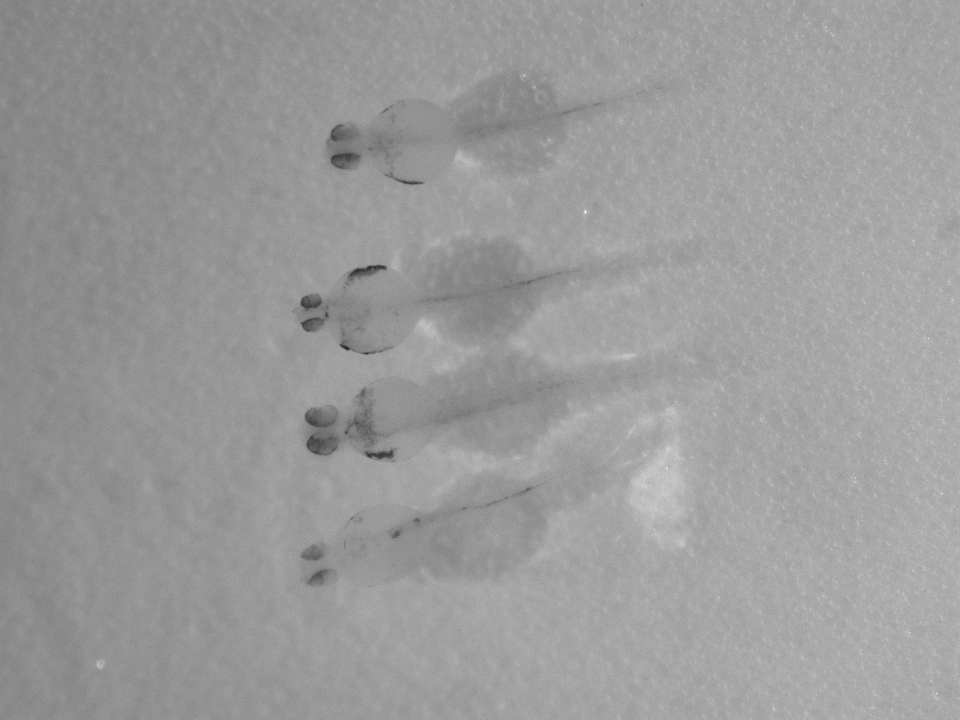

Supplement: Supplementary file 8 — Source data Fig. 4 [file 44321_2025_368_MOESM8_ESM.zip › FIGURE_4/4G/cRNA_rps19_DMSO (3).tif]

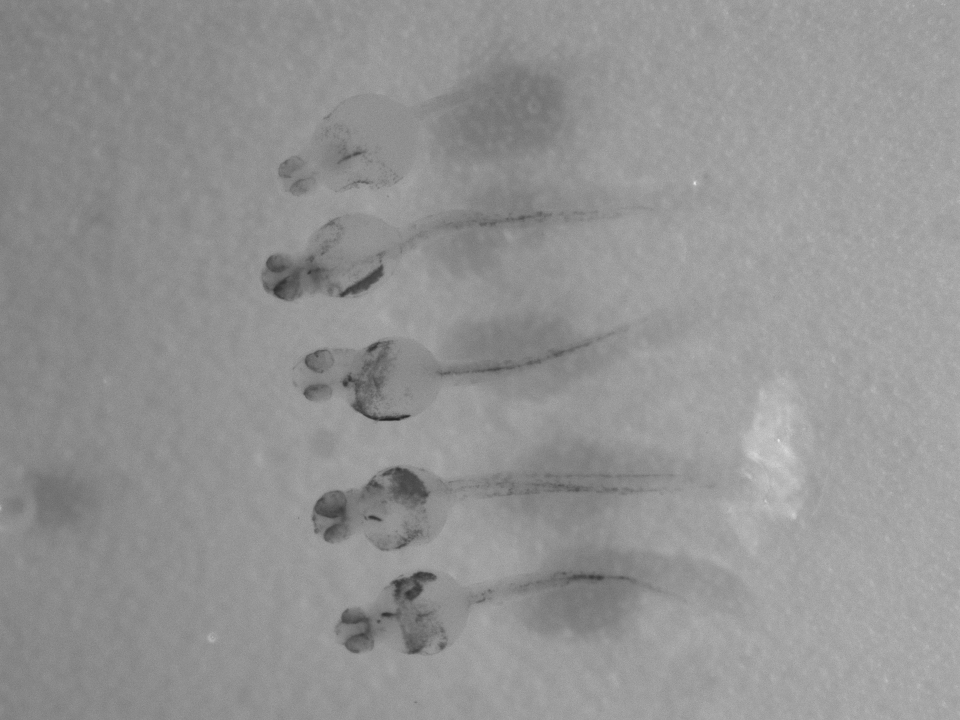

Supplement: Supplementary file 8 — Source data Fig. 4 [file 44321_2025_368_MOESM8_ESM.zip › FIGURE_4/4G/cRNA_rps19_DMSO (4).tif]

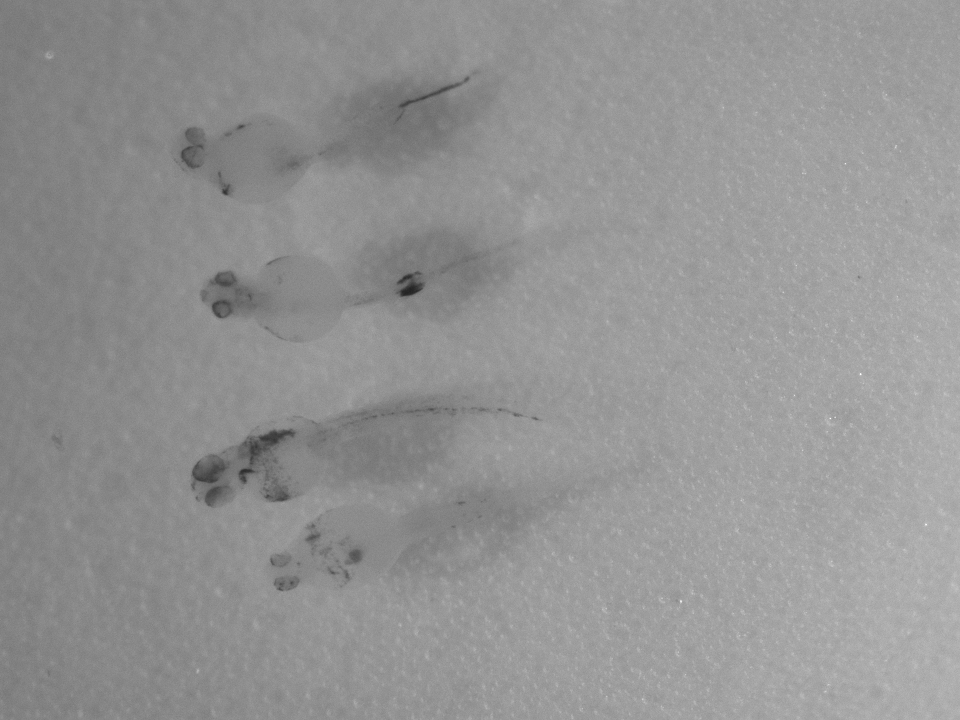

Supplement: Supplementary file 8 — Source data Fig. 4 [file 44321_2025_368_MOESM8_ESM.zip › FIGURE_4/4G/cRNA_rps19_DMSO (5).tif]

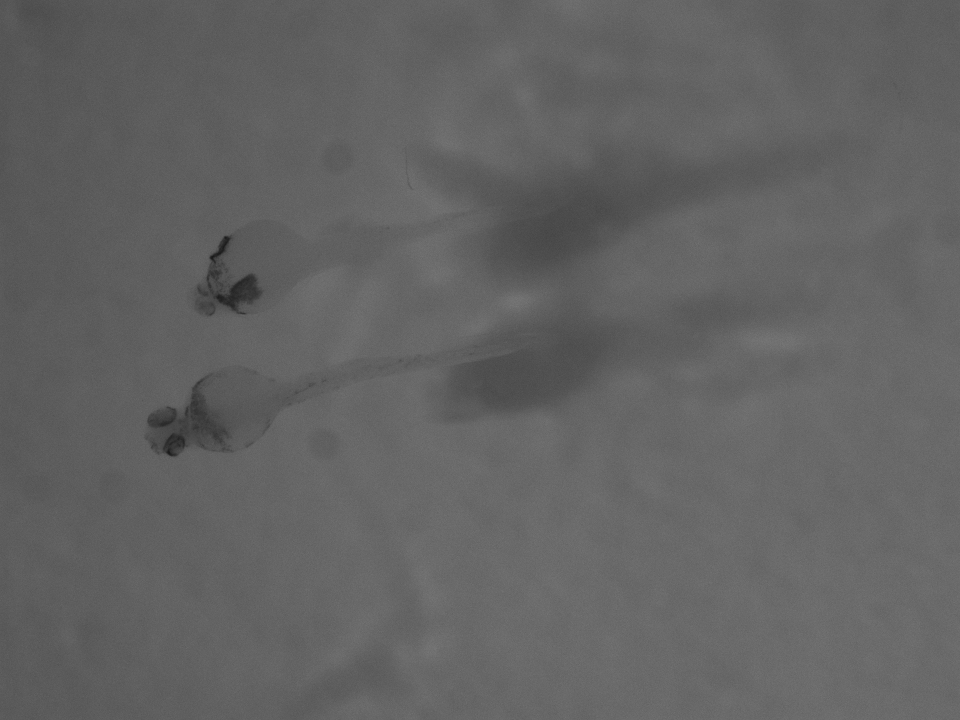

Supplement: Supplementary file 8 — Source data Fig. 4 [file 44321_2025_368_MOESM8_ESM.zip › FIGURE_4/4G/crRNA_rps19_bosutinib_1uM (1).tif]

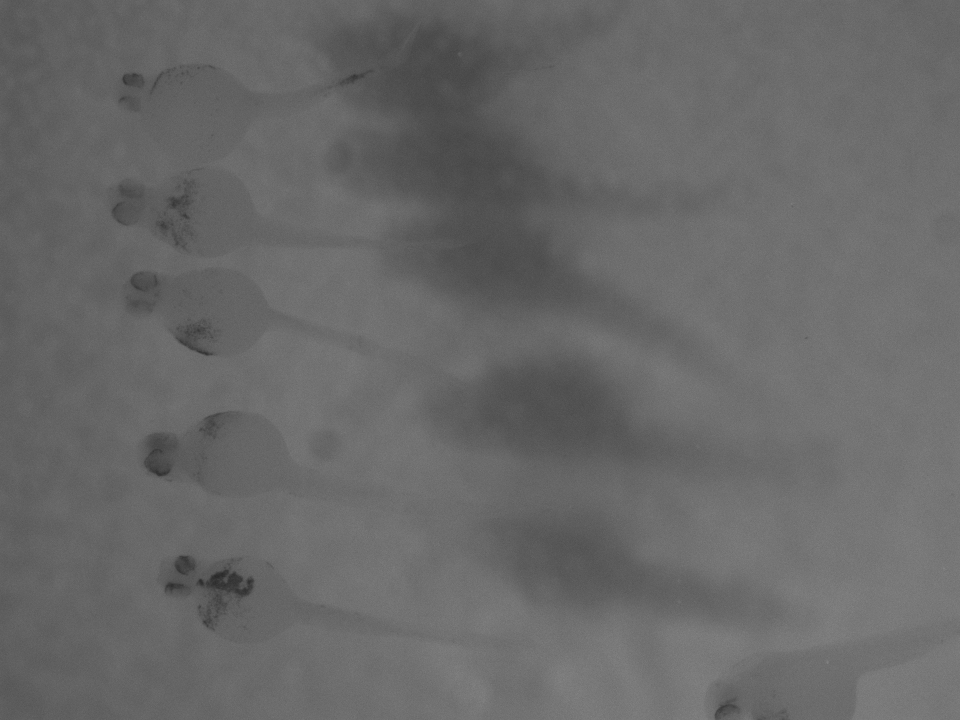

Supplement: Supplementary file 8 — Source data Fig. 4 [file 44321_2025_368_MOESM8_ESM.zip › FIGURE_4/4G/crRNA_rps19_bosutinib_1uM (2).tif]

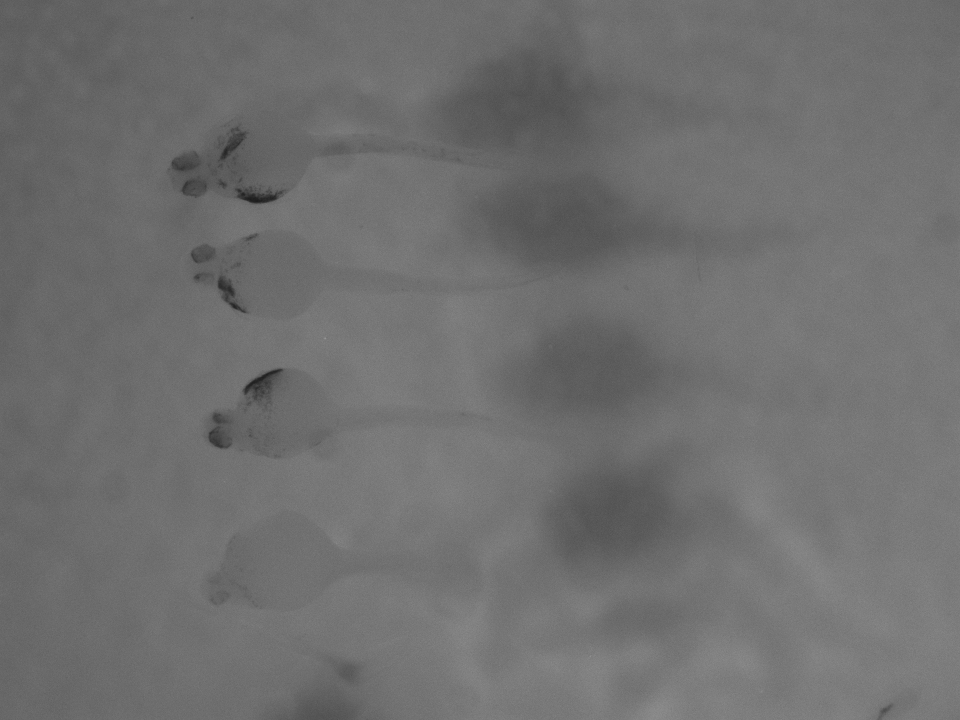

Supplement: Supplementary file 8 — Source data Fig. 4 [file 44321_2025_368_MOESM8_ESM.zip › FIGURE_4/4G/crRNA_rps19_bosutinib_1uM (3).tif]

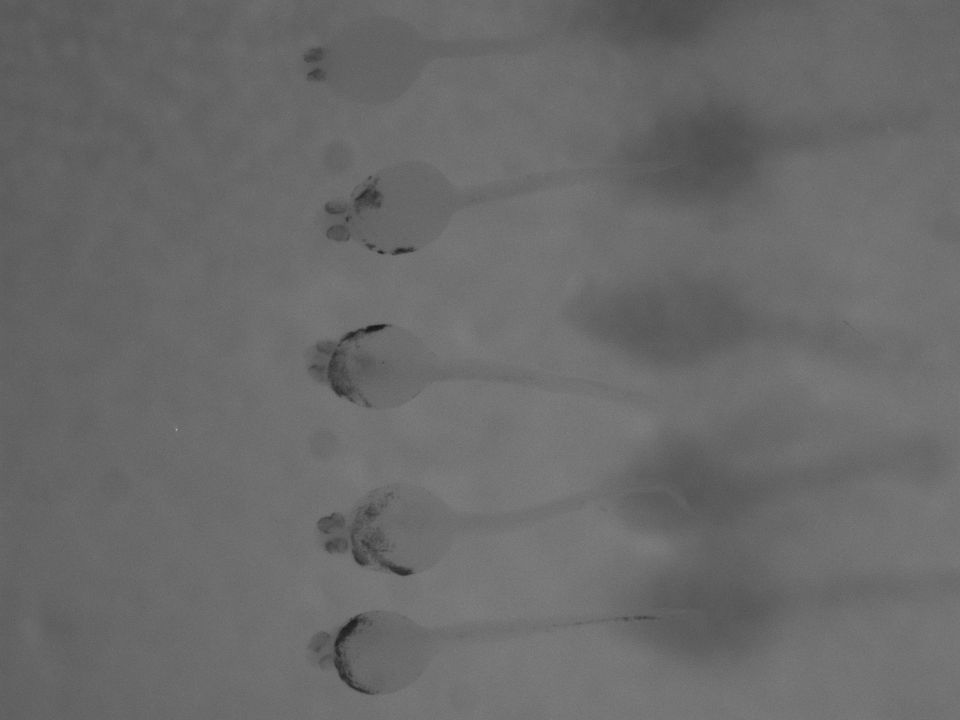

Supplement: Supplementary file 8 — Source data Fig. 4 [file 44321_2025_368_MOESM8_ESM.zip › FIGURE_4/4G/crRNA_rps19_bosutinib_1uM (4).tif]

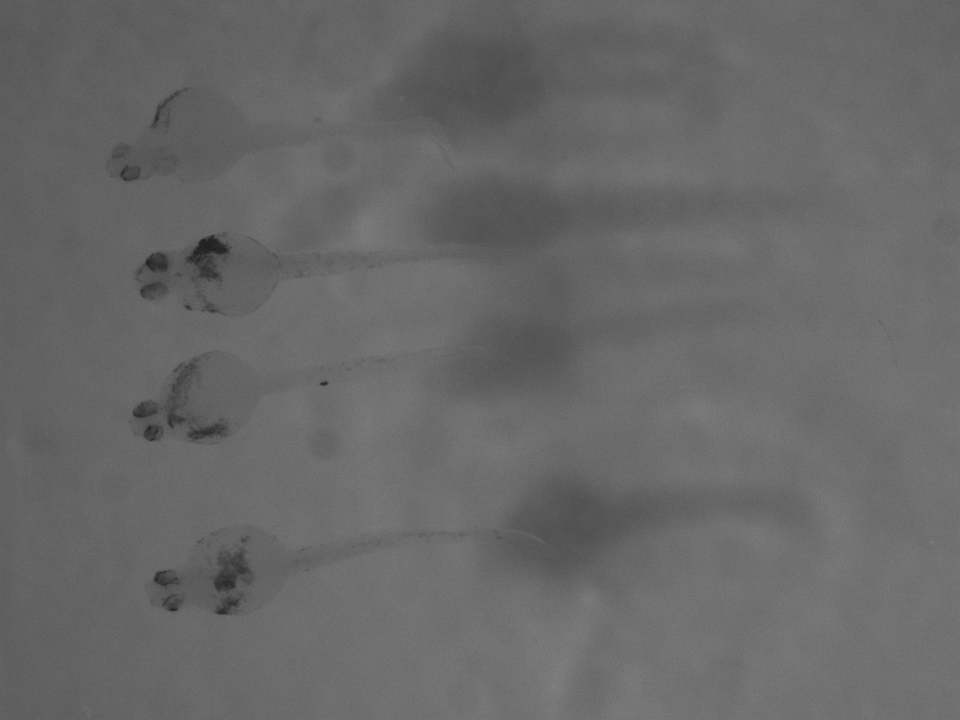

Supplement: Supplementary file 8 — Source data Fig. 4 [file 44321_2025_368_MOESM8_ESM.zip › FIGURE_4/4G/crRNA_rps19_bosutinib_1uM (5).tif]

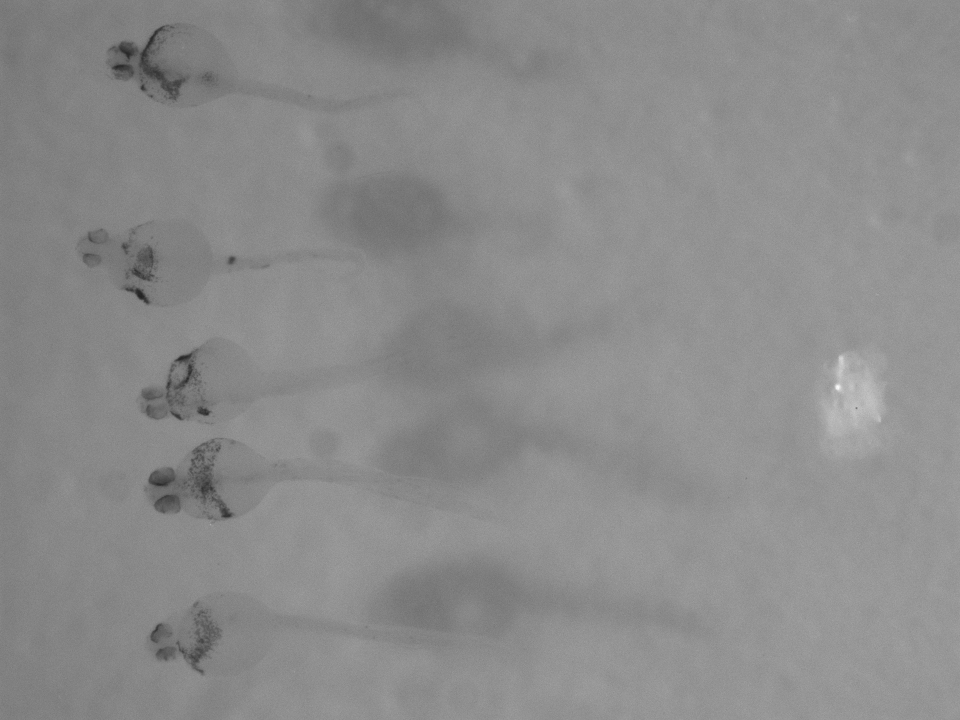

Supplement: Supplementary file 8 — Source data Fig. 4 [file 44321_2025_368_MOESM8_ESM.zip › FIGURE_4/4G/crRNA_rps19_dasatinib_1uM (1).tif]

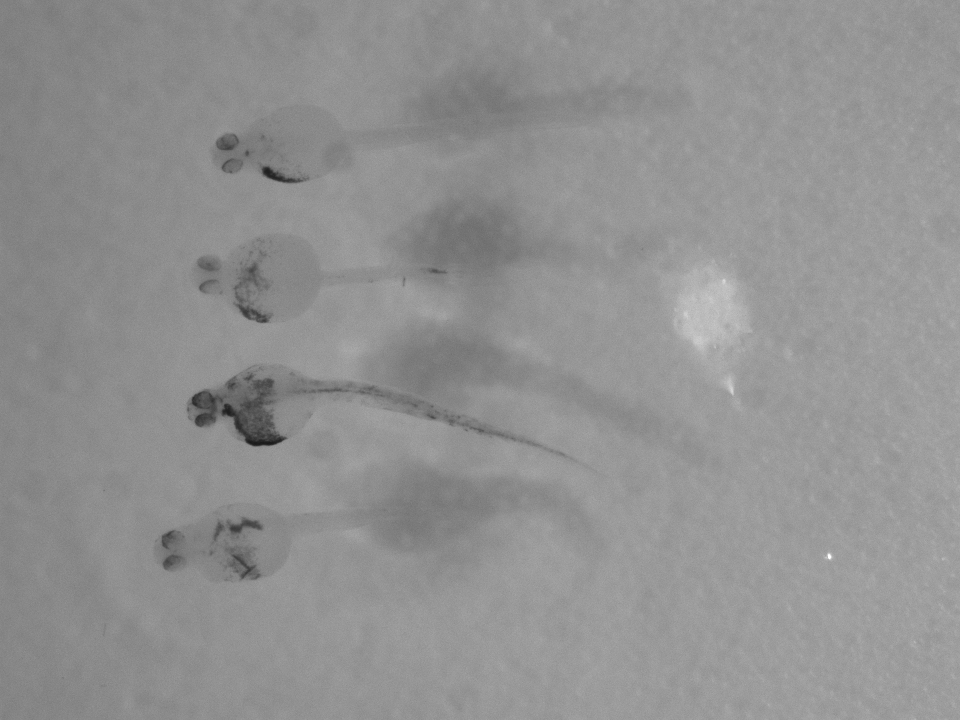

Supplement: Supplementary file 8 — Source data Fig. 4 [file 44321_2025_368_MOESM8_ESM.zip › FIGURE_4/4G/crRNA_rps19_dasatinib_1uM (2).tif]

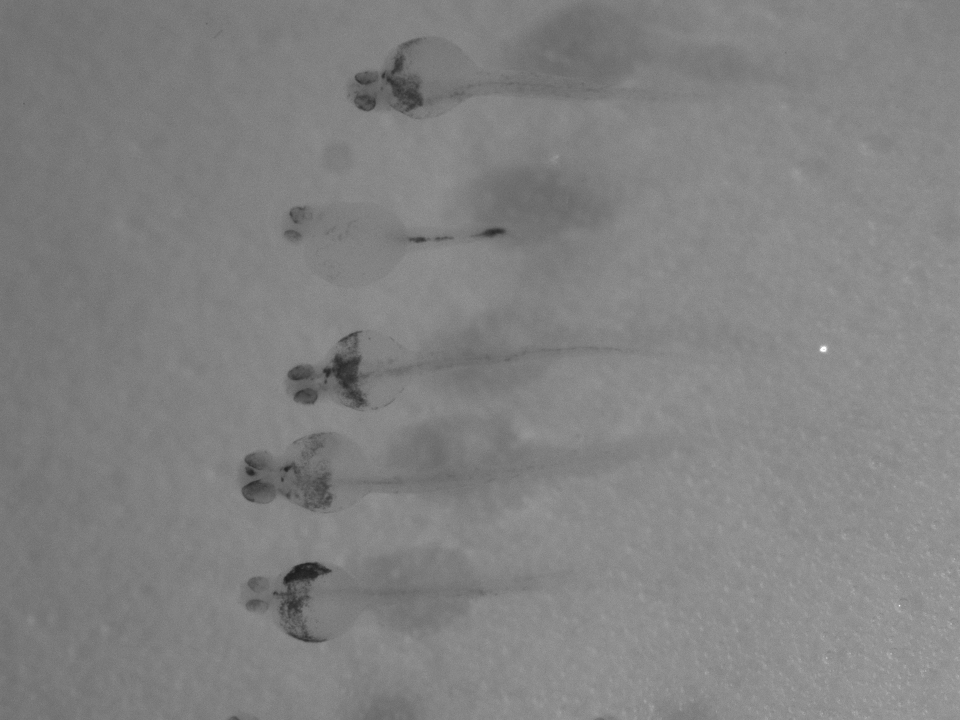

Supplement: Supplementary file 8 — Source data Fig. 4 [file 44321_2025_368_MOESM8_ESM.zip › FIGURE_4/4G/crRNA_rps19_dasatinib_1uM (3).tif]

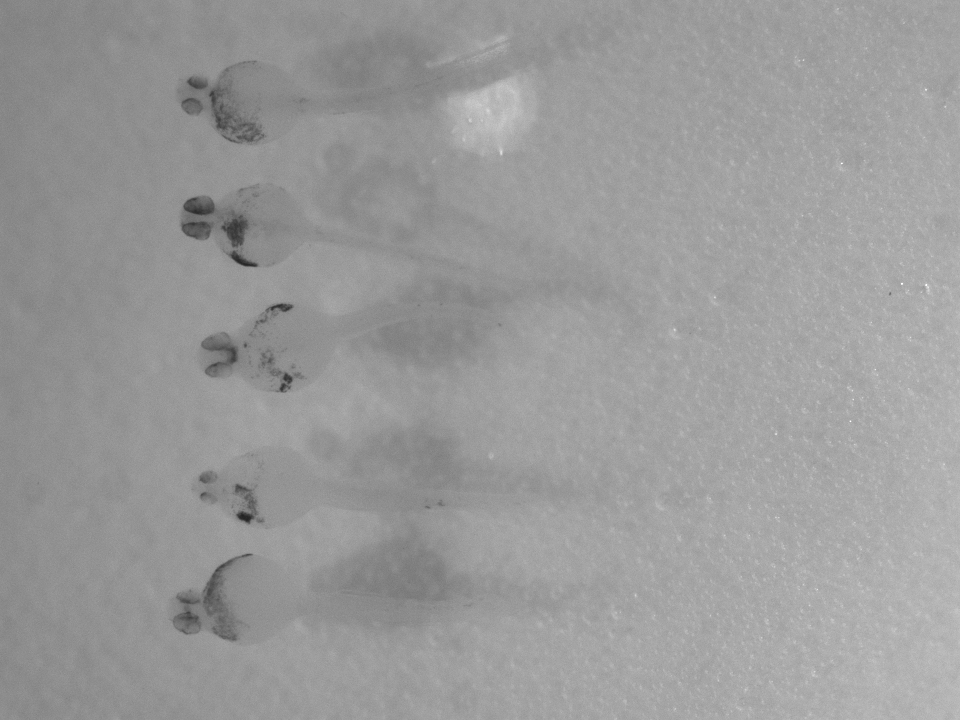

Supplement: Supplementary file 8 — Source data Fig. 4 [file 44321_2025_368_MOESM8_ESM.zip › FIGURE_4/4G/crRNA_rps19_dasatinib_1uM (4).tif]

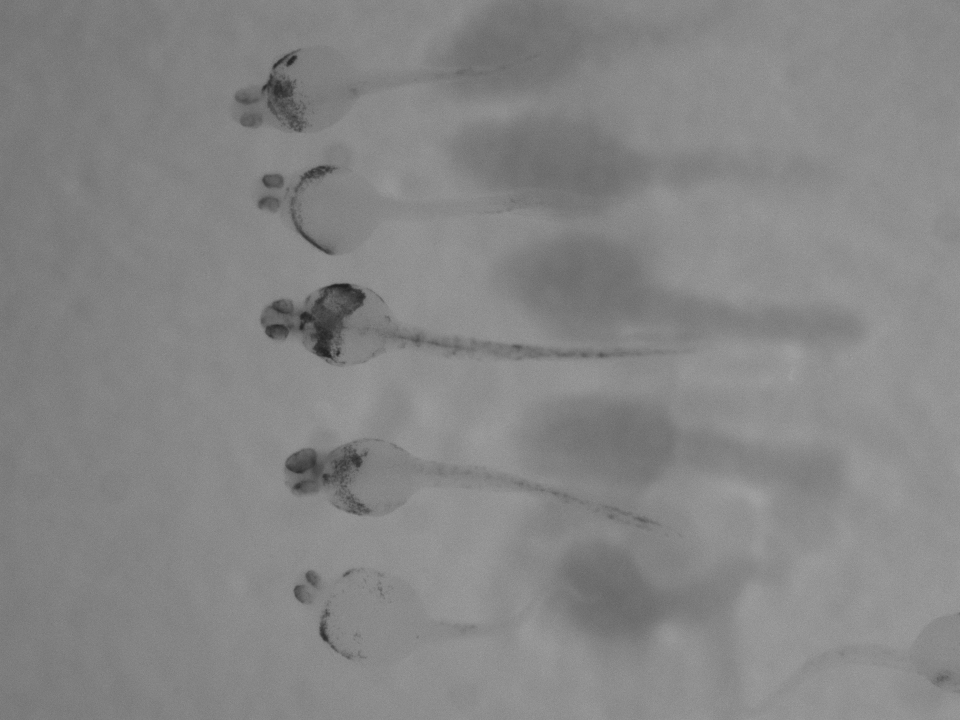

Supplement: Supplementary file 8 — Source data Fig. 4 [file 44321_2025_368_MOESM8_ESM.zip › FIGURE_4/4G/crRNA_rps19_dasatinib_1uM (5).tif]

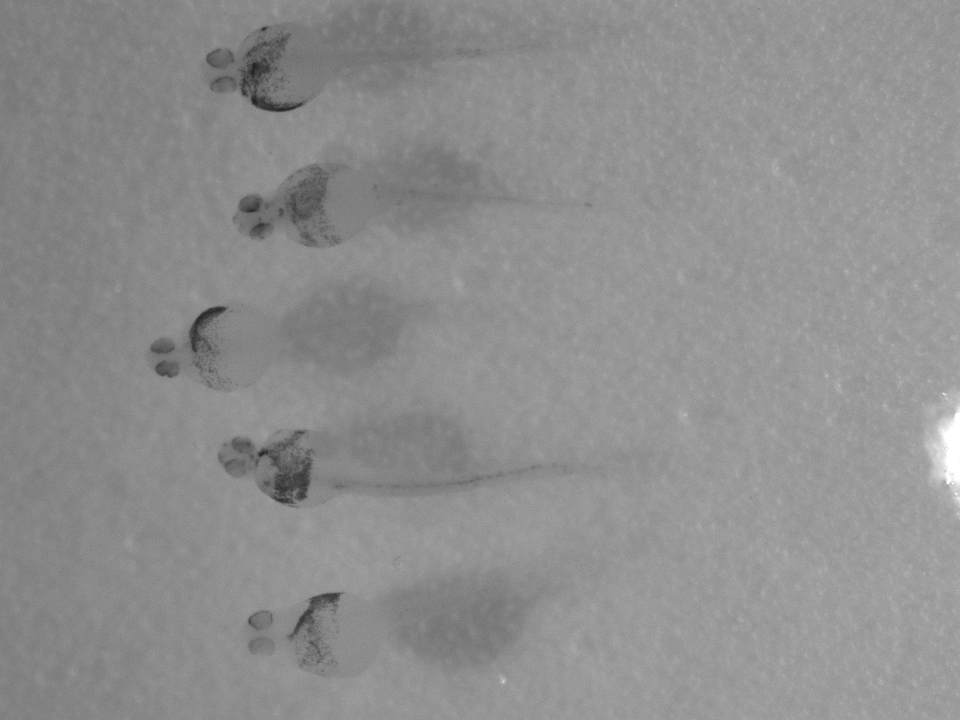

Supplement: Supplementary file 8 — Source data Fig. 4 [file 44321_2025_368_MOESM8_ESM.zip › FIGURE_4/4G/crRNA_rps19_imatinib_1uM (1).tif]

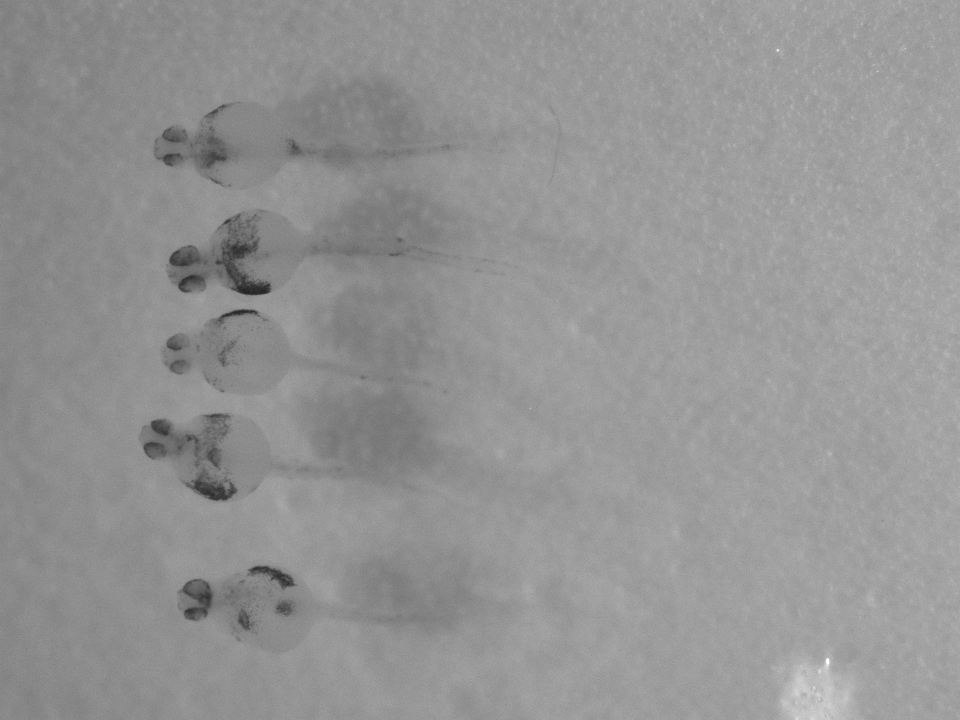

Supplement: Supplementary file 8 — Source data Fig. 4 [file 44321_2025_368_MOESM8_ESM.zip › FIGURE_4/4G/crRNA_rps19_imatinib_1uM (2).tif]

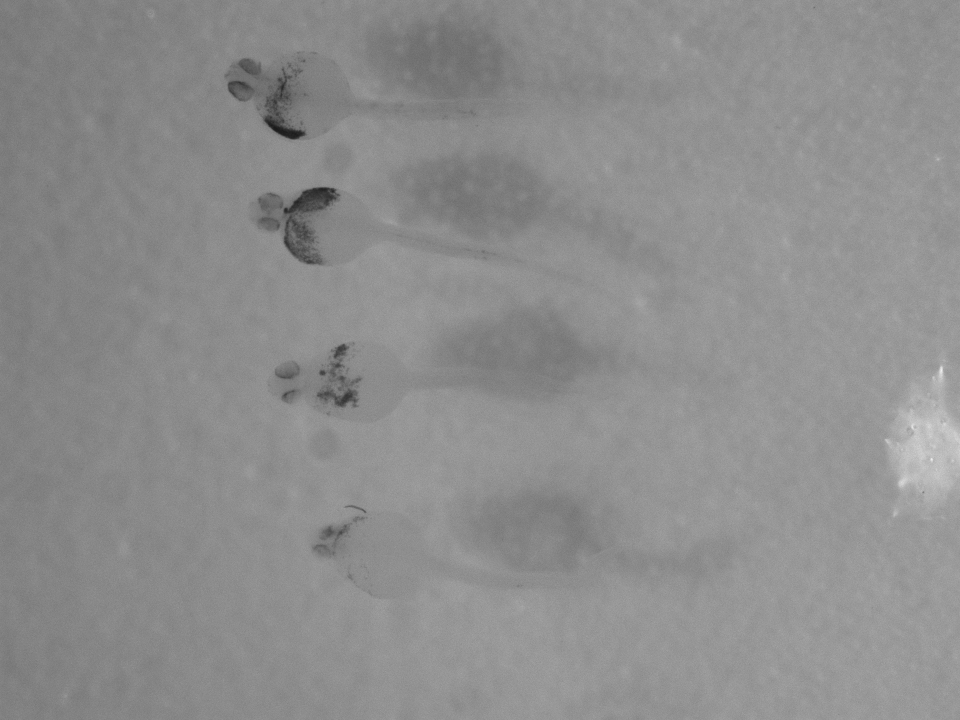

Supplement: Supplementary file 8 — Source data Fig. 4 [file 44321_2025_368_MOESM8_ESM.zip › FIGURE_4/4G/crRNA_rps19_imatinib_1uM (3).tif]

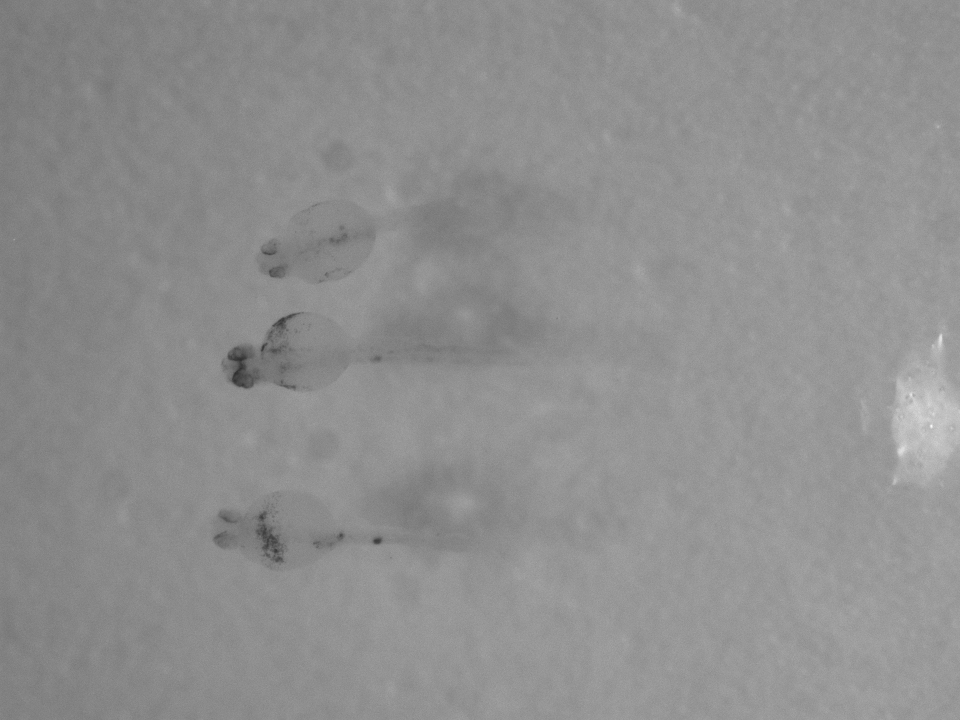

Supplement: Supplementary file 8 — Source data Fig. 4 [file 44321_2025_368_MOESM8_ESM.zip › FIGURE_4/4G/crRNA_rps19_imatinib_1uM (4).tif]

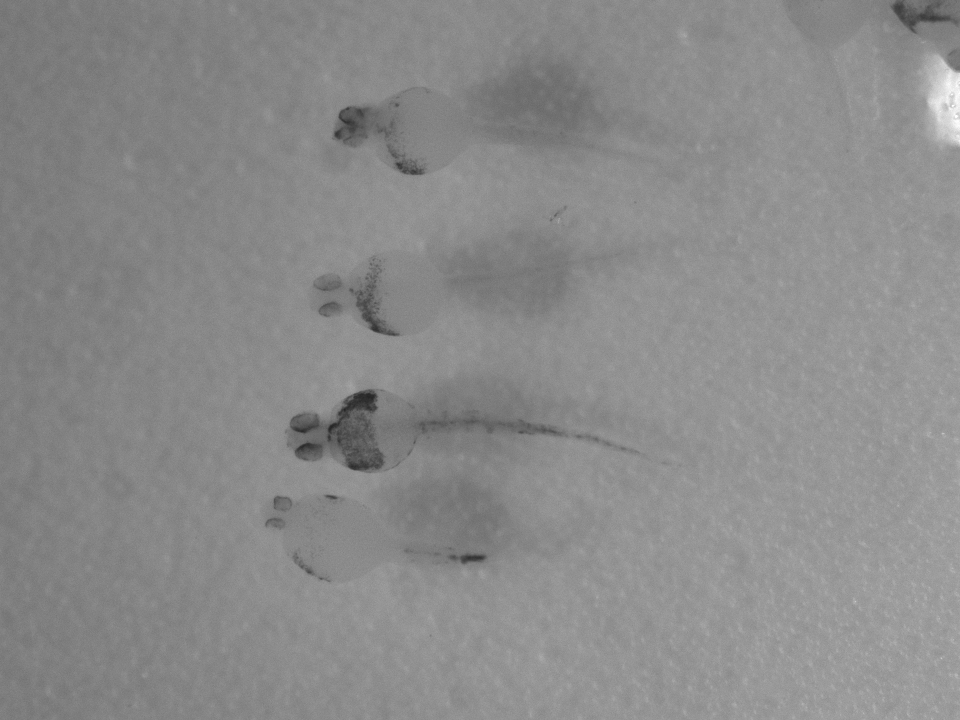

Supplement: Supplementary file 8 — Source data Fig. 4 [file 44321_2025_368_MOESM8_ESM.zip › FIGURE_4/4G/crRNA_rps19_imatinib_1uM (5).tif]

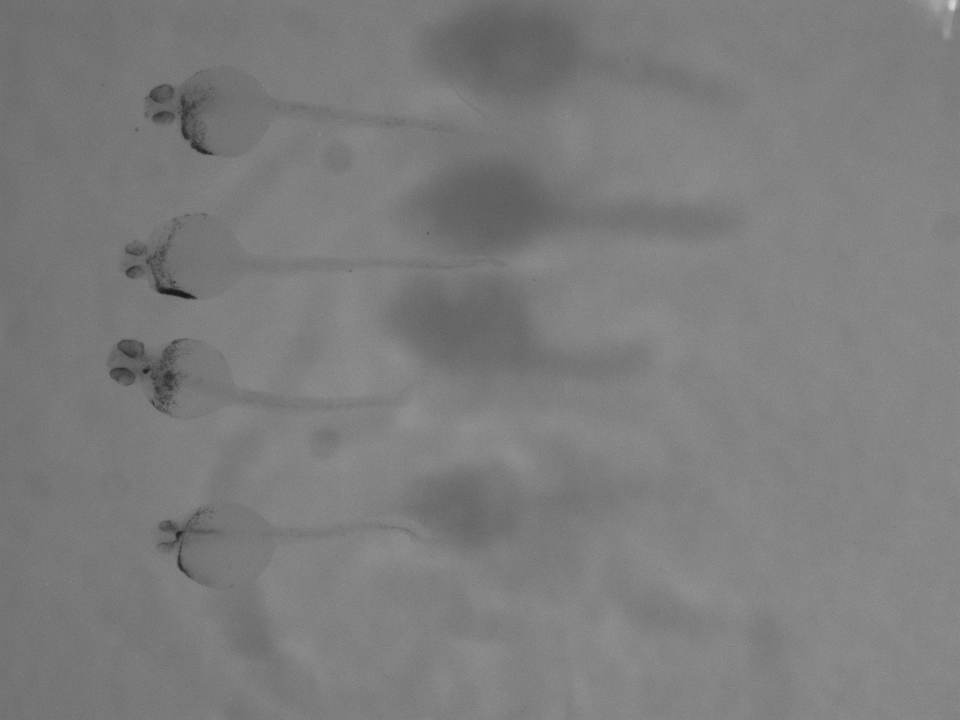

Supplement: Supplementary file 8 — Source data Fig. 4 [file 44321_2025_368_MOESM8_ESM.zip › FIGURE_4/4G/crRNA_rps19_nilotinib_1uM (1).tif]

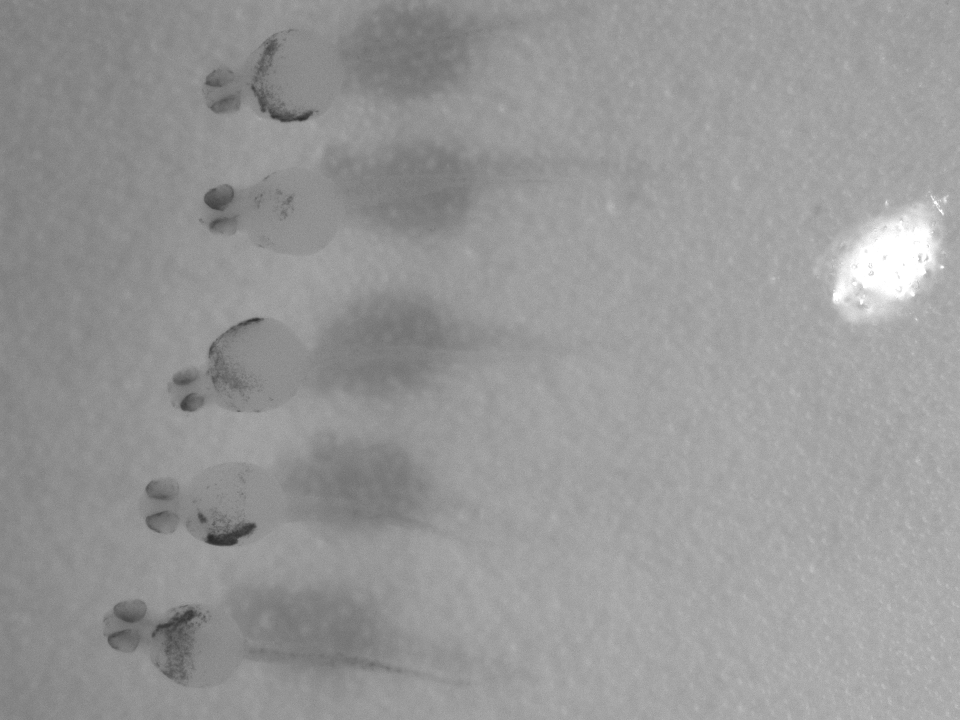

Supplement: Supplementary file 8 — Source data Fig. 4 [file 44321_2025_368_MOESM8_ESM.zip › FIGURE_4/4G/crRNA_rps19_nilotinib_1uM (2).tif]
