# Supplementary material for: TKI-mediated inhibition of NLRP1 inflammasome restores erythropoiesis in DBA syndrome
Source: EMBO Mol Med. 2026 Jan 9;18(2):702–24. doi: 10.1038/s44321-025-00368-3 (PMC12905221; doi:10.1038/s44321-025-00368-3)
Supplement: Supplementary file 9 — Source data Fig. 5 [file 44321_2025_368_MOESM9_ESM.zip › FIGURE_5/5C.pptx]

## Slide 1
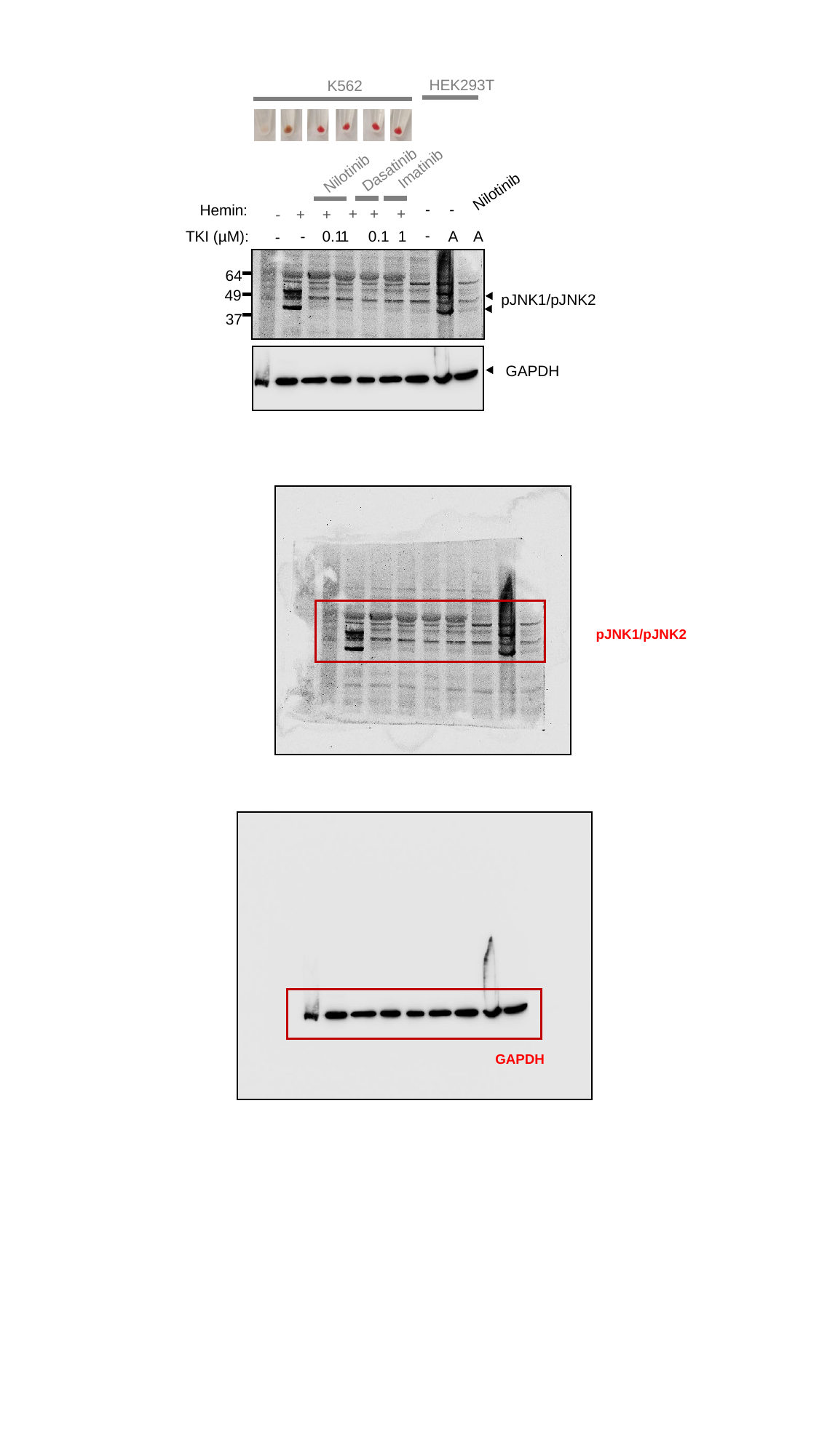

HEK293T
K562
Imatinib
Dasatinib
Nilotinib
Nilotinib
-
-
Hemin:
+
+
+
-
+
+
-
-
0.1
1
0.1
1
A
A
 TKI (µM):
-
64
49
37
pJNK1/pJNK2
GAPDH
pJNK1/pJNK2
GAPDH
