# Supplementary material for: TKI-mediated inhibition of NLRP1 inflammasome restores erythropoiesis in DBA syndrome
Source: EMBO Mol Med. 2026 Jan 9;18(2):702–24. doi: 10.1038/s44321-025-00368-3 (PMC12905221; doi:10.1038/s44321-025-00368-3)
Supplement: Supplementary file 9 — Source data Fig. 5 [file 44321_2025_368_MOESM9_ESM.zip › FIGURE_5/5D.pptx]

## Slide 1
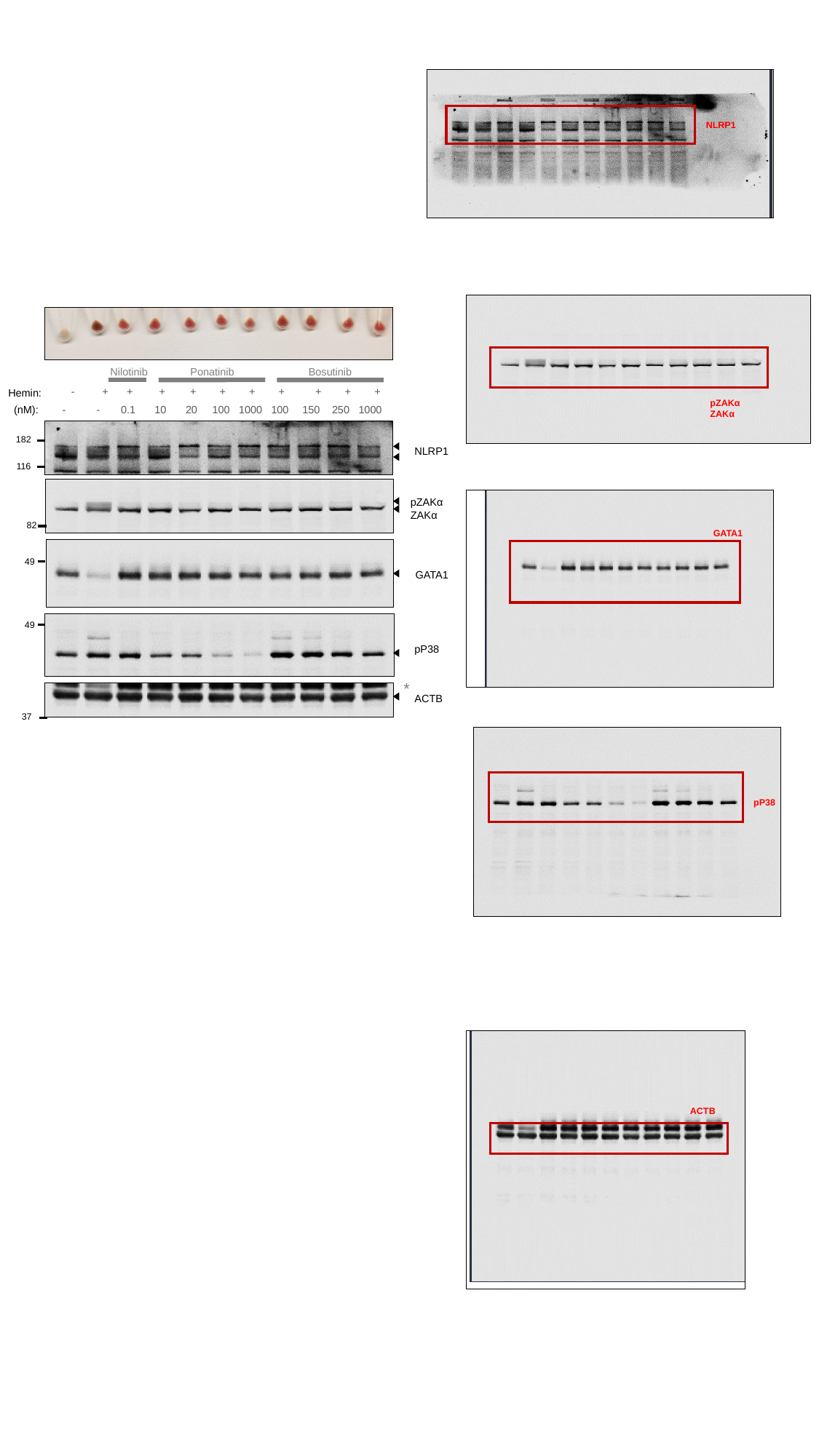

NLRP1
Nilotinib
Ponatinib
Bosutinib
-
+
+
+
+
+
+
+
+
+
+
Hemin:
 (nM):
-
-
0.1
10
20
100
1000
100
150
250
1000
182
NLRP1
116
pZAKα
ZAKα
82
49
GATA1
49
pP38
ACTB
37
*
pZAKα
ZAKα
GATA1
pP38
ACTB
