# Supplementary material for: TKI-mediated inhibition of NLRP1 inflammasome restores erythropoiesis in DBA syndrome
Source: EMBO Mol Med. 2026 Jan 9;18(2):702–24. doi: 10.1038/s44321-025-00368-3 (PMC12905221; doi:10.1038/s44321-025-00368-3)
Supplement: Supplementary file 17 — Expanded View Figures [file 44321_2025_368_MOESM17_ESM.pdf]

Expanded View Figures

Figure EV1. (related to Figs. 2 and 6). TKIs increased GATA1 protein amount during the erythroid differentiation of primary $CD34^+$ HSPCs.

(**A**) Primary human $CD34^+$ cells were purified from human cord blood and differentiated for 7 days with EPO in the presence of either DMSO, 100 nM nilotinib, 100 nM imatinib or 1 nM dasatinib. (**B**, **C**) Cells were stained with anti-GATA1-APC and $GATA1^{high}$ cells analyzed by flow cytometry at 3 and 7 days post-differentation. Representative dot plots at different differentiation times are also shown. Data are shown as the mean ± SEM. *P* values were calculated using one-way ANOVA and Tukey's multiple range test. All the significant comparisons have a $p < 0.0001$****. Source data are available online for this figure.

 © The Author(s)

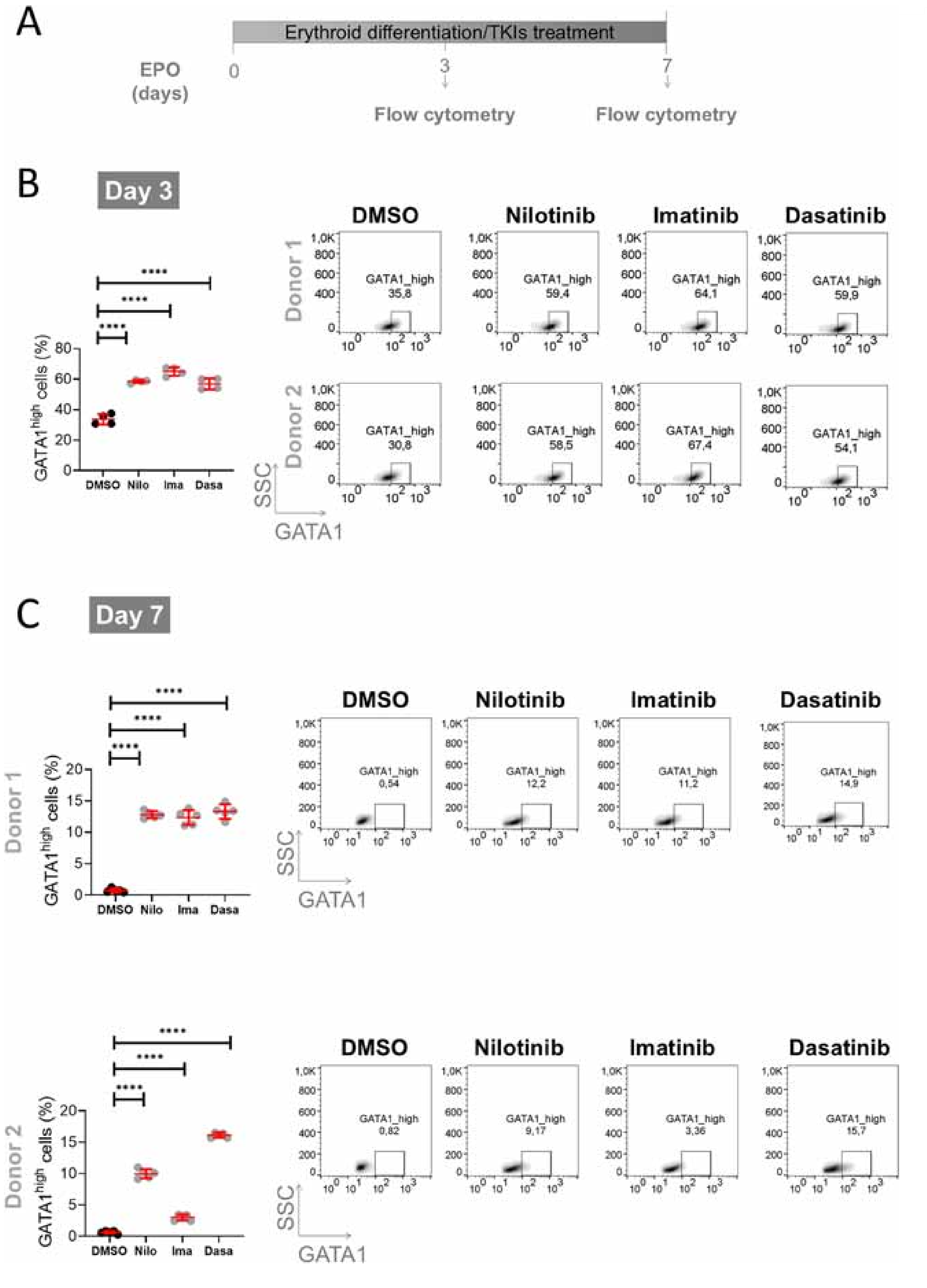
A
Erythroid differentiation/TKIs treatment
EPO (days)
0
3
7
Flow cytometry
Flow cytometry
B
Day 3
DMSO
Nilotinib
Imatinib
Dasatinib
Donor 1
Donor 2
GATA1_high 35,8
GATA1_high 59,4
GATA1_high 64,1
GATA1_high 59,9
GATA1_high 30,8
GATA1_high 58,5
GATA1_high 67,4
GATA1_high 54,1
GATA1high cells (%)
DMSO Nilo Ima Dasa
****
SSC
GATA1
C
Day 7
Donor 1
GATA1_high 0,54
GATA1_high 12,2
GATA1_high 11,2
GATA1_high 14,9
Donor 2
GATA1_high 0,82
GATA1_high 9,17
GATA1_high 3,36
GATA1_high 15,7

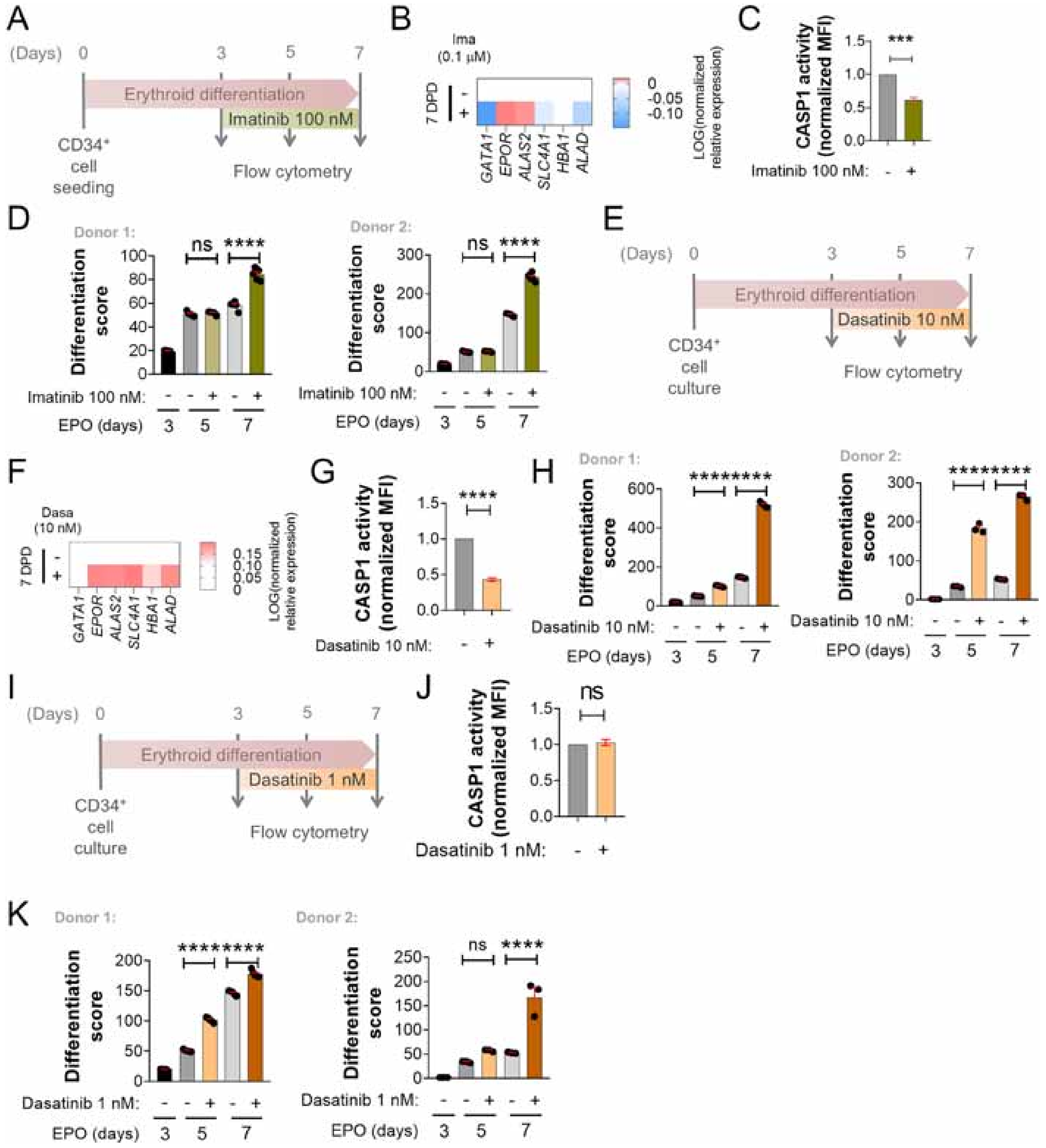
A
(Days) 0 3 5 7
Erythroid differentiation
Imatinib 100 nM
CD34+ cell seeding
Flow cytometry
B
Ima (0.1 μM)
7 DPD - +
GATA1 EPOR ALAS2 SLC4A1 HBA1 ALAD
0 -0.05 -0.10
LOG(normalized relative expression)
C
CASP1 activity (normalized MFI)
***
Imatinib 100 nM: - +
D
Donor 1:
Donor 2:
Differentiation score
ns ****
Imatinib 100 nM: - - + - +
EPO (days) 3 5 7
E
(Days) 0 3 5 7
Erythroid differentiation
Dasatinib 10 nM
CD34+ cell culture
Flow cytometry
F
Dasa (10 nM)
7 DPD - +
GATA1 EPOR ALAS2 SLC4A1 HBA1 ALAD
0.15 0.10 0.05 0
LOG(normalized relative expression)
G
CASP1 activity (normalized MFI)
****
Dasatinib 10 nM: - +
H
Donor 1:
Donor 2:
Differentiation score
**** ****
Dasatinib 10 nM: - - + - +
EPO (days) 3 5 7
I
(Days) 0 3 5 7
Erythroid differentiation
Dasatinib 1 nM
CD34+ cell culture
Flow cytometry
J
CASP1 activity (normalized MFI)
ns
Dasatinib 1 nM: - +
K
Donor 1:
Donor 2:
Differentiation score
**** ****
ns ****
Dasatinib 1 nM: - - + - +
EPO (days) 3 5 7

Figure EV2. (related to Figs. 2 and 6). Imatinib and dasatinib replicate the effects of nilotinib on human HSPCs from healthy donors.

Primary human CD34^{+} HSPCs from healthy donors (ZenBio or StemCell Technologies) were differentiated with EPO in the presence of 0.1 µM imatinib (**A–D**), or 10 (**E–H**) and 1 nM (**I–K**) dasatinib from 3 to 7 days of culture. Cells were stained with anti-CD235A-APC (Glycophorin A) and anti-CD71-FITC (Transferrin Receptor), and erythroid differentiation was then analyzed by flow cytometry. The transcript levels of GATA1-dependent genes (**B**), caspase-1 activity determined with FAM FLICA (**D**, **G**) and the differentiation score calculated as the ratio between CD235A+/CD71+ (intermediate erythroid progenitors) and CD235A-/CD71+ (early erythroid progenitors) (**D**, **H**, **K**) at 7 dpd are shown. Data are shown as the mean ± SEM ($N = 3$). P values were calculated using one-way ANOVA and Tukey's multiple range test (**D**, **H**, **K**) or a Student's t-test (**C**, **G**). ns, non-significant; *$p < 0.05$; **$p < 0.01$; ***$p < 0.01$ and ****$p < 0.0001$. (**C**): $p = 0.0005$***, (**D**): (Donor 1) DMSO_D7 wrt IMA_D7 $p < 0.0001$, (Donor 2) DMSO_D7 wrt IMA_D7 $p < 0.0001$, (**G**): $p < 0.0001$****, (**H**): DMSO_D5 wrt DASA_D5 $p < 0.0001$****, DMSO_D7 wrt DASA_D7 $p < 0.0001$****, (**K**): DMSO_D5 wrt DASA_D5 $p < 0.0001$****, DMSO_D7 wrt DASA_D7 $p < 0.0001$****. Source data are available online for this figure.

© The Author(s)

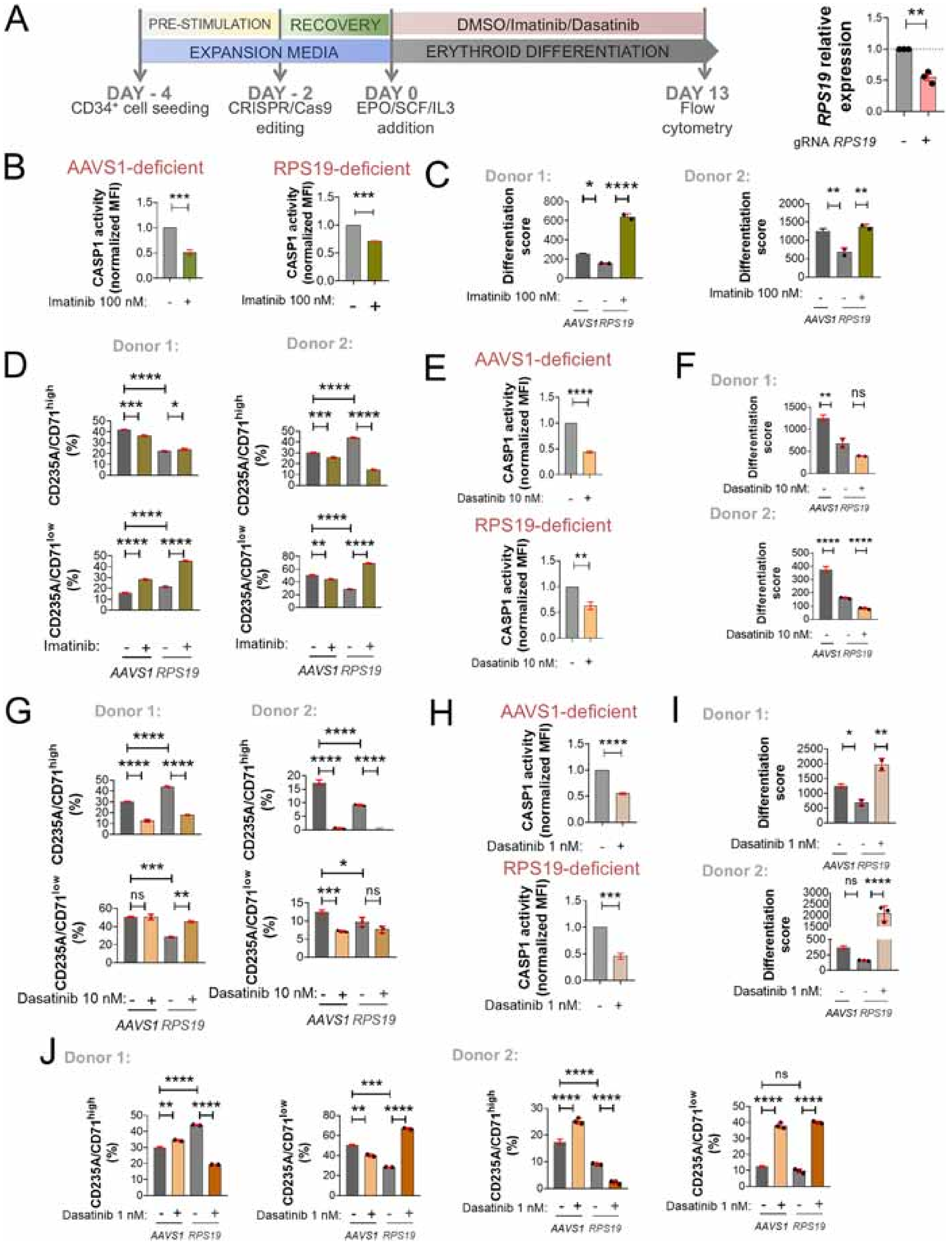
A
PRE-STIMULATION
RECOVERY
DMSO/Imatinib/Dasatinib
EXPANSION MEDIA
ERYTHROID DIFFERENTIATION
DAY - 4
CD34+ cell seeding
DAY - 2
CRISPR/Cas9 editing
DAY 0
EPO/SCF/IL3 addition
DAY 13
Flow cytometry
RPS19 relative expression
gRNA RPS19 - +
B
AAVS1-deficient
RPS19-deficient
CASP1 activity (normalized MFI)
Imatinib 100 nM: - +
C
Donor 1:
Donor 2:
Differentiation score
Imatinib 100 nM: - - +
AAVS1 RPS19
D
Donor 1:
Donor 2:
CD235A/CD71high (%)
CD235A/CD71low (%)
Imatinib: - + - +
AAVS1 RPS19
E
AAVS1-deficient
RPS19-deficient
CASP1 activity (normalized MFI)
Dasatinib 10 nM: - +
F
Donor 1:
Donor 2:
Differentiation score
Dasatinib 10 nM: - - +
AAVS1 RPS19
G
Donor 1:
Donor 2:
CD235A/CD71high (%)
CD235A/CD71low (%)
Dasatinib 10 nM: - + - +
AAVS1 RPS19
H
AAVS1-deficient
RPS19-deficient
CASP1 activity (normalized MFI)
Dasatinib 1 nM: - +
I
Donor 1:
Donor 2:
Differentiation score
Dasatinib 1 nM: - - +
AAVS1 RPS19
J
Donor 1:
Donor 2:
CD235A/CD71high (%)
CD235A/CD71low (%)
Dasatinib 1 nM: - + - +
AAVS1 RPS19

Figure EV3. (related to Figs. 2 and 6). Imatinib and dasatinib alleviate defective erythropoiesis of RPS19-deficient HSPCs.

(**A**) Primary human CD34+ from healthy donors (ZenBio or StemCell Technologies) were edited with CRISPR/Cas9 and differentiated for 13 days with EPO in the presence of of 0.1 µM imatinib (**A–D**) or 10 nM dasatinib (**E–J**) from 3 to 13 days of culture. Cells were stained with either FAM FLICA or anti-CD235A-APC (Glycophorin A) and anti-CD71-FITC (Transferrin Receptor), and CASP1 activity (**B**, **H**), erythroid differentiation (**B–D**, **F**, **G**, **I**, **J**) were then analyzed by flow cytometry. The differentiation score was calculated as the ratio between CD235A + /CD71+ (intermediate erythroid progenitors) and CD235A-/CD71+ (early erythroid progenitors) (**C**, **F**, **I**), and the percentage of CD235A + /CD71high (erythroblasts) and CD235A + /CD71low (reticulocytes) (**D**, **G**, **J**) at 13 days of culture (**E**). Data are shown as the mean ± SEM ($N = 3$). *P* values were calculated using one-way ANOVA and Tukey's multiple range test (**C**, **D**, **F**, **G**, **I**, **J**) or a Student's *t*-test (**A**, **B**, **E**, **H**). ns, non-significant; *$p < 0.05$; **$p < 0.01$; ***$p < 0.01$ and ****$p < 0.0001$. (**A**): $p = 0.0011$**, (**B**): AAVS1-deficient $p = 0.0009$***, RPS19-deficient p = 0.0009***, (**C**): (donor 1) AAVS1_DMSO wrt RPS19_DMSO $p = 0.0271$*, RPS19_DMSO wrt RPS19_IMA $p = 0.0002$***, (donor 2) AAVS1_DMSO wrt RPS19_DMSO $p = 0.0043$**, RPS19_DMSO wrt RPS19_IMA $p = 0.0034$**, (**D**): (Donor 1) (Upper graph) AAVS1_DMSO wrt: AAVS1_IMA $p = 0.0005$*** or RPS19_DMSO $p < 0.0001$****, RPS19_DMSO wrt RPS19_IMA $p = 0.0446$*, (lower graph) all $p < 0.0001$****, (Donor 2) (Upper graph) AAVS1_DMSO wrt: AAVS1_IMA $p = 0.0006$*** or RPS19_DMSO p < 0.0001****, RPS19_DMSO wrt RPS19_IMA $p < 0.0001$****, (lower graph) AAVS1_DMSO wrt: AAVS1_IMA $p = 0.0012$** or RPS19_DMSO $p < 0.0001$****, RPS19_DMSO wrt RPS19_IMA $p < 0.0001$****, (**E**): AAVS1-deficient $p < 0.0001$****, RPS19-deficient $p = 0.0076$**, (**F**): (Donor 1) AAVS1_DMSO wrt RPS19_DMSO $p = 0.0012$**, (Donor 2) all significant comparison have $p < 0.0001$****, (**G**): (Donor 1) (upper graph) all significant differences have $p < 0.0001$****, (lower graph) AAVS1_DMSO wrt RPS19_DMSO $p = 0.0006$***. RPS19_DMSO wrt RPS19 $p = 0.0018$**, (Donor 2) (upper graph) all significant differences have $p < 0.0001$****, (lower graph) AAVS1_DMSO wrt: AAVS1_DASA $p = 0.0002$*** or RPS19_DMSO $p = 0.0142$*, (**H**): AAVS1-deficient $p < 0.0001$****, RPS19-deficient $p = 0.0006$***, (**I**): (Donor 1) AAVS1_DMSO wrt RPS19_DMSO $p = 0.0311$*, RPS19_DMSO wrt RPS19_DASA $p = 0.0015$ **, (Donor 2) RPS19_DMSO wrt RPS19_DASA $p < 0.0001$****, (**J**): (Donor 1) (left) AAVS1_DMSO wrt: AAVS1_DASA $p = 0.0012$** or RPS19_DMSO $p < 0.0001$****, RPS19_DMSO wrt RPS19_DASA $p < 0.0001$****, (right) AAVS1_DMSO wrt AAVS1_DASA $p = 0.0015$** or RPS19_DMSO $p = 0.0001$***, RSP19_DMSO wrt RPS19_DASA $p < 0.0001$****, (Donor 2) all significant differences have $p < 0.0001$****. Source data are available online for this figure.

© The Author(s)
